# Discovery and biosynthesis of karnamicins as angiotensin converting enzyme inhibitors

**DOI:** 10.1038/s41467-023-35829-1

**Published:** 2023-01-13

**Authors:** Zhiyin Yu, Jian-Ping Huang, Jing Yang, Chongxi Liu, Yijun Yan, Li Wang, Junwei Zhao, Yin Chen, Wensheng Xiang, Sheng-Xiong Huang

**Affiliations:** 1 State Key Laboratory of Southwestern Chinese Medicine Resources, Innovative Institute of Chinese Medicine and Pharmacy, Chengdu University of Traditional Chinese Medicine, 611137 Chengdu, China; 2 Heilongjiang Provincial Key Laboratory of Agricultural Microbiology, Northeast Agricultural University, 150030 Harbin, China; 3 State Key Laboratory of Phytochemistry and Plant Resources in West China, and CAS Center for Excellence in Molecular Plant Sciences, Kunming Institute of Botany, Chinese Academy of Sciences, 650201 Kunming, China

**Keywords:** Oxidoreductases, Drug discovery and development, Biosynthesis

## Abstract

Angiotensin-converting enzyme inhibitors are widely used for treatment of hypertension and related diseases. Here, six karnamicins E_1_-E_6_ (**1**–**6**), which bear fully substituted hydroxypyridine and thiazole moieties are characterized from the rare actinobacterium *Lechevalieria rhizosphaerae* NEAU-A2. Through a combination of isotopic labeling, genome mining, and enzymatic characterization studies, the programmed assembly of the fully substituted hydroxypyridine moiety in karnamicin is proposed to be due to sequential operation of a hybrid polyketide synthase-nonribosomal peptide synthetase, two regioselective pyridine ring flavoprotein hydroxylases, and a methyltransferase. Based on AlphaFold protein structures predictions, molecular docking, and site-directed mutagenesis, we find that two pyridine hydroxylases deploy active site residues distinct from other flavoprotein monooxygenases to direct the chemo- and regioselective hydroxylation of the pyridine nucleus. Pleasingly, karnamicins show significant angiotensin-converting enzyme inhibitory activity with IC_50_ values ranging from 0.24 to 5.81 μM, suggesting their potential use for the treatment of hypertension and related diseases.

## Abstract (web summary)

Treatment of hypertension entails use of angiotensin-converting enzyme inhibitors. Here, the authors show a series of karnamicins with significant inhibitory activity and identify two unusual flavoprotein hydroxylases involved in the assembly of the fully-substituted hydroxypyridine core of karnamicins.

## Introduction

According to statistics from the World Health Organization (WHO), cardiovascular diseases (CVDs) are the leading cause of death globally, taking the lives of 17.9 million people in 2019. Perhaps more concerningly, 81% of these deaths occur in developing countries and over one third are defined as premature (i.e., 30–70 years of age)^1^. Hypertension, or elevated blood pressure, has been well recognized as the key risk factor for almost all CVDs acquired during life^2^. Of particular relevance to current public health measures, recent studies have also revealed that the prevalence of hypertension and CVD is clinically relevant in patients with COVID-19^3–5^. Angiotensin-converting enzyme (ACE), a zinc-dependent dipeptide carboxypeptidase, is responsible for elevating human blood pressure by catalyzing the production of potent vasoconstrictor angiotensin-II from inactive angiotensin-I, as well as the degradation of vasodilator bradykinin^6,7^. Inhibition of ACE is a modern therapeutic target in the treatment of hypertension and related CVDs^8,9^. However, the widely used ACE inhibitors, such as captopril and enalapril, have relatively high incidence of side effects^10–12^, hindering their broad clinical application. Considering the potential of microbial natural products in drug discovery^13^, mining natural ACE inhibitor candidates from microorganisms is a promising approach.

**Fig. 1:**
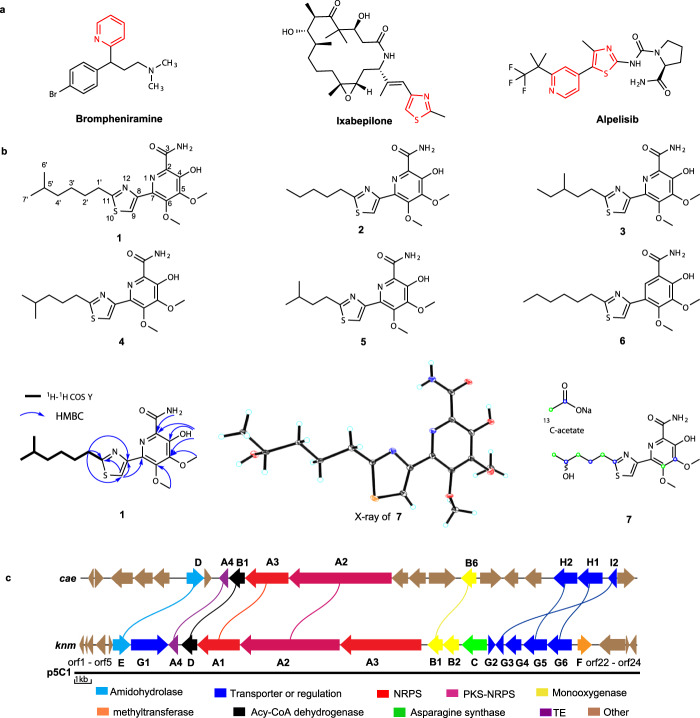
Structures of brompheniramine, ixabepilone, alpelisib, and 1–7 and organization of related genes in the *cae* and *knm* BGCs. **a** Pharmaceuticals containing thiazole and/or pyridine ring. **b** Structures of karnamicins **1**–**7**, key COSY and HMBC correlations of **1**, ORTEP representation (30% probability ellipsoids) of the X-ray structure of compound **7**, and isotope labeling result. **c** Comparison of the organization of the *cae* BGC from *A. cyanogriseus* strain NRRL B-2194 and *knm* BGC from *L. rhizosphaerae* NEAU-A2.

Pyridine, thiazole, and their bi-heteroaryl derivatives are common functionalities present in many clinically applied drugs, such as antihistamine brompheniramine, and anticancer agents ixabepilone and alpelisib^14–19^ (Fig. 1a). Notably, such moieties also form the core structure of the karnamicin natural products, a class of antibiotics originally isolated from the fermentation liquor of an actinobacterium, *Saccharothrix aerocolonigenes* N806-4, in 1989^20^. Although only weak antibacterial activity was found against Gram-positive bacteria^20^, the unique skeleton composed of fully substituted hydroxypyridine and thiazole moieties indicates that there could be a wealth of potential bioactivities. However, other than the total synthesis of karnamicin B_1_ achieved in 1997^21^, no further studies of these compounds have been carried out. Exploring the karnamicin biosynthetic assembly line and analyzing their biological activities (other than antimicrobial) will help broaden and deepen our understanding of the medicinal potential of thiazole pyridine hybrid compounds, and further develop clinically important drugs.

In this study, six karnamicins E_1_–E_6_ (**1**–**6**), together with five known karnamicins (**7**–**11**) are isolated from *Lechevalieria rhizosphaerae* NEAU-A2 (Fig. 1b), a rare actinobacterium isolated from rhizosphere soil of wheat (*Triticum aestivum*). Using ^13^C-labeled precursor feeding, genome sequencing, and in vivo gene knockout and in vitro enzyme activity assays, we here dissect the biosynthetic gene cluster (BGC) responsible for assembling karnamicins in *L. rhizosphaerae* NEAU-A2 (*knm*, Fig. 1c). Interestingly, an unusual hybrid polyketide synthase-nonribosomal peptide synthetase (PKS-NRPS) KnmA2 is found to cooperate with two flavoprotein monooxygenases (FPMOs) KnmB1 and KnmB2, and a methyltransferase KnmF for assembling the characteristic fully substituted hydroxypyridine core in karnamicins. Subsequent structure-function relationship analysis demonstrates that, although KnmB1 and KnmB2 behave like other known aromatic hydroxylases, phylogenetically distinct active-site residues are recruited to realize the regioselective hydroxylation of the pyridine system. Finally, strong ACE inhibitory activity is established for a range of karnamicins, including less substituted biosynthetic intermediates, highlighting the potential of karnamicins as drug leads for hypertension and related CVDs.

## Results

### Discovery and structural elucidation of karnamicins E_1_–E_6_ from *L. rhizosphaerae* NEAU-A2

Karnamicin E_1_ (**1**) was obtained as colorless amorphous powder, and its molecular formula C_18_H_25_N_3_O_4_S was determined by high-resolution electrospray ionization mass spectrometry (HRESIMS) data (*m/z* 380.1648 [M + H]^+^, Supplementary Fig. 1), corresponding to eight degrees of unsaturation. The ^1^H NMR spectrum and ^13^C NMR and HSQC spectra showed the resonances of 18 carbons, which were classified into eight aromatic quaternary carbons, one carbonyl carbon, four sp^3^ methylene carbons, one sp^3^ methine carbon, two methyl carbons, and two methoxy carbons (Supplementary Table 1 and Supplementary Figs. 2–5). ^1^H–^1^H correlation spectroscopy indicated the presence of one spin-coupling system, H-1′/H-2′/H-3′/H-4′/H-5′/H-6′(H-7′), as shown in Fig. 1b. Furthermore, the HMBC cross-peaks from O-Me-13 to C5, from O-Me-14 to C6, from NH to C2 and from OH-4 to C2/4/5 (Supplementary Fig. 6), revealed the presence of a 4-hydroxy-5,6-dimethoxy-pyridinecarboxamide moiety. Cross-peaks from H-9 to C-7/8/11 were observed in the HMBC spectrum (Supplementary Fig. 6), suggesting a 4-hydroxy-5,6-dimethoxy-7-thiazolyl-picolinamide moiety. Lastly, the 4-hydroxy-5,6-dimethoxy-7-thiazolyl-pyridinecarboxamide moiety and the alkyl chain were found to be attached at the C11 position, based on the key HMBC correlation from H-1′ to C11/C-9/C-8 (Fig. 1b). It is noteworthy that the assignment of compound **1** was confounded by the lack of clear HMBC correlations, largely due to the high fraction of quaternary carbons and heteroatoms. Fortunately, however, the crystal structure of the also isolated compound **7** enabled unambiguous structural confirmation of the karnamicins by analogy (Fig. 1b). The ^1^H and ^13^C NMR spectra of co-isolated **2**–**6** also showed high similarity to those of **1** and **7**, except for alkyl side chain substitutions (Fig. 1b). Their structures were then determined based on extensive analyses of spectroscopic data (Supplementary Tables 2–6 and Supplementary Figs. 7–37).

### Identification of the karnamicin biosynthetic gene cluster

Encouraged by their unique structural features, we set out to dissect the molecular basis for the biosynthetic assembly of karnamicins. Initially, an isotopic feeding study was conducted with *L. rhizosphaerae* NEAU-A2 using sodium [1-^13^C] and [2-^13^C] acetate and then spectrally analyzing the major product **7**. Treatment with [1-^13^C] acetate contributed to the enrichment in carbons C11, C5, C4′, and C2′ (Supplementary Fig. 38), while the ^13^C signals derived from [2-^13^C] acetate were found to be distributed among carbons C6, C1′, C3′, and C5′ (Supplementary Fig. 39), suggesting intact incorporation of one acetate unit into the pyridine ring moiety (C5 to C6) and head-to-tail incorporation of three acetate units into the alkyl chain and thiazole ring (C5′ to C1′ and C11, Fig. 1b). Further evidence supporting incorporation of intact acetate into the pyridine ring moiety was obtained by feeding [1, 2-^13^C_2_] acetate (Supplementary Fig. 40). The presence of the acetate unit in the pyridine ring strongly suggested that a PKS participates in pyridine ring construction in karnamicin biosynthetic pathway.

To gain further insight into the biosynthetic mechanism of the karnamicins, the genome of *L. rhizosphaerae* NEAU-A2 was sequenced and annotated using the prokaryotic genome annotation pipeline (GeneMarkS Version 2.0)^22^. Subsequent antiSMASH 4.0-^23^ and PRISM 3-based^24^ genome mining allowed the discovery of a potential BGC (*knm*) for synthesizing karnamicin in *L. rhizosphaerae* NEAU-A2. The *knm* BGC consisted of 16 open reading frames (ORFs), encoding proteins homologous to amidohydrolase (KnmE), transporters or regulators (KnmG1-KnmG6), thioesterase (TE, KnmA4), acyl-CoA dehydrogenase (KnmD), NRPSs (KnmA1 and KnmA3), PKS-NRPS (KnmA2), flavin adenine dinucleotide (FAD)-dependent monooxygenases (KnmB1 and KnmB2), asparagine synthase (KnmC), and methyltransferase (KnmF) (Fig. 1c and Supplementary Table 7). Intriguingly, gene cluster highly similar to the *knm* BGC was recognized in the genome of *Actinoalloteichus cyanogriseus* (*cae*, GenBank NO. JQ687072) (Fig. 1c), and moreover, *cae* BGC has been well established for biosynthesizing 2,2′-bipyridine antibiotics caerulomycin^25,26^.

**Fig. 2:**
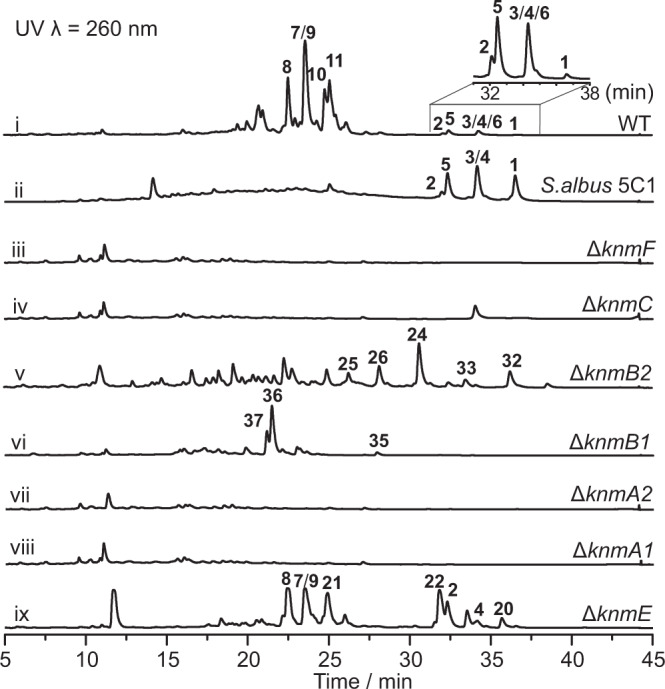
HPLC analysis of metabolic extracts from wild-type *L. rhizosphaerae* NEAU-A2 (i, magnified region focuses on **1**–**6**), heterologous expression strain *S. albus* 5C1 (ii) and mutant strains (iii-ix). Source data are provided as a Source Data file.

To examine the involvement of *knm* BGC in assembling the karnamicins, we first chose to inactivate the putative PKS-NRPS hybrid gene *knmA2* in the cluster, whose homologs *caeA2* and *colA2* in *cae* and *col* clusters have been demonstrated to be responsible for constructing pyridine ring in caerulomycin and collismycin biosynthesis, respectively^25–27^. Based on the gene knockout system we developed in *L*. *rhizosphaerae* NEAU-A2^28,29^, a Δ*knmA2* mutant was obtained by homologous recombination (Supplementary Fig. 41). As expected, Δ*knmA2* completely abolished the production of the karnamicins (Fig. 2, trace vii), providing initial evidence supporting the relevance of the *knm* cluster in biosynthesizing karnamicin. Further single gene deletion experiments were then performed for *knmA1*, *knmB1*, *knmB2*, *knmC*, *knmE*, and *knmF* in the cluster (Supplementary Fig. 41), with the altered (or absent) metabolite profile in the corresponding mutants (Fig. 2) confirming that the *knm* BGC was required for karnamicin assembly. Meanwhile, heterologous expression of *knm* BGC (cosmid p5C1, Fig. 1c) in *Streptomyces albus* J1074 was carried out. Successful detection of karnamicins in the engineered strain *S. albus* 5C1 instead of the *S. albus* J1074 control (strain containing vector alone) further confirmed the dedicated role of *knm* BGC in synthesizing karnamicins (Fig. 2, trace ii and Supplementary Fig. 42, trace iii and iv). Notably, we found that the fatty acid side chain of karnamicins produced by *S. albus* 5C1 was not oxidized, indicating the lack of specific fatty acid oxidase in the heterologous host *S. albus* J1074.

### Functional characterization of pyridine tailoring enzymes in *knm* BGC

KnmA3 is a potential NRPS showing the greatest similarity to EpoB (*Sorangium cellulosum*) that assembles the thiazole ring in epothilone biosynthesis^30–32^ (Supplementary Table 7). Meanwhile, KnmA1, KnmA2, KnmA4, and KnmD strongly resemble CaeA3, CaeA2, CaeA4, and CaeB1 involved in generating pyridine unit in caerulomycin biosynthesis^25,26^ (Supplementary Table 7). Therefore, we instead sought to focus on potential post-tailoring enzymes for the targeted modification of the pyridine ring. Candidate enzymes based on gene annotations included a metal-dependent amidohydrolase (KnmE), two putative FAD-dependent monooxygenases (KnmB1 and KnmB2), asparagine synthase (KnmC), and methyltransferase (KnmF). We individually inactivated these enzyme genes in the heterologous host strain *S. albus* 5C1 using *λ* red-mediated PCR targeting mutagenesis^33^ (Supplementary Fig. 43). However, only the *S. albus* 5C1-Δ*knmB2* mutant was observed to accumulate presumable intermediates (**24**–**26** and **32**–**33**, Fig. 2, trace v), and therefore we chose to conduct further gene inactivation in wild-type strain *L. rhizosphaerae* NEAU-A2.

**Fig. 3:**
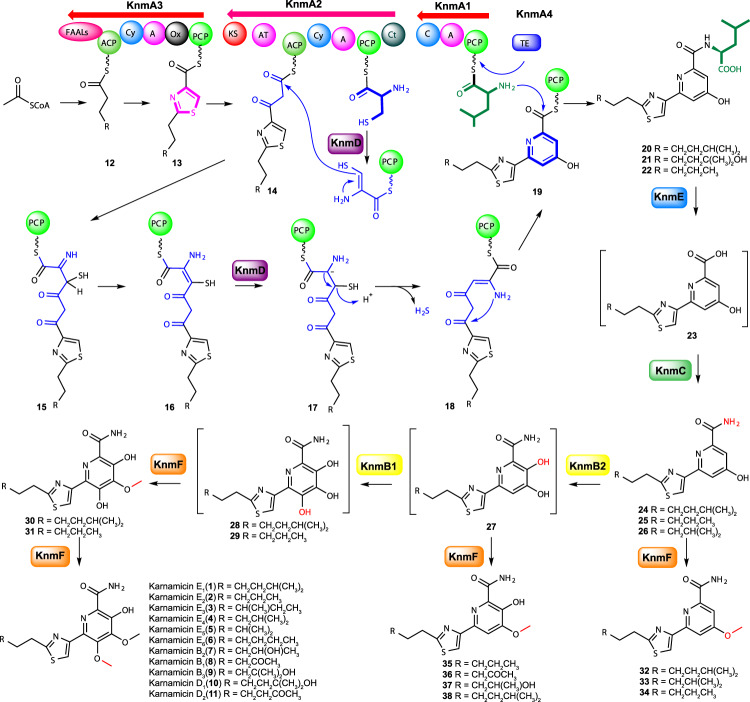
Proposed biosynthetic pathway for karnamicins. FAALs fatty acyl-AMP ligases, ACP acyl carrier protein, Cy condensation/cyclization domain, A adenylation domain, Ox flavin-dependent oxidase domain, PCP peptidyl carrier protein, KS ketosynthase, AT acyl transferase, C condensation domain, Ct terminal condensation domain, TE thioesterase.

KnmE, the putative metal-dependent amidohydrolase, showed the highest identity with CaeD from *A. cyanogriseus* NRRL B-2194^34^ (Supplementary Table 7). Interestingly, the Δ*knmE* strain retained the ability to produce karnamicins, while the accumulation of other metabolites was also observed (**20**–**22**, Fig. 2, trace ix). NMR and HRESIMS (Supplementary Figs. 37, 44–61 and Supplementary Tables 8–10) analysis determined that compounds **20**–**22** are presumed karnamicin biosynthesis intermediates tailored with a l-leucine residue on C3 (Fig. 3). Given the absence of a leucine group on C3 of final karnamicin products, we proposed that KnmE was responsible for removing the l-leucine residue of intermediates **20**–**22** to afford the supposed intermediate **23** (Fig. 3). Moreover, since the formation of the target products karnamicins could be detected along with the accumulation of the intermediates **20**–**22** in the Δ*knmE* strain, it could be concluded that other amidohydrolases could also perform this catalysis, though they may not be as efficient as KnmE. KnmC was a predicted asparagine synthase that could be involved in the transamination of **23** (Fig. 3). We then inactivated *knmC* in the wild-type *L. rhizosphaerae* NEAU-A2 strain (Supplementary Fig. 41). The resultant mutant Δ*knmC* did not produce the karnamicins, nor its putative substrate **23** or other products (Fig. 2, trace iv). Meanwhile, the gene complementation mutant *S. albus* 5C1-Δ*knmC*/*knmC* restored karnamicin production (Supplementary Fig. 42).

Bioinformatics analysis revealed that KnmB1 and KnmB2 resemble various group A FPMOs containing one Rossmann fold domain (GxGxxG signature) involved in FAD binding, one FAD-NAD(P)H binding motif (DG fingerprint), and one GD motif involved in contacting the riboflavin moiety of FAD^35–38^ (Supplementary Table 7 and Supplementary Fig. 62). We thus hypothesized that KnmB1 and KnmB2 may be responsible for the hydroxylation of the pyridine core. Inactivation of *knmB1* or *knmB2* in the wild-type *L. rhizosphaerae* NEAU-A2 strain was then performed by PCR-targeted insertional mutation^29,33^ (Supplementary Fig. 41). As expected, karnamicin production was not observed in either Δ*knmB1* or Δ*knmB2* mutant strains. Instead, we noticed the presence of the major products **36** and **37**, and a small amount of **35** in the Δ*knmB1* mutant strain (Fig. 2, trace vi), as well as major products **24–26** and minor products **32** and **33** in the Δ*knmB2* mutant strain (Fig. 2, trace v). NMR and HRESIMS-based structure elucidation (Supplementary Figs. 37, 63–110 and Supplementary Tables 11–18) indicated that compounds **35–37** found in the Δ*knmB1* strain possess an additional hydroxyl group on the pyridine core compared to **32–33** found in the Δ*knmB2* mutant, while **32** and **33** were potential *O*-methylated derivatives of **24** and **26**, thus supporting the possible assembly logic that KnmB2 and KnmB1 were employed for the C4- and C6-hydroxylation of the pyridine core (Fig. 3), respectively. Notably, substrate promiscuity of the assembly line methyltransferase (likely KnmF) resulted in C5-*O*-methylation of the corresponding pyridine monohydroxylated or dihydroxylated intermediates **24–27**, yielding the methylated compounds **32–33** and **35**–**37** (Fig. 3) observed in the Δ*knmB2* or Δ*knmB1* mutant strains. To test the hypothesized role of KnmF, we chose to delete the *knmF* gene in-frame to avoid potential polar effects on the expression of downstream genes^33^ (Supplementary Fig. 41). However, HPLC analysis of the culture extract of the Δ*knmF* mutant strain did not show the expected C5 or C6 demethylated products, or other karnamicin analogues (Fig. 2, trace iii), which may be due to the instability of the trihydroxypyridine core of intermediate **28** (Fig. 3).

### In vitro characterization of hydroxylation and methylation at the pyridine core

**Fig. 4:**
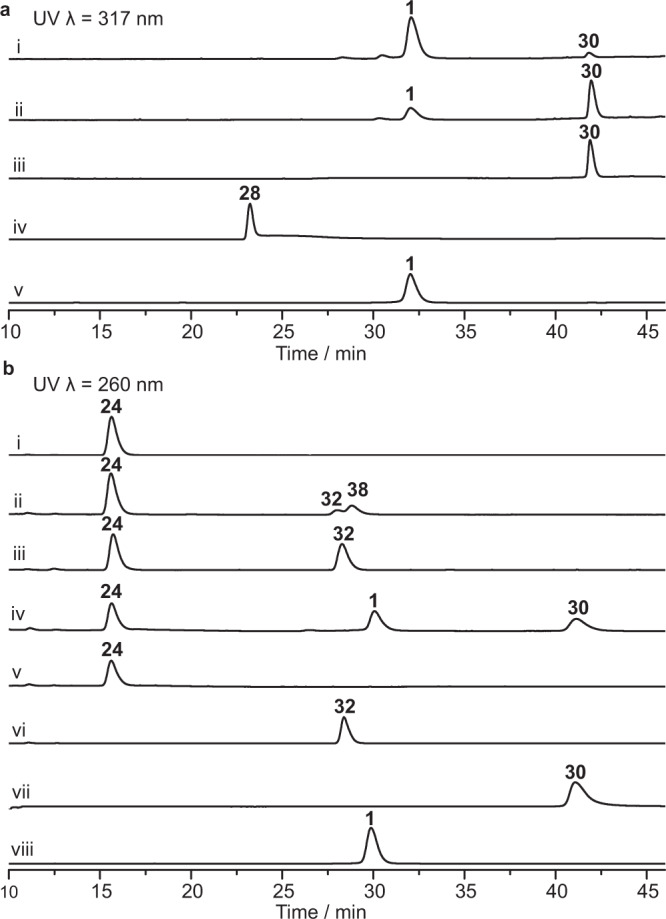
HPLC analysis of the reactions catalyzed by KnmF, KnmB1, and KnmB2. **a** HPLC analysis of KnmF-catalyzed reactions in the presence of **28**, SAM and KnmF. (i) 2 h; (ii) 30 min; (iii) 15 min; (iv) the control reaction in the presence of **28** and SAM; (v) standard **1**. **b** HPLC analysis of KnmB1- and KnmB2-catalyzed reactions. (i) the control reaction in the presence of **24**, SAM and NADPH; (ii) the reaction in the presence of **24**, SAM, NADPH, KnmB2 and KnmF; (iii) the reaction in the presence of **24**, SAM and KnmF; (iv) the reaction in the presence of **24**, SAM, NADPH, KnmF, KnmB1 and KnmB2; (v) the reaction in the presence of **24**, NADPH, KnmB1 and KnmB2; (vi) standard **32**; (vii) standard **30**; (viii) standard **1**. Source data are provided as a Source Data file.

To further examine the catalytic role of methyltransferase KnmF and monooxygenases KnmB1 and KnmB2 during the karnamicin assembly process, we decided to characterize their enzyme activities in vitro. For KnmF, we synthesized **28** (Fig. 3, Supplementary Figs. 111–116, and Supplementary Table 19), a putative dedicated substrate of KnmF with a trihydroxypyridine core. Subsequently, a time course analysis of the enzyme-catalyzed reaction was performed with KnmF expressed by *Escherichia coli* BL21 (DE3) cells (Supplementary Fig. 117) and **28**. Intriguingly, immediately following the consumption of **28**, a compound **30** appeared. The subsequent decrease in abundance of **30** was accompanied by the accumulation of karnamicin E_1_ (**1**) (Fig. 4a). The structure of **30** was established by HRESIMS (C_17_H_24_N_3_O_4_S, *m/z* [M + H]^+^ 366.1477, Supplementary Fig. 118) and NMR analyses (Supplementary Figs. 37, 119–123 and Supplementary Table 20). In addition, we also observed that synthetic **28** was degraded rapidly in the reaction buffer. Consequently, it was suggested that methyltransferase KnmF catalyzed the C5-*O*-methylation of **28** to afford **30**, followed by C6-*O*-methylation to give karnamicin E_1_ (**1**) (Fig. 3).

The purified KnmB1 and KnmB2 proteins expressed in *E. coli* BL21(DE3) cells (Supplementary Fig. 117) were light yellow and released FAD upon boiling (Supplementary Fig. 124). Incubation of KnmB2 with **24**, the major product isolated from the Δ*knmB2* mutant strain, did not generate any detectable product in the presence of the cofactors FAD and NAD(P)H, while the amount of **24** was reduced (Supplementary Fig. 125a, trace vi). Considering the accumulation of intermediates **35**–**37** with a C5-*O*-methylated instead of 4,5-dihydroxylated pyridine ring in the Δ*knmB1* mutant strain, we speculated that the KnmB2-catalyzed hydroxylation product may be unstable, and that additional methyltransferase activity could stabilize the product through the *O*-methylation reaction. Consequently, we then investigated the catalytic reaction by incubating KnmB2 with **24** or **25** in the presence of the identified methyltransferase KnmF (Supplementary Fig. 125). In the event, HPLC analysis showed the conversion of **24** into two products **32** and **38** (Fig. 4b, trace ii). Based on the molecular formulas of **24**, **32**, and **38** (Supplementary Fig. 126), we proposed that **32** was the methylation product of **24**, and **38** was the methylation and oxidation product. Since KnmB2 could not catalyze conversion of **32** to **38** (Supplementary Fig. 125a, trace ii), this indicated that the C5-hydroxyl group of the pyridine core is necessary for KnmB2 activity. Overall, these findings indicated that KnmB2 selectively catalyzes the C4-hydroxylation of the pyridine ring.

Investigation of the function of the likely hydroxylase KnmB1 in vitro was initially carried out by incubating the enzyme with **36**, the major product isolated from the Δ*knmB1* mutant strain (Fig. 2, trace vi). However, neither formation of products nor consumption of substrate was observed in the presence of NADPH (Supplementary Fig. 127a), suggesting that KnmB1 was incapable of hydroxylating **36** at C6. In several previous examples, FPMO-catalyzed hydroxylation of aromatic rings is stimulated by deprotonation of an unmodified hydroxyl group *ortho* or *para* to the target position and the subsequent electrophilic attack by the reactive flavin C4a-hydroperoxide intermediate^38–40^. KnmB1 may behave in a similar way, meaning that C6-hydroxylation would proceed prior to the *O*-methylation of the C5-hydroxyl group. Accordingly, we set about preparing demethylated precursor **27** as a substrate for KnmB1 (Fig. 3). Unfortunately, attempts to obtain **27** by chemically demethylating **36** or **37** failed. Therefore, we chose to incubate **24** with KnmB1 in the presence of KnmB2 and KnmF to test the role of KnmB1. By HPLC analysis, the production of karnamicin E_1_ (**1**) and monomethylated intermediate **30** was observed (Fig. 4b, trace iv). However, due to the instability of the trihydroxypyridine core, the putative enzymatic reaction product **28** was not observed when incubating **24** with KnmB1 and KnmB2 (Fig. 4b, trace v), which was in agreement with our observation that synthetic **28** was degraded rapidly in the reaction buffer. Altogether, these results highlight a biosynthetic procedure in which following the KnmB2-catalyzed C4-hydroxylation of **24**, KnmB1 activity completed the C6-hydroxylation of the pyridine core, and then KnmF was responsible for successive methylations of the C5- and C6-hydroxyl groups of **28** to yield karnamicin E_1_ (**1**) (Fig. 3). Consistent with this procedure, in the presence of KnmF, **28** and **30** were completely converted into **1** within 2 h, while the transformation of **24** into **32** took more than 24 h. This also explains why the main product of the Δ*knmB2* mutant was intermediate **24** rather than methylated product **32**.

### Catalytic mechanism of regioselective hydroxylases KnmB1 and KnmB2

In order to further understand the functional mechanism of regioselective hydroxylases KnmB1 and KnmB2, multiple sequence alignment and phylogenetic analyses of KnmB1, KnmB2, and other reported FPMOs responsible for hydroxylation of pyridine nucleus or aromatic rings were performed (Supplementary Figs. 62 and 128). It was established that KnmB1 and KnmB2 belong to group A FPMOs^41^ with the characteristic conserved FAD binding sites^35^ (GXGXXG…DG…GD, Supplementary Fig. 62). KnmB1 showed the highest sequence identity with CaeB6 (55%), a selective hydroxylase in the biosynthesis of the 2,2ʹ-bipyridine compounds caerulomycins^40^, and 2-methyl-3-hydroxypyridine-5-carboxylic acid oxygenase (MHPCO, 45%), an enzyme catalyzing the oxidative pyridine ring-opening reaction of vitamin B6 degradative pathway in *Mesorhizobium loti* MAFF303099^42^. Although only 30.6% of the amino acid sequence identity was elucidated, the enzyme most similar to KnmB2 was found to be HpxO, a flavin-dependent urate oxidase that catalyzes the hydroxylation of uric acid to yield a 5-hydroxyisourate intermediate of a purine catabolic pathway in *Klebsiella pneumoniae*^43^. Among these enzymes, crystal structures and catalytic mechanisms of MHPCO and HpxO have been investigated^42,43^. However, the conserved catalytic residues elucidated in MHPCO/HpxO could not be identified in KnmB1/KnmB2 by amino acid sequence alignments (Supplementary Fig. 62). We hypothesized that distinct active-site architecture may be employed by KnmB1/B2 for hydroxylation of the pyridine nucleus.

**Fig. 5:**
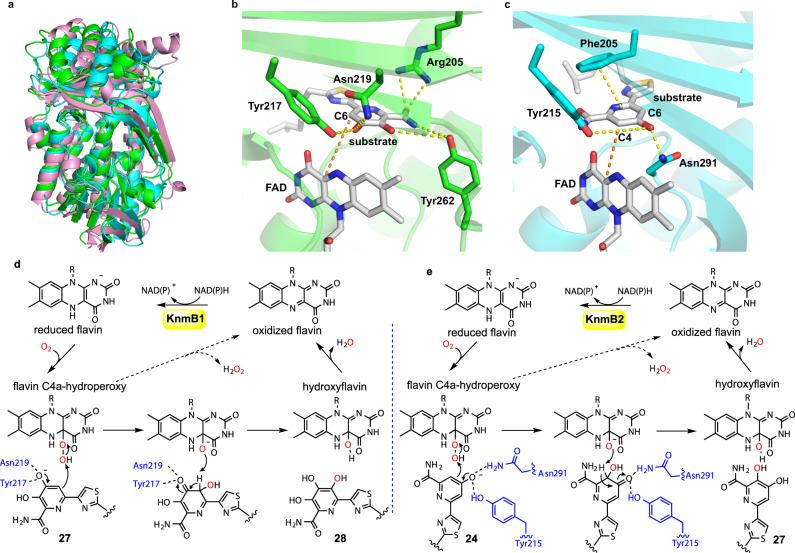
Structures and catalytic mechanisms of KnmB1 and KnmB2. **a** Comparison of the overall structures of KnmB1 (green), KnmB2 (cyan), and *p*-hydroxybenzoate hydroxylase (PHBH; pdb:1pbe; pink). **b** Molecular docking of **27** in KnmB1. **c** Molecular docking of **24** in KnmB2. N, O, and S atoms are colored blue, red and yellow, respectively. C atoms in KnmB1 residues, KnmB2 residues, and small molecules are colored as green, cyan, and white, respectively. **d** Proposed catalytic mechanism of KnmB1. **e** Proposed catalytic mechanism of KnmB2. R is ribityl adenosine diphosphate for FAD.

Despite many attempts, we were unable to obtain crystals of the KnmB1 or KnmB2 protein suitable for X-ray crystallographic analysis. Fortunately, the recent success of AlphaFold has demonstrated the values of artificial intelligence (AI) methods in predicting highly accurate protein structures, which we chose to deploy in this setting^44^. Interestingly, the overall structure of KnmB1 or KnmB2 predicted by AlphaFold was highly similar to that of PHBH (Fig. 5a) and other flavoprotein aromatic hydroxylases^41^, thus raising the question of whether KnmB1 and KnmB2 acomplish the pyridine ring hydroxyation in a way similar to that of the reported aromatic ring hydroxylases. To unravel the structure-function relationship of KnmB1 and KnmB2, we performed a molecular docking analysis using the predicted protein structures and their corresponding substrates (**27** for KnmB1, and **24** for KnmB2). As shown in Fig. 5, potential hydrogen bond interactions between substrate **27**, especially its amide and hydroxyl groups, and residues Asn219, Tyr262, Arg205, and Tyr217, in KnmB1 were present (Fig. 5b). For KnmB2, the residues Asn291 and Tyr215 were predicted to form hydrogen bonds with substrate **24**, while Phe205 was suggested to exhibit π-π stacking interaction with the pyridine ring (Fig. 5c). Particularly, the side chains of Asn219/291 and Tyr217/215 in KnmB1 and KnmB2 were predicted to be hydrogen bond donors, which may act by deprotonating substrate for oxygen activation. While the active sites of KnmB1 and KnmB2 do bear some homology to other flavoprotein aromatic hydroxylases and group A FPMOs (MHPCO^42^ and HpxO^43^ also utilize tyrosine in forming hydrogen bond with their respective substrates), other residues, such as Asn219 and Tyr262 in KnmB1 and Asn291 and Phe205 in KnmB2, are notably unique.

To test the role of these predictive active-site residues in determining the catalytic activity of KnmB1/B2, a series of site-directed mutants of KnmB1 (N219D, Y217A/F, Y262A and R205A) and KnmB2 (N291D, Y215A/F and F205A) were respectively generated. In comparison with wild-type, ~26–70% decrease in FAD content was observed in all enzyme variants except the Y217A mutant of KnmB1 (Supplementary Fig. 129), indicating that the FAD binding is perturbed in these variants. Interestingly, in vitro enzymatic assays revealed that these enzyme mutants lost all hydroxylation activity towards **27** or **24** (Supplementary Fig. 130). However, the possibility that site-directed mutations may have led to gross changes in tertiary protein structure, which would also lead to a substantial loss of FAD binding and catalytic activity, could be ruled out, since these variants retained the NADPH oxidase activity observed in wild-type enzymes (Supplementary Fig. 131a–c). Inspired by this result, we chose to explore the catalytic features of KnmB1 and KnmB2 by monitoring the kinetics of NADPH consumption and hydrogen peroxide (H_2_O_2_) formation in time. First, it is interesting to note that, KnmB1 and particularly KnmB2 have noticeable NADPH oxidase activity regardless of the presence of a hydroxylatable (**24** for KnmB2, coupling efficiency 0–45%) or non-hydroxylatable (**24** for KnmB1, and **32** for KnmB2) effector (Supplementary Fig. 131d–j), suggesting that these two group A FPMOs are inefficient at inhibiting wasteful flavin reduction in the absence of any effector. Second, as a non-deprotonatable ligand, methylated product **32** could dramatically stimulate the reaction of KnmB2 with NADPH, even to a greater extent than that the substrate **24** did (Supplementary Fig. 131i, j). This means that features other than an activating substituent on the pyridine ring were sensed and triggered rapid acceleration of flavin reduction in KnmB2. For KnmB1, its direct substrate **27** is not available. However, **24** but not **32** or **36** could act as a non-substrate effector to accelerate the reduction of flavin in KnmB1 by NADPH (Supplementary Fig. 131f), indicating that the deprotonatable C5-hydroxyl group of the ligand is specifically required for exerting effector function upon binding to KnmB1.

Based on the established understanding of group A FPMO-catalyzed hydroxylation of aromatic compounds^45,46^, it is reasonable to infer that the side chains of residues such as Asn219/291 and Tyr217/215 likely participate in forming the critical hydrogen bond networks responsible for proton transfer in the catalytic cycle of KnmB1/2. The proton transfer system is suggested to be required for substrate deprotonation, which is an essential step to drive the conformational changes (“out” and “in”) of cofactor flavin for reduction by NADPH, and to activate the substrate for electrophilic attack by flavin C4a-hydroperoxide intermediate^47^. Therefore, mutation of KnmB1 at Asn219 (N219D) or Tyr217 (Y217A/F) resulted in complete loss of response to non-substrate effector **24** (Supplementary Fig. 131e–g). Meanwhile, in agreement with the finding that accelerated flavin reduction in KnmB2 was independent of the presence of an activating substituent on the pyridine ring, the substrate effector role of **24** in promoting flavin reduction was retained (though decreased) in N291D or Y215F mutant of KnmB2 (Supplementary Fig. 131h–j). Nevertheless, the two mutants lost all hydroxylation activity towards **24**, which is probably due to impaired electrophilic substitution in the oxidative half-reaction resulting from deficiency in substrate deprotonation.

Notably, binding and the proper orientation of substrate in the active site, which may be attributed to the side chains of Tyr262/Arg205 (KnmB1) and Phe205 (KnmB2), is also decisive for the reaction. Mutation in any of these residues resulted in a reduced (Y262A) or absent (R205A and F205A) response of KnmB1/2 to their effectors (Supplementary Fig. 131g, i). In addition, different from Y215F mutant, large-to-small amino acid substitution at Tyr215 in KnmB2 (Y215A) led to loss of response to substrate **24** (Supplementary Fig. 131i), which may be ascribed to improper binding or positioning of the substrate owing to steric change of the active-site cavity. Based on these results, a possible catalytic mechanism for the KnmB1- and KnmB2-catalyzed hydroxylation of the pyridine ring in karnamicin biosynthesis was proposed (Fig. 5d, e), which is analogous to that of group A FPMOs employing a flavin C4a-hydroperoxide intermediate^48^. More importantly, though, these structural studies suggest that KnmB1 and KnmB2 facilitate the chemo- and regiospecific hydroxylation of the pyridine nucleus through the exquisite arrangement of the enzyme-specific active-site residues, which are notably distinct from other FPMOs. This underlines the great diversity and unique regioselectivity of group A FPMOs^38,41^.

### Karnamicins as efficient ACE inhibitors

Derivates of pyridines, thiazoles, and pyridine-thiazole hybrids have been well recognized as bioactive candidates with great potential for medical applications^14–19^. As a class of compounds with a unique skeleton composed of fully substituted hydroxypyridine and thiazole moieties, we first conducted a preliminary investigation of antibacterial and antifungal activities of karnamicins **1**–**11** against plant pathogens. However, only weak activity was found. Interestingly, in an attempt to explore natural ACE inhibitors for hypertension treatment, we surprisingly found that karnamicins isolated from wild-type and mutant strains had significant ACE inhibitory activity with IC_50_ (the concentration yielding 50% inhibition) values ranging from 0.24 to 5.81 μM (except compound **32**, Table 1). Furthermore, analysis of the structure-activity relationship seems to imply that alkyl side chain hydroxylation leads to a two- to fivefold increase in activity. These activities bode well for future medicinal chemistry optimization, and point to the potential use of karnamicin derivatives as a treatment for hypertension.

**Table 1:** ACE inhibitory activity^a^ of karnamicins

Compounds	IC_50_ (μM)	Compounds	IC_50_ (μM)
**2**	1.22 ± 0.02	**21**	5.36 ± 2.97
**5**	1.10 ± 0.09	**25**	5.81 ± 0.47
**7**	0.30 ± 0.05	**29**	2.94 ± 0.38
**9**	0.24 ± 0.01	**31**	5.42 ± 0.10
**10**	0.71 ± 0.05	**32**	0^c^
**11**	0.50 ± 0.03	**36**	0.62 ± 0.07
**20**	2.58 ± 0.30	Captopril^b^	0.014.45 ± 0.51

^a^Results are expressed as mean IC_50_ ± standard deviation from *n* = 3 independent replicates.

^b^Positive control.

^c^No activity at 50 μM.

## Discussion

In this study, we presented the isolation and structural elucidation of karnamicins **1**–**11** with a unique skeleton composed of fully substituted hydroxypyridine and thiazole moieties from *L. rhizosphaerae* NEAU-A2 (Fig. 1b). On the basis of ^13^C-labeled precursor feeding, genome sequencing, bioinformatic analysis, in vivo gene knockout, and heterogeneous expression, we determined that the karnamicin biosynthetic assembly line was encoded by an unreported BGC named as *knm* (Fig. 1c). Among the deduced protein products of *knm* BGC, KnmA1, KnmA2, KnmA3, KnmA4, and KnmD, showed clear similarities to enzymes that have been identified to be involved in generation of thiazole ring and pyridine ring moieties^25–27,30–32^. Subsequent characterization of enzymes responsible for the post-modification of the pyridine moiety led to the final elucidation of the complete biosynthetic pathway for karnamicins (Fig. 3).

Quite recently, enzymes showing high similarity to PKS/NRPS hybrid protein KnmA2 and the acyl-CoA dehydrogenase KnmD (Supplementary Table 7) have been found to act synergistically to generate 2,2’-bipyridine in the caerulomycin (CaeA2 and CaeB1) and collismycin (ColA2 and ColB1) biosynthetic assembly lines^26^. Therefore, we speculated here that KnmA2 and KnmD afforded the pyridine ring moiety in the karnamicins by the same mechanism. The domains of NRPS KnmA1 were organized as C-A-PCP for l-leucine extension of **19**, and with the help of thioesterase KnmA4, the corresponding products **20**–**22** were released. Subsequently, KnmE removed the l-leucine residue to afford intermediate **23**. KnmC activity was required for the transamination reaction to yield **24–26**. Then, KnmB2-catalyzed hydroxylation of C4 affording dihydroxylated pyridine intermediate **27**, which was further oxidized by KnmB1 to form the trihydroxypyridine core of **28**. Finally, the karnamicin assembly was completed by successive KnmF-catalyzed *O*-methylation of **28** (Fig. 3). Intriguingly, genome mining using *knm* BGC as bait further resulted in the identification of another putative karnamicin-producing cluster from *L. aerocolonigenes* NBRC 13195 (*knmH*, GenBank No. GCA_000974445.1, Supplementary Fig. 132), providing additional resources for karnamicin production.

FPMOs are a class of enzymes that are capable of catalyzing a diverse set of chemo-, regio-, and enantio-selective oxyfunctionalization reactions^41^. In the *knm* BGC, KnmB2 and KnmB1 were identified as two FPMOs responsible for regioselective hydroxylation of the pyridine core, contributing substantially to the formation of the unique skeleton composed of fully substituted hydroxypyridine and thiazole moieties in the karnamicins. In agreement with this specific catalytic activity, amino acid sequence alignment, AlphaFold-based protein structure prediction, and site-directed mutations demonstrated that KnmB2/B1 recruited active sites distinct from other known aromatic ring hydroxylases to accomplish the hydroxylation of the pyridine nucleus, broadening our understanding of the diverse origin and functional evolution of pyridine hydroxylases in the FPMOs family. Consequently, the characterization of KnmB2/B1 provided important insights into the development of pyridine hydroxylase variants with designer properties for the production of pharmaceutical ingredients and fine chemicals. This is all the more important given the significant ACE inhibitory activity of the karnamicins, which could be used for the development of natural product-derived antihypertensive agents.

## Methods

### General experimental procedures

General materials and reagents used in this study are summarized in Supplementary Information. The bacterial strains, plasmids, and primers used in this study are summarized in Supplementary Tables 21–23. Chromaster was used for HPLC data collection. MEGA version 7 was used for amino acid alignment and phylogenetic tree construction. OriginPro 9.0 was used for data visualization. MestReNova 6.1 was used for NMR data analysis. ChemBioDraw Ultra 20.0 was used for drawing chemical structures (Figs. 1–3). GeneMarkS Version 2.0^22^ was used for the prokaryotic genome annotation. antiSMASH 4.0^23^ and PRISM 3^24^ were used for genome mining. The sequence alignment of gene clusters was created using Clustal Omega and the figure was produced using EsPript 3.0.

### Isolation and purification of 1–11 from *L. rhizosphaerae* NEAU-A2

*L. rhizosphaerae* NEAU-A2 was grown on ISP3 agar plates^28^ (Oatmeal 2%, KNO_3_ 0.02%, MgSO_4_·7H_2_O 0.02%, K_2_HPO_4_·3H_2_O 0.05% and Agar 2%, w/v, pH 7.2) for 5 days at 30 °C. Then, the mycelium was inoculated into 250 mL baffled Erlenmeyer flasks containing 50 mL of seed medium (TSB, Tryptone Soy Broth, 30 g/L) and cultivated for 2 days at 30 °C on a rotary shaker (200 rpm). After that, 5% (v/v) of the seed culture was transferred into 1000 mL baffled Erlenmeyer flasks filled with 300 mL of production medium B consisting of 0.5% soluble starch (w/v), 0.2% tryptone (w/v), 1% glucose (w/v), 0.2% yeast extract (w/v), 0.4% NaCl (w/v), 0.05% K_2_HPO_4_·3H_2_O (w/v), 0.05% MgSO_4_·7H_2_O (w/v), and 0.2% CaCO_3_ (w/v), and cultured on a rotary shaker (200 rpm) at 30 °C for a week.

The fermentation broth (40 L) was centrifuged (3488×*g*, 20 min), and the supernatant was extracted with ethyl acetate thrice. The ethyl acetate extract was concentrated to afford 10.0 g of oily crude extract. The mycelia were extracted with methanol (1 L × 3) and then concentrated in vacuo to remove the methanol to yield the aqueous concentrate. This aqueous concentrate was finally extracted with ethyl acetate (1 L × 3) to give 1.0 g of oily crude extract. Finally, two parts of the extracts were combined for further purification.

The crude extract in total (11.0 g) was applied to silica gel column chromatography using a successive elution of petroleum ether/ethyl acetate (1:0, 20:1, 10:1, 5:1, 3:1, 1:1, and 0:1, v/v) to yield fractions A–F. Fr. A (petroleum ether/ethyl acetate, 1:1, v/v) was subjected to semipreparative HPLC-DAD (0–40.0 min, 50% CH_3_CN in H_2_O; 40.1–60.0 min, 55% CH_3_CN in H_2_O; 60.1–70.0 min, 70% CH_3_CN in H_2_O) directly to afford **5** (*t*_R_ = 37.0 min, 5.0 mg), **2** (*t*_R_ = 39.2 min, 6 mg), and **6** (*t*_R_ = 55.0 min, 4.4 mg). Fr. A-52 min was further purified by semipreparative HPLC-DAD (CH_3_CN:H_2_O = 44:56, v/v) to give **3** (3.0 mg) and **4** (5.0 mg). Fr. A-67 min was also further separated using the same semipreparative HPLC-DAD (CH_3_OH:H_2_O = 80:20, v/v) to provide **1** (*t*_R_ = 32.6 min, 4.8 mg). Fr. D (ethyl acetate) was subjected to semipreparative HPLC (0–28.0 min, 35% CH_3_CN in H_2_O; 28.1–43.0 min, 40% CH_3_CN in H_2_O) to yield **7** (*t*_R_ = 26.0 min, 20 mg). Fr. D-31.7 min was further purified by semipreparative HPLC to give **9** (13 mg) and **8** (11.2 mg). Fr. D-41.3 min was further purified by means of semipreparative HPLC to yield **11** (7 mg). Fr. E was submitted to semipreparative HPLC (0–27.0 min, 35% CH_3_CN in H_2_O; 27.1–43.0 min, 40% CH_3_CN in H_2_O; 43.1–48.0 min, 100% CH_3_CN) to obtain **10** (*t*_R_ = 26.0 min, 11.0 mg).

### X-ray crystallographic analysis of compound 7

Crystal data for **7**: C_16_H_21_N_3_O_5_S, *M* = 367.42, *a* = 8.489(2) Å, *b* = 20.732(5) Å, *c* = 9.931(2) Å, *α* = 90°, *β* = 106.838(4)°, *γ* = 90°, *V* = 1672.9(7) Å^3^, *T* = 100(2) K, space group *P*21, *Z* = 4, *μ*(MoKα) = 0.227 mm^-1^, 17516 reflections measured, 8938 independent reflections (*R*_*int*_ = 0.0755). The final *R*_*1*_ values were 0.0629 (*I* > 2*σ*(*I*)). The final *wR*(*F*^2^) values were 0.1387 (*I* > 2*σ*(*I*)). The final *R*_*1*_ values were 0.1055 (all data). The final *wR*(*F*^2^) values were 0.1583 (all data). The goodness of fit on *F*^2^ was 1.011. Flack parameter = 0.21(10).

### Isolation and purification of metabolites from the mutant strains

Based on HPLC analysis results of the mutant cultures, we selected Δ*knmE*, Δ*KnmB1*, and Δ*KnmB2* mutants for large-scale fermentation (20 L) using the aforementioned method and medium. The metabolites of the mutant strains were isolated and purified by silica gel column chromatography combined with thin-layer chromatography and semipreparative RP-HPLC. Finally, **20**, **21**, and **22** were isolated from the mutant strain NEAU-A2-Δ*knmE*. **24**, **25**, **26**, **32**, and **33** were obtained from the mutant strain *S. albus* 5C1-Δ*knmB2*. Compounds **35**, **36**, and **37** were separated from the mutant strain NEAU-A2-Δ*knmB1*.

### Isotopic labeling experiments

Three ^13^C-labeled compounds, [1-^13^C] sodium acetate, [2-^13^C] sodium acetate, and [1, 2-^13^C_2_] sodium acetate, were used for the feeding experiments. Briefly, three ^13^C-labeled compounds (500 mg) were individually dissolved in ddH_2_O at a concentration of 250 mg/mL as stock solution. *L. rhizosphaerae* NEAU-A2 was grown in seed medium (TSB) for 30 h, and the resultant seed culture (5% inocula) was then inoculated into 500 mL production medium at 30 °C on a rotary shaker (220 rpm). After 12, 24, and 36 h of incubation, a ^13^C-labeled precursor ([1-^13^C], [2-^13^C], or [1, 2-^13^C_2_] sodium acetate) was added into culture with the volume of 0.5 mL, 1 mL, and 0.5 mL, respectively, fermented for additional 3 days. Then, the culture was harvested and extracted with ethyl acetate for three times. The extracts were subjected to semipreparative HPLC-DAD directly to afford isotopic labeling compound **7** using an isocratic elution of 35% (v/v) acetonitrile containing 0.1% (v/v) acetic acid with a flow rate of 3 mL/min.

### Genomic library construction

DNA isolation and manipulation in *L. rhizosphaerae* NEAU-A2 were performed according to standard manufacturer’s protocol^33^. Genomic library of *L. rhizosphaerae* NEAU-A2 was constructed in SuperCos I and pJTU2554 by using the Gigapack III Gold Packaging Extract system according to the manufacturer’s instructions (Stratagene). Briefly, the genomic DNA was partially digested with *Mbo*I. Then, the 30–42 kb DNA fragments were isolated and ligated to cosmid pJTU2554 and pSuperCos I. MaxPlax Lambda packaging extracts were used to packaging. About 4000 *E. coli* clones were picked from cosmid pJTU2554 genomic library, and about 2000 *E. coli* clones were picked from cosmid SuperCos I genomic library. They were then stored in 96-well microplates at −80 °C.

### Heterologous expression and gene inactivation experiments

The positive cosmid, p5C1 from cosmid pJTU2554 genomic library, was introduced to the selected model *Streptomyces* host (*S. albus* J1074) by *E. coli*–*Streptomyces* conjugation to obtain strain *S. albus* 5C1 according to the standard protocol^33^. Cosmid p5C1 which contains the whole *knm* gene cluster was used for the construction of gene deletion mutant. The *λ*-Red-mediated gene replacements were carried out following standard procedures^33,49^ using the primers listed in Supplementary Table 22. Finally, five markerless gene inactivation mutants (Δ*knmE*, Δ*knmB1*, Δ*knmB2*, Δ*knmC* and Δ*knmF*) were constructed in this study. The PCR verification of the mutants are shown in Supplementary Fig. 43 with corresponding primers listed in Supplementary Table 22.

The gene inactivation experiments were carried out in wild-type strain *L. rhizosphaerae* NEAU-A2. Briefly, apramycin resistance gene cassettes (*aac(3)IV-oriT* surrounded by FRT sites*)* with 39 bp homologous to each side of the gene to be inactivated were amplified from plasmid pIJ773 by PCR using the primers listed in Supplementary Table 22. Each of the apramycin resistance gene cassette was then introduced by electroporation into *E. coli* BW25113/pIJ790/S4H1, BW25113/pIJ790/S20G3, or BW25113/pIJ790/S12G3. The correctly mutated cosmids were introduced into *E. coli* ET12567/pUZ8002 by electroporation and then transferred into *L. rhizosphaerae* NEAU-A2 through intergeneric conjugation. Exconjugants resistant to apramycin were randomly isolated and tested for the loss of kanamycin resistance. Further, double crossover mutant strains were confirmed by PCR using primers listed in Supplementary Table 22. Finally, seven mutants (Δ*knmE*, Δ*knmA1*, Δ*knmA2*, Δ*knmB1*, Δ*knmB2*, Δ*knmC* and Δ*knmF*) were successfully constructed in *L. rhizosphaerae* NEAU-A2. The PCR verification of the mutants are shown in Supplementary Fig. 41. All mutants were cultivated in medium B for 5 days and the metabolic extracts were monitored by HPLC-DAD. The HPLC analysis was performed at a flow rate of 1 mL/min with DAD detection using a 45 min solvent gradient as follows: *T* = 0 min, 8% B; *T* = 32 min, 80% B; *T* = 36.1 min, 90% B; *T* = 40 min, 100% B; *T* = 40.1 min, 8% B; *T* = 45 min, 8% B (A, H_2_O; B, MeCN), and the column temperature was 25 °C (Analytical method A).

### Chemical synthesis of compound 28

To a solution of **1** (9 mg) in dichloromethane (2 mL) was added BBr_3_ (500 µL) at −78 °C under N_2_ atmosphere^50^. The reaction was stirred at −78 °C for 30 min and was then warmed up to rt. The reaction was then stirred at rt for 10 h. The reaction mixture was slowly added into 4 mL saturated NaHCO_3_ solution at 0 °C. The aqueous layer was extracted by ethyl acetate for three times and the combined organic layers were purified using RP-HPLC to afford **28** (4.4 mg).

### In vitro assay of KnmB1, KnmB2, and KnmF

The reaction of KnmF was carried out at 30 °C in a 100 µL system containing 50 mM Tris-HCl buffer (pH 7.5), 1 mM compound **28**, 2 mM SAM, and 2 μM KnmF. After 15 min, 30 min, and 2 h, an equal volume of MeOH was added to the assay to quench the reaction. After centrifugation (10 min at 13,523×*g*), the supernatant was analyzed by HPLC-DAD with solvent gradient as follows: *T* = 0 min, 55% B; *T* = 13.5 min, 55% B; *T* = 19 min, 65% B; *T* = 35 min, 65% B; *T* = 40 min, 100% B; *T* = 45 min, 100% B; *T* = 45.1 min, 55% B; *T* = 51 min, 55% B (A, H_2_O + 0.1% CH_3_COOH, v/v; B, MeCN), and the column temperature was 25 °C (Analytical method B).

The reaction of KnmB1 was carried out at 30 °C in a 100 µL system containing 50 mM Tris-HCl buffer (pH 7.5), 0.2 mM compound **36**, 1 mM NADPH, and 5 μM KnmB1. The reaction of KnmB2 and KnmF was carried out at 30 °C in a 100 µL system containing 50 mM Tris-HCl buffer (pH 7.5), 0.2 mM compound **24** or **25**, 1 mM NADPH (NADH), 1 mM SAM, 2 μM KnmF, and 5 μM KnmB2. The reaction of KnmB1, KnmB2, and KnmF was performed at 30 °C in a 100 µL system containing 50 mM Tris-HCl buffer (pH 7.5), 0.2 mM compound **24**, 2 mM NADPH (NADH), 1 mM SAM, 5 μM KnmB1, 5 μM KnmB2 and 2 μM KnmF. After 4 h, an equal volume of MeOH was added to the assay to quench the reaction. After centrifugation (10 min at 13,523×*g*), the supernatant was analyzed by HPLC-DAD with a YMC-Triart C_18_ column (250 mm × 4.6 mm i.d., 5 μm) at a flow rate of 1.0 mL/min, solvent gradient as follows: *T* = 0 min, 55% B; *T* = 13.5 min, 55% B; *T* = 19 min, 65% B; *T* = 48 min, 65% B; *T* = 48.1 min, 100% B; *T* = 53 min, 100% B; *T* = 53.1 min, 55% B; *T* = 58 min, 55% B (A, H_2_O + 0.1% CH_3_COOH, v/v; B, MeCN), and the column temperature was 25 °C (Analytical method C).

### Determination of the flavin cofactor of KnmB1 and KnmB2

The protein solutions of KnmB1 and KnmB2 were incubated at 100 °C for 10 min for denaturation. After centrifugation, the supernatants were subjected to HPLC-DAD analysis on a C_18_ column eluted with a flow rate of 1 mL/min over a 28 min gradient as follows: *T* = 0 min, 10% B; *T* = 20 min, 100% B; *T* = 24 min, 100% B; *T* = 24.1 min, 10% B; *T* = 28 min, 10% B (A, H_2_O; B, CH_3_OH) (Analytical method D). Standard FAD was used as control.

### Molecular docking

Molecular docking was performed using Autodock Vina 1.2.2^51^. The ligand substrates **24**, **27**, and FAD were generated using ChemBio3D Ultra 12.0, and then energy minimized with Molecular Mechanics (MM2) until a minimum root-mean-square (RMS) gradient of 0.100 was performed (https://www.chemdraw.com.cn/). Then, substrates **24** and **27** were docked into the binding pocket of protein KnmB1 and KnmB2 based on AlphaFold protein structures prediction. During docking, the critical parameters such as grid number and algorithm were set to default values, but rotatable bonds in the ligand were not set in this manner in order to allow for flexible docking. Finally, 100 independent docking runs were performed, and the complex structure with the best combination of low binding energy and favorable orientation was selected. The resulting poses were analyzed and checked for hydrogen bonding in the PyMOL 2.5.2 (http://www.pymol.org) Molecular Graphics System.

### Angiotensin-converting enzyme inhibitor of assay

ACE inhibitory activities were evaluated according to literatures^52–54^. Test compounds were dissolved in DMSO (10 mM), then diluted to afford serial concentrations with 80 mM HEPES-300 mM NaCl buffer (HEPES-NaCl, pH 8.3). Each compound solution was incubated with ACE solution (0.02 U/mL in HEPES-NaCl buffer) at 37 °C for 10 min in the 96-well microtiter plate. Then, FAPGG (dissolved in HEPES-NaCl buffer) was added and fully mixed. The initial absorbance value of each well was immediately measured at 340 nm and recorded as *A*_0_. After incubating at 37 °C for 30 min, the absorbance value at the end of the reaction of each well was measured at 340 nm again and denoted as *A*_30_. The decrease in the 340 nm absorbance was expressed as $\varDelta A={A}_{0}-{A}_{30}$. The ACE inhibition (percent) was calculated as follows:

1 $$R(\%)=\left(\varDelta {A}_{{NC}}-\varDelta {A}_{S}\right)/\varDelta {A}_{{NC}}\times 100$$

where Δ*A*_NC_ is the absorbance value of negative control and Δ*A*_S_ is the absorbance value of the sample to be tested. The percentages of ACE inhibition were plotted vs Log_10_ inhibitor concentrations obtaining a sigmoid curve. The IC_50_ values (50% ACE activity inhibition), were estimated with GraphPad Prism 7.0 sigmoidal dose-response plots of inhibitor concentration (μM) versus percent inhibition. Captopril was used as a positive control. The results represent the mean IC_50_ value ± standard deviation of three independent assays.

### Reporting summary

Further information on research design is available in the Nature Portfolio Reporting Summary linked to this article.

## Supplementary information

Supplementary Information

Reporting Summary

## Data Availability

The sequence data for ORFs in *knm* cluster of *L. rhizosphaerae* NEAU-A2 have been deposited in GenBank with accession codes OM436385, OM436386, OM436387, OM436388, OM436389, OM436390, OM436391, OM436392, OM436393, OM436394, OM436395, OM436396, OM436397, OM436398, OM436399, OM436400, OM436401, OM436402, OM436403, OM436404, OM436405, OM436406, OM436407, and OM436408. Crystallographic data for the structure of **7** reported in this Article have been deposited at the Cambridge Crystallographic Data Centre, under deposition number CCDC 2143094. Copies of the data can be obtained free of charge via https://www.ccdc.cam.ac.uk/structures/. All data that support the findings of this study are available in the main text and the supplementary information. Plasmids generated in this study are available from the corresponding author. Source data are provided with this paper.

## Source data

Source Data

## Materials availability

All unique materials are readily available from the corresponding author on request.

## References

[1] World Health Organization. *WHO List of Priority Medical Devices for Management of Cardiovascular Diseases and Diabetes* (World Health Organization, 2021).

[2] Kjeldsen SE (2018). Hypertension and cardiovascular risk: general aspects. Pharmacol. Res..

[3] Hessami A (2021). Cardiovascular diseases burden in COVID-19: systematic review and meta-analysis. Am. J. Emerg. Med..

[4] Pranata R, Huang I, Lim MA, Wahjoepramono EJ, July J (2020). Impact of cerebrovascular and cardiovascular diseases on mortality and severity of COVID-19-systematic review, meta-analysis, and meta-regression. J. Stroke Cerebrovasc. Dis..

[5] Tadic M, Cuspidi C, Mancia G, Dell′Oro R, Grassi G (2020). COVID-19, hypertension and cardiovascular diseases: should we change the therapy?. Pharmacol. Res..

[6] Coates D (2003). The angiotensin converting enzyme (ACE). Int. J. Biochem. Cell Biol..

[7] Fleming I (2006). Signaling by the angiotensin-converting enzyme. Circ. Res..

[8] Ancion A, Tridetti J, Nguyen Trung ML, Oury C, Lancellotti P (2019). A review of the role of bradykinin and nitric oxide in the cardioprotective action of angiotensin-converting enzyme inhibitors: focus on perindopril. Cardiol. Ther..

[9] Izzo JL, Weir MR (2011). Angiotensin-converting enzyme inhibitors. J. Clin. Hypertens..

[10] Marte, F., Sankar, P. & Cassagnol, M. *Captopril In StatPearls* (StatPearls Publishing, 2022). 30571007

[11] Messerli FH, Bangalore S, Bavishi C, Rimoldi SF (2018). Angiotensin-converting enzyme inhibitors in hypertension: to use or not to use?. J. Am. Coll. Cardiol..

[12] Lancaster SG, Todd PA (1988). Lisinopril, a preliminary review of its pharmacodynamic and pharmacokinetic properties, and therapeutic use in hypertension and congestive heart failure. Drugs.

[13] Abdel-Razek AS, El-Naggar ME, Allam A, Morsy OM, Othman SI (2020). Microbial natural products in drug discovery. Processes.

[14] Eryılmaz S (2020). Derivatives of pyridine and thiazole hybrid: synthesis, DFT, biological evaluation via antimicrobial and DNA cleavage activity. Bioorg. Chem..

[15] Gümüş M, Yakan M, Koca İ (2019). Recent advances of thiazole hybrids in biological applications. Future Med. Chem..

[16] Sharma PC, Bansal KK, Sharma A, Sharma D, Deep A (2020). Thiazole-containing compounds as therapeutic targets for cancer therapy. Eur. J. Med. Chem..

[17] Altaf AA (2015). A review on the medicinal importance of pyridine derivatives. J. Drug Des. Med. Chem..

[18] André F (2019). Alpelisib for PIK3CA-mutated, hormone receptor-positive advanced breast cancer. N. Engl. J. Med..

[19] Vitaku E, Smith DT, Njardarson JT (2014). Analysis of the structural diversity, substitution patterns, and frequency of nitrogen heterocycles among U. S. FDA approved pharmaceuticals. J. Med. Chem..

[20] Nishio M (1989). Karnamicin, a complex of new antifungal antibiotics. I. taxonomy, fermentation, isolation and physico-chemical and biological properties. J. Antibiot..

[21] Umemura K, Watanabe K, Ono K, Yamaura M, Yoshimura J (1997). Total synthesis of antibiotic karnamicin B_1_. Tetrahedron Lett..

[22] Besemer J, Lomsadze A, Borodovsky M (2001). GeneMarkS: a self-training method for prediction of gene starts in microbial genomes. Implications for finding sequence motifs in regulatory regions. Nucleic Acids Res..

[23] Blin K (2017). antiSMASH 4.0-improvements in chemistry prediction and gene cluster boundary identification. Nucleic Acids Res..

[24] Skinnider MA, Merwin NJ, Johnston CW, Magarvey NA (2017). PRISM 3: expanded prediction of natural product chemical structures from microbial genomes. Nucleic Acids Res..

[25] Qu X (2012). Caerulomycins and collismycins share a common paradigm for 2,2′-bipyridine biosynthesis via an unusual hybrid polyketide-peptide assembly logic. J. Am. Chem. Soc..

[26] Pang B (2021). Caerulomycin and collismycin antibiotics share a trans-acting flavoprotein-dependent assembly line for 2,2’-bipyridine formation. Nat. Commun..

[27] Garcia I (2012). Elucidating the biosynthetic pathway for the polyketide-nonribosomal peptide collismycin A: mechanism for formation of the 2,2′-bipyridyl ring. Chem. Biol..

[28] Zhao J (2017). *Lechevalieria rhizosphaerae* sp. nov., a novel actinomycete isolated from rhizosphere soil of wheat (*Triticum aestivum* L.) and emended description of the genus *Lechevalieria*. Int. J. Syst. Evol. Microbiol..

[29] Yu Z (2021). Secondary metabolites and genetic system of the rare actinobacteria *Lechevalieria rhizosphaerae* NEAU-A2. Microbiol. China.

[30] Chen H, O’Connor S, Cane DE, Walsh CT (2001). Epothilone biosynthesis: assembly of the methylthiazolylcarboxy starter unit on the EpoB subunit. Chem. Biol..

[31] Dowling DP (2016). Structural elements of an NRPS cyclization domain and its intermodule docking domain. Proc. Natl Acad. Sci. USA.

[32] Schneider TL, Walsh CT (2004). Portability of oxidase domains in nonribosomal peptide synthetase modules. Biochemistry.

[33] Gust B, Challis GL, Fowler K, Kieser T, Chater KF (2003). PCR-targeted *Streptomyces* gene replacement identifies a protein domain needed for biosynthesis of the sesquiterpene soil odor geosmin. Proc. Natl Acad. Sci. USA.

[34] Chen M, Pang B, Du YN, Zhang YP, Liu W (2017). Characterization of the metallo-dependent amidohydrolases responsible for “auxiliary” leucinyl removal in the biosynthesis of 2,2′-bipyridine antibiotics. Synth. Syst. Biotechnol..

[35] Eppink MHM, Schreuder HA, van Berkel WJH (1997). Identification of a novel conserved sequence motif in flavoprotein hydroxylases with a putative dual function in FAD / NAD(P)H binding. Protein Sci..

[36] Van Berkel WJH, Kamerbeek NM, Fraaije MW (2006). Flavoprotein monooxygenases, a diverse class of oxidative biocatalysts. J. Biotechnol..

[37] Westphal AH (2018). Pyridine nucleotide coenzyme specificity of *p*-hydroxybenzoate hydroxylase and related flavoprotein monooxygenases. Front. Microbiol..

[38] Westphal AH, Tischler D, van Berkel WJH (2021). Natural diversity of FAD-dependent 4-hydroxybenzoate hydroxylases. Arch. Bioche. Biophys..

[39] Treiber N, Schulz GE (2008). Structure of 2,6-dihydroxypyridine 3-hydroxylase from a nicotine-degrading pathway. J. Mol. Biol..

[40] Chen M (2017). Enzymatic competition and cooperation branch the caerulomycin biosynthetic pathway toward different 2,2′-bipyridine members. Org. Biomol. Chem..

[41] Paul CE, Eggerichs D, Westphal AH, Tischler D, van Berkel WJ (2021). Flavoprotein monooxygenases: versatile biocatalysts. Biotechnol. Adv..

[42] McCulloch KM, Mukherjee T, Begley TP, Ealick SE (2009). Structure of the PLP degradative enzyme 2-methyl-3-hydroxypyridine-5-carboxylic acid oxygenase from *Mesorhizobium loti* MAFF303099 and its mechanistic implications. Biochemistry.

[43] Hicks KA, O′Leary SE, Begley TP, Ealick SE (2013). Structural and mechanistic studies of HpxO, a novel flavin adenine dinucleotide-dependent urate oxidase from *Klebsiella pneumoniae*. Biochemistry.

[44] Jumper J (2021). Highly accurate protein structure prediction with AlphaFold. Nature.

[45] Gatti DL (1994). The mobile flavin of 4-OH benzoate hydroxylase. Science.

[46] Entsch B, Van Berkel WJH (1995). Structure and mechanism of para-hydroxybenzoate hydroxylase. FASEB J..

[47] Palfey BA, Moran GR, Entsch B, Ballou DP, Massey V (1999). Substrate recognition by “password” in p-hydroxybenzoate hydroxylase. Biochemistry.

[48] Entsch BE, Ballou DP, Massey V (1976). Flavin-oxygen derivatives involved in hydroxylation by *p*-hydroxybenzoate hydroxylase. J. Biol. Chem..

[49] Gust B (2004). Lambda red-mediated genetic manipulation of antibiotic-producing *Streptomyces*. Adv. Appl. Microbiol.

[50] Deng Y, Yang CH, Smith Iii AB (2021). Enantioselective total synthesis of (+)-peniciketals A and B: two architecturally complex spiroketals. J. Am. Chem. Soc..

[51] Eberhardt J, Santos-Martins D, Tillack AF, Forli S (2021). AutoDock Vina 1.2.0: new docking methods, expanded force field, and python bindings. J. Chem. Inf. Model..

[52] Buttery JE, Stuart S (1993). Assessment and optimization of kinetic methods for angiotensin-converting enzyme in plasma. Clin. Chem..

[53] Bramucci M (1997). Different modulation of steroidogenesis and prostaglandin production in frog ovary in vitro by ACE and ANG II. Am. J. Physiol..

[54] Gorski TP, Campbell DJ (1991). Angiotensin-converting enzyme determination in plasma during therapy with converting enzyme inhibitor: two methods compared. Clin. Chem..

