# Supplementary material for: Discovery and biosynthesis of karnamicins as angiotensin converting enzyme inhibitors
Source: Nat Commun. 2023 Jan 13;14:209. doi: 10.1038/s41467-023-35829-1 (PMC9838390; doi:10.1038/s41467-023-35829-1)
Supplement: Supplementary file 1 — Supplementary Information [file 41467_2023_35829_MOESM1_ESM.pdf]

Supplementary Information

Discovery and biosynthesis of karnamicins as angiotensin converting enzyme inhibitors

Zhiyin Yu, Jian-Ping Huang, Jing Yang, Chongxi Liu, Yijun Yan, Li Wang, Junwei Zhao, Yin Chen, Wensheng Xiang, Sheng-Xiong Huang

Correspondence and requests for materials should be addressed to S.-X.H. and W. X. (email: sxhuang@mail.kib.ac.cn; xiangwensheng@neau.edu.cn)

This PDF file includes:

Supplementary Methods

Supplementary Tables 1-26

Supplementary Figures 1-150

Supplementary References

Supplementary Methods

General experimental procedures

NMR spectra were recorded using Bruker AVANCE III-600 spectrometer or AV 800 MHz (Bruker Corp., Switzerland), and tetramethylsilane (TMS) was used as internal standard. HRESIMS data were obtained using an Agilent G6230 Q-TOF mass instrument (Agilent Corp., USA). Optical rotation data were determined in MeOH on an Autopol VI S2 & Plus polarimeter (Rudolph Research Analytical, Hackettstown, USA). X-ray crystallographic analysis was carried out with a Bruker APEX DUO single crystal X-ray diffractometer using Mo Kα radiation (Bruker Corp., Switzerland). Thin-layer chromatography (TLC) was performed using precoated silica gel GF254 plates (0.25 mm Qingdao Marine Chemical Inc., China), and spots were visualized by UV light (254 nm) and colored by spraying heated silica gel plates with 10% H_2SO_4 in ethanol. Semipreparative HPLC was conducted on a HITACHI Chromaster system equipped with a DAD detector, an YMC-Hydrosphere C_{18} column (250 mm x 10 mm i.d., 5 μm) at a flow rate of 3.0 mL/min. HPLC analysis was carried out on HITACHI Chromaster system equipped with a DAD detector, a YMC-Triart C_{18} column (250 mm x 4.6 mm i.d., 5 μm, Japan) at a flow rate of 1.0 mL/min and a column temperature of 25 °C.

DNA Sequencing and primer synthesis were conducted by TsingKe Biological Technology Co (China). PCR amplifications were carried out on Biometra professional thermocycler using either Taq DNA polymerase (Takara, Japan) or Pfu DNA polymerase (Vazyme, China). Recombinant proteins were purified on a GE AKTA pure system with a 5 mL Histrap HP column (Cytiva, USA).

Three ^{13}C-labeled compounds, [1-^{13}C] sodium acetate, [2-^{13}C] sodium acetate, and [1, 2-$^{13}C_2$] sodium acetate, were purchased from Cambridge Isotope Laboratories, Inc. (USA). Angiotensin converting enzyme (SLBQ5896V), Captopril (BCBS0901), HEPES (4-hydroxyethylpiperazine ethanesulfonic acid, SLBV6923) and FAPGG (Furan acryloyl tripeptide, SLBS2881V), were purchased from Sigma-Aldrich Co. (USA). ACE inhibitory activities were recorded using a Multifunctional microplate reader (FlexStation3, USA).

Protein expression and purification

The genes encoding *knmB1*, *knmB2* and *knmF* were amplified by PCR from genomic DNA of *L. rhizosphaerae* NEAU-A2 with primers listed in Supplementary Table 22. The gene *knmF* was cloned into the pET-28a vector using the *Nde*I and *Sal*I restriction sites. The genes *knmB1* and *knmB2* were cloned into the pET-32a vector using the *Hin*dIII and *Bam*HI restriction sites. Single amino acid mutants of KnmB1 and KnmB2 were amplified by PCR from constructed pET32a plasmid with primers listed in Supplementary Table 23.

The resulting constructs of *knmF* and *knmB1* (or *knmB1* mutants) were used to transform into *Escherichia coli* BL21(DE3) cells, and cultivated in 500 mL LB media containing kanamycin (50 μg/mL) or ampicillin (100 μg/mL) at 37 °C until the OD_{600} reached 0.6. The cultures were cooled to 16 °C and induced with 0.3 mM isopropyl-*β*-D-thiogalactopyranoside (IPTG) for 18 h at 16 °C. The resulting construct of *knmB2* or

knmB2 mutant was used to transform into *E. coli* BL21(DE3) which harboring pGro7 (Takara) for expression, and cultivated in 500 mL LB media containing chloromycetin (25 μg/mL) and ampicillin (100 μg/mL) at 37 °C until the OD_{600} reached 0.6. The cultures were cooled to 16 °C and induced with 0.3 mM IPTG and 2 mM L-arabinose for 18 h at 16 °C.

The cells were centrifuged for 20 min at 3,488 x g at 4 °C and the pellet was resuspended in 50 mL of lysis buffer A (50 mM Tris, 300 mM NaCl, 15 mM imidazole, 10% glycerol, pH 8.0) and lysed on ice by sonication. The cell lysates were centrifugated at 68,905 x g for 40 min and the supernatant was filtered (0.22 μm filter) and purified using the AKTA pure system with a 5 mL Histrap^{TM} FF column (GE Healthcare). The target proteins were eluted at a flow rate of 2 mL/min over 15 min with a linear gradient from 15 to 500 mM imidazole in buffer A and buffer B (0–5 min, 100% buffer A; 5–10 min, 50% buffer A, 50% buffer B; 10–12 min, 100% buffer B; 12–15 min, 100% buffer A. buffer B: 500 mM imidazole, 50 mM Tris, 300 mM NaCl, 10% glycerol, pH = 8.0). The target proteins were concentrated by ultrafiltration using Amicon Ultra-4 (10 K, Millipore) and stored at −80 °C in storage buffer (100 mM NaH_2PO_4, 10% glycerol, pH 7.2). Protein concentrations were determined by 280 nm absorbance (Nanodrop 2000c, Fisher Scientific). SDS-PAGE analysis of proteins was shown in Supplementary Fig. 117.

Chemical synthesis of compounds 29 and 31

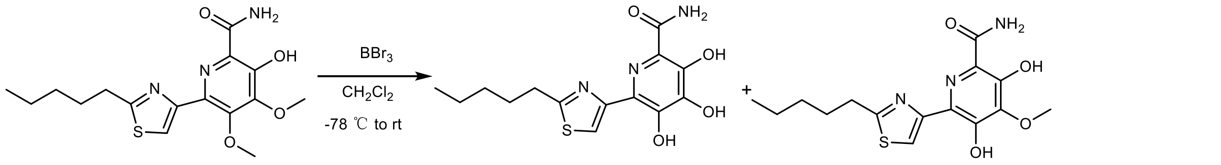

2 29 31

To a solution of **2** (20 mg) in DCM (2 mL) was added BBr_3 (500 μL) at −78 °C under N_2 atmosphere[1]. The reaction was stirred at −78 °C for 30 minutes and was then warmed up to rt. The reaction was then stirred at rt for 2 h. The reaction mixture was slowly added into 4 mL saturated $NaHCO_3$ solution at 0 °C. The aqueous layer was extracted by ethyl acetate for three times and the combined organic layers were purified using reverse-phase HPLC to afford **31** (1.6 mg) and **29** (7.6 mg). Compound **29**: HRESIMS, ^{1}H, ^{13}C, COSY, HSQC and HMBC NMR data, see Supplementary Table 24 and Supplementary Figs 133-138. Compound **31**: HRESIMS, ^{1}H, ^{13}C, COSY, HSQC and HMBC NMR data, see Supplementary Table 25 and Supplementary Figs 139-144. The spectral data of **29** and **31** are also reported in the section "Spectral data for new compounds".

In vitro preparation 30 and 34

To isolate the product **30** for structure determination, in vitro enzymatic reactions were performed as described above. Dozens of 100 μL reaction mixtures containing 50 mM Tris-HCl buffer (pH 7.5), 1 mM **28**, 2 mM SAM and 2 μM KnmF, were incubated at 30 °C for 15 min. The reaction was quenched and further isolated by HPLC directly to afford **30** (1 mg) using an isocratic elution of 60% acetonitrile (v/v) containing 0.1%

acetic acid (v/v) with a flow rate of 1 mL/min. Compound **30**: HRESIMS, ^{1}H NMR and ^{13}C NMR data see Supplementary Table 20 and Supplementary Figs 118-123. The spectral data of **30** are also reported in the section "Spectral data for new compounds".

To isolate the product **34** for structure determination, in vitro enzymatic reactions were performed as described above. Dozens of 200 μL enzymatic reactions containing 50 mM Tris-HCl buffer (pH 7.5), 1 mM **25**, 2 mM SAM and 20 μM KnmF, were incubated at 30 °C for 30 h. The reaction was quenched and further isolated by HPLC directly to afford **34** (0.8 mg) using an isocratic elution of 55% acetonitrile (v/v) with a flow rate of 1 mL/min. Compound **34**: HRESIMS, ^{1}H NMR and ^{13}C NMR data see Supplementary Table 26 and Supplementary Figs 145-150. The spectral data of **34** are also reported in the section "Spectral data for new compounds".

FAD detection

KnmB2 (83 μM) or variants (83 μM) was dissolved in storage buffer to give a final volume of 200 μL, and KnmB1 (86 μM) and variants (86 μM) were treated in the same way to obtain a final volume of 200 μL. FAD content was determined by boiling protein samples for 10 min in the dark, followed by centrifugation at 13,523 x g for 10 min to remove coagulated protein. The absorbance of the released FAD was measured at 450 nm by NanoDrop 2000c (Fisher Scientific)[2-3]. The level of FAD was calculated according to a standard concentration curve from FAD standard.

Hydrogen peroxide formation assay and NADPH oxidation

The hydrogen peroxide formed from KnmB1, KnmB2 and their variants were measured using the Hydrogen Peroxide Assay Kit (Beyotime Industrial Co. Ltd, Shanghai, China)[4-5]. 5–100 μL of enzyme reaction mixture was added to 100 μL of working reagent (S0038-1). The mixture was incubated for 30 min at 25 °C, at which time the absorbance at 560 nm was measured by NanoDrop 2000c (Fisher Scientific). The level of H_2O_2 was calculated according to a standard concentration curve from standard solution.

NADPH detection was carried out in the cuvette by NanoDrop 2000c (Fisher Scientific). NADPH signal at 340 nm was monitored in time using 6220 M^{-1} cm^{-1} as extinction coefficient[6].

Spectral data for new compounds

Karnamicin E_1 (**1**): white amorphous solid; $[\alpha]_D^{25.0}$ -35.62 (c 0.1, MeOH); ^{1}H NMR (600 MHz, $CDCl_3$) δ 12.51 (s, 1H), 8.67 (s, 1H), 7.82 (s, 1H), 5.68 (s, 1H), 4.11 (s, 3H), 3.95 (s, 3H), 3.08 (t, J = 7.9 Hz, 2H), 1.81 (m, 2H), 1.54 (m, 1H), 1.43 (m, 2H), 1.23 (m, 2H), 0.87 (d, J = 6.6 Hz, 6H); ^{13}C NMR (150 MHz, $CDCl_3$) δ 172.0, 171.3, 152.8, 150.7, 150.4, 148.0, 137.9, 127.3, 118.6, 61.1, 61.0, 38.7, 33.6, 30.6, 28.0, 27.1, 22.7. UV (MeOH) λ_{max} (log ε): 220 (4.40), 243 (4.36), 316 (3.84) nm; HRMS (*m/z*): $[M+H]^+$ calcd. for $C_{18}H_{26}N_3O_4S$, 380.1639; found, 380.1648; analysis (calcd., found for $C_{18}H_{25}N_3O_4S$): C (56.97), H (6.64), N (11.07), O (16.86), S (8.45).

Karnamicin E_2 (**2**): white amorphous solid; $[\alpha]_D^{25.0}$ -34.28 (c 0.1, MeOH); ^{1}H NMR (600 MHz, $CDCl_3$) δ 12.48 (s, 1H), 8.59 (s, 1H), 7.81 (s, 1H), 5.73 (s, 1H), 4.11 (s, 3H), 3.95 (s, 3H), 3.06 (t, J = 7.9 Hz, 2H), 1.82 (m, 2H), 1.42 (m, 2H), 1.38 (m, 2H), 0.90 (t, J = 7.1 Hz, 3H); ^{13}C NMR (150 MHz, $CDCl_3$) δ 172.0, 171.3, 152.8, 150.7, 150.4, 148.0,

137.9, 127.3, 118.6, 61.1, 61.0, 33.5, 31.5, 30.0, 22.5, 14.1; UV (MeOH) λ_{max} (log ε): 224 (4.60), 243 (4.61), 317 (4.13) nm; HRMS (*m/z*): $[M+Na]^+$ calcd. for $C_{16}H_{21}N_3O_4SNa$, 374.1145; found, 374.1148; analysis (calcd., found for $C_{16}H_{21}N_3O_4S$): C (54.69), H (6.02), N (11.96); O (18.21), S (9.12).

Karnamicin E_3 (**3**): white amorphous solid; $[\alpha]_D^{25.0}$ -30.62 (c 0.1, MeOH); ^{1}H NMR (600 MHz, $CDCl_3$) δ 12.43 (s, 1H), 8.41 (s, 1H), 7.79 (s, 1H), 5.64 (s, 1H), 4.12 (s, 3H), 3.94 (s, 3H), 3.09 (m, 2H), 1.87 (m, 1H), 1.65 (m, 1H), 1.49 (m, 1H), 1.42 (m, 1H), 1.23 (m, 1H), 0.95 (d, *J* = 6.6 Hz, 3H), 0.90 (t, *J* = 7.3 Hz, 3H); ^{13}C NMR (150 MHz, $CDCl_3$) δ 171.9, 171.5, 152.8, 150.8, 150.4, 148.1, 138.1, 127.2, 118.6, 61.2, 61.0, 37.1, 34.3, 31.4, 29.4, 19.1, 11.5; UV (MeOH) λ_{max} (log ε): 220 (4.42), 243 (4.36), 311 (3.90) nm; HRMS (*m/z*): $[M+H]^+$ calcd. for $C_{17}H_{24}N_3O_4S$, 366.1482; found, 366.1492; analysis (calcd., found for $C_{17}H_{23}N_3O_4S$): C (55.87), H (6.34), N (11.50), O (17.51), S (8.77).

Karnamicin E_4 (**4**): white amorphous solid; $[\alpha]_D^{25.0}$ -32.64 (c 0.1, MeOH); ^{1}H NMR (600 MHz, $CDCl_3$) δ 8.49 (s, 1H), 7.82 (s, 1H), 5.85 (s, 1H), 4.12 (s, 3H), 3.96 (s, 3H), 3.07 (t, *J* = 7.9 Hz, 2H), 1.83 (m, 2H), 1.60 (m, 1H), 1.33 (m, 2H), 0.90 (d, *J* = 6.6 Hz, 6H); ^{13}C NMR (150 MHz, $CDCl_3$) δ 172.0, 171.5, 152.8, 150.8, 150.3, 148.0, 137.9, 127.2, 118.6, 61.2, 61.0, 38.5, 33.7, 28.2, 27.9, 22.7; UV (MeOH) λ_{max} (log ε): 220 (4.45), 244 (4.41), 314 (3.91) nm; HRMS (*m/z*): $[M+H]^+$ calcd. for $C_{17}H_{24}N_3O_4S$, 366.1482; found, 366.1489; analysis (calcd., found for $C_{17}H_{23}N_3O_4S$): C (55.87), H (6.34), N (11.50), O (17.51), S (8.77).

Karnamicin E_5 (**5**): white amorphous solid; $[\alpha]_D^{25.0}$ -33.28 (c 0.1, MeOH); ^{1}H NMR (600 MHz, $CDCl_3$) δ 12.44 (s, 1H), 8.48 (s, 1H), 7.80 (s, 1H), 5.68 (s, 1H), 4.12 (s, 3H), 3.95 (s, 3H), 3.09 (t, *J* = 7.5 Hz, 2H), 1.73 (m, 2H), 1.70 (m, 1H), 0.97 (d, *J* = 6.3 Hz, 6H); ^{13}C NMR (150 MHz, $CDCl_3$) δ 172.0, 171.4, 152.8, 150.8, 150.4, 148.0, 138.0, 127.2, 118.6, 61.2, 61.0, 39.3, 31.6, 28.0, 22.5; UV (MeOH) λ_{max} (log ε): 220 (4.40), 244 (4.37), 317(3.85) nm; HRMS (*m/z*) $[M+H]^+$ calcd. for $C_{16}H_{22}N_3O_4S$, 352.1326; found, 352.1335; analysis (calcd., found for $C_{16}H_{21}N_3O_4S$): C (54.69), H (6.02), N (11.96), O (18.21), S (9.12).

Karnamicin E_6 (**6**): white amorphous solid; $[\alpha]_D^{25.0}$ -34.12 (c 0.1, MeOH); ^{1}H NMR (600 MHz, $CDCl_3$) δ 12.48 (s, 1H), 8.59 (s, 1H), 7.81 (s, 1H), 5.65 (s, 1H), 4.11 (s, 3H), 3.95 (s, 3H), 3.07 (t, *J* = 7.7 Hz, 2H), 1.82 (m, 2H), 1.42 (m, 2H), 1.32 (m, 4H), 0.89 (t, *J* = 6.8 Hz, 3H); ^{13}C NMR (150 MHz, $CDCl_3$) δ 172.0, 171.2, 152.8, 150.8, 150.4, 148.0, 138.0, 127.3, 118.6, 61.2, 61.0, 33.6, 31.6, 30.3, 29.0, 22.7, 14.2; UV (MeOH) λ_{max} (log ε): 220 (4.42), 243 (4.39), 317(3.86) nm; HRMS (*m/z*): $[M+H]^+$ calcd. for $C_{17}H_{24}N_3O_4S$, 366.1482; found, 366.1493; analysis (calcd., found for $C_{17}H_{23}N_3O_4S$): C (55.87), H (6.34), N (11.50), O (17.51), S (8.77).

Compound **20**: white amorphous solid; ^{1}H NMR (600 MHz, MeOD) δ 8.22 (s, 1H), 7.58 (d, *J* = 1.5 Hz, 1H), 7.45 (d, *J* = 1.5 Hz, 1H), 4.69 (dd, *J* = 9.7, 4.4 Hz, 1H), 3.10 (t, *J* = 7.6 Hz, 2H), 1.88 (m, 1H), 1.83 (m, 2H), 1.77 (m, 2H), 1.57 (m, 1H), 1.46 (m, 2H), 1.26 (m, 2H), 1.00 (d, *J* = 6.1 Hz, 3H), 0.99 (d, J = 6.1 Hz, 3H), 0.90 (d, *J* = 6.6 Hz, 6H); ^{13}C NMR (150 MHz, MeOD) δ 176.4, 173.9, 168.0, 166.5, 154.9, 154.2, 152.0, 118.9, 111.7, 110.2, 52.7, 42.0, 39.8, 34.2, 31.5, 29.1, 27.9, 26.3, 23.5, 23.0, 22.1; HRMS (*m/z*): $[M+H]^+$ calcd. for $C_{22}H_{32}N_3O_4S$, 434.2108; found, 434.2117; analysis (calcd., found for $C_{22}H_{31}N_3O_4S$): C (61.72), H (7.43), N (9.39), O (14.30), S (7.16).

Compound **21**: white amorphous solid; ^{1}H NMR (600 MHz, MeOD) *δ* 8.21 (s, 1H), 7.57 (d, *J* = 2.2 Hz, 1H), 7.44 (d, *J* = 2.2 Hz, 1H), 4.70 (dd, *J* = 9.8, 4.5 Hz, 1H), 3.11 (t, *J* = 7.6 Hz, 2H), 1.88 (m, 1H), 1.85 (m, 2H), 1.80 (m, 1H), 1.77 (m, 1H), 1.53 (m, 4H), 1.18 (s, 6H), 1.00 (d, *J* = 6.5 Hz, 3H), 0.99 (d, *J* = 6.5 Hz, 3H); ^{13}C NMR (150 MHz, MeOD) *δ* 176.1, 173.8, 168.2, 166.5, 154.9, 154.1, 152.1, 118.9, 111.7, 110.2, 71.3, 52.5, 44.3, 41.9, 34.2, 31.9, 29.2, 26.3, 24.9, 23.4, 22.1; HRMS (*m/z*): $[M+Na]^+$ calcd. for $C_{22}H_{31}N_3O_5SNa$, 472.1877; found, 472.1881; analysis (calcd., found for $C_{22}H_{31}N_3O_5S$): C (58.78), H (6.95), N (9.35), O (17.79), S (7.13).

Compound **22**: white amorphous solid; ^{1}H NMR (600 MHz, MeOD) *δ* 8.22 (s, 1H), 7.58 (d, *J* = 2.2 Hz, 1H), 7.44 (d, *J* = 2.2 Hz, 1H), 4.69 (dd, *J* = 9.8, 4.5 Hz, 1H), 3.09 (t, *J* = 7.7 Hz, 2H), 1.89 (m, 1H), 1.86 (m, 2H), 1.82 (m, 1H), 1.77 (m, 1H), 1.45 (m, 2H), 1.41 (m, 2H), 1.00 (d, *J* = 6.2 Hz, 3H), 0.99 (d, *J* = 6.2 Hz, 3H), 0.94 (t, *J* = 7.1 Hz, 3H); ^{13}C NMR (150 MHz, MeOD) *δ* 176.3, 174.0, 168.1, 166.5, 154.9, 154.1, 152.1, 118.9, 111.7, 110.2, 52.6, 42.0, 34.2, 32.4, 31.0, 26.3, 23.4, 23.4, 22.1, 14.3; HRMS (m/z): $[M+Na]^+$ calcd. for $C_{20}H_{27}N_3O_4SNa$, 428.1614; found, 428.1612; analysis (calcd., found for $C_{20}H_{27}N_3O_4S$): C (59.24), H (6.71), N (10.36), O (15.78), S, (7.91).

Compound **24**: white amorphous solid; ^{1}H NMR (600 MHz, DMSO-*d6*) *δ* 8.57 (s, 1H), 8.35 (s, 1H), 7.60 (s, 1H), 7.54 (d, *J* = 2.2 Hz, 1H), 7.31 (d, *J* = 2.2 Hz, 1H), 3.03 (t, *J* = 7.6 Hz, 2H), 1.74 (m, 2H), 1.52 (m, 1H), 1.37 (m, 2H), 1.21 (m, 2H), 0.85 (d, *J* = 6.6 Hz, 6H); ^{13}C NMR (150 MHz, DMSO-*d6*) *δ* 170.9, 167.0, 166.1, 153.8, 152.4, 151.8, 118.1, 110.0, 108.6, 38.1, 32.7, 29.6, 27.3, 26.2, 22.5; HRMS (*m/z*): $[M+Na]^+$ calcd. for $C_{16}H_{21}N_3O_2SNa$, 342.1247; found, 342.1251; analysis (calcd., found for $C_{16}H_{21}N_3O_2S$): C (60.16), H (6.63), N (13.16), O (10.02), S (10.04).

Compound **25**: white amorphous solid; ^{1}H NMR (600 MHz, DMSO-*d6*) *δ* 8.58 (s, 1H), 8.35 (s, 1H), 7.61 (s, 1H), 7.55 (d, *J* = 2.2 Hz, 1H), 7.31 (d, *J* = 2.2 Hz, 1H), 3.03 (t, *J* = 7.6 Hz, 2H), 1.77 (m, 2H), 1.37 (m, 2H), 1.34 (m, 2H), 0.88 (t, *J* = 7.0 Hz, 3H); ^{13}C NMR (150 MHz, DMSO-*d6*) *δ* 170.9, 166.8, 166.1, 153.7, 152.4, 151.8, 118.1, 110.0, 108.6, 32.7, 30.6, 29.1, 21.8, 13.9; HRMS (*m/z*): $[M+Na]^+$ calcd. for $C_{14}H_{17}N_3O_2SNa$, 314.0934; found, 314.0936; analysis (calcd., found for $C_{14}H_{17}N_3O_2S$): C (57.71), H (5.88), N (14.42), O (10.98), S (11.00).

Compound **26**: white amorphous solid; ^{1}H NMR (600 MHz, MeOD) *δ* 8.23 (s, 1H), 7.57 (d, *J* = 2.0 Hz, 1H), 7.45 (d, *J* = 2.0 Hz, 1H), 3.07 (t, *J* = 7.6 Hz, 2H), 1.85 (m, 2H), 1.62 (m, 1H), 1.34 (m, 2H), 0.93 (d, *J* = 6.6 Hz, 6H); ^{13}C NMR (150 MHz, MeOD) *δ* 173.9, 169.4, 168.4, 155.0, 154.0, 152.2, 118.9, 111.6, 110.3, 39.4, 34.4, 29.2, 29.0, 22.9; HRMS (*m/z*): $[M+Na]^+$ (calcd. for $C_{15}H_{19}N_3O_2SNa$, 328.1090; found, 328.1098; analysis (calcd., found for $C_{15}H_{19}N_3O_2S$): C (58.99), H (6.27), N (13.76), O (10.48), S (10.50).

Compound **28**: white amorphous solid; ^{1}H NMR (600 MHz, acetone-*d6*) *δ* 12.63 (s, 1H), 12.12 (s, 1H), 8.36 (s, 1H), 7.19 (s, 1H), 3.18 (t, *J* = 7.6 Hz, 2H), 1.86 (m, 2H), 1.57 (m, 1H), 1.47 (m, 2H), 1.28 (m, 2H), 0.88 (d, *J* = 6.6 Hz, 6H); ^{13}C NMR (150 MHz, acetone-*d6*) *δ* 172.9, 172.9, 154.2, 149.2, 148.9, 141.2, 129.6, 124.9, 115.7, 39.3, 33.4, 30.7, 28.6, 27.5, 22.9; HRMS (*m/z*): $[M+H]^+$ calcd. for $C_{16}H_{22}N_3O_4S$, 352.1326; found, 352.1329; analysis (calcd., found for $C_{16}H_{21}N_3O_4S$): C (54.69), H (6.02), N (11.96), O (18.21), S (9.12).

Compound **29**: white amorphous solid; ^{1}H NMR (600 MHz, DMSO-*d$_6$*) *δ* 12.79 (s, 1H), 12.02 (s, 1H), 8.58 (s, 2H), 8.06 (s, 1H), 3.11 (t, *J* = 7.5 Hz, 2H), 1.78 (m, 2H), 1.35 (m, 4H), 0.87 (t, *J* = 6.9 Hz, 3H). ^{13}C NMR (150 MHz, DMSO-*d$_6$*) *δ* 171.9, 171.6, 152.6, 148.0, 144.5, 140.1, 128.3, 123.8, 115.8, 32.0, 30.5, 28.7, 21.8, 13.8; HRMS (*m/z*): [M+H]$^+$ calcd. for $C_{14}H_{18}N_3O_4S$, 324.1013; found, 324.1019; analysis (calcd., found for $C_{14}H_{17}N_3O_4S$): C (52.00), H (5.30), N (12.99), O (19.79), S (9.91).

Compound **30**: white amorphous solid; ^{1}H NMR (800 MHz, $CDCl_3$) *δ* 12.55 (s, 1H), 12.12 (s, 1H), 7.78 (s, 1H), 7.63 (s, 1H), 5.56 (s, 1H), 4.12 (s, 3H), 3.08 (t, *J* = 7.7 Hz, 2H), 1.84 (m, 1H), 1.55 (m, 1H), 1.42 (m, 2H), 1.26 – 1.22 (m, 3H), 0.88 (d, *J* = 6.6 Hz, 6H); ^{13}C NMR (200 MHz, $CDCl_3$) *δ* 171.9, 171.7, 153.0, 152.9, 150.5, 142.3, 130.0, 124.0, 113.9, 60.8, 38.7, 33.2, 29.9, 28.0, 26.9, 22.7; HRMS (*m/z*): [M+H]$^+$ calcd. for $C_{17}H_{24}N_3O_4S$, 366.1482; found, 366.1477; analysis (calcd., found for $C_{17}H_{23}N_3O_4S$): C (55.87), H (6.34), N (11.50), O (17.51), S (8.77).

Compound **31**: white amorphous solid; ^{1}H NMR (600 MHz, DMSO-*d$_6$*) *δ* 13.07 (s, 1H), 12.33 (s, 1H), 8.64 (s, 2H), 8.12 (s, 1H), 3.91 (s, 3H), 3.12 (t, *J* = 7.5 Hz, 2H), 1.78 (m, 2H), 1.35 (m, 4H), 0.88 (t, *J* = 7.0 Hz, 3H); ^{13}C NMR (150 MHz, DMSO-*d$_6$*) *δ* 171.8, 171.7, 152.2, 152.1, 149.3, 141.4, 129.2, 124.1, 116.2, 59.8, 32.0, 30.5, 28.8, 21.8, 13.9; HRMS (*m/z*): [M+H]$^+$ calcd. for $C_{15}H_{20}N_3O_4S$, 338.1169; found, 338.1175; analysis (calcd., found for $C_{15}H_{19}N_3O_4S$): C (53.40), H (5.68), N (12.45), O (18.97), S (9.50).

Compound **32**: white amorphous solid; ^{1}H NMR (600 MHz, DMSO-*d$_6$*) *δ* 8.69 (s, 1H), 8.45 (s, 1H), 7.73 (s, 1H), 7.65 (d, *J* = 2.4 Hz, 1H), 7.48 (d, *J* = 2.4 Hz, 1H), 3.95 (s, 3H), 3.05 (t, *J* = 7.6 Hz, 2H), 1.75 (m, 2H), 1.53 (m, 1H), 1.39 (m, 2H), 1.22 (m, 2H), 0.85 (d, *J* = 6.6 Hz, 6H); ^{13}C NMR (150 MHz, DMSO-*d$_6$*) *δ* 171.3, 167.5, 165.7, 153.2, 152.6, 152.1, 119.0, 108.1, 106.6, 55.8, 38.0, 32.7, 29.7, 27.3, 26.2, 22.5. HRMS (*m/z*): [M+H]$^+$ calcd. for $C_{17}H_{24}N_3O_2S$, 334.1584; found, 334.1586; analysis (calcd., found for $C_{17}H_{23}N_3O_2S$): C (61.23), H (6.95), N (12.60), O (9.60), S (9.61).

Compound **33**: white amorphous solid; ^{1}H NMR (600 MHz, DMSO-*d$_6$*) *δ* 8.69 (s, 1H), 8.45 (s, 1H), 7.73 (s, 1H), 7.65 (d, *J* = 2.3 Hz, 1H), 7.48 (d, *J* = 2.3 Hz, 1H), 3.04 (t, *J* = 7.6 Hz, 2H), 1.77 (m, 2H), 1.59 (m, 1H), 1.28 (m, 2H), 0.88 (d, *J* = 6.6 Hz, 6H); ^{13}C NMR (150 MHz, DMSO-*d$_6$*) *δ* 171.3, 167.5, 165.7, 153.2, 152.6, 152.1, 119.0, 108.1, 106.5, 55.8, 37.7, 32.9, 27.3, 27.2, 22.5; HRMS (*m/z*): [M+H]$^+$ calcd. for $C_{16}H_{22}N_3O_2S$, 320.1427; found, 320.1426; analysis (calcd., found for $C_{16}H_{21}N_3O_2S$): C (60.16), H (6.63), N (13.16), O (10.02), S (10.04).

Compound **34**: white amorphous solid; ^{1}H NMR (800 MHz, DMSO-*d$_6$*) *δ* 8.68 (s, 1H), 8.45 (s, 1H), 7.73 (s, 1H), 7.65 (d, *J* = 2.4 Hz, 1H), 7.48 (d, *J* = 2.4 Hz, 1H), 3.95 (s, 3H), 3.05 (t, *J* = 7.7 Hz, 2H), 1.77 (m, 2H), 1.36 (m, 4H), 0.88 (t, *J* = 7.1 Hz, 3H); ^{13}C NMR (200 MHz, DMSO-*d$_6$*) *δ* 171.3, 167.5, 165.7, 153.2, 152.6, 152.1, 119.0, 108.1, 106.6, 55.8, 32.7, 30.7, 29.2, 21.8, 13.9. HRMS (*m/z*): [M+H]$^+$ calcd. for $C_{15}H_{20}N_3O_2S$, 306.1271; found, 306.1275; analysis (calcd., found for $C_{15}H_{19}N_3O_2S$): C (58.99), H (6.27), N (13.76), O (10.48), S (10.50).

Compound **35**: white amorphous solid; ^{1}H NMR (600 MHz, $CDCl_3$) *δ* 12.17 (s, 1H), 7.96 (s, 1H), 7.79 (s, 1H), 7.75 (s, 1H), 5.71 (s, 1H), 4.05 (s, 3H), 3.05 (t, *J* = 7.8 Hz, 2H), 1.85 (m, 2H), 1.44 (m, 2H), 1.40 (m, 2H), 0.93 (t, *J* = 7.1 Hz, 3H); ^{13}C NMR (150

MHz, $CDCl_3$) δ 172.0, 172.0, 156.2, 154.0, 148.8, 144.5, 129.5, 115.0, 108.1, 56.5, 33.7, 31.5, 29.9, 22.5, 14.1; HRMS (*m/z*): $[M+Na]^+$ calcd for $C_{15}H_{19}N_3O_3SNa$, 344.1039; found, 344.1036; analysis (calcd., found for $C_{15}H_{19}N_3O_3S$): C (56.06), H (5.96), N (13.07), O (14.93), S (9.98).

Compound **36**: white amorphous solid; ^1H NMR (600 MHz, $CDCl_3$) δ 12.19 (s, 1H), 7.97 (s, 1H), 7.77 (s, 2H), 5.96 (s, 1H), 4.04 (s, 3H), 3.07 (t, *J* = 7.5 Hz, 2H), 2.58 (t, *J* = 7.2 Hz, 2H), 2.16 (s, 3H), 2.12 (m, 2H); ^{13}C NMR (150 MHz, $CDCl_3$) δ 208.2, 172.1, 170.6, 156.1, 154.2, 148.8, 144.4, 129.6, 115.3, 108.1, 56.5, 42.5, 32.7, 30.2, 23.9; HRMS (m/z): $[M+Na]^+$ calcd. for $C_{15}H_{17}N_3O_4SNa^+$, 358.0832; found, 358.0836; analysis (calcd., found for $C_{15}H_{17}N_3O_4S$): C (53.72), H (5.11), N (12.53), O (19.08), S (9.56).

Compound **37**: white amorphous solid; ^1H NMR (600 MHz, $CDCl_3$) δ 12.31 (s, 1H), 8.33 (s, 1H), 7.70 (s, 1H), 7.67 (s, 1H), 5.74 (s, 1H), 4.04 (s, 3H), 3.90 (m, 1H), 3.11 (m, 2H), 2.00 (m, 2H),1.62 (m, 2H), 1.22 (d, *J* = 6.2 Hz, 3H); ^{13}C NMR (150 MHz, $CDCl_3$) δ 172.2, 171.8, 156.2, 154.0, 148.9, 144.1, 129.7, 115.1, 107.7, 67.3, 56.5, 38.7, 33.2, 25.3, 23.8; HRESIMS (*m/z*): $[M+Na]^+$ calcd. for $C_{15}H_{19}N_3O_4SNa$, 360.0988; found, 360.0992; analysis (calcd., found for $C_{15}H_{19}N_3O_4S$): C (53.40), H (5.68), N (12.45), O (18.97), S (9.50).

Supplementary Tables

Supplementary Table 1. NMR data of compound **1** ($CDCl_3$, δ in ppm).

No	δc	δ_H mult (*J* in Hz)	^1H-^1H COSY	HMBC
2	127.27, C			
3	172.02, C			
4	152.84, C			
5	148.01, C			
6	150.73, C			
7	137.87, C			
8	150.38, C			
9	118.60, CH	7.82, s		C-7, 8, 11
11	171.28, C			
13	60.97, CH_3	4.11, s		C-5
14	61.12, CH_3	3.95, s		C-6
1'	33.61, CH_2	3.08, t (7.9)	H-2',	C-3', 2', 11, 8, 9
2'	30.60, CH_2	1.81, m	H-3', H-2'	C-3', 1', 4', 11
3'	27.14, CH_2	1.43, m	H-2', H-4'	C-5', 2', 1', 4'
4'	38.73, CH_2	1.23, m	H-3', H-5'	C-6', 7', 5', 2'
5'	27.99, CH	1.54, m	H-6', H-7', H-4'	C-6', 7', 3', 4'
6'	22.73, CH_3	0.87, d (6.6)	H-5'	C-7', 5', 4'
7'	22.73, CH_3	0.87, d (6.6)	H-5'	C-6', 5', 4;
NH-a		5.68, s	NH-b	C-2
NH-b		8.67, s	NH-a	
OH-4		12.51, s		C-2, 4, 5

Supplementary Table 2. NMR data of compound **2** ($CDCl_3$, δ in ppm).

No	δ_C	δ_H mult (J in Hz)	^1H-^1H COSY	HMBC
2	127.24, C			
3	171.99, C			
4	152.83, C			
5	148.02, C			
6	150.73, C			
7	137.92, C			
8	150.37, C			
9	118.60, CH	7.81, s		C-8, 11, 7
11	171.30, C			
13	60.97, CH_3	4.11, s		C-5
14	61.13, CH_3	3.95, s		C-6
1'	33.51, CH_2	3.06, t (7.9)	H-2'	C-2', 3', 9, 8, 11
2'	30.03, CH_2	1.82, m	H-1', H-3'	C-4', 3', 1', 11
3'	31.48, CH_2	1.41, m	H-4', H-2'	C-4', 2', 1', 5'
4'	22.50, CH_2	1.38, m	H-5', H-4'	C-5', 3', 2'
5'	14.09, CH_3	0.90, t (7.1)	H-4'	C-4', 3'
NH-a		5.73, s	NH-b	C-2
NH-b		8.59, s	NH-a	
OH-4		12.48, s		C-2, 4, 5

Supplementary Table 3. NMR data of compound **3** ($CDCl_3$, δ in ppm).

No.	δ_C	δ_H mult (*J* in Hz)	^1H-^1H COSY	HMBC
2	127.19, C			
3	171.88, C			
4	152.80, C			
5	150.77, C			
6	148.05, C			
7	138.09, C			
8	150.41, C			
9	118.57, CH	7.79, s		C-7, 8, 11
11	171.47, C			
13	60.98, CH_3	4.12, s		C-5
14	61.17, CH_3	3.94, s		C-6
1'	31.41, CH_2	3.09, m	H-2'	C-3', 2', 11
2'a	37.05, CH_2	1.65, m	H-2'b, H-1'	C-6', 4', 1', 3', 11
2'b		1.87, m	H-2'a, H-1', H-3'	C-6', 4', 1', 3', 11
3'	34.29, CH	1.49, m	H-6', 4', 2'	C-5', 6', 4', 1', 2'
4'a	29.36, CH_2	1.23, m	H-4'b, H-5', H-3'	C-5', 6', 3', 2'
4'b		1.42, m	H-4'a, H-5'	C-5', 6', 3', 2'
5'	11.46, CH_3	0.90, t (7.3)	H-4'	C-4', 3'
6'	19.11, CH_3	0.95, d (6.6)	H-3'	C-4', 3', 2'
NH-a		5.64, s	NH-b	
NH-b		8.41, s	NH-a	
OH-4		12.43, s		C-2, 4, 5

Supplementary Table 4. NMR data of compound **4** ($CDCl_3$, δ in ppm).

No.	δ_C	δ_H mult (*J* in Hz)	^1H-^1H COSY	HMBC
2	127.15, C			
3	172.02, C			
4	152.83, C			
5	148.03, C			
6	150.76, C			
7	137.92, C			
8	150.29, C			
9	118.62, CH	7.82, s		C-7, 8, 11
11	171.49, C			
13	60.97, CH_3	4.12, s		C-5'
14	61.15, CH_3	3.96, s		C-6'
1'	33.71, CH_2	3.07, t (7.8)	H-2'	C-2', 3', 11
2'	28.22, CH_2	1.83, m	H-1', H-3'	C-11, 4', 1', 3'
3'	38.54, CH_2	1.33, m	H-2', H-4'	C-5', 6', 2', 4', 1'
4'	27.93, CH	1.60, m	H-3', H-5', H-6'	C-5', 6', 2', 3'
5'	22.65, CH_3	0.90, d (6.6)	H-4'	C-6', 4', 3'
6'	22.65, CH_3	0.90, d (6.6)	H-4'	C-5', 4', 3'
NH-a		5.85, s	NH-b	
NH-b		8.49, s	NH-a	

Supplementary Table 5. NMR data of compound **5** ($CDCl_3$, δ in ppm).

No	δ_C	δ_H mult (J in Hz)	^1H-^1H COSY	HMBC
2	127.21, C			
3	171.95, C			
4	152.82, C			
5	148.04, C			
6	150.76, C			
7	138.00, C			
8	150.38, C			
9	118.58, CH	7.80, s		C-7, 8, 11
11	171.44, C			
13	60.98, CH_3	4.12, s		C-5
14	61.15, CH_3	3.95, s		C-6
1'	31.62, CH_2	3.09, t (7.5)	H-2'	C-3', 2',11
2'	39.30, CH_2	1.73, m	H-1', H-3'	C-4', 5', 3', 1', 11
3'	27.96, CH	1.70, m	H-2', H-4', H-5'	C-2', 4', 5', 1'
4'	22.51, CH_3	0.97, d (6.3)	H-3'	C-5', 3', 2'
5'	22.51, CH_3	0.97, d (6.3)	H-3'	C-4', 3', 2'
NH-a		5.68, s		
NH-b		8.48, s		
OH-4		12.44, s		C-2, 4, 5

Supplementary Table 6. NMR data of compound **6** ($CDCl_3$, *δ* in ppm).

No	*δc*	δ_H mult (*J* in Hz)	^1H-^1H COSY	HMBC
2	127.24, C			
3	171.96, C			
4	152.82, C			
5	148.04, C			
6	150.75, C			
7	137.99, C			
8	150.42, C			
9	118.59, CH	7.81, s		C-7, 8, 11
11	171.24, C			
13	60.97, CH_3	4.11, s		C-5
14	61.15, CH_3	3.95, s		C-6
1'	33.62, CH_2	3.07, t (7.7)	H-2'	C-2', 3', 11, 8, 9
2'	30.31, CH_2	1.82, m	H-1', H-3'	C-3', 4', 1', 11
3'	29.03, CH_2	1.42, m	H-2', H-4'	C-4', 1', 5'
4'	31.64, CH_2	1.32, overlapped	H-3', H-5'	C-5', 3', 6', 2'
5'	22.66, CH_2	1.32, overlapped	H-4', H-6'	C-6', 4', 3'
6'	14.19, CH_3	0.89, t (6.8)	H-5'	C-5', 4'
NH-a		5.65, s		C-2
NH-b		8.59, s		
OH-4		12.48, s		C-2, 4, 5

Supplementary Table 7. Deduced functions of *ORFs* in the *knm* biosynthetic gene cluster from *L. rhizosphaerae* NEAU-A2.

Genes	Size in AA	Proposed function	Protein homologue and origin	Identity/ Similarity (%)	Accssion number
knmE	431	amidohydrolase family protein	CaeD (*Actinoalloteichus cyanogriseus*)	61/74	AFK24521
knmG1	902	AAA family ATPase	HOW59_23070 (*Nonomuraea* sp.)	48/63	NUP00804
knmA4	227	thioesterase	CaeA4 (*A. cyanogriseus* NRRL B-2194)	52/64	AFK24519
knmD	385	acyl-CoA dehydrogenase	CaeB1 (*A. cyanogriseus* NRRL B-2194)	59/73	AFK24518
knmA1	1030	NRPS	CaeA3 (*A. cyanogriseus* NRRL B-2194)	48/62	AFK24517
knmA2	2405	PKS/NRPS	CaeA2 (*A. cyanogriseus* NRRL B-2194)	53/65	AFK24516
knmA3	1961	NRPS	EpoB (*Sorangium cellulosum*)	41/56	AAF62881
knmB1	367	FAD-dependent monooxygenase	CaeB6 (*A. cyanogriseus* NRRL B-2194)	55/64	AFK24512
knmB2	393	FAD-dependent monooxygenase	HpxO (*Klebsiella pneumoniae*)	31/47	3RP8
knmC	607	asparagine synthase	Asparagine Synthetase B (*Escherichia coli*)	32/48	1CT9_A
knmG2	177	MerR family transcriptional regulator	D5S18_02340 (*Nocardia panacis*)	59/73	RJO79203
knmG3	202	TetR/AcrR family transcriptional regulator	SAMN05661093_07517 (*Kibdelosporangium aridum*)	41/56	SMD22588
knmG4	412	MFS transporter	D5S19_30915 (*Amycolatopsis panacis*)	45/61	RJQ75842
knmG5	578	ABC transporter ATP-binding protein	IU483_16320 (*S. gardneri*)	61/77	MBF6205647
knmG6	596	ABC transporter ATP-binding protein	IU486_15890 (*S. gardneri*)	69/79	MBF6166235
knmF	339	O-methyltransferase	CaeG1 (*A.cyanogriseus* NRRL B-2194)	32/48	AFK24511.1

Supplementary Table 8. NMR data of compound **20** (MeOD, *δ* in ppm).

**No**	*δc*	*δ*H mult (*J* in Hz)	1H-1H COSY	HMBC
2	152.04, C			
3	166.47, C			
4	110.16, CH	7.45, d (1.5)	H-6	C-6, 5, 3
5	168.03, C			
6	111.65, CH	7.58, d (1.5)	H-4	C-4, 8, 5
7	154.19, C			
8	154.91, C			
9	118.89, CH	8.22, s		C-8, 7, 11
11	173.92, C			
1'	34.20, CH_2	3.10, t (7.6)	H-2'	C-3', 2', 11, (9, 8)
2'	31.51, CH_2	1.83, m	H-1', H-3'	C-3', 4', 11
3'	27.94, CH_2	1.46, m	H-2', H-4'	C-5', 2', 1', 4'
4'	39.77, CH_2	1.26, m	H-3', H-5'	C-6', 7', 3', 5', 2'
5'	29.06, CH	1.57, m	H-4', H-6', H-7'	C-6', 7', 3', 4'
6'	22.98, CH_3	0.90, d (6.6)	H-5'	C-7', 5', 4'
7'	22.98, CH_3	0.90, d (6.6)	H-5'	C-6', 5', 4'
1''	176.35, C			
2''	52.67, CH	4.69, dd (9.7, 4.4)	H-3'',	C-4'', 3'', 3, 1''
3''	42.02, CH_2	1.88, m	H-2'', H-4''	C-5'', 6'', 4'', 2'', 1''
		1.77, m	H-2'', H-4''	C-5'', 6'', 4'', 2'', 1''
4''	26.28, CH	1.77, m	H-3'', H-5''	C-5'', 6', 2''
5''	23.46, CH_3	1.00, d (6.1)	H-4''	C-6'', 4 '', 3''
6''	22.11, CH_3	0.99, d (6.1)	H-4''	C-5'', 4'', 3''

Supplementary Table 9. NMR data of compound **21** (MeOD, δ in ppm).

No	δc	δ_H mult (J in Hz)	^1H-^1H COSY	HMBC
2	152.06, C			
3	166.49, C			
4	110.18, CH	7.44, d (2.2)	H-6	C-6, 5, 3
5	168.18, C			
6	111.68, CH	7.57, d (2.2)	H-4	C-4, 8, 5
7	154.08, C			
8	154.92, C			
9	118.92, CH	8.21, s		C-8, 7, 11
11	173.78, C			
1'	34.17, CH_2	3.11, t (7.6)	H-2'	C-3', 2', 11, (9, 8)
2'	31.87, CH_2	1.85, m	H-1', H-3'	C-3', 4', 11,
3'	24.87, CH_2	1.53, overlapped	H-2', H-4'	C-5', 2', 1', 4'
4'	44.33, CH_2	1.53, overlapped	H-3'	C-6', 7', 3', 5', 2'
5'	71.33, C			
6'	29.18, CH_3	1.18, s		C-7', 5', 4'
7'	29.18, CH_3	1.18, s		C-6', 5', 4'
1''	176.06, C			
2''	52.47, CH	4.70, dd (9.8, 4.5)	H-3'',	C-4'',3'',3,1''
3''	41.89, CH_2	1.88, m	H-2'', H-4''	C-5'', 6'', 4'', 2'', 1''
		1.80, m	H-2'', H-4''	C-5'', 6'', 4'', 2'', 1''
4''	26.26, CH	1.77, m	H-3'', H-5'', H-6''	C-5'', 6', 2''
5''	23.43, CH_3	1.00, d (6.5)	H-4''	C-6'', 4'', 3''
6''	22.08, CH_3	0.99, d (6.5)	H-4''	C-5'', 4'', 3''

Supplementary Table 10. NMR data of compound **22** (MeOD, *δ* in ppm).

No	*δc*	δ_H mult (*J* in Hz)	^1H-^1H COSY	HMBC
2	152.14, C			
3	166.48, C			
4	110.17, CH	7.44, d (2.2)	H-6	C-6, 5, 3
5	168.10, C			
6	111.66, CH	7.58, d (2.2)	H-4	C-4, 8, 5
7	154.10, C			
8	154.92, C			
9	118.89, CH	8.22, s		C-8, 7, 11
11	173.93, C			
1'	34.16, CH_2	3.09, t (7.7)	H-2'	C-3', 2', 11, (9, 8)
2'	30.99, CH_2	1.86, m	H-1', H-3'	C-3', 1', 4', 11
3'	32.37, CH_2	1.45, m	H-2', H-4'	C-5', 2', 1', 4'
4'	23.44, CH_2	1.41, m	H-3', H-5'	C-3', 5', 2'
5'	14.29, CH_3	0.94, t (7.1)	H-4'	C-3', 4'
1''	176.26, C			
2''	52.62, CH	4.69, dd (9.8, 4.5)	H-3'',	C-4'', 3'', 3, 1''
3''	42.00, CH_2	1.89, m	H-2'', H-4''	C-5'', 6'', 4'', 2'', 1''
		1.82, m	H-2'', H-4''	C-5'', 6'', 4'', 2'', 1''
4''	26.28, CH	1.77, m	H-3'', H-5'', H-6''	C-5'', 6', 2''
5''	23.42, CH_3	1.00, d (6.2)	H-4''	C-6'', 4'', 3''
6''	22.10, CH_3	0.99, d (6.2)	H-4''	C-5'', 4'', 3''

Supplementary Table 11. NMR data of compound **24** (DMSO-d_6, δ in ppm).

No	δ_C	δ_H mult (J in Hz)	^{1}H-^{1}H COSY	HMBC
2	151.81, C			
3	166.10, C			
4	108.64, CH	7.31, d (2.2)	H-6	C-6, 3, 5
5	166.98, C			
6	110.03, CH	7.54, d (2.2)	H-4	C-4, 8, 5
7	152.37, C			
8	153.75, C			
9	118.08, CH	8.57, s		C-7, 8, 11
11	170.88, C			
1'	32.74, CH_2	3.03, t (7.6)	H-2'	C-2', 3', 9, 11, 8
2'	29.60, CH_2	1.74, m	H-3', H-1'	C-3', 1', 4', 11
3'	26.18, CH_2	1.37, m	H-4', H-2'	C-5', 2', 1', 4'
4'	38.05, CH_2	1.21, m	H-5', H-3'	C-6', 7', 2', 5'
5'	27.32, CH	1.52, m	H-4', H-6', H-7'	C-6', 7', 3', 4'
6'	22.50, CH_3	0.85, d (6.6)	H-5'	C-7', 5', 4'
7'	22.50, CH_3	0.85, d (6.6)	H-5'	C-6', 5', 4'
NH-a		7.60, s	NH-b	C-2
NH-b		8.35, s	NH-a	C-3

Supplementary Table 12. NMR data of compound **25** (DMSO-d_6, δ in ppm).

No	δ_C	δ_H mult (J in Hz)	^{1}H-^{1}H COSY	HMBC
2	151.84, C			
3	166.05, C			
4	108.57, CH	7.31, d (2.2)	H-6	C-6, 3, 5
5	166.79, C			
6	109.95, CH	7.55, d (2.2)	H-4	C-4, 8, 5
7	152.38, C			
8	153.70, C			
9	118.13, CH	8.58, s		C-7, 8, 11
11	170.92, C			
1'	32.69, CH_2	3.03, t (7.6)	H-2'	C-2', 3', 9, 11, 8
2'	29.07, CH_2	1.77, m	H-3', H-1'	C-3', 1', 4', 11
3'	30.64, CH_2	1.37, m	H-4', H-2'	C-5', 2', 1', 4'
4'	21.83, CH_2	1.34, m	H-5', H-3'	C-2', 3', 5'
5'	13.88, CH_3	0.88, t (7.0)	H-4'	C-3', 4'
NH-a		7.61, s	NH-b	C-2
NH-b		8.35, s	NH-a	C-3

Supplementary Table 13. NMR data of compound **26** (MeOD, δ in ppm).

No	δ_C	δ_H mult (J in Hz)	^1H-^1H COSY	HMBC
2	152.23, C			
3	169.35, C			
4	110.26, CH	7.45, d (2.0)	H-6	C-6, 3, 5
5	168.35, C			
6	111.61, CH	7.57, d (2.0)	H-4	C-4, 5, 8
7	154.04, C			
8	155.01, C			
9	118.86, CH	8.23, s		C-8, 11
11	173.86, C			
1'	34.38, CH_2	3.07, t (7.6)	H-2'	C-2', 3', 9, 11, 8
2'	28.97, CH_2	1.85, m	H-1', H-3'	C-3', 1', 4', 11
3'	39.43, CH_2	1.34, m	H-2', H-4'	C-5', 6', 2', 1', 4'
4'	29.17, CH	1.62, m	H-3', H-5', H-6'	C-5', 6', 2', 3'
5'	22.89, CH_3	0.93, d (6.6)	H-4'	C-6', 4', 3'
6'	22.89, CH_3	0.93, d (6.6)	H-4'	C-5', 4', 3'

Supplementary Table 14. NMR data of compound **32** (DMSO-d_6, δ in ppm).

No	δc	δ_H mult (*J* in Hz)	^{1}H-^{1}H COSY	HMBC
2	152.08, C			
3	165.66, C			
4	106.56, CH	7.48, d (2.4)	H-6	C-6, 3, 5
5	167.51, C			
6	108.08, CH	7.65, d (2.4)	H-4	C-4, 8, 5
7	152.61, C			
8	153.22, C			
9	118.95, CH	8.69, s		C-11, 8, 7
11	171.26, C			
13	55.76, CH_3	3.95, s		C-5
1′	32.73, CH_2	3.05, t (7.6)	H-2'	C-11, 8, 9, 3', 2'
2′	29.65, CH_2	1.75, m	H-3', H-1'	C-11, 3', 1', 4'
3′	26.18, CH_2	1.39, m	H-4', H-2'	C-5', 4', 2', 1',
4′	38.04, CH_2	1.22, m	H-5', H-3'	C-6', 7', 5', 2'
5′	27.33, CH	1.53, m	H-4', H-6', H-7'	C-6', 7', 3', 4'
6′	22.50, CH_3	0.85, d (6.6)	H-5'	C-7', 5', 4'
7′	22.50, CH_3	0.85, d (6.6)	H-5'	C-6', 5', 4'
NH-a		7.73, s	NH-b	C-3
NH-b		8.45, s	NH-a	C-2

Supplementary Table 15. NMR data of compound **33** (DMSO-d_6, δ in ppm).

No	δc	δ_H mult (*J* in Hz)	^{1}H-^{1}H COSY	HMBC
2	152.08, C			
3	165.65, C			
4	106.53, CH	7.48, d (2.3)	H-6	C-6, 3, 5
5	167.50, C			
6	108.09, CH	7.65, d (2.3)	H-4	C-4, 8, 5
7	152.60, C			
8	153.20, C			
9	118.97, CH	8.69, s		C-11, 8, 7
11	171.28, C			
13	55.76, CH_3	3.95, s		C-5
1′	32.93, CH_2	3.04, t (7.6)	H-2'	C-11, 8, 9,3', 2'
2′	27.31, CH_2	1.77, m	H-3', H-1'	C-11, 3', 1', 4'
3′	37.72, CH_2	1.28, m	H-4', H-2'	C-5', 6', 2', 4', 1'
4'	27.22, CH	1.59, m	H-5', H-6', H-3'	C-5', 6', 2', 3'
5′	22.45, CH_3	0.88, d (6.6)	H-4'	C-6', 4', 3'
6′	22.45, CH_3	0.88, d (6.6)	H-4'	C-5', 4', 3'
NH-a		7.73, s	NH-b	C-3
NH-b		8.45, s	NH-a	C-2

Supplementary Table 16. NMR data of compound **35** ($CDCl_3$ *δ* in ppm).

No	*δc*	δ_H mult (*J* in Hz)	^1H-^1H COSY	HMBC
2	129.53, C			
3	172.01, C			
4	148.75, C			
5	156.16, C			
6	108.12, CH	7.79, s		C-5, 8, 4
7	144.54, C			
8	154.03, C			
9	115.03, CH	7.75, s		C-8, 11, 7
11	172.04, C			
13	56.51, CH_3	4.05, s		C-5
1'	33.73, CH_2	3.05, t (7.8)	H-2'	C-11, 3', 2', 8
2'	29.94, CH_2	1.85, m	H-1', H-3'	C-11, 4', 3', 1'
3'	31.46, CH_2	1.44, m	H-2', H-4'	C-4',
4'	22.52, CH_2	1.40, m	H-3', H-5'	C-5', 2', 3'
5'	14.12, CH_2	0.93, t (7.1)	H-4'	C-4', 3'
NH-a		5.71, s	NH-b	
NH-b		7.96, s	NH-a	
OH-4		12.17, s		C-2, 4, 5

Supplementary Table 17. NMR data of compound **36** ($CDCl_3$ *δ* in ppm).

No	*δc*	δ_H mult (*J* in Hz)	^1H-^1H COSY	HMBC
2	129.55, C			
3	172.07, C			
4	148.78, C			
5	156.14, C			
6	108.05, CH	7.77, s		C-5, 8, 7, 4
7	144.37, C			
8	154.20, C			
9	115.26, CH	7.77, s		C-8, 11, 7
11	170.57, C			
13	56.48, CH_3	4.04, s		C-5
1'	32.67, CH_2	3.07, t (7.5)	H-2'	C-11, 3', 2', 9, 8
2'	23.85, CH_2	2.12, m	H-1', H-3'	C-11, 3', 1'
3'	42.48, CH_2	2.58, t (7.2)	H-2'	C-2', 1', 4
4'	208.15, C			
5'	30.23, CH_3	2.16, s		C-4', 3'
OH-4		12.19, s		C-2, 4, 5
NH-a		5.96, s	NH-b	
NH-b		7.97, s	NH-a	

Supplementary Table 18. NMR data of compound **37** ($CDCl_3$ δ in ppm).

No	*δc*	δ_H mult (*J* in Hz)	^1H-^1H COSY	HMBC
2	129.70, C			
3	172.18, C			
4	148.89, C			
5	156.15, C			
6	107.71, CH	7.67, s		C-4, 8, 5
7	144.08, C			
8	153.97, C			
9	115.08, CH	7.70, s		C-7, 8, 11
11	171.76, C			
13	56.48, CH_3	4.04, s		C-5
1'	33.17, CH_2	3.11, m	H-2'	C-11, 3', 2'
2'	25.33, CH_2	2.00, m	H-3', 1'	C-1', 3', 4', 11
3'	38.65, CH_2	1.62, m	H-4', 2'	C-5', 2', 1', 4'
4'	67.33, CH	3.90, m	H-5', 3'	C-5', 3'
5'	23.78, CH_3	1.22, d (6.2)	H-4'	C-3', 4'
NH-a		5.74, s	NH-b	
NH-b		8.33, s	NH-a	
OH-4		12.31, s		C-2, 4, 5

Supplementary Table 19. NMR data of compound **28** (Acetone-d_6, δ in ppm).

No	*δc*	δ_H mult (*J* in Hz)	^1H-^1H COSY	HMBC
2	124.92, C			
3	172.92, C			
4	149.16, C			
5	141.16, C			
6	148.87, C			
7	129.62, C			
8	154.21, C			
9	115.73, CH	8.36, s		C-7, 8, 11, 1'
11	172.89, C			
1'	33.40, CH_2	3.18, t (7.6)	H-2'	C-3', 2', 11, 8, 9
2'	30.70, CH_2	1.86, m	H-1', H-3'	C-3', 1', 4', 11
3'	27.45, CH_2	1.47, m	H-2', H-4'	C-2', 1', 4'
4'	39.31, CH_2	1.28, m	H-3', H-5'	C-6', 7', 3', 5', 2'
5'	28.62, CH	1.57, m	H-4', H-6, H-7	C-6', 7', 3', 4'
6'	22.91, CH_3	0.88, d (6.6)	H-5'	C-7', 5', 4'
7'	22.91, CH_3	0.88, d (6.6)	H-5'	C-6', 5', 4'
NH-a		7.19, s		
NH-b		12.12, s		
OH-4		12.63, s		C-2, 5, 4

Supplementary Table 20. NMR data of compound **30** ($CDCl_3$, δ in ppm).

No	δc	δ_H mult (*J* in Hz)	^1H-^1H COSY	HMBC
2	123.98, C			
3	171.69, C			
4	152.89, C			
5	142.34, C			
6	150.49, C			
7	129.99, C			
8	152.98, C			
9	113.92, CH	7.78, s		C-7, 8, 11
11	171.89, C			
13	60.75, CH_3	4.12, s		C-5
1'	33.17, CH_2	3.08, t (7.7)	H-2'	C-3', 2', 11
2'	29.91, CH_2	1.25, m	H-3'	C-3'
		1.84, m	H-3', H-1'	C-3', 1', 4', 11
3'	26.89, CH_2	1.42, m	H-2', H-4'	C-5', 2', 1', 4'
4'	38.65, CH_2	1.23, s	H-3', H-5'	C-6', 7', 2', 5'
5'	27.96, CH	1.55, m	H-4', H-6', H-7'	C-6, 7',4'
6'	22.71, CH_3	0.88, d (6.6)	H-5'	C-7', 5', 4'
7'	22.71, CH_3	0.88, d (6.6)	H-5'	C-6', 5', 4'
NH-a		5.56, s	NH-b	
NH-b		7.63, s	NH-a	
OH-4		12.12, s		C-2, 5, 4
OH-6		12.55, s		C-7, 5, 6

Supplementary Table 21. Strains and plasmids used and generated in this study.

**Strains/plasmids**	**Purpose**	**Sources**
E. coli		
ET12567/pUZ8002	Donor strain for conjugation	Ref. 7
BW25113/pIJ790	Host strain for PCR targeting	Ref. 8
DH5α/BT340	Host strain for in-frame deletion	Ref. 9
XL1-Blue	Construction of gene library	Agilent
DH5α	Host strain for cloning	Invitrogen
BL21(DE3)	Heterologous host for protein expression	NEB
**strain**		
NEAU-A2	Knrnamicins wild type producing strain	This study
NEAU-A2-Δ*knmE*	*knmE* inactivation mutant of *L. rhizosphaerae* NEAU-A2	This study
NEAU-A2-Δ*knmA1*	*knmA1* inactivation mutant of *L. rhizosphaerae* NEAU-A2	This study
NEAU-A2-Δ*knmA2*	*knmA2* inactivation mutant of *L. rhizosphaerae* NEAU-A2	This study
NEAU-A2-Δ*knmB1*	*knmB1* inactivation mutant of *L. rhizosphaerae* NEAU-A2	This study
NEAU-A2-Δ*knmB2*	*knmB2* inactivation mutant of *L. rhizosphaerae* NEAU-A2	This study
NEAU-A2-Δ*knmC*	*knmC* inactivation mutant of *L. rhizosphaerae* NEAU-A2	This study
NEAU-A2-Δ*knmF*	*knmF* inactivation mutant of *L. rhizosphaerae* NEAU-A2	This study
S. albus J1074	Host strain for heterologous expression	Ref. 10
*S. albus*5C1	*S. albus* J1074 integrated with plasmid 5C1 which contains *knm* BGCs	This study
*S. albus*5C1-Δ*knmE*	*knmE* inactivation mutant of *S. albus* 5C1	This study
*S. albus*5C1-Δ*knmB1*	*knmB1* inactivation mutant of *S. albus* 5C1	This study
*S. albus*5C1-Δ*knmB2*	*knmB2* inactivation mutant of *S. albus* 5C1	This study
*S. albus*5C1-Δ*knmC*	*knmC* inactivation mutant of *S. albus* 5C1	This study
*S. albus*5C1-Δ*knmF*	*knmF* inactivation mutant of *S. albus* 5C1	This study
*S. albus-5C1-*Δ*knmC/knmC*	*knmC* complement strain	This study
**Plasmids**		
pSuperCos I	Kan^r, Cosmid vector for genomic library	Stratagene

	construction	
pJTU2554	Apr^r, Cosmid vector for genomic library construction	Ref. 8
pJTU6722	Ery^r, Vector for PCR targeting	Ref. 11
pIJ773	Apr^r, Vector for PCR targeting	Ref. 12
p5C1	Apr^r, Cosmid which contains *knm* biosynthetic gene cluster	This study
pS4H1	A cosmid which contains partial *knm* biosynthetic gene cluster	This study
pS20G3	A cosmid which contains partial *knm* biosynthetic gene cluster	This study
pS12G3	A cosmid which contains partial *knm* biosynthetic gene cluster	This study
p5C1-Δ*knmE*	Apr^r, gene inactivation clone used for Δ*knmE* mutant	This study
p5C1-Δ*knmB1*	Apr^r, gene inactivation clone used for Δ*knmB1* mutant	This study
p5C1-Δ*knmB2*	Apr^r, gene inactivation clone used for Δ*knmB2* mutant	This study
p5C1-Δk*nmC*	Apr^r, gene inactivation clone used for Δ*knmC* mutant	This study
p5C1-Δ*knmF*	Apr^r, gene inactivation clone used for Δ*knmF* mutant	This study
pET28a	Kan^r, Protein expression vector used in *E. coli*, encoding N-terminal His-tag	Novagen
pET32a	Amp^r, Protein expression vector used in *E. coli*, encoding N-terminal a thioredoxin (Trx)-tag (109 residues), 6X His-tag (6 residues), a thrombin site (6 residues), a S-tag (15 residues) and an enterokinase site	Novagen
pGro7	Cml^r, Protein coexpression vector used to increase recovery of target proteins in the soluble fraction in *E. coli*	Takara

Supplementary Table 22. Primers used in this study.

Primer	Sequence (5' to 3')
**For PCR targeting**	
ΔKnmE-F	cagcgtccgcgacctcggcggctacggcgtgcacctcgcATTCCGGGGATCCGTCGACC
ΔKnmE-R	gatgttcctcggccagccgtgccaccttggaccgccagtTGTAGGCTGGAGCTGCTTC
ΔKnmB1-F	gcccggcggcctgcgcatgatcaccctgacccgccaggaATTCCGGGGATCCGTCGACC
ΔKnmB1-R	ggtgcccgcgccctggcccagcgtcggcggcatggcatgTGTAGGCTGGAGCTGCTTC
ΔKnmB2-F	gcgagttctaccagcgccacggcaccccgttcgtcgtcaATTCCGGGGATCCGTCGACC
ΔKnmB2-R	tgtgccgcgtccccgagcaacgtgatccgccccttgctcTGTAGGCTGGAGCTGCTTC
ΔKnmC-F	tcaacggagagatctacaacttccgtgaactgcgcaacgATTCCGGGGATCCGTCGACC
ΔKnmC-R	cgccaccgtcgagcgcatcttgacttcccggaacagcagTGTAGGCTGGAGCTGCTTC
ΔKnmF-F	actgctcgagtcggaaggcgagtacttccggaactccgcATTCCGGGGATCCGTCGACC
ΔKnmF-R	ctcaggaccccagttgtgcagcaccgtcgacagcagtgcTGTAGGCTGGAGCTGCTTC
ΔKnmA1-F	ttcctcgcccgcttcggcgactccgactgagaggcagtgATTCCGGGGATCCGTCGACC
ΔKnmA1-R	gaactcccggacggtgtcccgccactcctgctcagtcacTGTAGGCTGGAGCTGCTTC
ΔKnmA2-F	ctcctgaaaaccccgacgcaccacggaggactaccagtgATTCCGGGGATCCGTCGACC
ΔKnmA2-R	gtacaggtcctcgatgttcttcgccatcactgcctctcaTGTAGGCTGGAGCTGCTTC
**For gene validation**	
KnmE-check-F	gcgcaagccacactgacgat
KnmE-check-R	tggaccgaggatggggaagc
KnmA1-check-F	cttcggcgactccgactgag
KnmA1-check-R	gacggtgtcccgccactcct
KnmB2-check-F	gagatgctcgtccgtgttga
KnmB2-check-R	gatctcgacgtgtcgtctca
KnmC-check-F	cgattccacgacggggagga
KnmC-check-R	catcagctacccgtgaccac
KnmF-check-F	caaggggaaatggcgtggtt
KnmF-check-R	tcgtcgtcacgaagggtcgc
KnmA2-check-F	tgaaaaccccgacgcaccac

KnmA2-check-R	tgttcttcgccatcactgcc
KnmB1-check-F	gacaggtcacggaggcgcggt
KnmB1-check-R	gacgaaggcggcgttccacc
**Protein expression**	
KnmF-ND-F-28a	GTGCCGCGCGGCAGCCATATGgtggtcaccccgatgccgct
KnmF-SA-R-28a	TGCGGCCGCAAGCTTGTCGACtcagtgcggcttgcgtccca
KnmB2-BM-F-32a	CGCGGATCCatgaacgttgcggtgatcggtg
KnmB2-HD-R-32a	CCCAAGCTTtcacgaccgcgcctccgtga
KnmB1-BM-F-32a	CGCGGATCCgtgagacgacacgtcgagatcgcgg
KnmB1-HD-R-32a	CCCAAGCTTtcaaccgcgtggcgagcgc

Supplementary Table 23. Primers used for constructing KnmB1 and KnmB2 protein variants.

Primer	Sequence (5' to 3')
KnmB1-N219D1-F	gccatggctgatatcggatccgtgagacgacacgtcgagatc
KnmB1-N219D1-R	accgtcgggggcaccgaggtcgaggtacaggtccgtggagt
KnmB1-N219D2-F	gacctcggtgcccccgacggt
KnmB1-N219D2-R	ctcgagtgcggccgcaagctttcaaccgcgtggcgagcgcgccg
KnmB1-R205A1-F	gccatggctgatatcggatccgtgagacgacacgtcgagatcg
KnmB1-R205A1-R	ttgcacggcacgtacagcaccgcccgccggagctccacgttcca
KnmB1-R205A2-F	gcggtgctgtacgtgccgtgcaa
KnmB1-R205A2-R	ctcgagtgcggccgcaagctttcaaccgcgtggcgagcgcgccg
KnmB1-Y217A1-F	gccatggctgatatcggatccgtgagacgacacgtcgagatcg
KnmB1-Y217A1-R	ggggcaccgaggttgagtgccaggtccgtggagttgcacg
KnmB1-Y217A2-F	gcactcaacctcggtgcccccga
KnmB1-Y217A2-R	ctcgagtgcggccgcaagctttcaaccgcgtggcgagcgcg
KnmB1-Y217F1-F	gccatggctgatatcggatccgtgagacgacacgtcgagatcgcg
KnmB1-Y217F1-R	tcgggggcaccgaggttgaggaacaggtccgtggagttgcacg
KnmB1-Y217F2-F	ttcctcaacctcggtgcccccga
KnmB1-Y217F2-R	ctcgagtgcggccgcaagctttcaaccgcgtggcgagcgcgccg
KnmB1-Y262A1-F	gccatggctgatatcggatccgtgagacgacacgtcgagatcg
KnmB1-Y262A1-R	cgcagccgcagcacctctgcccggtcgaacctgggcgccg
KnmB1-Y262A2-F	gcagaggtgctgcggctgcgca
KnmB1-Y262A2-R	ctcgagtgcggccgcaagctttcaaccgcgtggcgagcgcg
KnmB2-F205A1-F	gccatggctgatatcggatccatgaacgttgcggtgatcgg
KnmB2-F205A1-R	cgggccgagcggcgcgaaggccatgccgagaccagggccga
KnmB2-F205A2-F	gccttcgcgccgctcggcccgg
KnmB2-F205A2-R	ctcgagtgcggccgcaagctttcacgaccgcgcctccgtga
KnmB2-N291D1-F	gccatggctgatatcggatccatgaacgttgcggtgatcgg
KnmB2-N291D1-R	cgcgccctggccgaggaagtcggtcatggcgtgtgccgcgt
KnmB2-N291D2-F	gacttcctcggccagggcgcg
KnmB2-N291D2-R	ctcgagtgcggccgcaagctttcacgaccgcgcctccgtga
KnmB2-Y215A1-F	gccatggctgatatcggatccatgaacgttgcggtgatcgg
KnmB2-Y215A1-R	atgatcgtggcggtccatgccatctcggccgggccgagcg
KnmB2-Y215A2-F	gcatggaccgccacgatcatctc
KnmB2-Y215A2-R	ctcgagtgcggccgcaagctttcacgaccgcgcctccgtga
KnmB2-Y215F1-F	gccatggctgatatcggatccatgaacgttgcggtgatcggtg
KnmB2-Y215F1-R	gagatgatcgtggcggtccagaacatctcggccgggccgagcg
KnmB2-Y215F2-F	ttctggaccgccacgatcatctc
KnmB2-Y215F2-R	ctcgagtgcggccgcaagctttcacgaccgcgcctccgtga

Supplementary Table 24. NMR data of compound **29** (DMSO-d_6, δ in ppm).

No	δc	δ_H mult (J in Hz)	^1H-^1H COSY	HMBC
2	123.79, C			
3	171.91, C			
4	148.03, C			
5	140.11, C			
6	144.49, C			
7	128.33, C			
8	152.57, C			
9	115.81, CH	8.58, overlapped		C-7, 8, 11
11	171.55, C			
1'	32.01, CH_2	3.11, t (7.5)	H-2'	C-2', 3', 9, 8, 11
2'	28.70, CH_2	1.78, m	H-1', H-3'	C-4', 3', 1', 11
3'	30.50, CH_2	1.35, overlapped	H-4', H-2'	C-4', 2', 5'
4'	21.80, CH_2	1.35, overlapped	H-5', H-4'	C-5', 3', 2'
5'	13.84, CH_3	0.87, t (6.9)	H-4'	C-4', 3'
NH-a		8.06, s	NH-b	C-2
NH-b		8.58, overlapped	NH-a	C-3
OH-4		12.79, s		C-2, 5, 4
OH-6		12.02, s		

Supplementary Table 25. NMR data of compound **31** (DMSO-d_6, δ in ppm).

No	δc	δ_H mult (J in Hz)	^1H-^1H COSY	HMBC
2	124.13, C			
3	171.76, C			
4	152.12, C			
5	141.41, C			
6	149.25, C			
7	129.19, C			
8	152.21, C			
9	116.21, CH	8.64, overlapped		C-8, 11
11	171.66, C			
13	59.84, CH_3	3.91, s		C-5
1'	31.99, CH_2	3.12, t (7.5)	H-2'	C-2', 3', 11
2'	28.76, CH_2	1.78, m	H-1', H-3'	C-4', 3', 1', 11
3'	30.51, CH_2	1.35, overlapped	H-4', H-2'	C-4', 2', 5'
4'	21.77, CH_2	1.35, overlapped	H-5', H-4'	C-5', 3', 2'
5'	13.85, CH_3	0.88, t (7.0)	H-4'	C-4', 3'
NH-a		8.12, s	NH-b	
NH-b		8.64, overlapped	NH-a	C-3
OH-4		12.33, s		
OH-6		13.07, s		

Supplementary Table 26. NMR data of compound **34** (DMSO-d_6, δ in ppm).

No	δc	δ_H mult (J in Hz)	^1H-^1H COSY	HMBC
2	152.09			
3	165.70			
4	106.58	7.48, d (2.4)	H-6	C-3, 5, 6
5	167.53			
6	108.13	7.65, d (2.4)	H-4	C-4, 5, 8
7	152.63			
8	153.23			
9	118.97	8.68, s		C-7, 8, 11
11	171.34			
13	55.79	3.95, s,		C-5
1'	32.72	3.05, t (7.7)	H-2'	C-2', 3', 9, 11, 8
2'	29.15	1.77, m	H-3', H-1'	C-3', 1', 4', 11
3'	30.68	1.36, overlapped	H-4', H-2'	C-5', 2', 1', 4'
4'	21.83	1.36, overlapped	H-5', H-3'	C-2', 3’, 5'
5'	13.90	0.88, t (7.1)	H-4'	C-3', 4'
NH-a		7.73, s		C-2
NH-b		8.45, s		C-3

Supplementary Figures

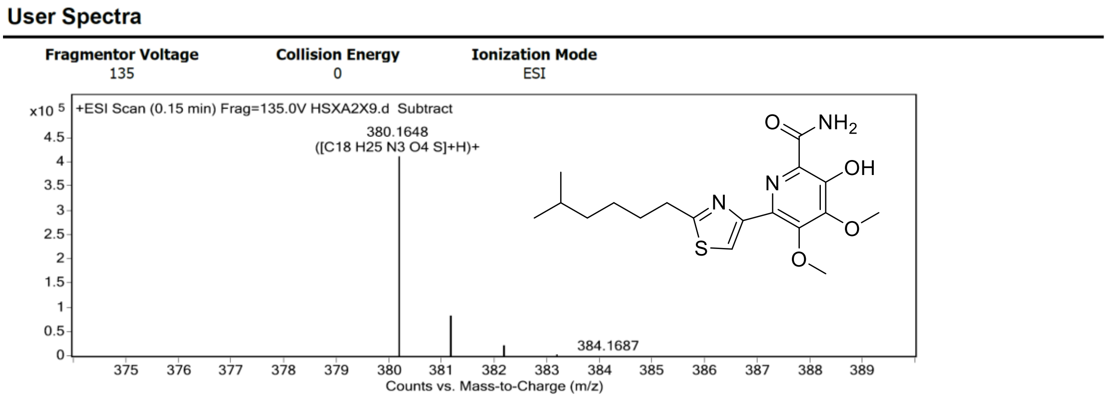

Supplementary Figure 1. HRESIMS spectrum of compound **1**.

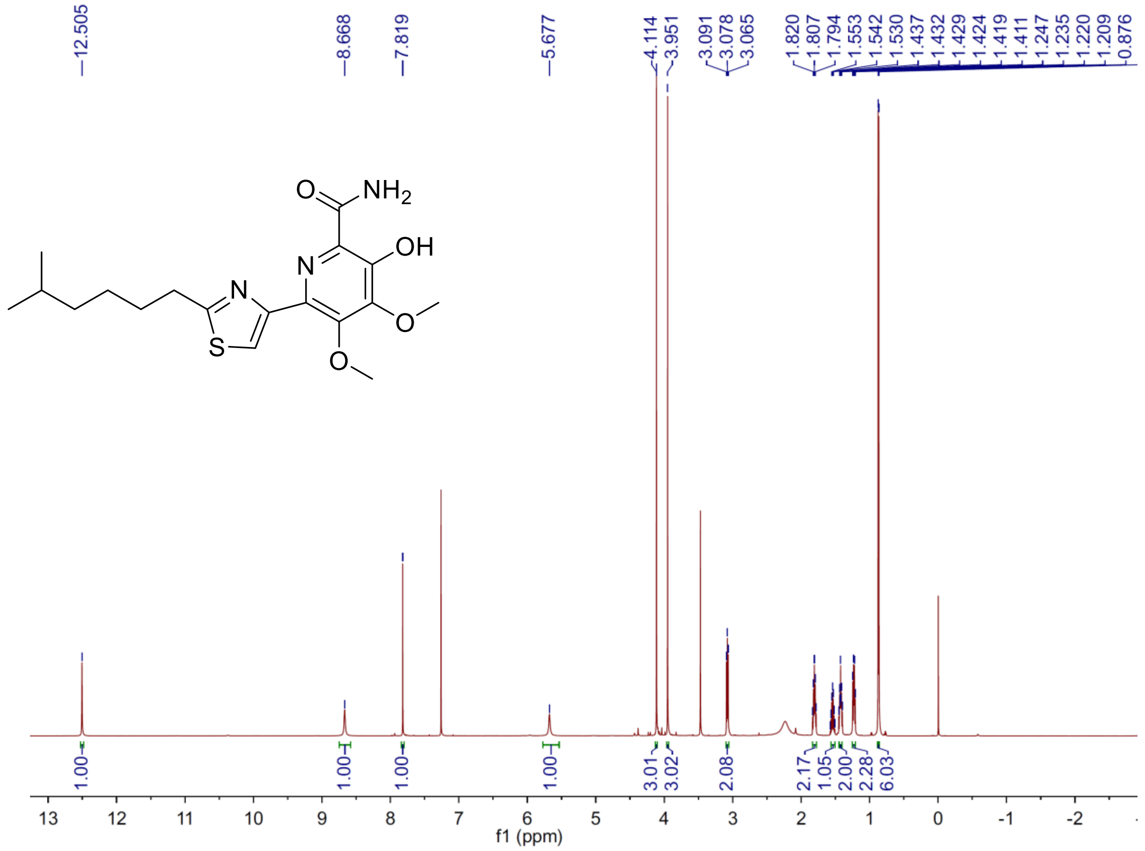

Supplementary Figure 2. ^{1}H-NMR (600 MHz) spectrum of compound **1** in $CDCl_3$.

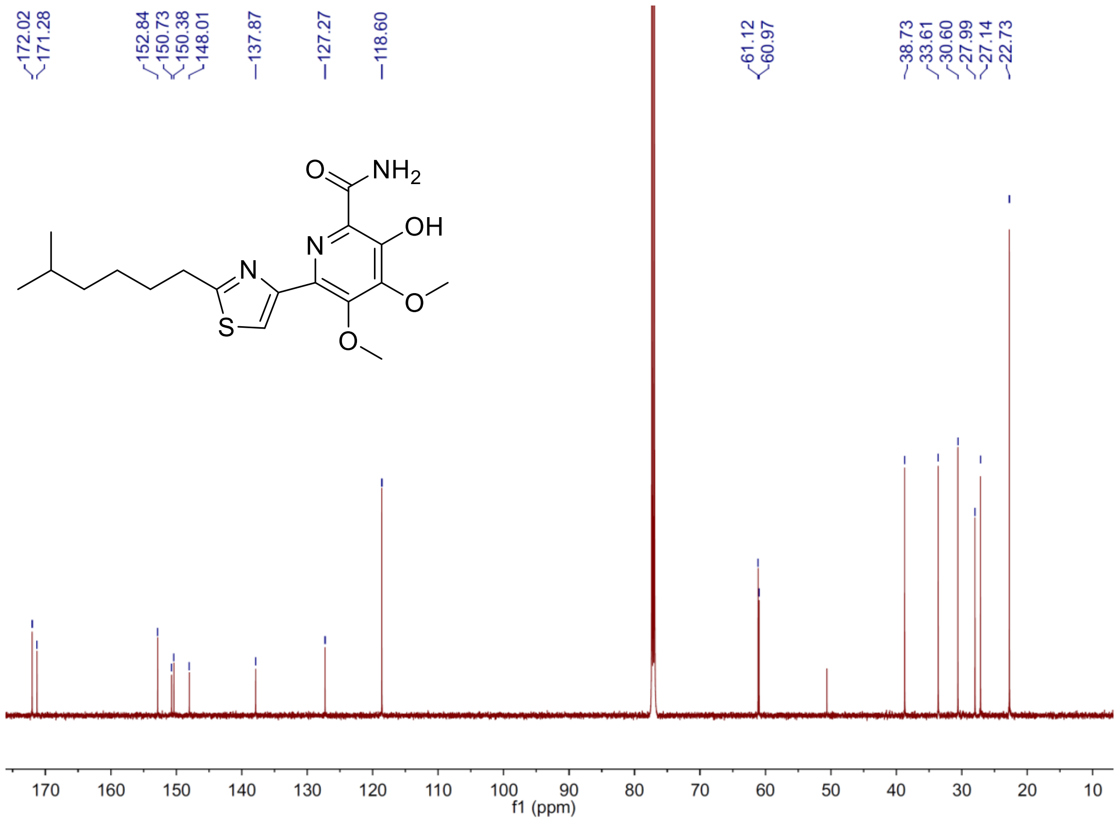

Supplementary Figure 3. ^{13}C-NMR (150 MHz) spectrum of compound **1** in $CDCl_3$.

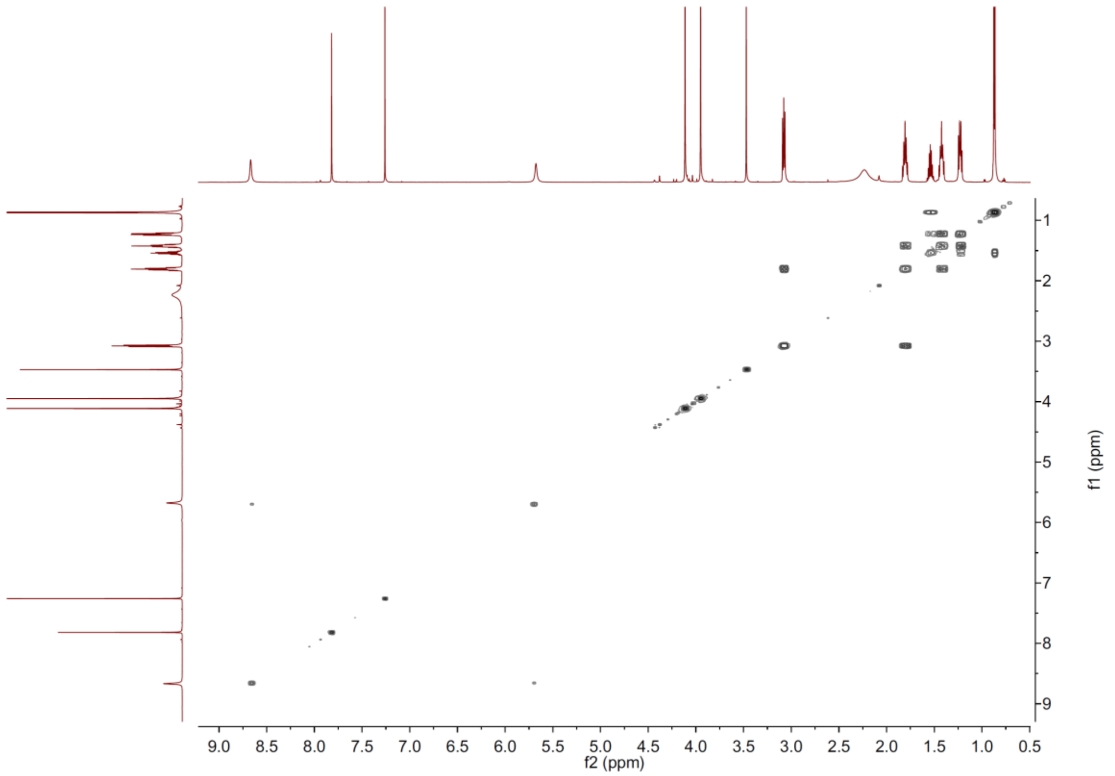

Supplementary Figure 4. ^{1}H-^{1}H COSY (600 MHz) spectrum of compound **1** in $CDCl_3$.

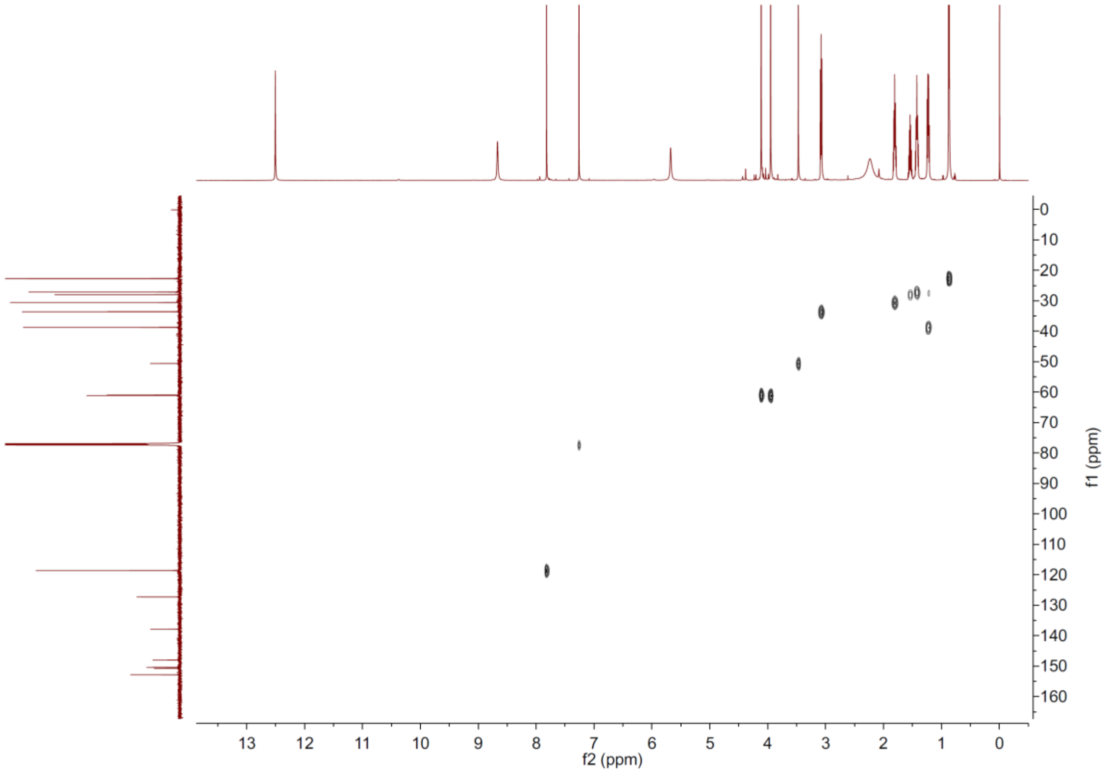

Supplementary Figure 5. HSQC (600 MHz) spectrum of compound **1** in $CDCl_3$.

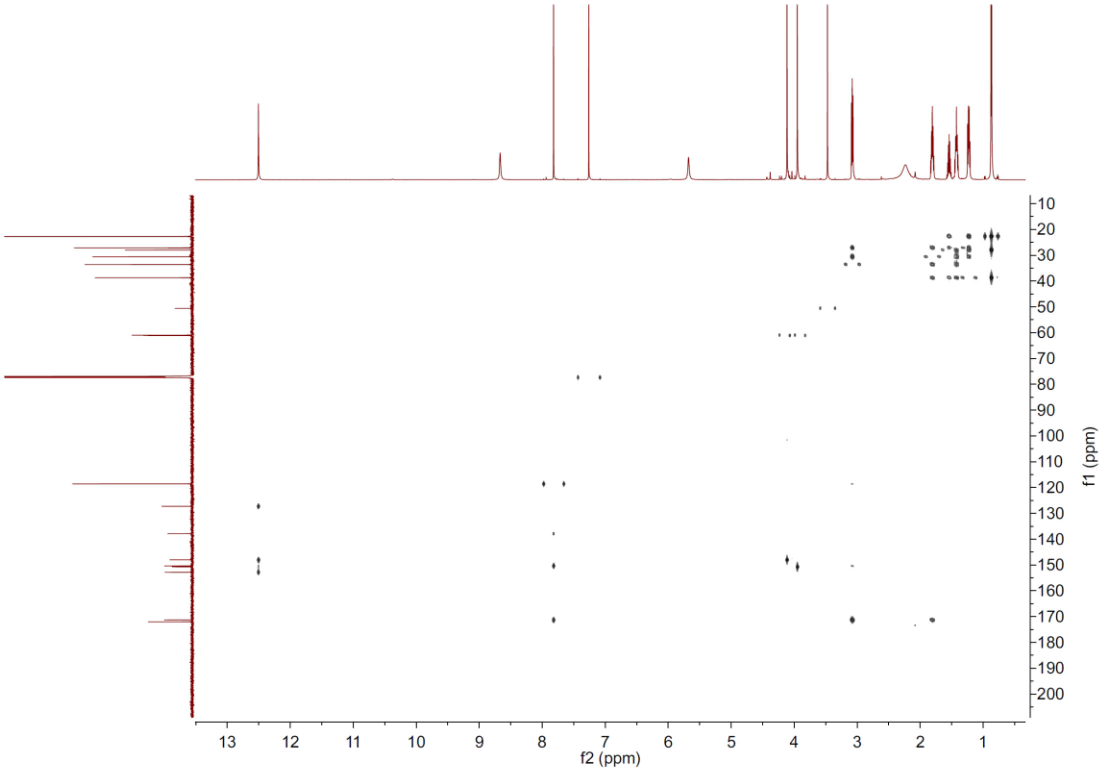

Supplementary Figure 6. HMBC (600 MHz) spectrum of compound **1** in $CDCl_3$.

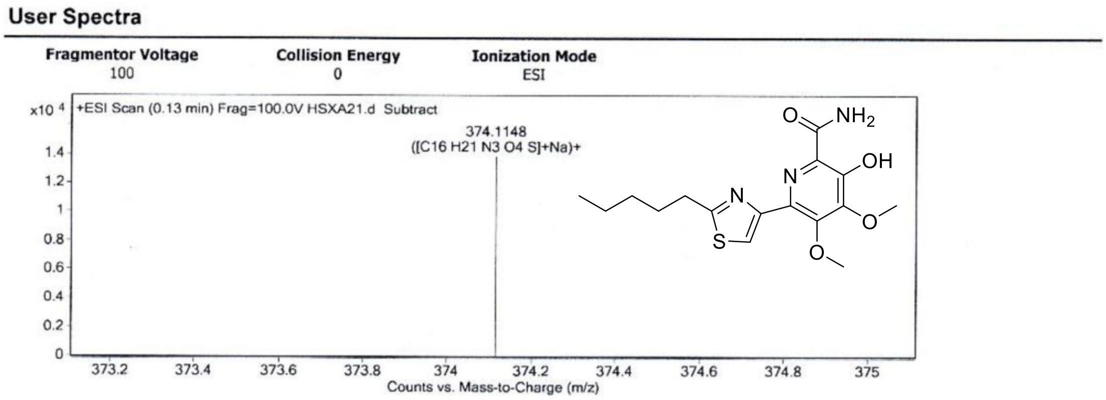

Supplementary Figure 7. HRESIMS spectrum of compound **2**.

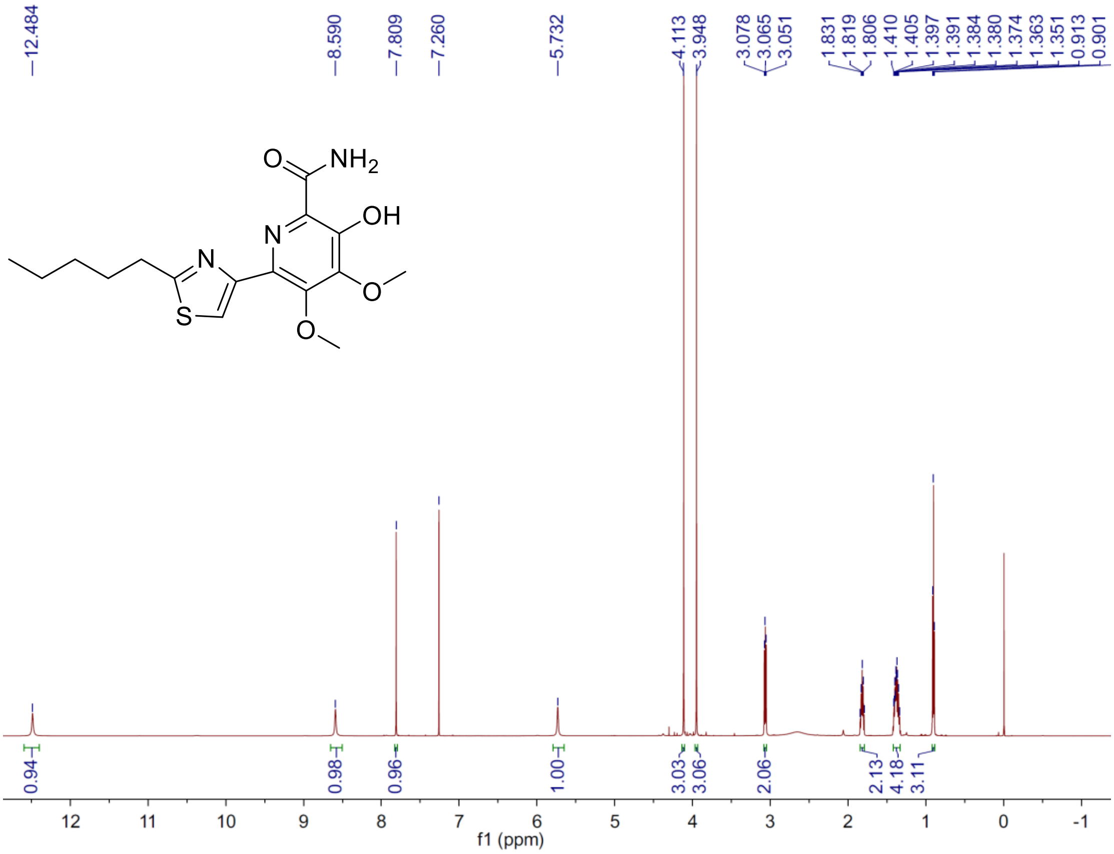

Supplementary Figure 8. ^{1}H NMR (600 MHz) spectrum of compound **2** in $CDCl_3$.

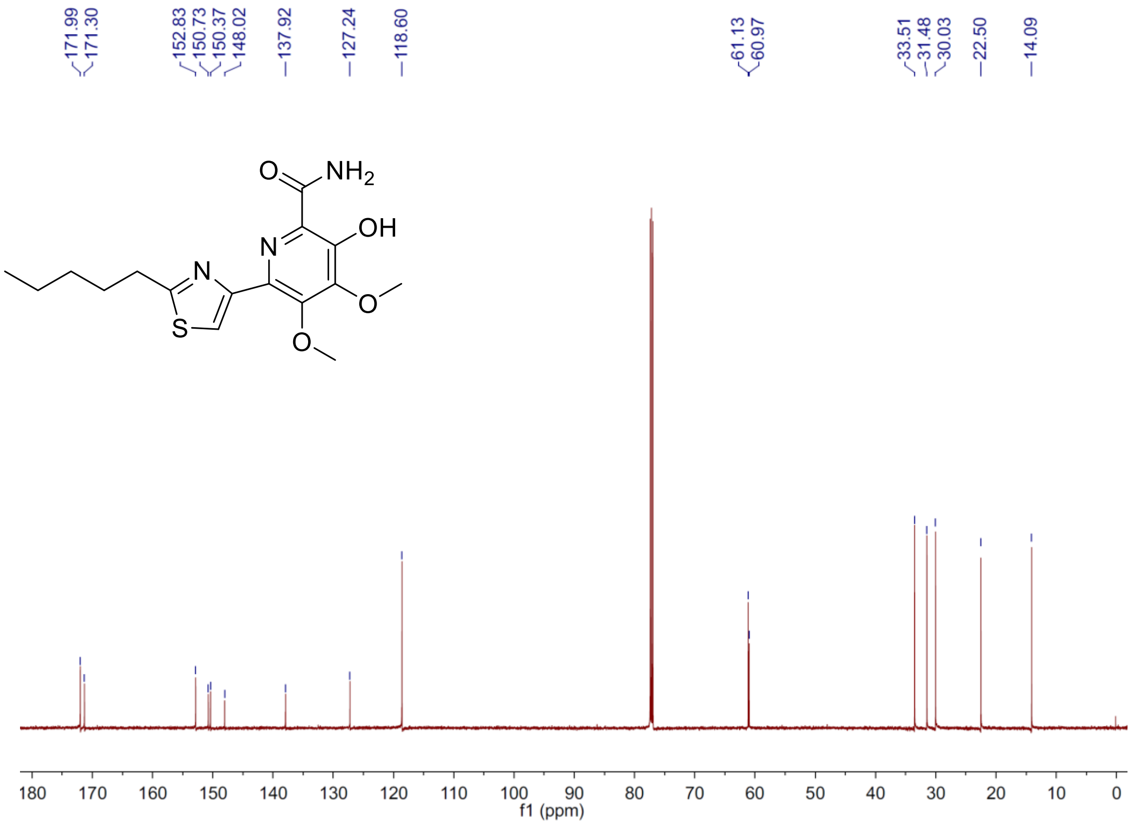

Supplementary Figure 9. ^{13}C NMR (150 MHz) spectrum of compound **2** in $CDCl_3$.

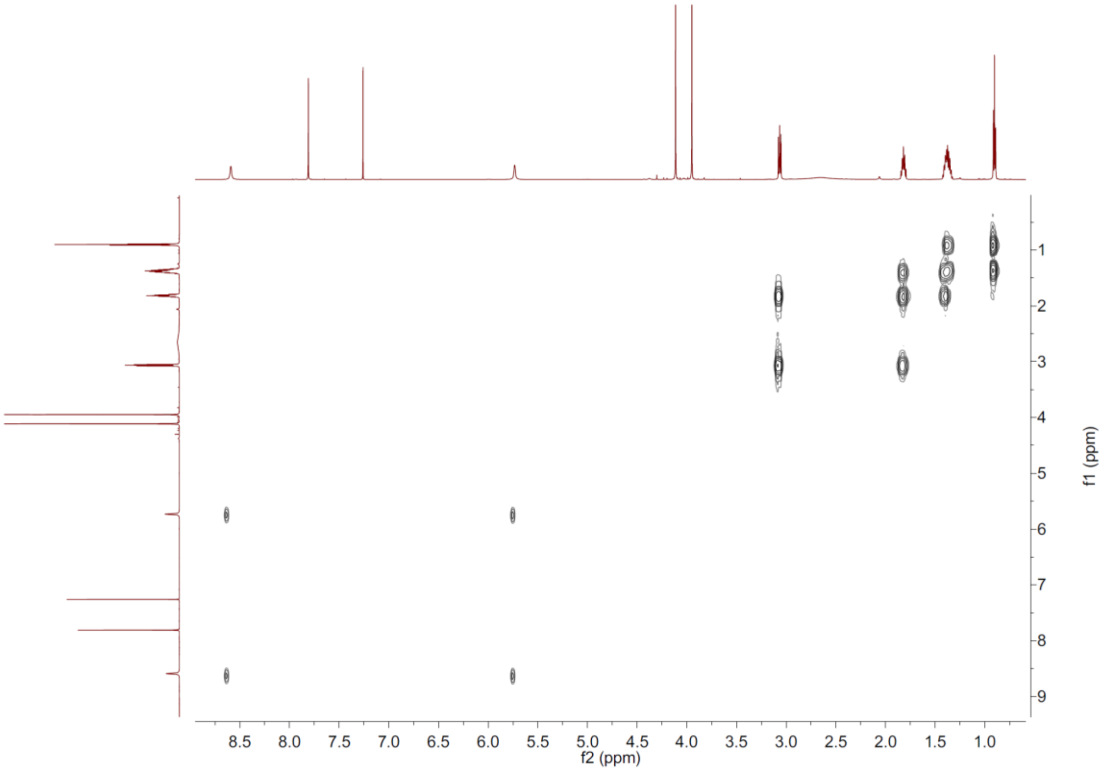

Supplementary Figure 10. ^{1}H-^{1}H COSY (600 MHz) spectrum of compound **2** in $CDCl_3$.

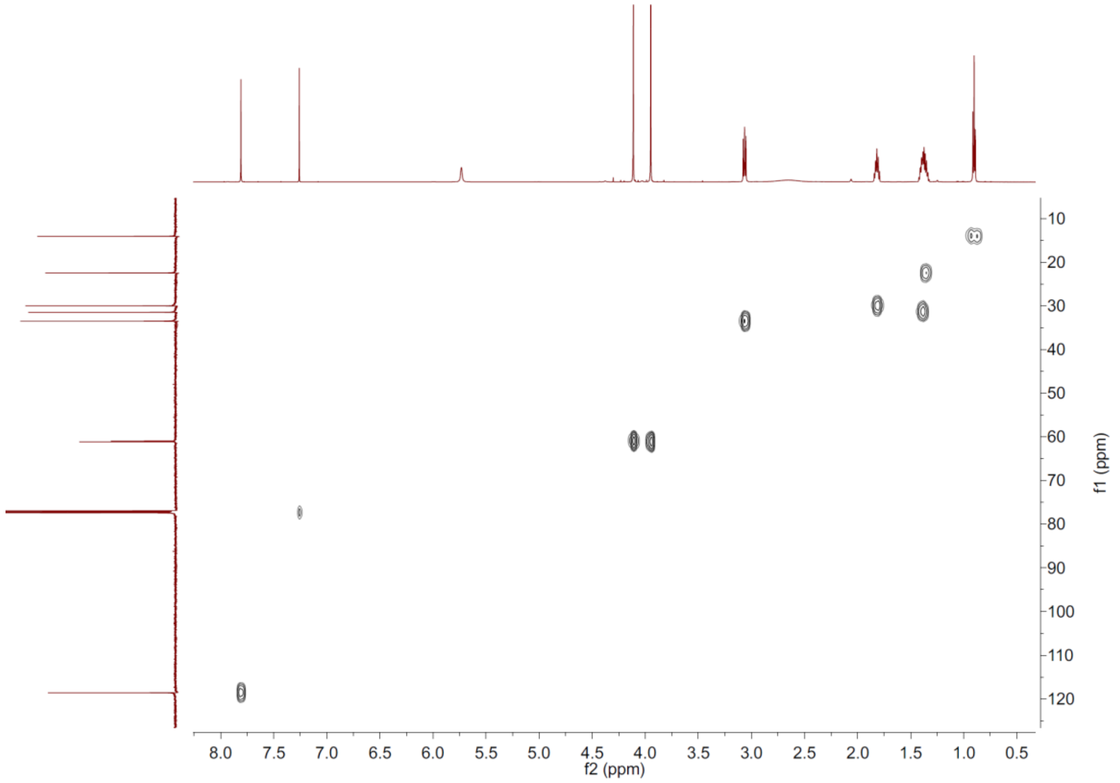

Supplementary Figure 11. HSQC (600 MHz) spectrum of compound **2** in $CDCl_3$.

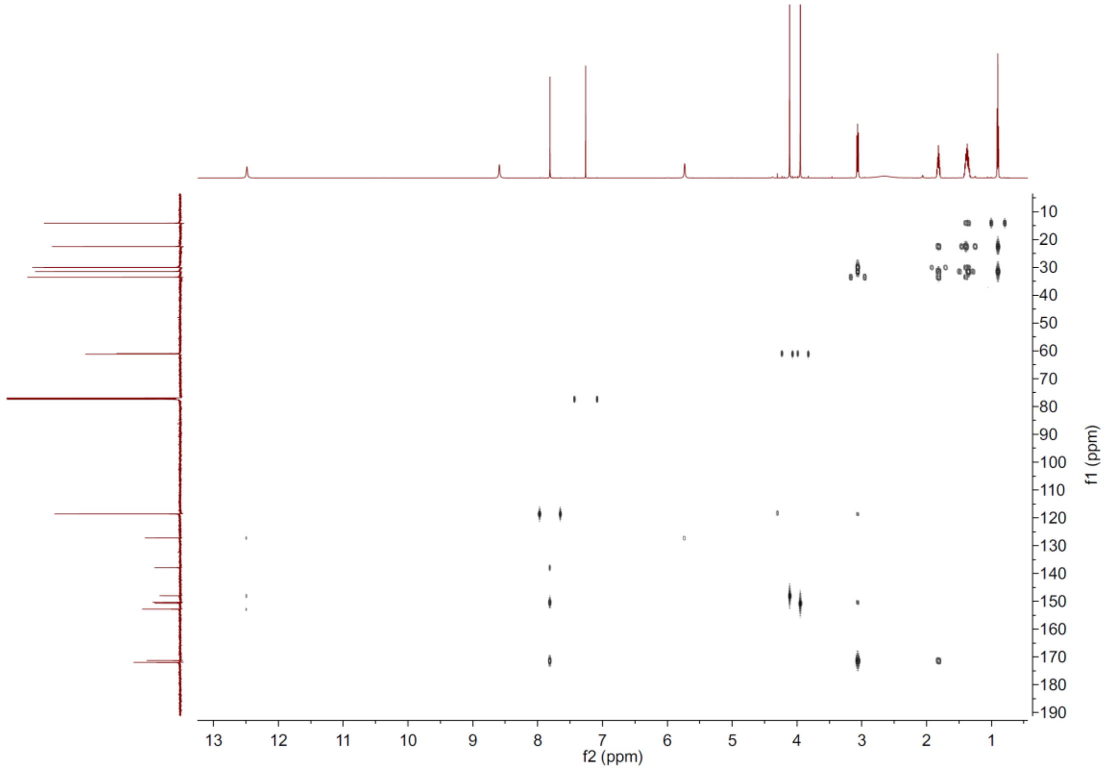

Supplementary Figure 12. HMBC (600 MHz) spectrum of compound **2** in $CDCl_3$.

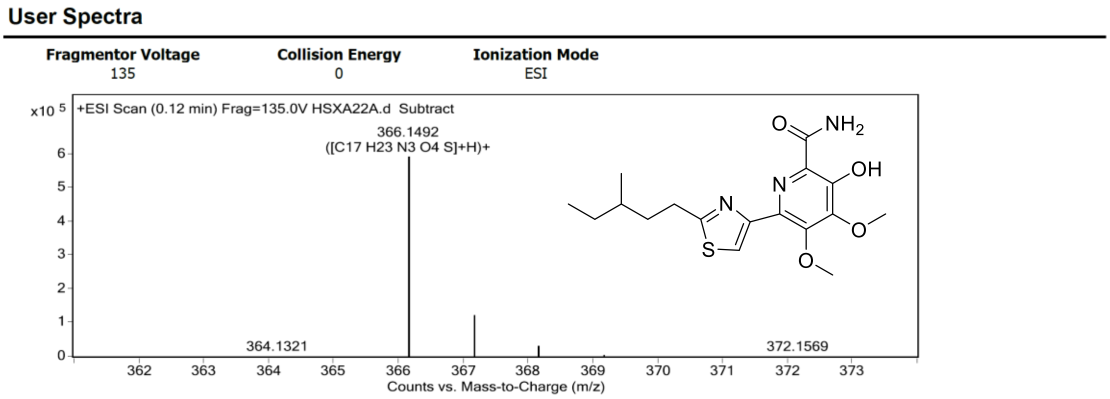

Supplementary Figure 13. HRESIMS spectrum of compound **3**.

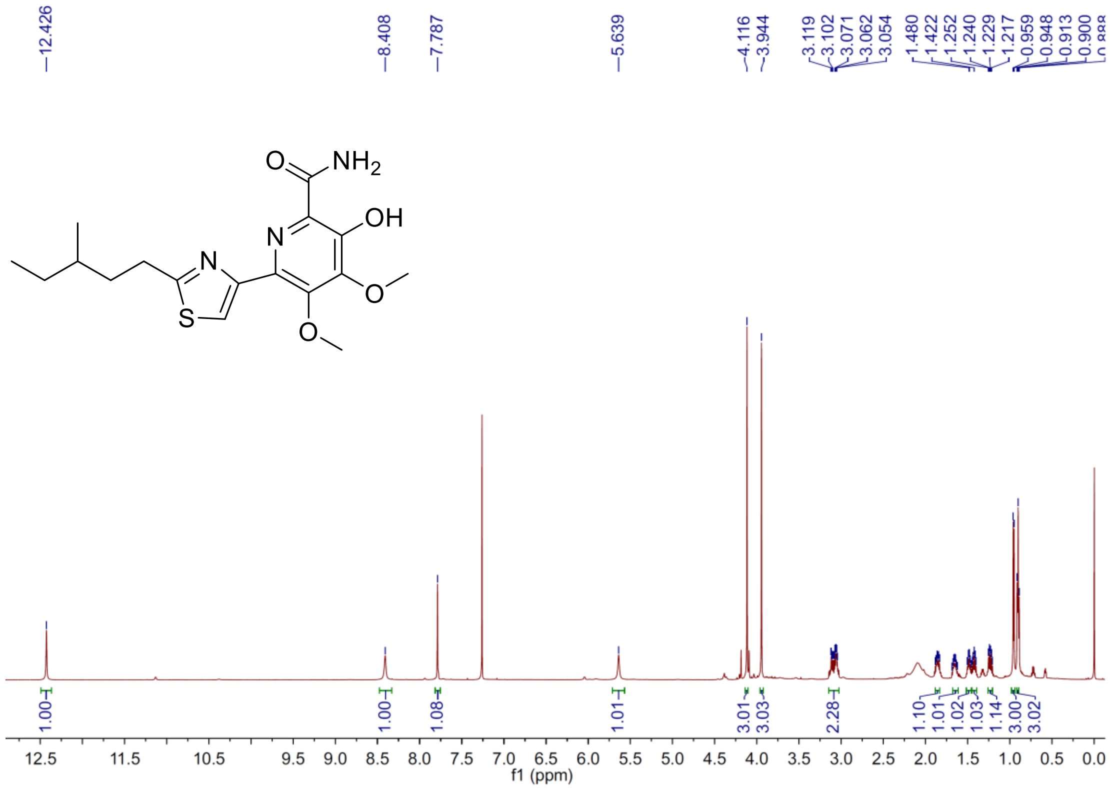

Supplementary Figure 14. ^{1}H-NMR (600 MHz) spectrum of compound **3** in $CDCl_3$.

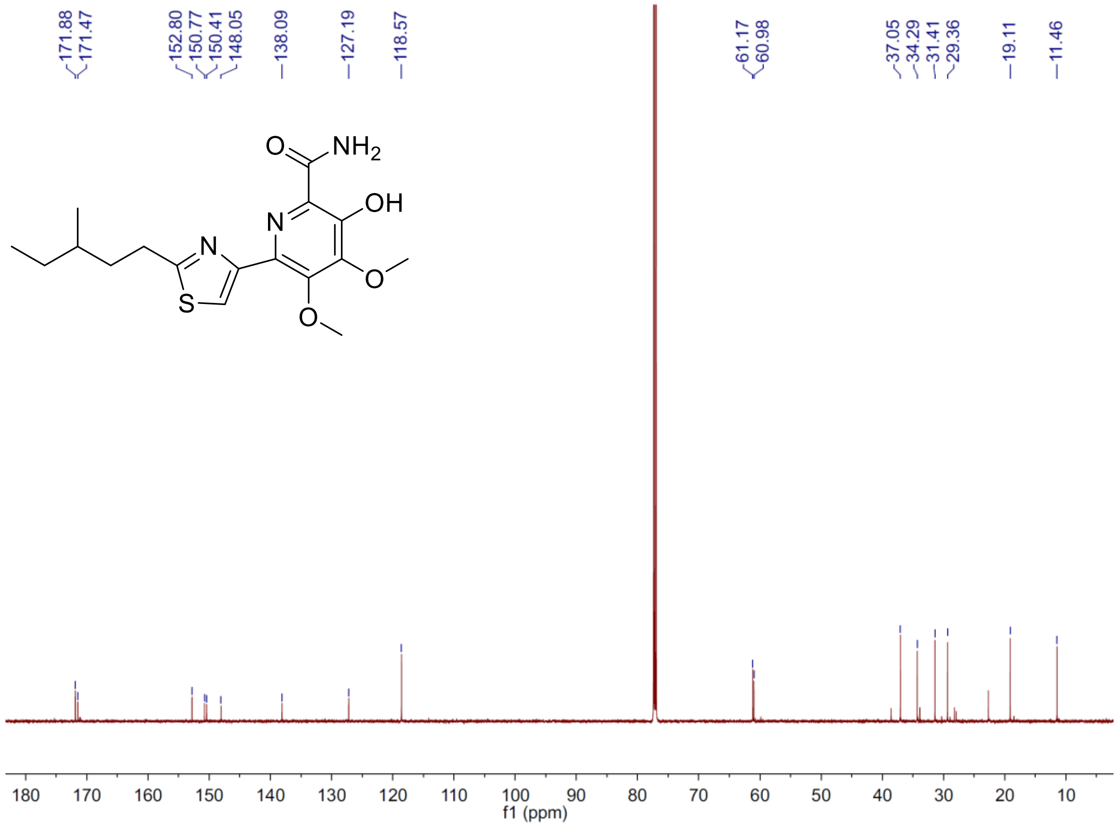

Supplementary Figure 15. ^{13}C -NMR (150 MHz) spectrum of compound **3** in $CDCl_3$.

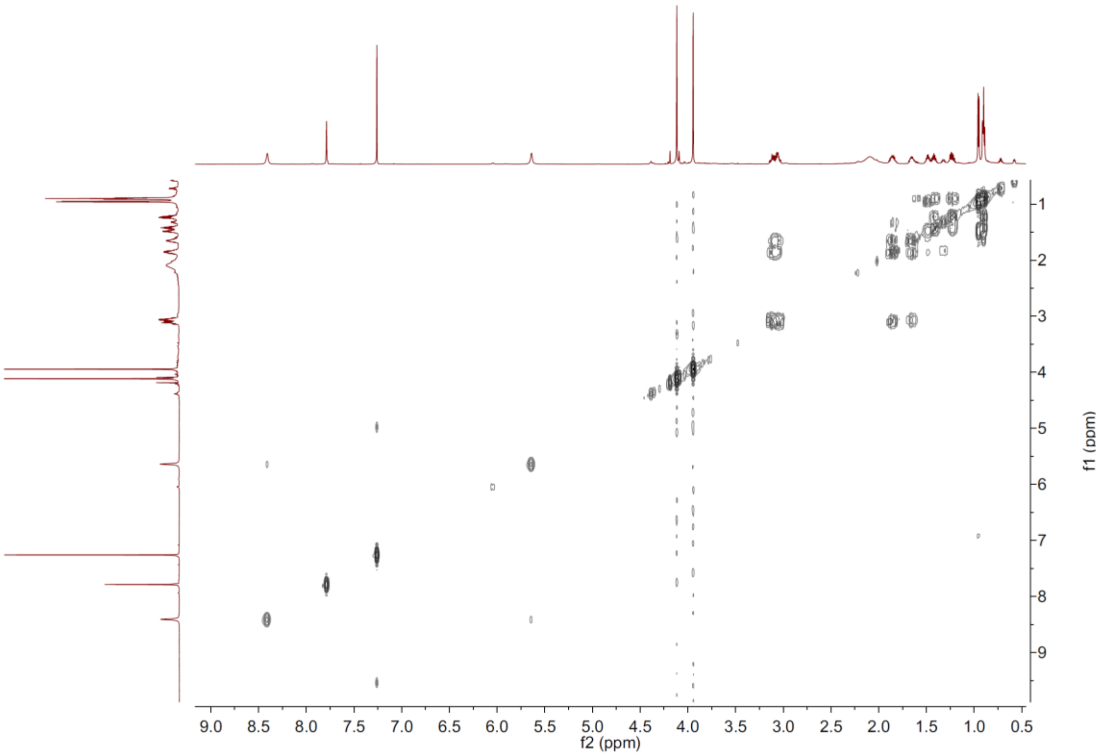

Supplementary Figure 16. ^{1}H-^{1}H COSY (600 MHz) spectrum of compound **3** in $CDCl_3$.

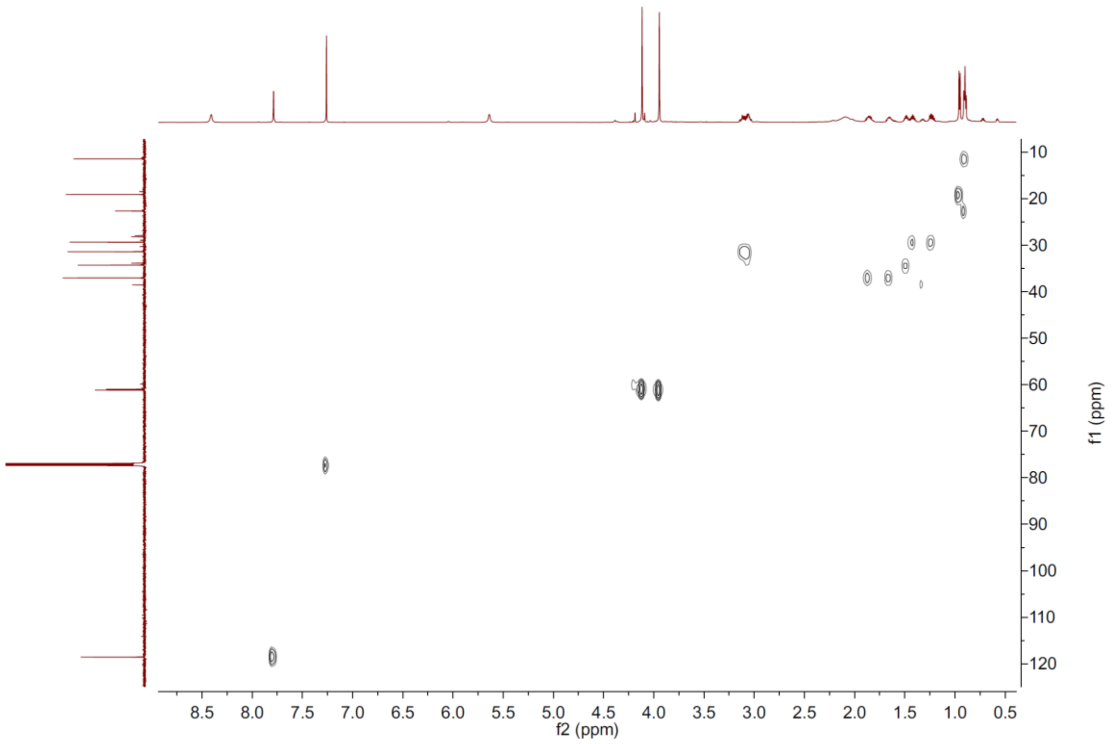

Supplementary Figure 17. HSQC (600 MHz) spectrum of compound **3** in $CDCl_3$.

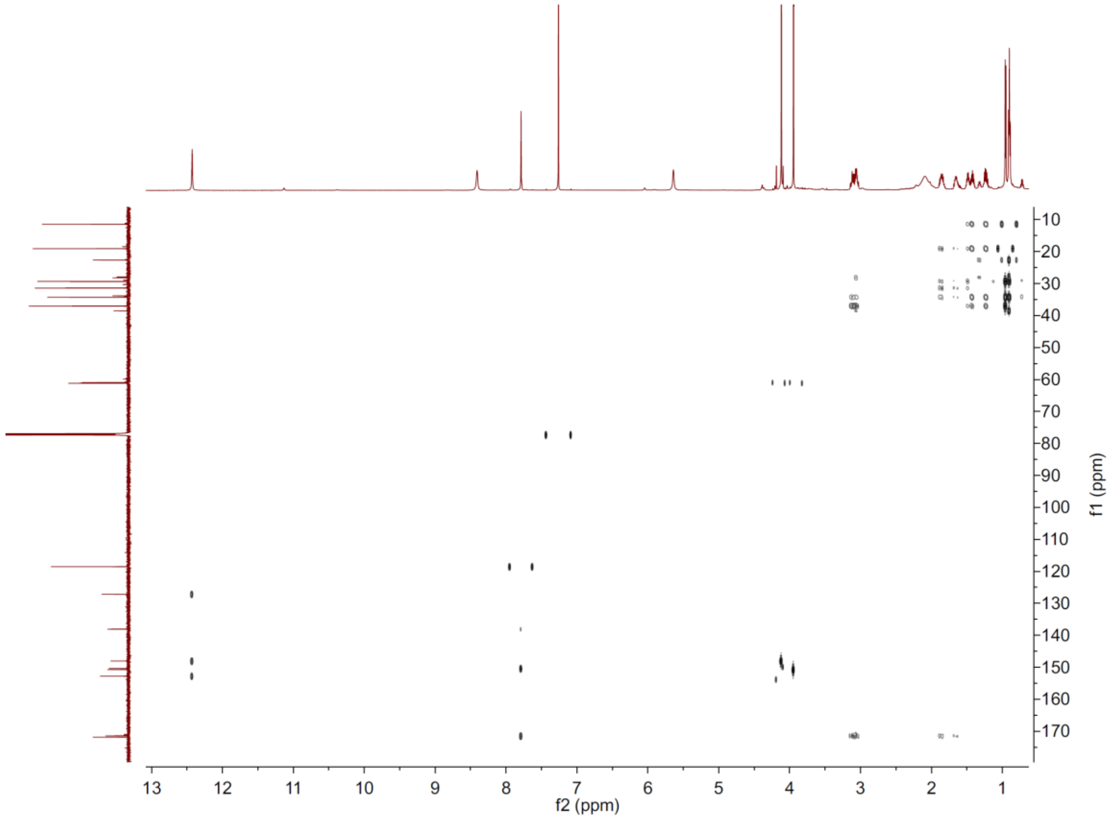

Supplementary Figure 18. HMBC (600 MHz) spectrum of compound **3** in $CDCl_3$.

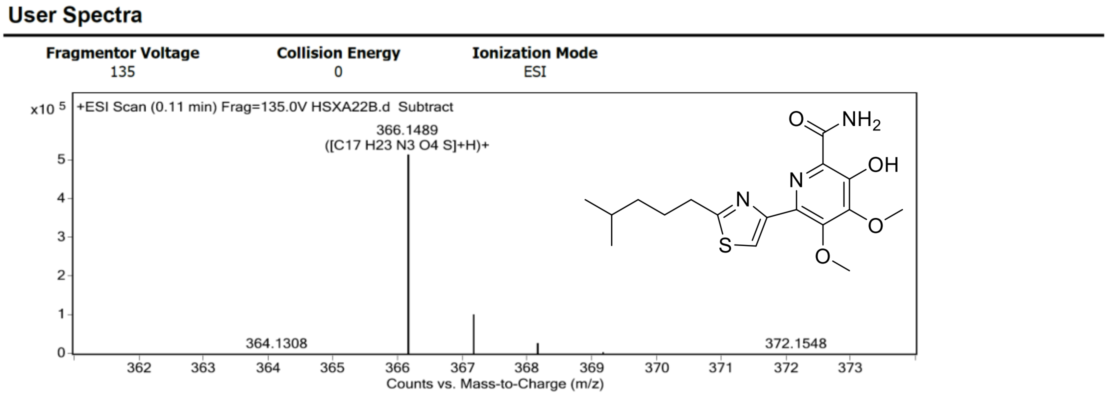

Supplementary Figure 19. HRESIMS spectrum of compound **4**.

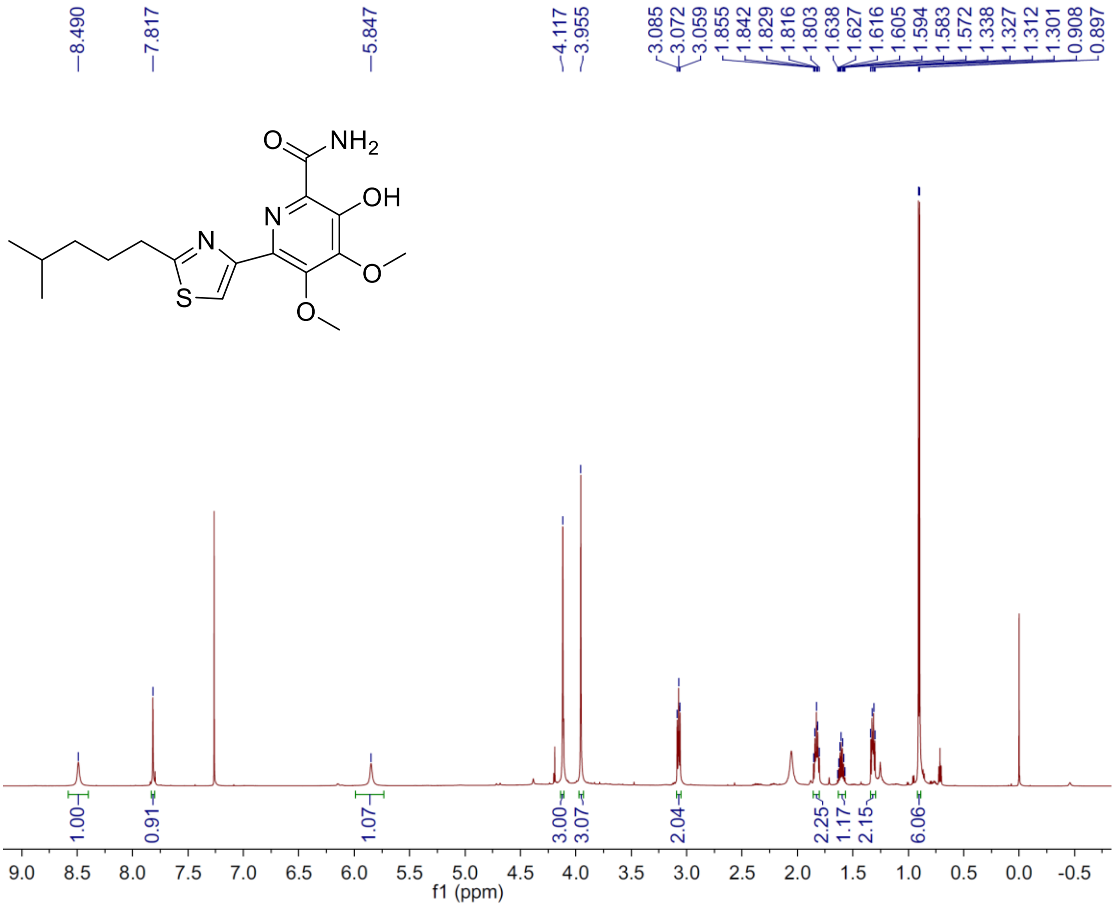

Supplementary Figure 20. ^{1}H-NMR (600 MHz) spectrum of compound **4** in $CDCl_3$.

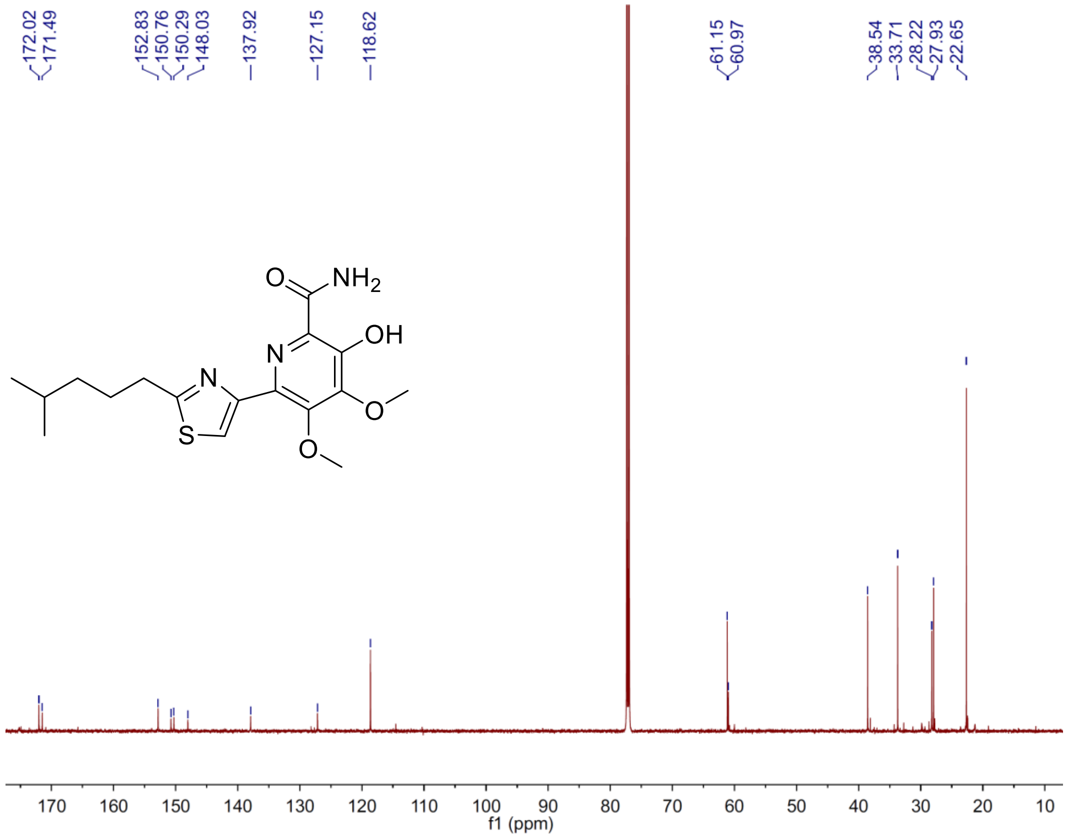

Supplementary Figure 21. ^{13}C-NMR (150 MHz) spectrum of compound **4** in $CDCl_3$.

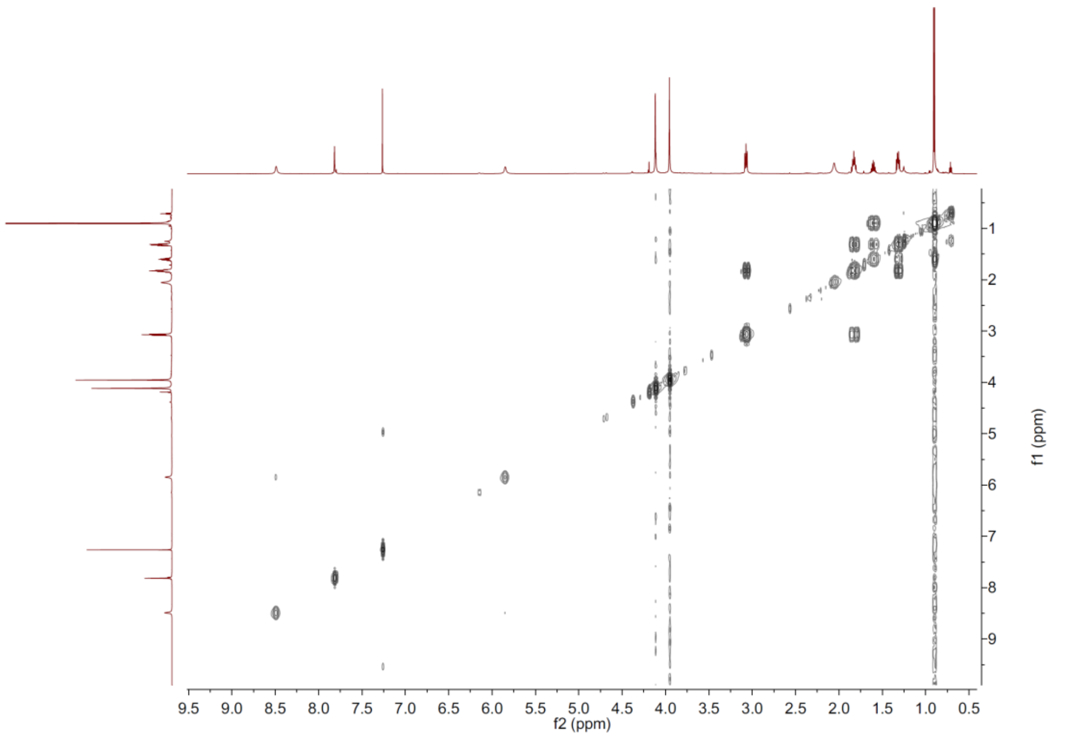

Supplementary Figure 22. ^{1}H-^{1}H COSY (600 MHz) spectrum of compound **4** in $CDCl_3$.

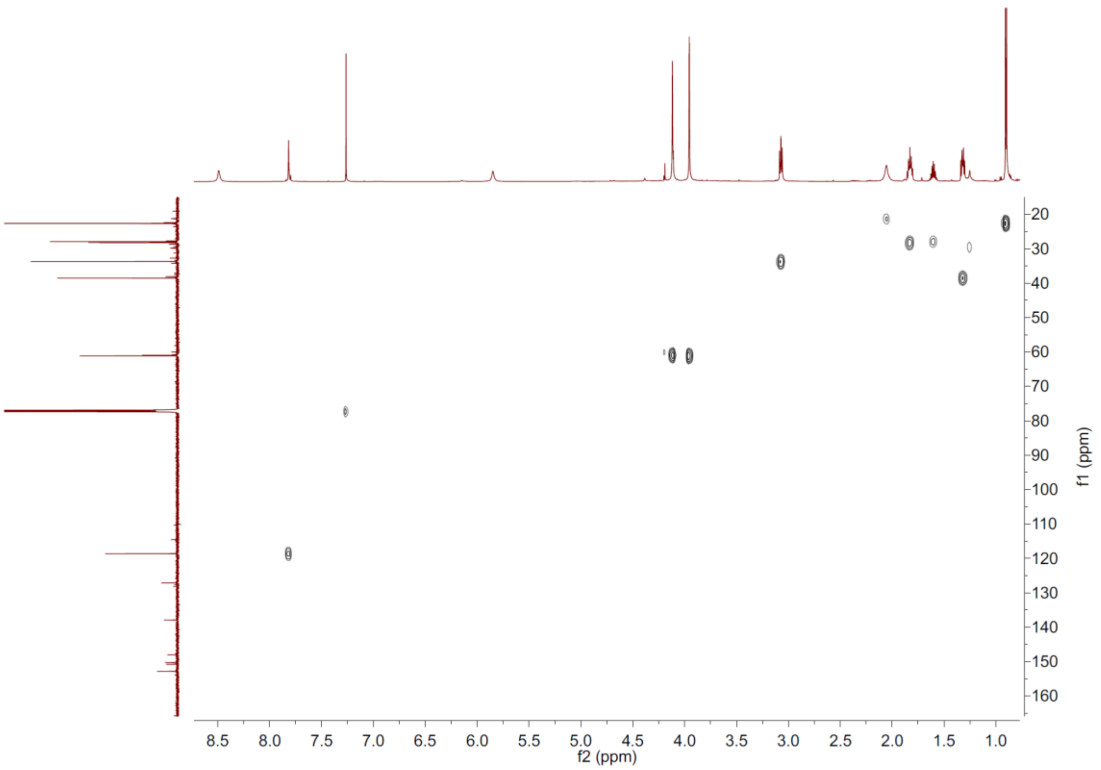

Supplementary Figure 23. HSQC (600 MHz) spectrum of compound **4** in $CDCl_3$.

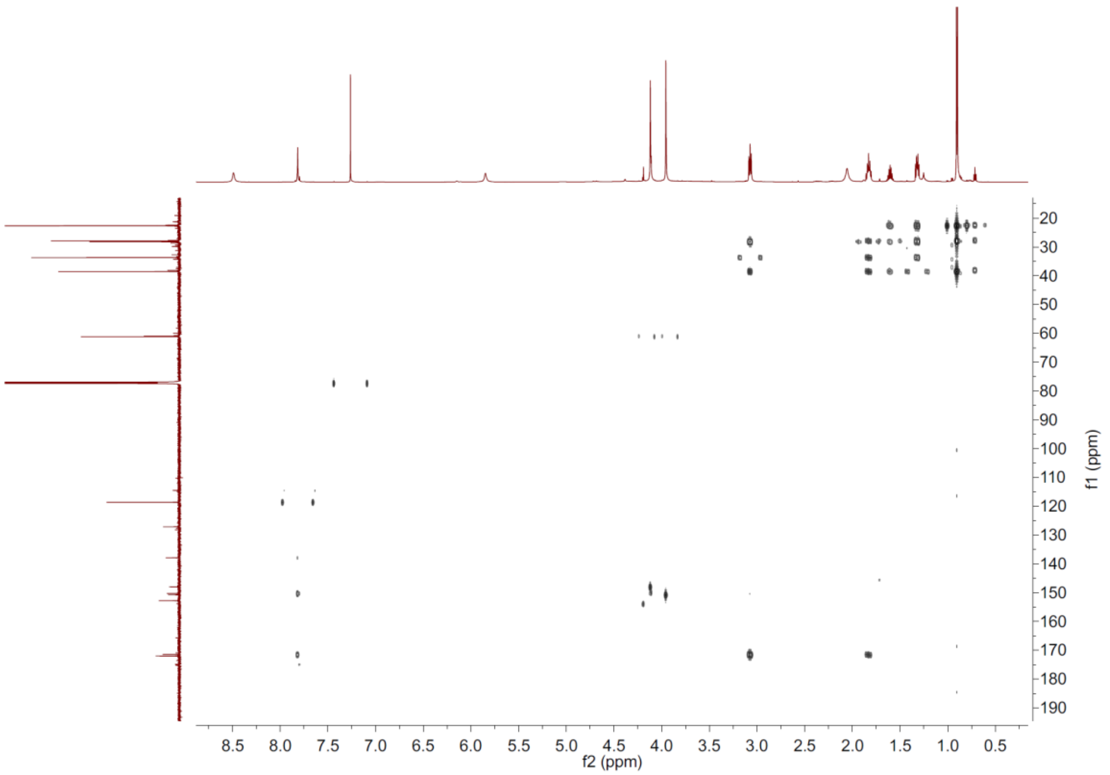

Supplementary Figure 24. HMBC (600 MHz) spectrum of compound **4** in $CDCl_3$.

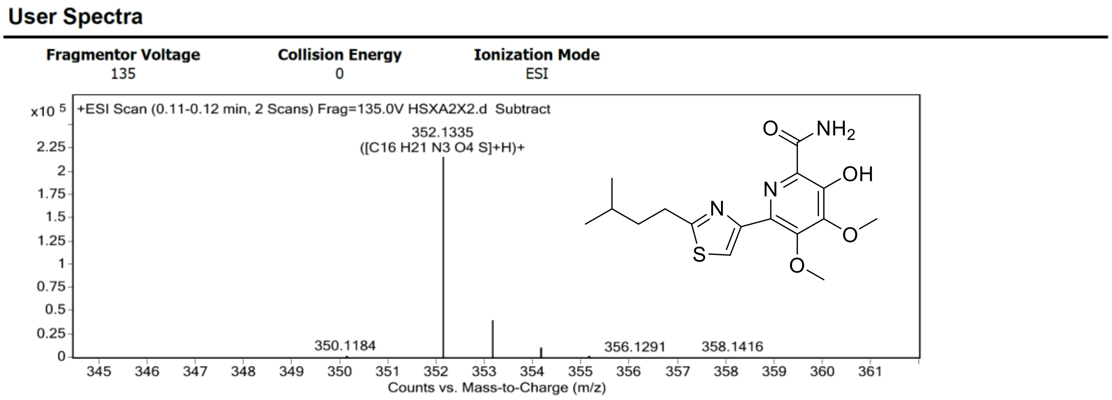

Supplementary Figure 25. HRESIMS spectrum of compound **5**.

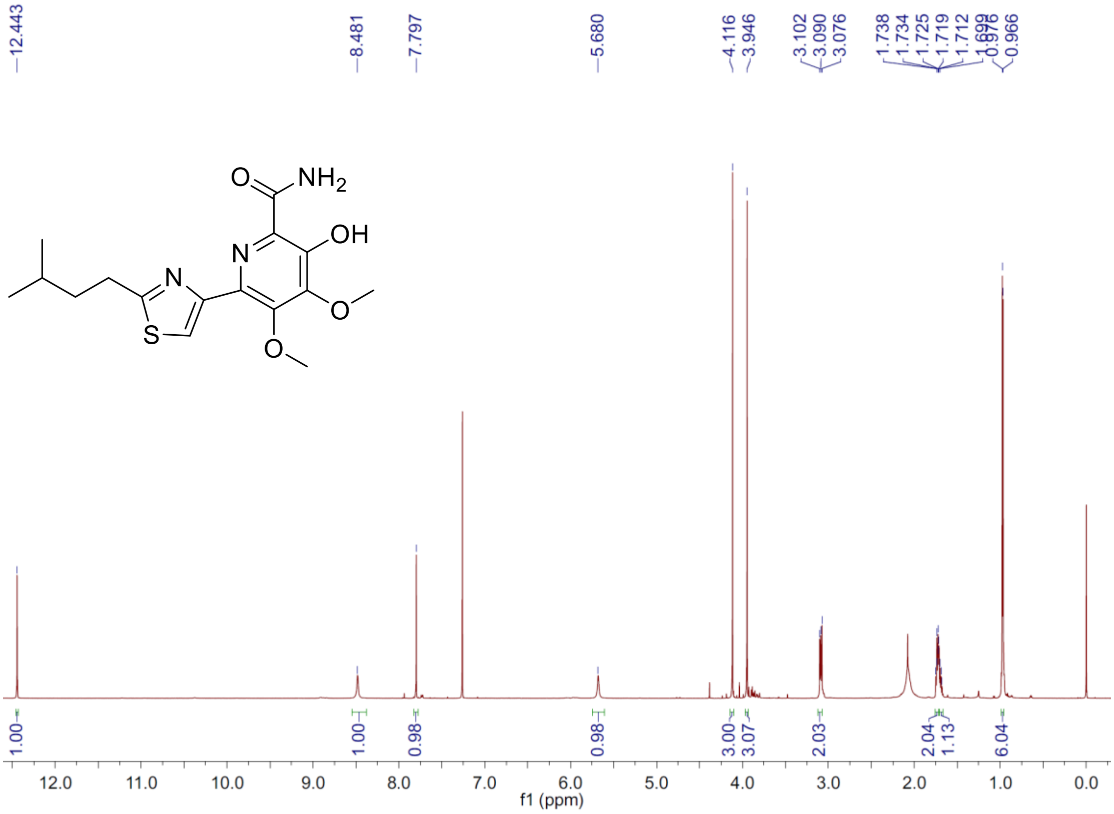

Supplementary Figure 26. ^{1}H-NMR (600 MHz) spectrum of compound **5** in $CDCl_3$.

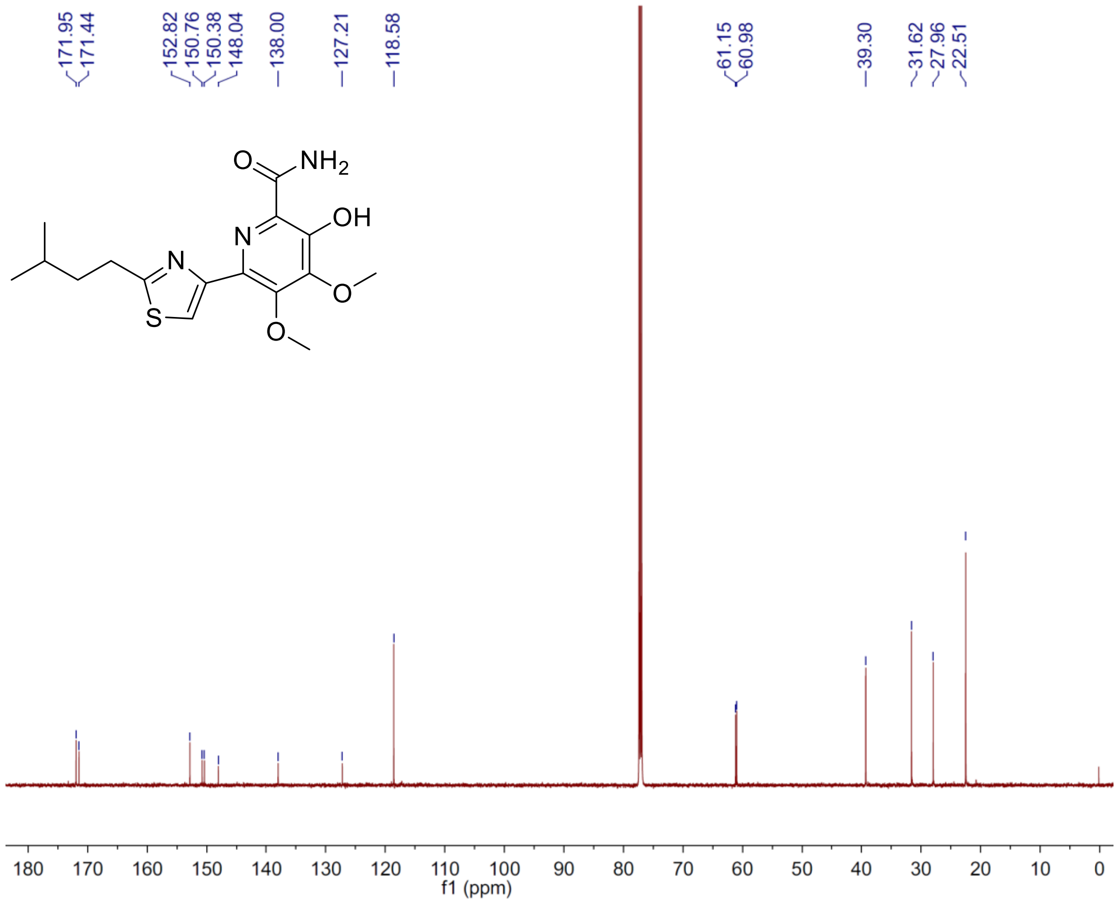

Supplementary Figure 27. ^{13}C-NMR (150 MHz) spectrum of compound **5** in $CDCl_3$.

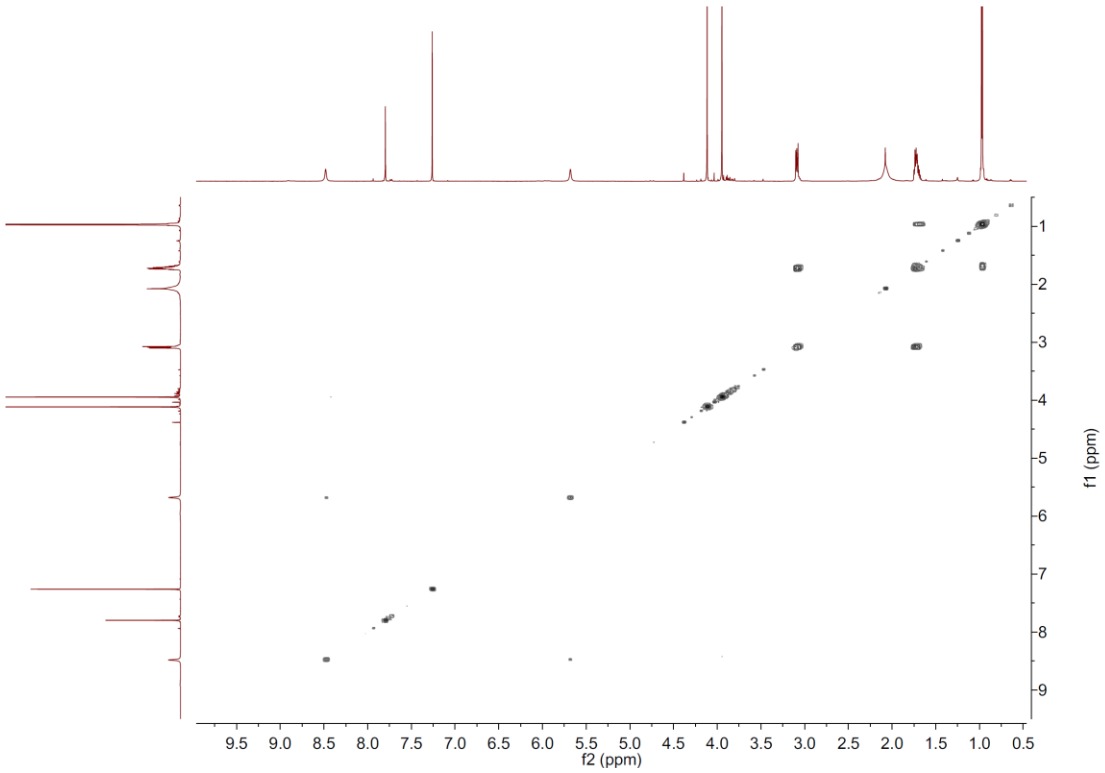

Supplementary Figure 28. ^{1}H-^{1}H COSY (600 MHz) spectrum of compound **5** in $CDCl_3$.

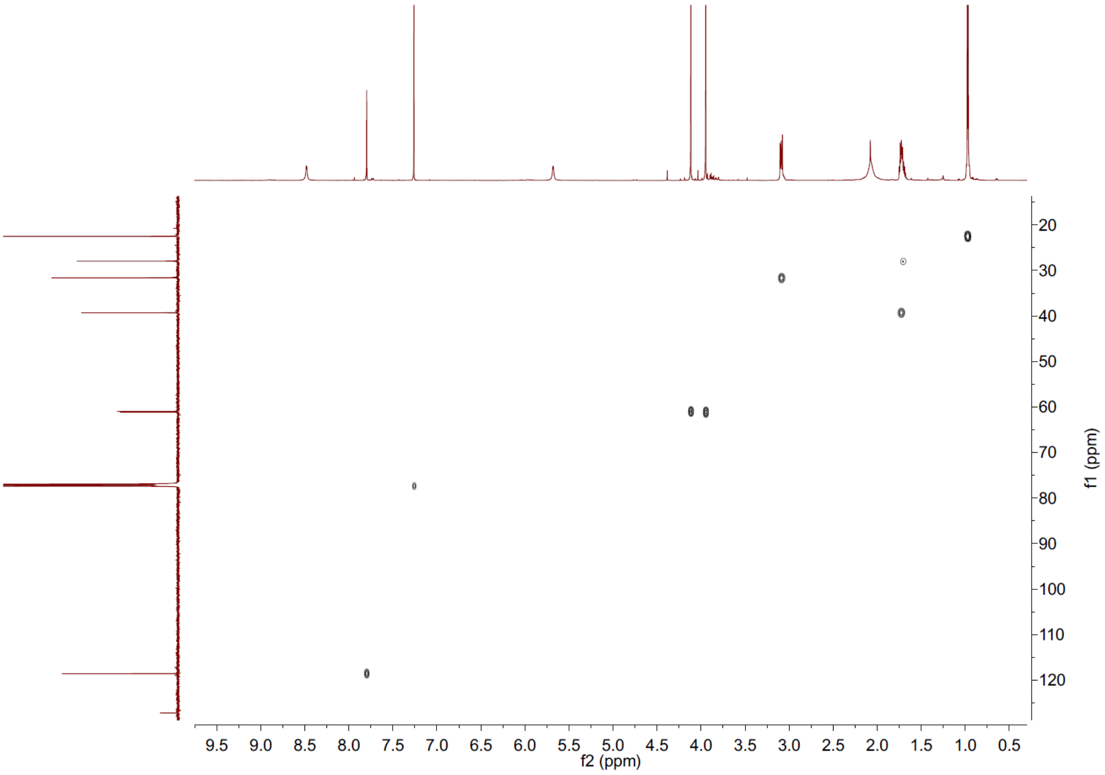

Supplementary Figure 29. HSQC (600 MHz) spectrum of compound **5** in $CDCl_3$.

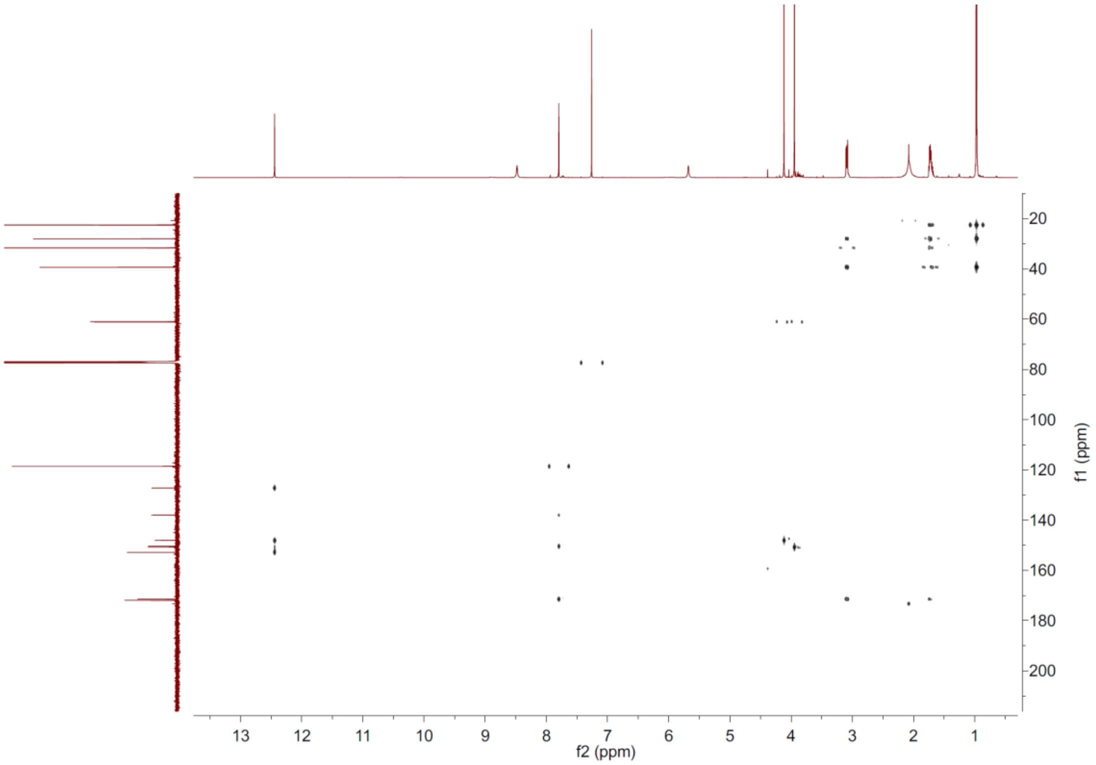

Supplementary Figure 30. HMBC (600 MHz) spectrum of compound **5** in $CDCl_3$.

User Spectra

Fragmentor Voltage	Collision Energy	Ionization Mode
135	0	ESI

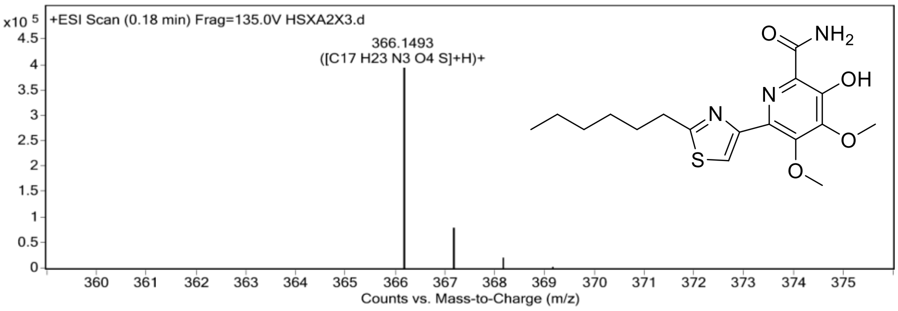

Supplementary Figure 31. HRESIMS spectrum of compound **6**

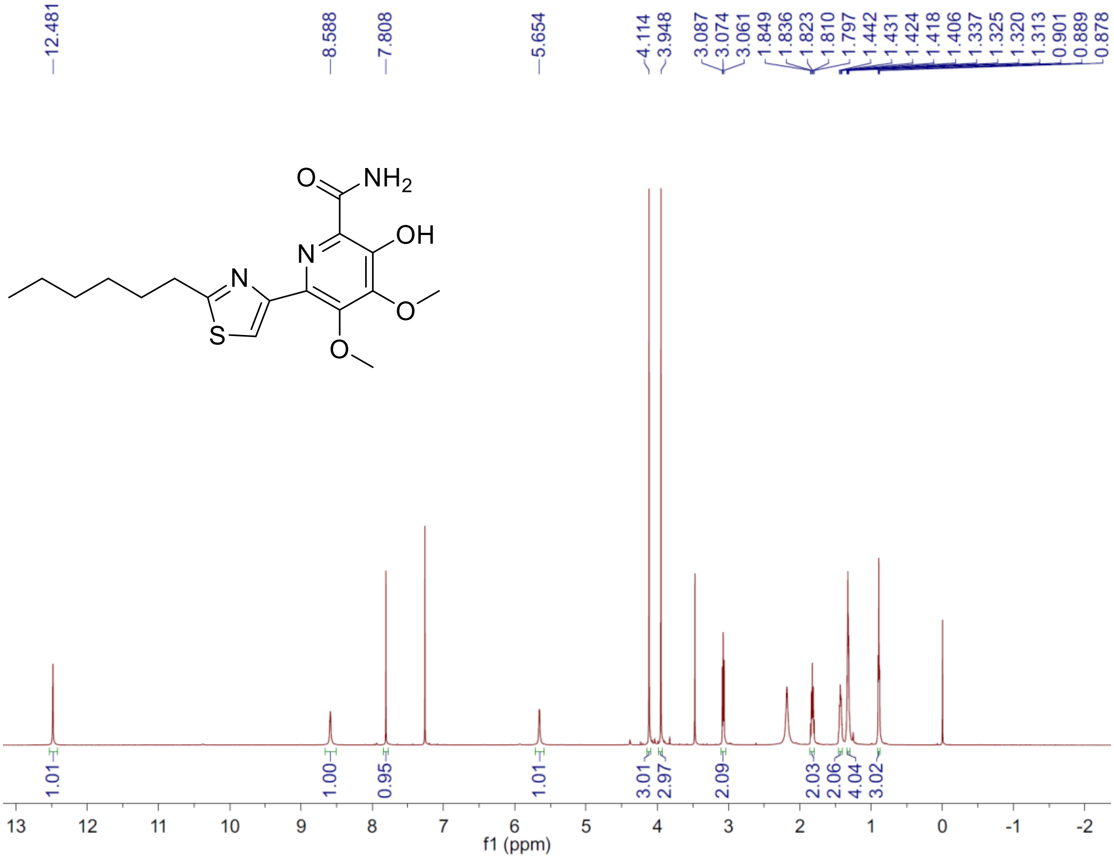

Supplementary Figure 32. ^{1}H-NMR (600 MHz) spectrum of compound **6** in $CDCl_3$.

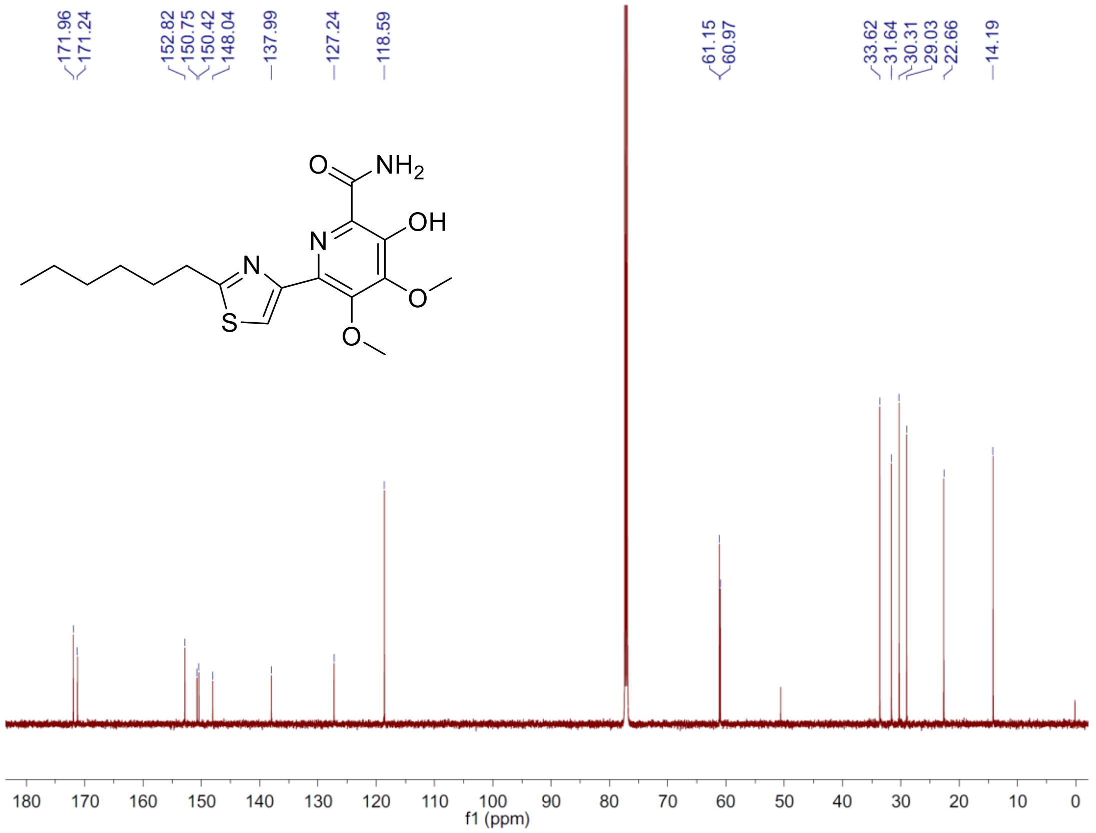

Supplementary Figure 33. ^{13}C-NMR (150 MHz) spectrum of compound **6** in $CDCl_3$.

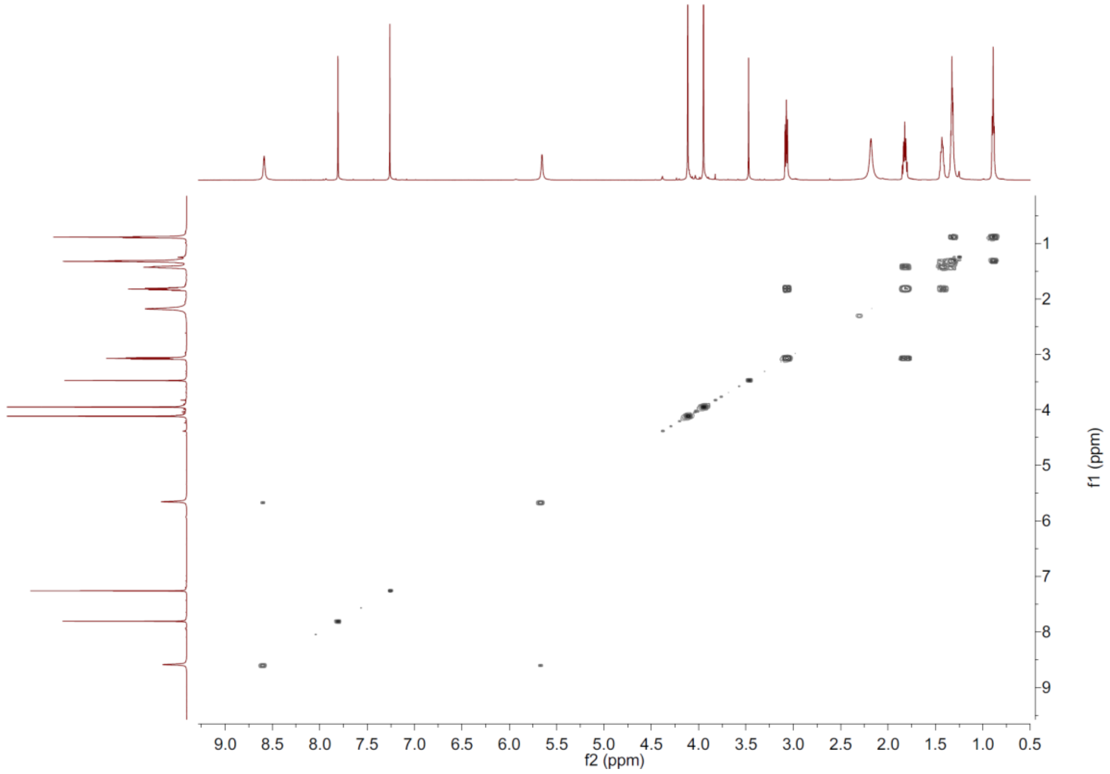

Supplementary Figure 34. ^{1}H-^{1}H-COSY (600 MHz) spectrum of compound **6** in $CDCl_3$.

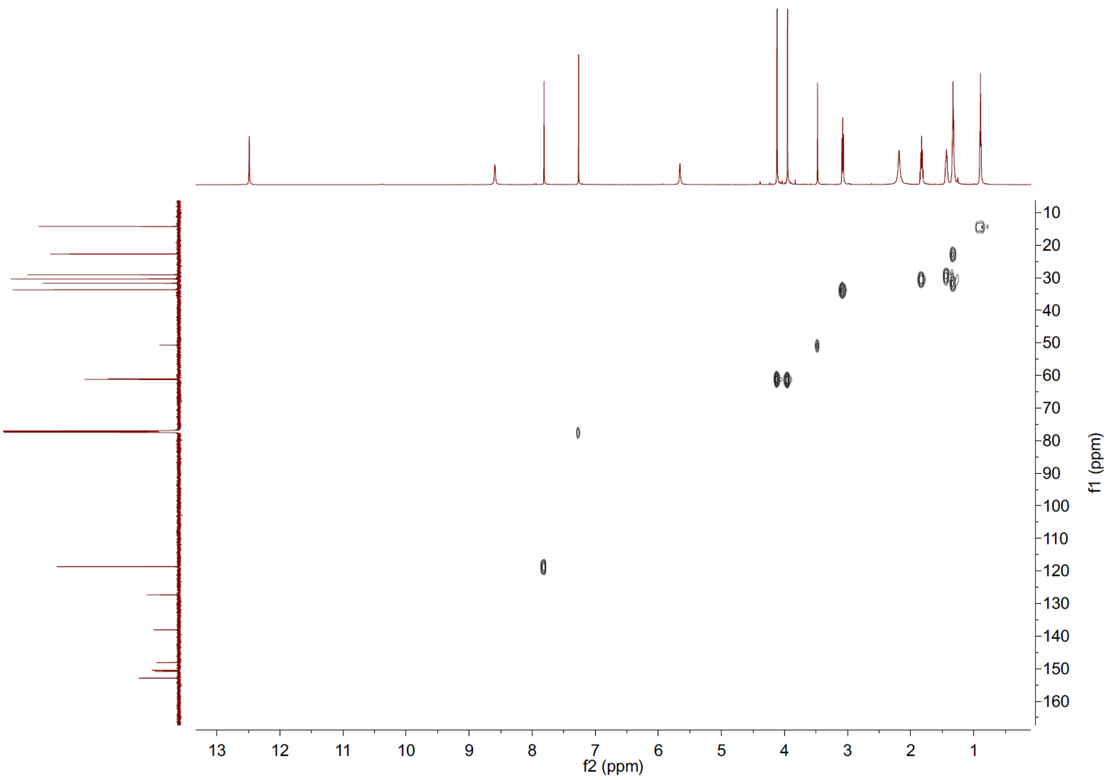

Supplementary Figure 35. HSQC (600 MHz) spectrum of compound **6** in $CDCl_3$.

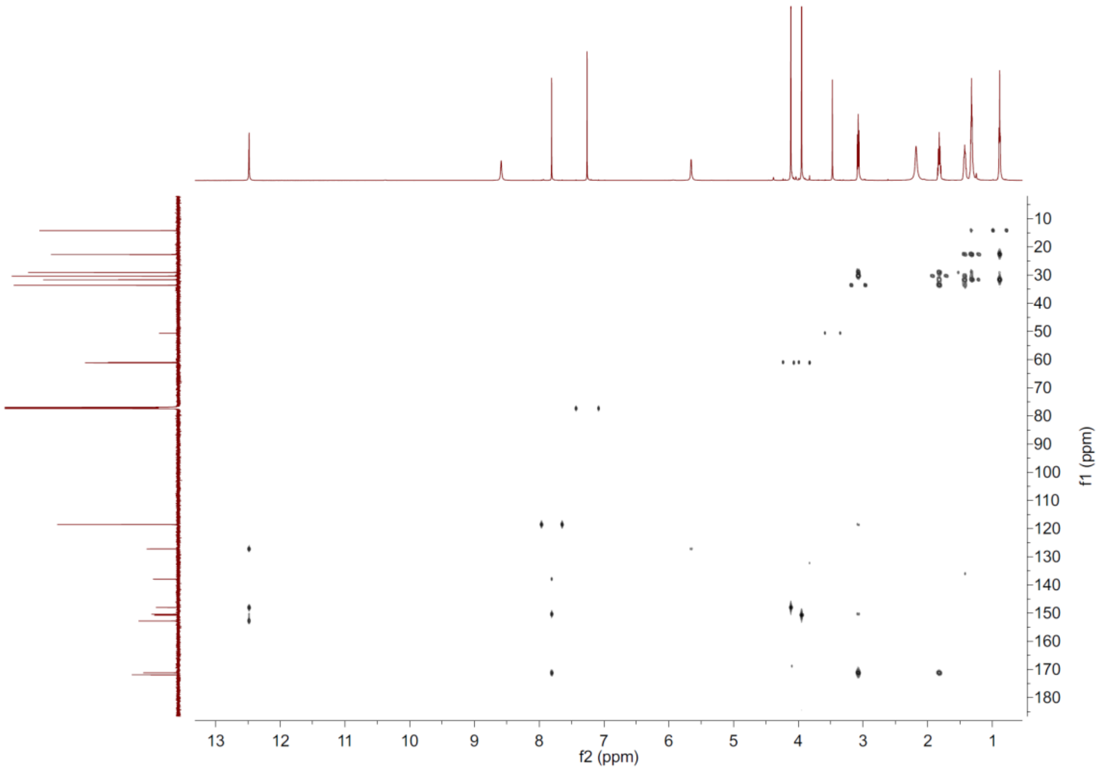

Supplementary Figure 36. HMBC (600 MHz) spectrum of compound **6** in $CDCl_3$.

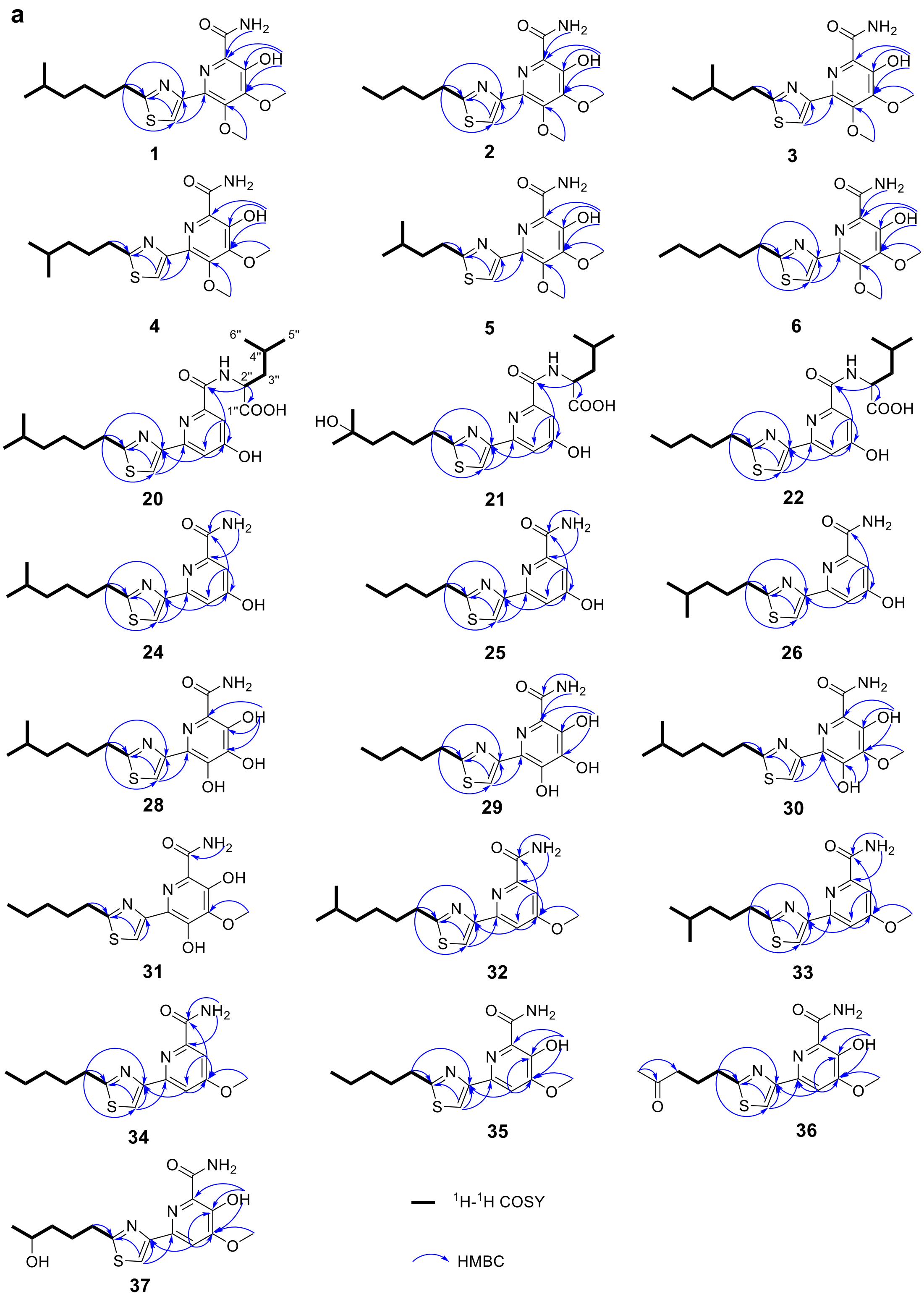
a
1
2
3
4
5
6
20
21
22
24
25
26
28
29
30
31
32
33
34
35
36
37
1H-1H COSY
HMBC

b

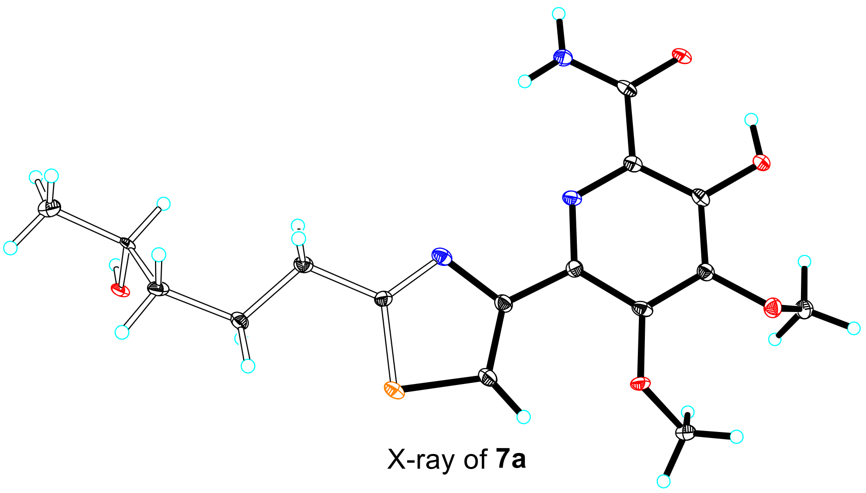

X-ray of **7a**

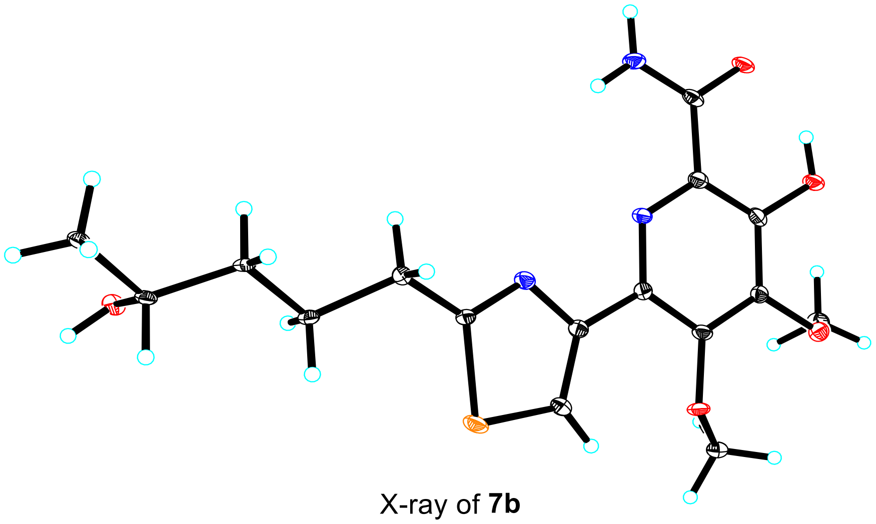

X-ray of **7b**

Supplementary Figure 37. 2D NMR correlations and ORTEP structures. (**a**) 2D NMR correlations of compounds **1-6**, **20-22**, **24-26** and **28-37**. (**b**) ORTEP representation (30% probability ellipsoids) of the X-ray structure of compound **7**.

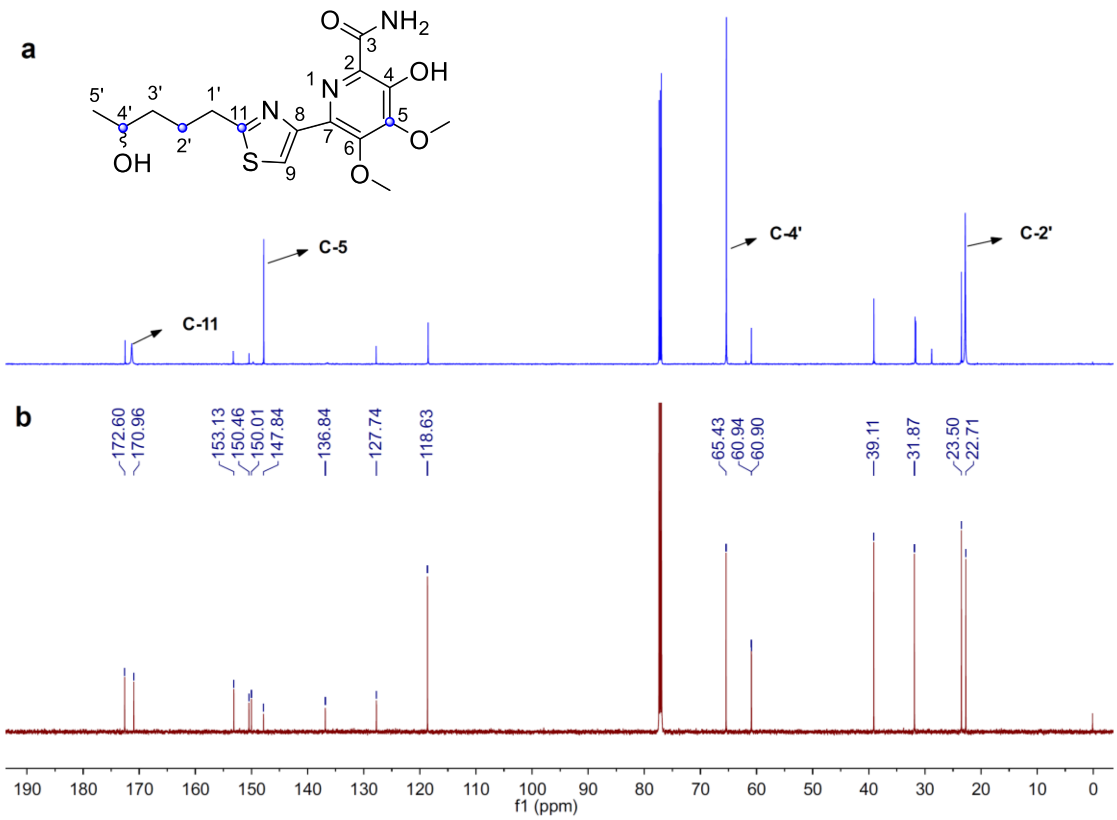

Supplementary Figure 38. ^{13}C NMR (150 MHz) spectrum of **7** in $CDCl_3$. (**a**) **7** labeled with [1-^{13}C] acetate. (**b**) Natural abundance.

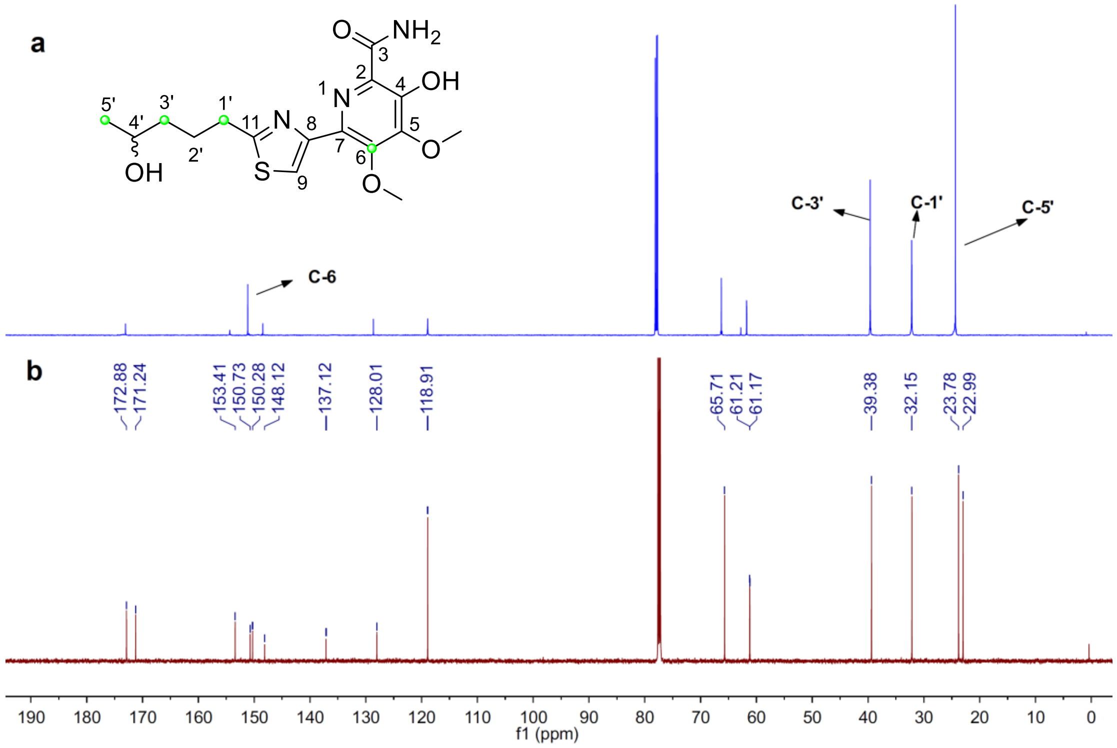

Supplementary Figure 39. ^{13}C NMR (150 MHz) spectrum of **7** in CDCl3. (**a**) **7** labeled with [2-^{13}C] acetate. (**b**) Natural abundance.

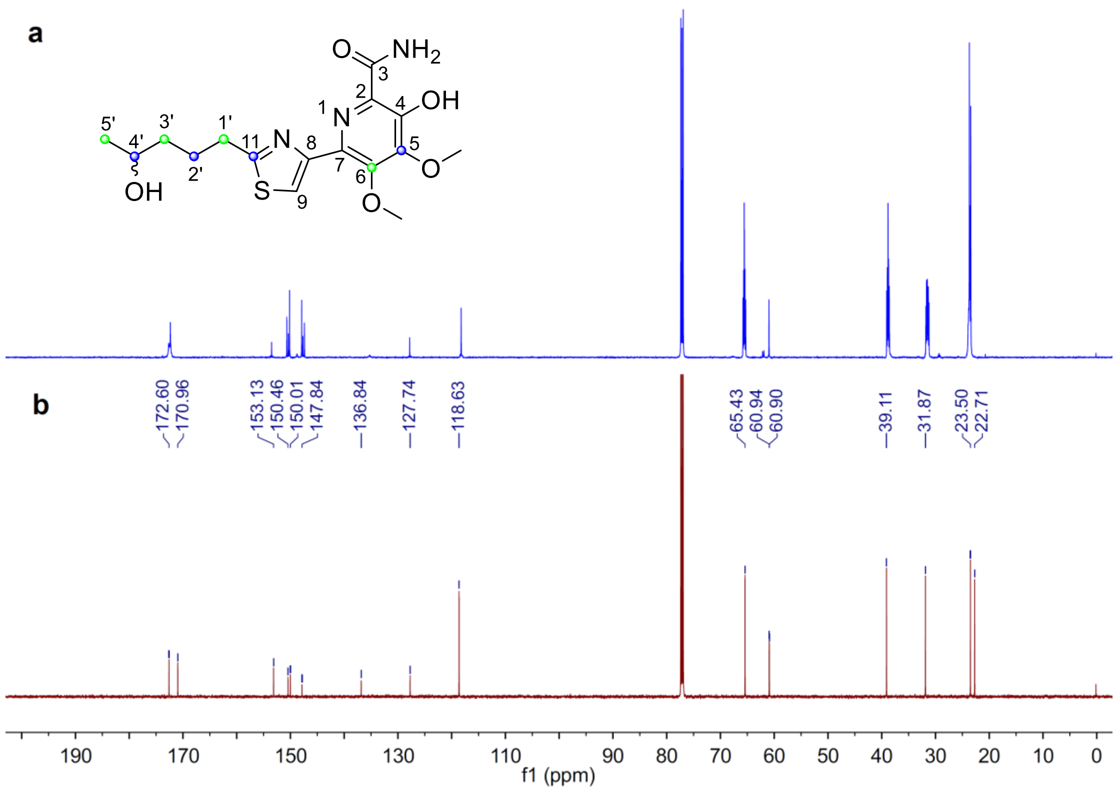

Supplementary Figure 40. ^{13}C NMR (150 MHz) spectrum of **7** in $CDCl_3$. (**a**) **7** labeled with [1, 2-$^{13}C_2$] acetate. (**b**) Natural abundance.

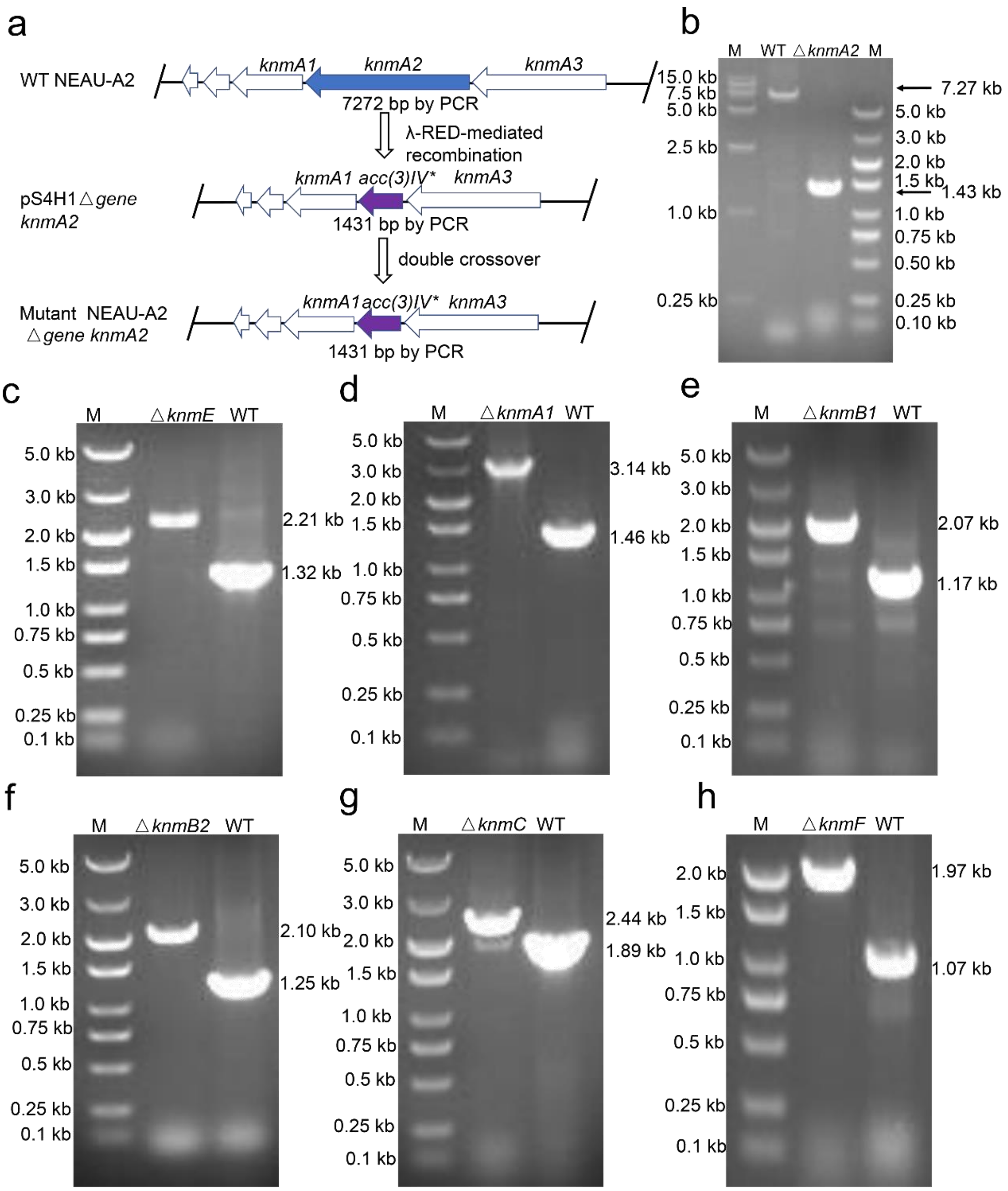

Supplementary Figure 41. Construction of in-frame deletion in *L. rhizosphaerae* NEAU-A2. (**a**) Gene replacement of *knm* using the PCR-targeting method. (**b-h**) PCR verification of *knm* mutants by PCR using the primers listed in supplementary Table 22. Lane M: DNA molecular ladder; Lane Δ*knm*, PCR product from NEAU-A2 mutants; Lane WT, PCR product from the strain NEAU-A2. A representative result of *n* = 2 independent experiments is shown.

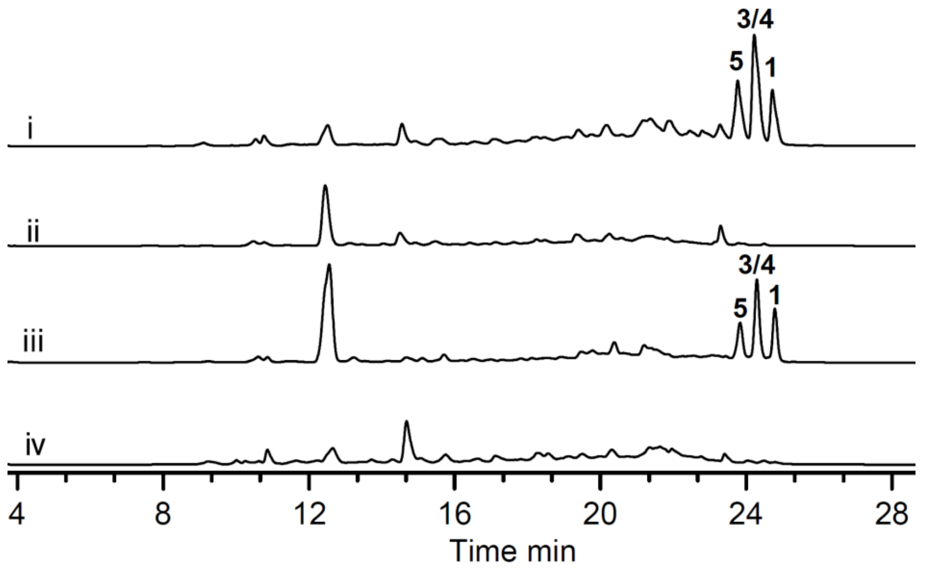

Supplementary Figure 42. HPLC analysis (analytical method D) of metabolites from the heterologous expression strains: (i) *S. albus* 5C1-Δ*knmC*/*knmC*; (ii) *S. albus* 5C1-Δ*knmC*; (iii) *S. albus* 5C1; (iv) *S. albus* J1074 control (strain containing vector alone). HPLC analysis was performed with a 30 min gradient elution system as follows: T = 0 min, 10% B; T = 20 min, 100% B; T = 25 min, 100% B; T = 25.1 min, 10% B; T = 30 min, 10% B (A, H_2O; B, CH_3OH). A representative result of *n* = 3 independent experiments is shown. Source data are provided as a Source Data file.

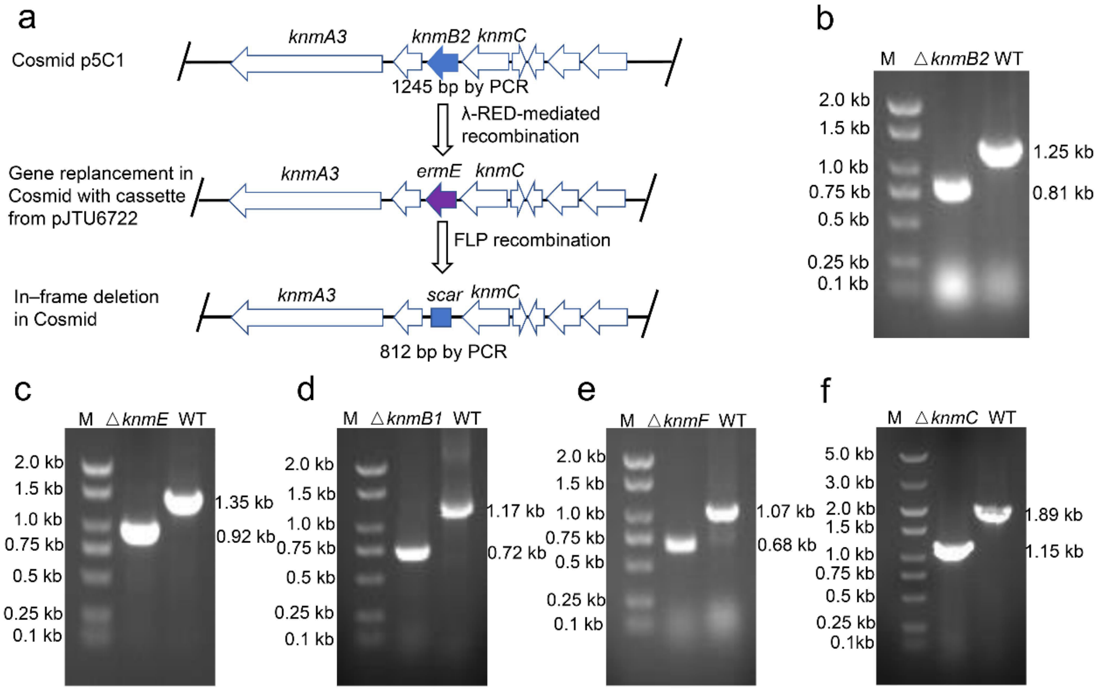

Supplementary Figure 43. Construction of in-frame deletion in *S. albus* 5C1. (**a**) Gene replacement of *knm* using the PCR-targeting method. (**b-f**) PCR verification of *knm* mutants by PCR using the primers listed in supplementary Table 22. Lane M: DNA molecular ladder; Lane Δ*knm*, PCR product from *S. albus* 5C1 mutants; Lane WT, PCR product from the strain *S. albus* 5C1. A representative result of *n* = 2 independent experiments is shown.

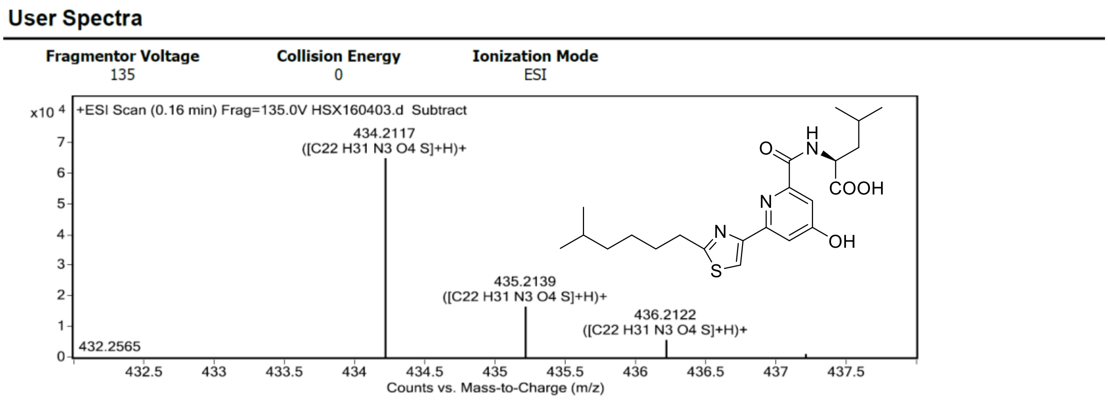

Supplementary Figure 44. HRESIMS spectrum of compound **20**.

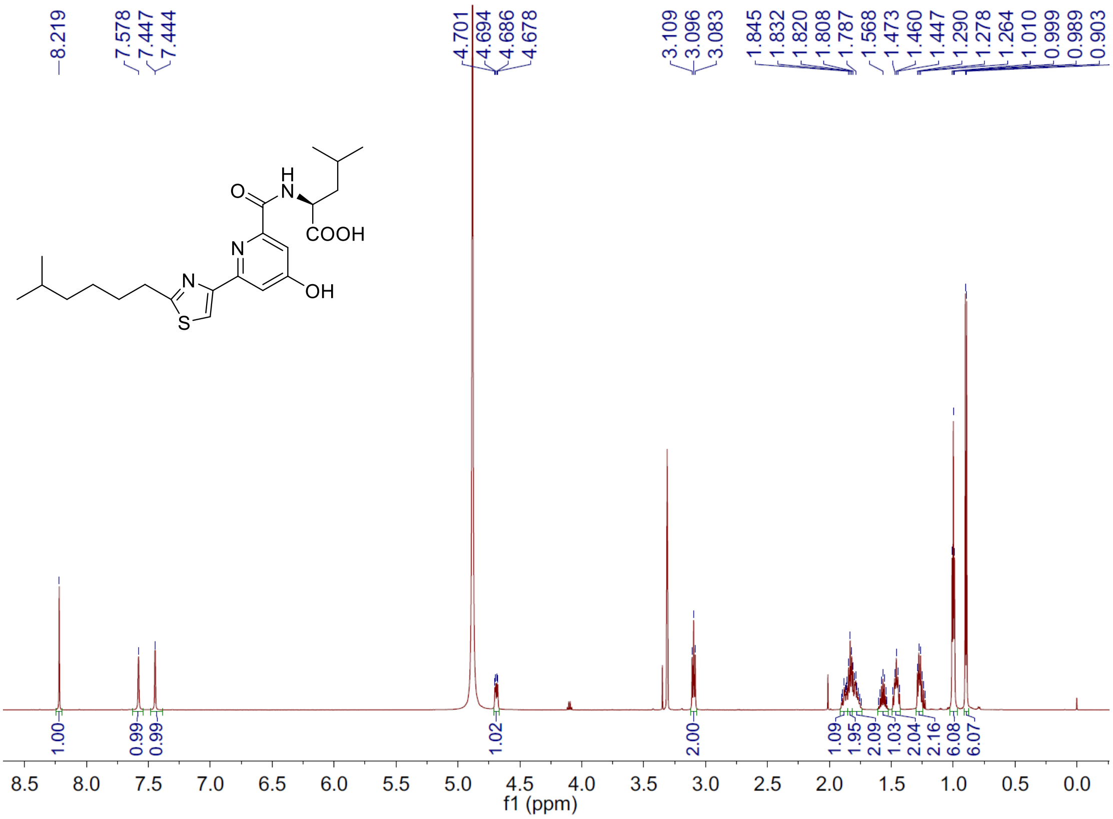

Supplementary Figure 45. ^{1}H NMR (600 MHz) spectrum of compound **20** in MeOD.

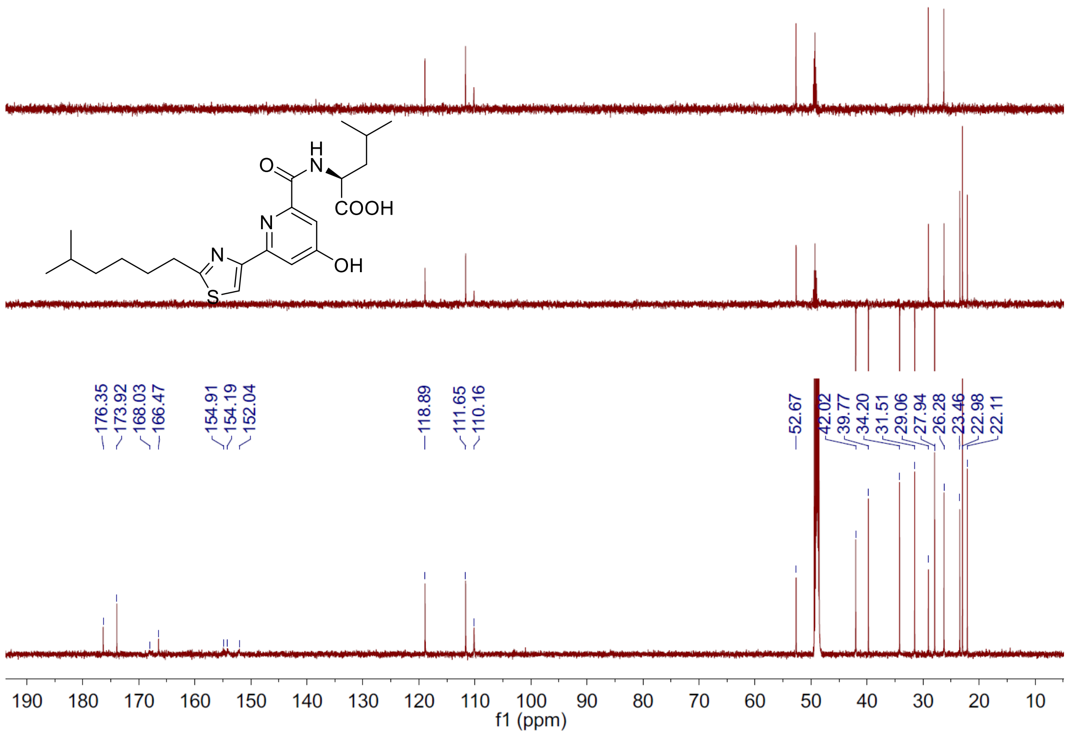

Supplementary Figure 46. ^{13}C NMR (150 MHz) spectrum of compound **20** in MeOD.

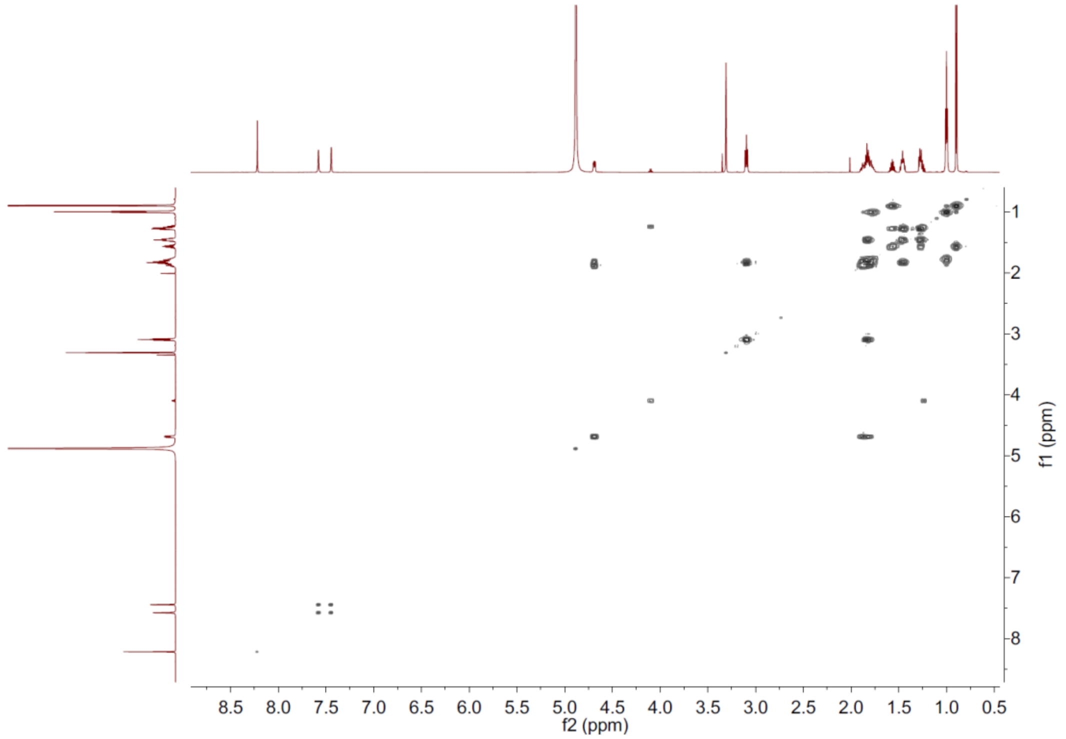

Supplementary Figure 47. ^{1}H-^{1}H COSY (600 MHz) spectrum of compound **20** in MeOD.

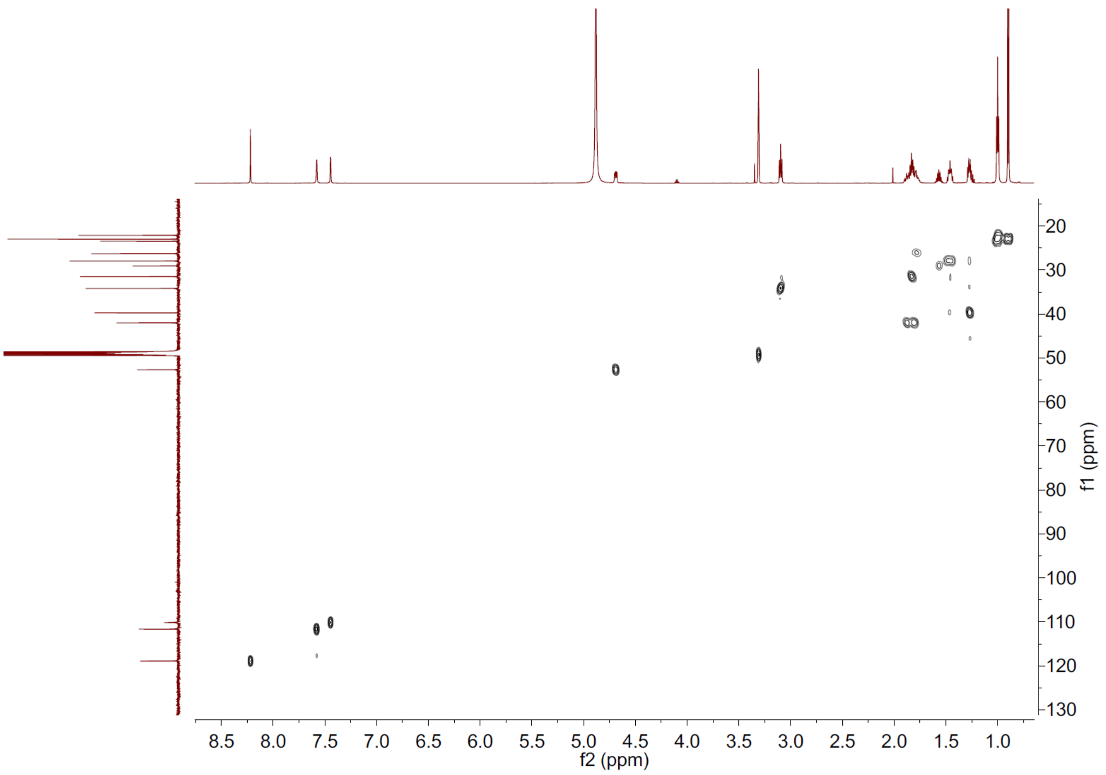

Supplementary Figure 48. HSQC (600 MHz) spectrum of compound **20** in MeOD.

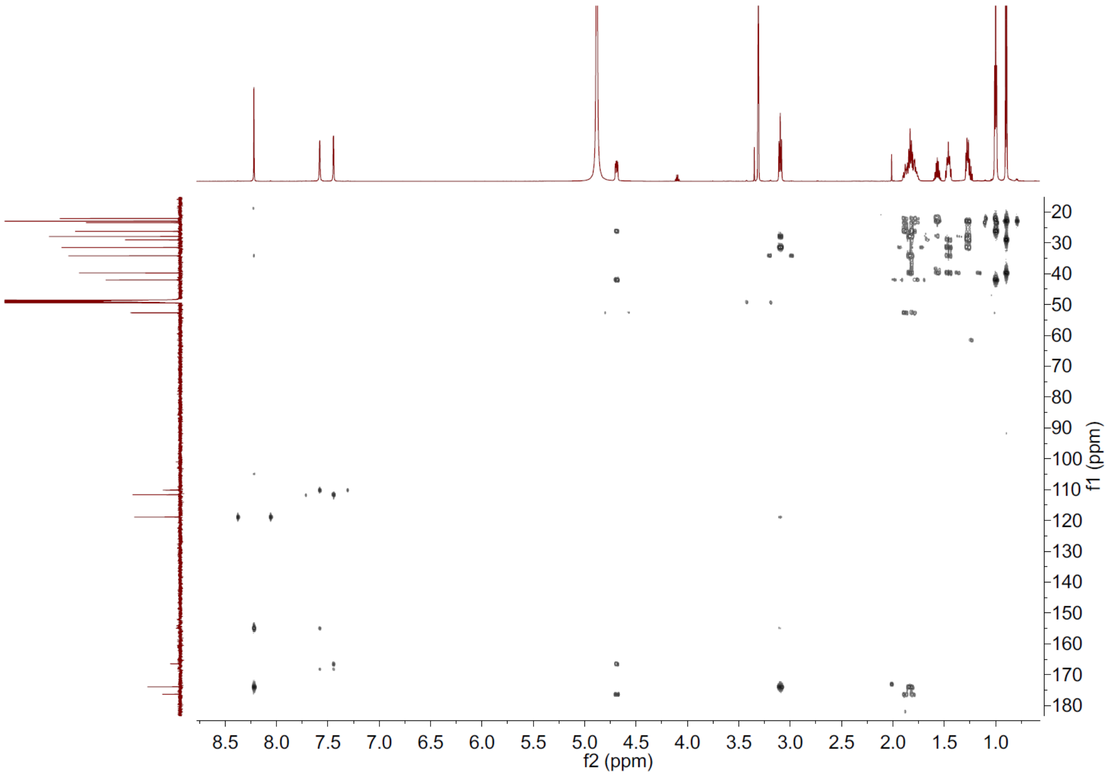

Supplementary Figure 49. HMBC (600 MHz) spectrum of compound **20** in MeOD.

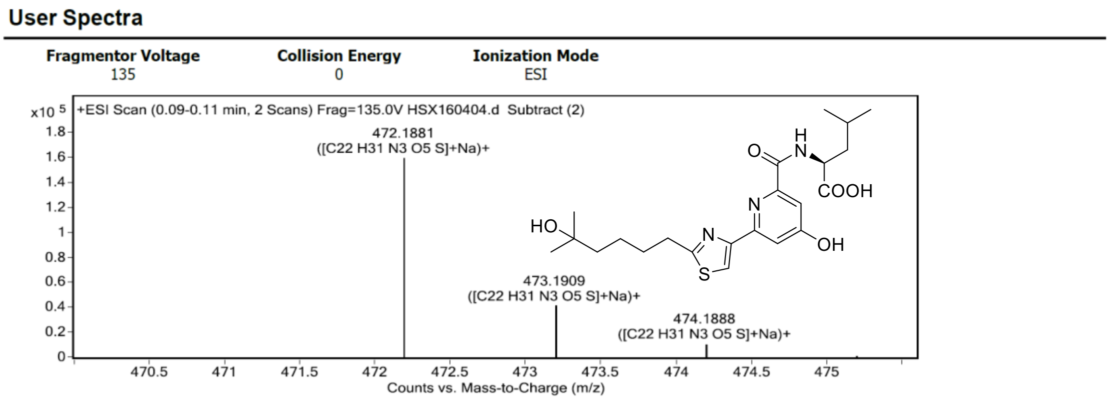

Supplementary Figure 50. HRESIMS spectrum of compound **21**.

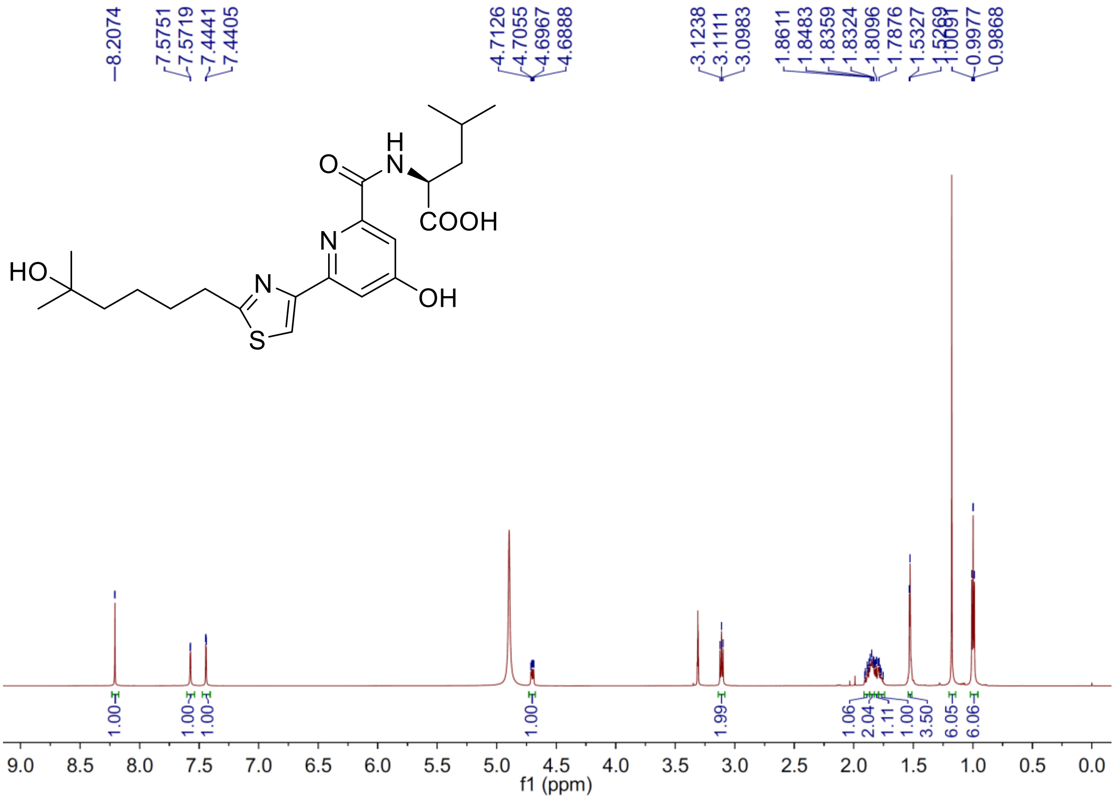

Supplementary Figure 51. ^{1}H-NMR (600 MHz) spectrum of compound **21** in MeOD.

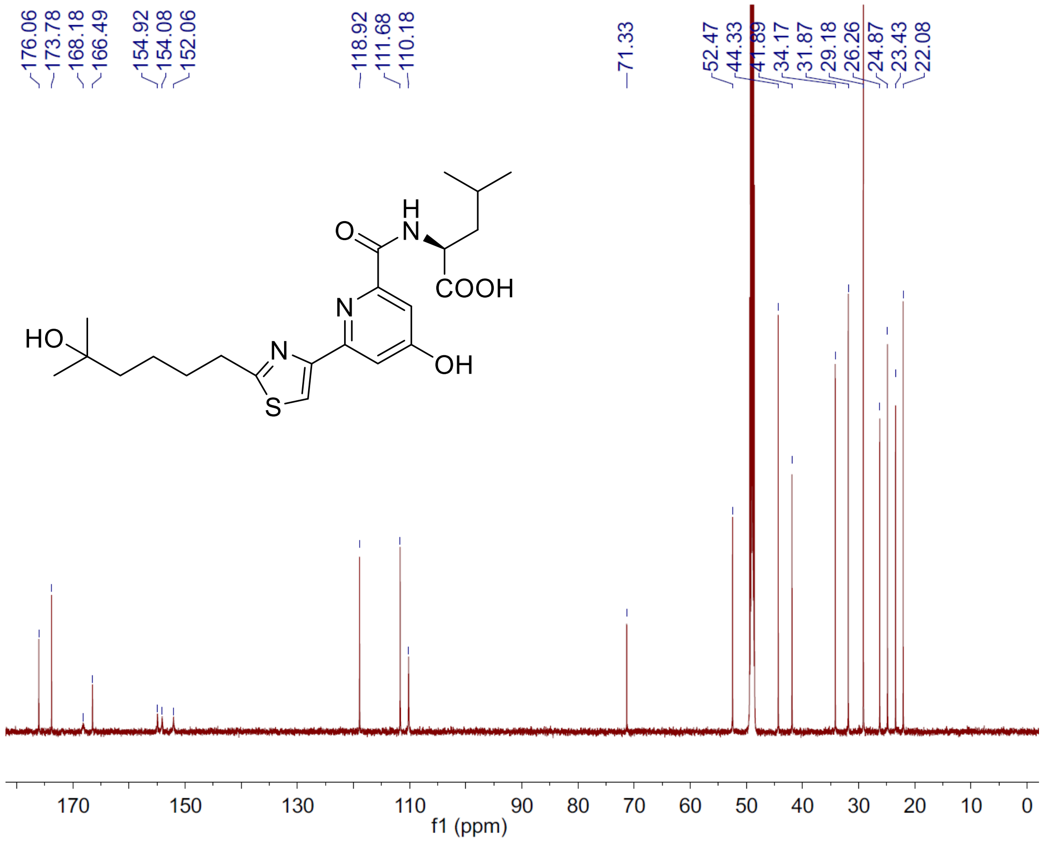

Supplementary Figure 52. ^{13}C-NMR (150 MHz) spectrum of compound **21** in MeOD.

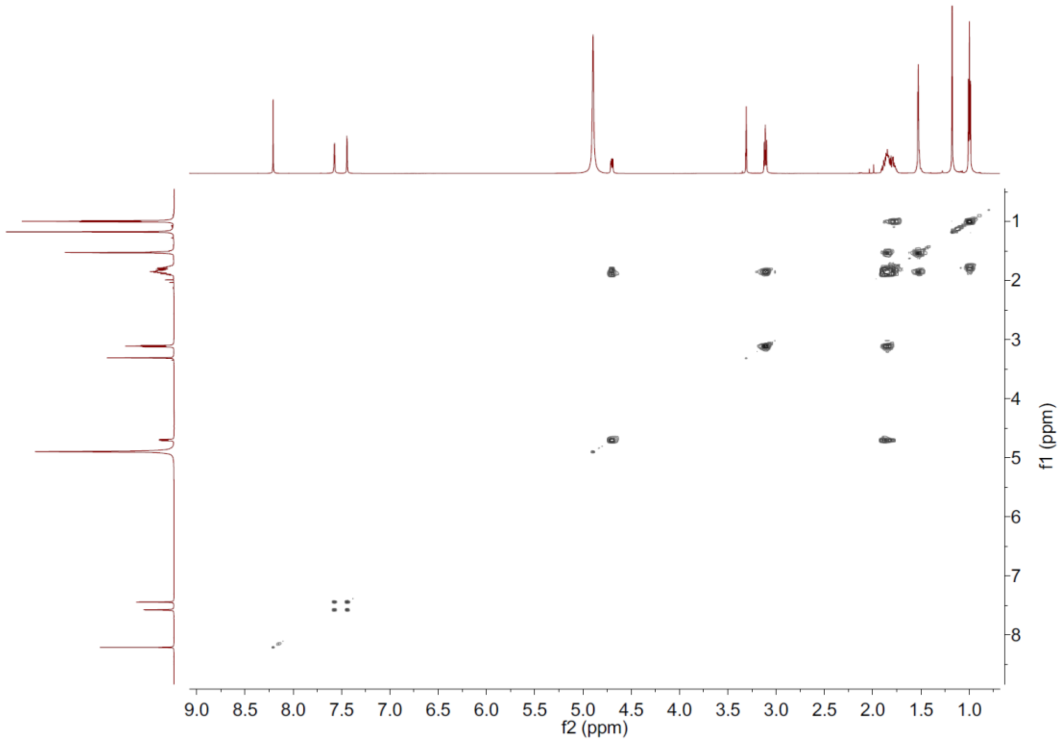

Supplementary Figure 53. ^{1}H-^{1}H COSY (600 MHz) spectrum of compound **21** in MeOD.

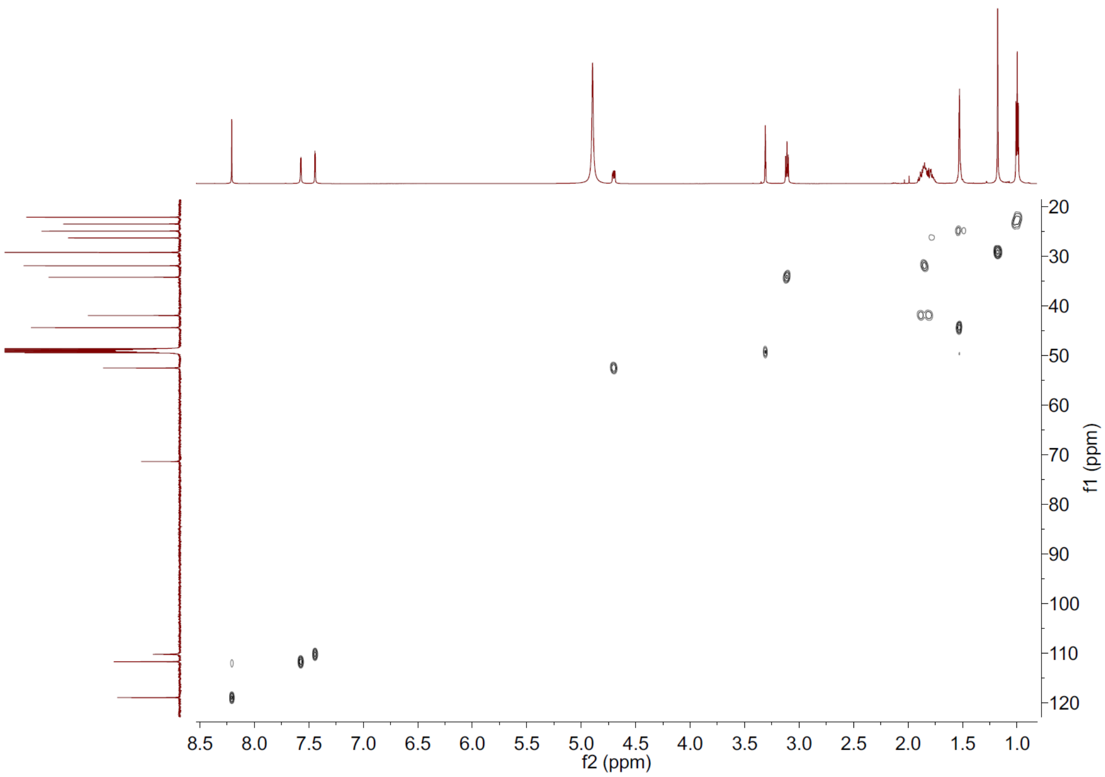

Supplementary Figure 54. HSQC (600 MHz) spectrum of compound **21** in MeOD.

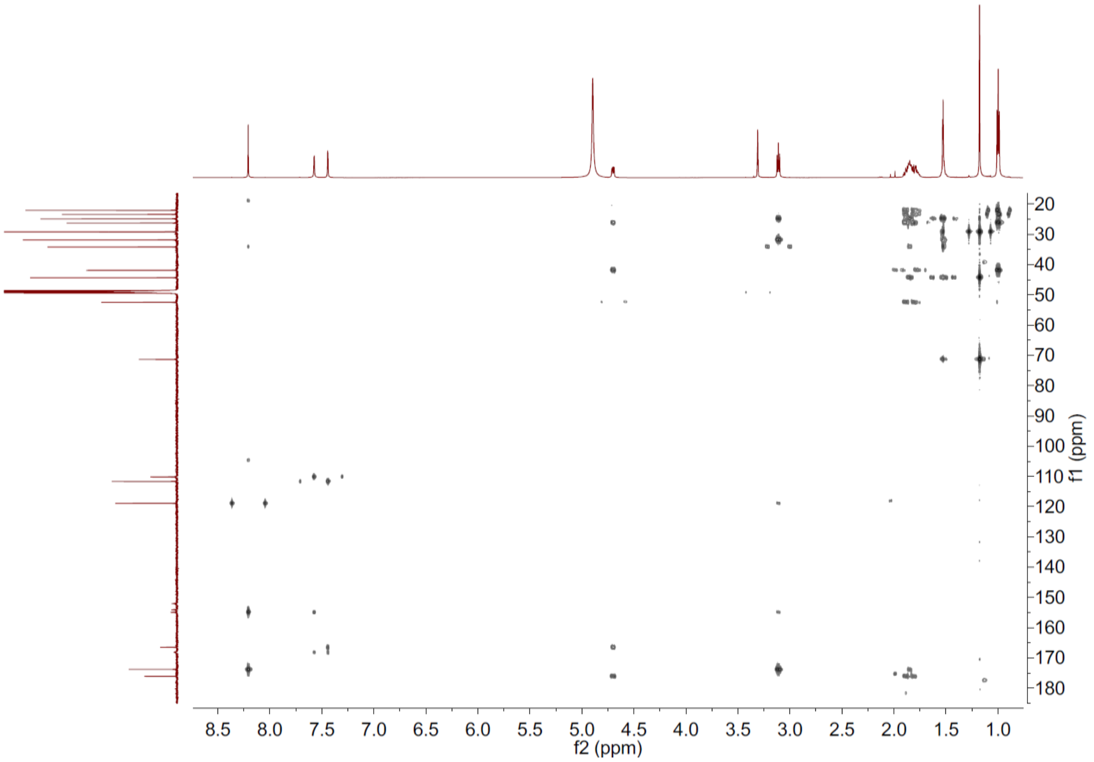

Supplementary Figure 55. HMBC (600 MHz) spectrum of compound **21** in MeOD.

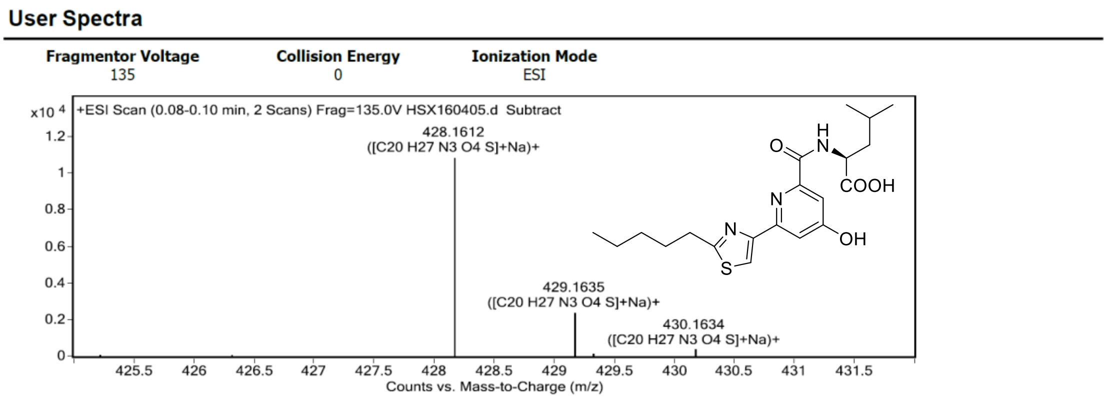

Supplementary Figure 56. HRESIMS spectrum of compound **22**.

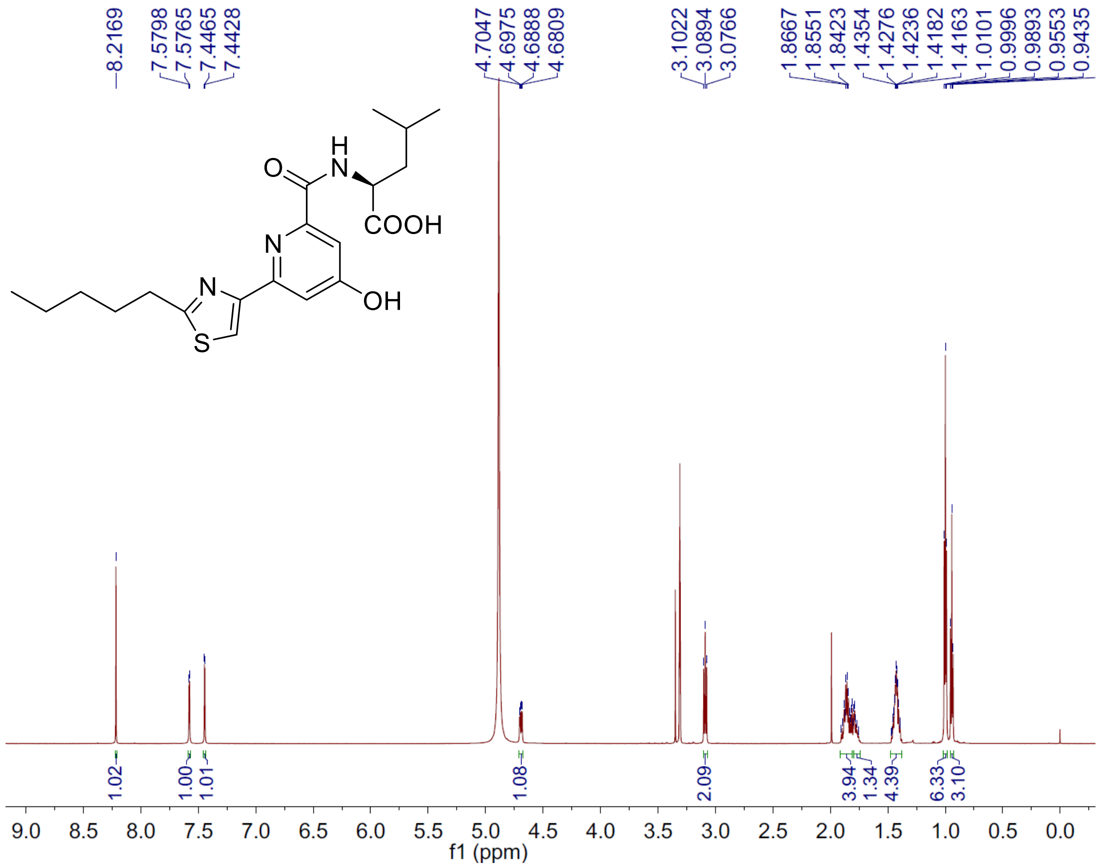

Supplementary Figure 57. ^{1}H-NMR (600 MHz) spectrum of compound **22** in MeOD.

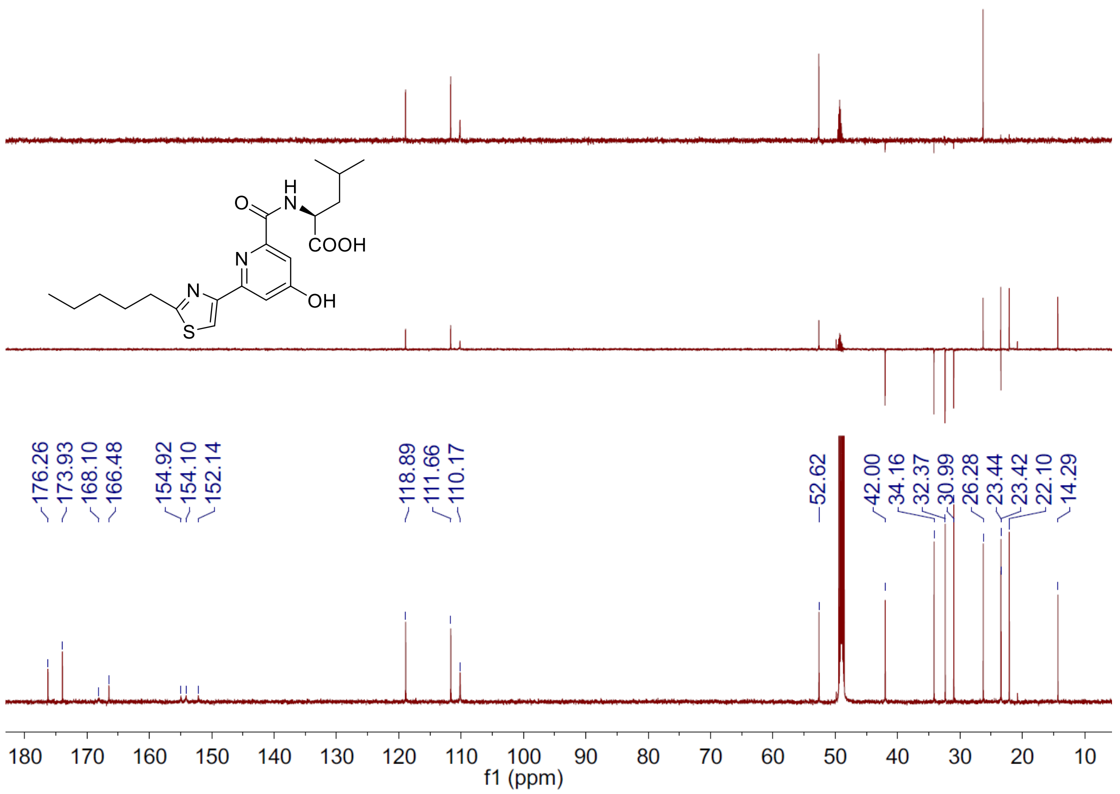

Supplementary Figure 58. ^{13}C-NMR (150 MHz) spectrum of compound **22** in MeOD.

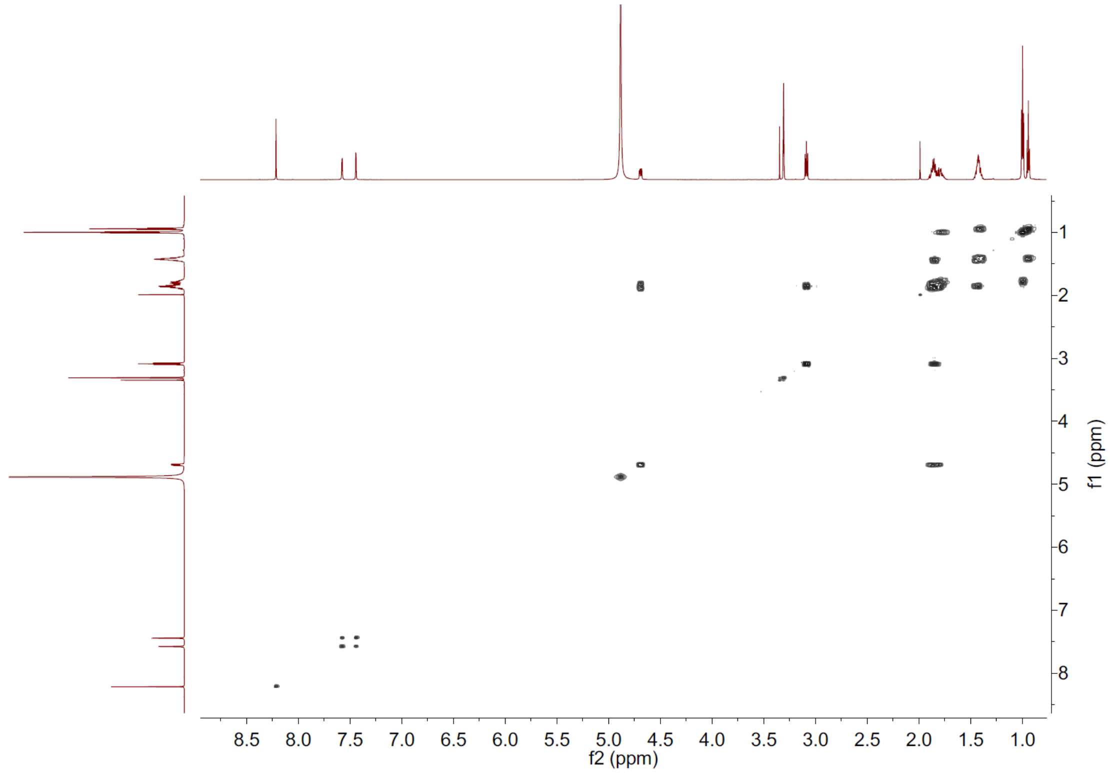

Supplementary Figure 59. ^{1}H-^{1}H COSY (600 MHz) spectrum of compound **22** in MeOD.

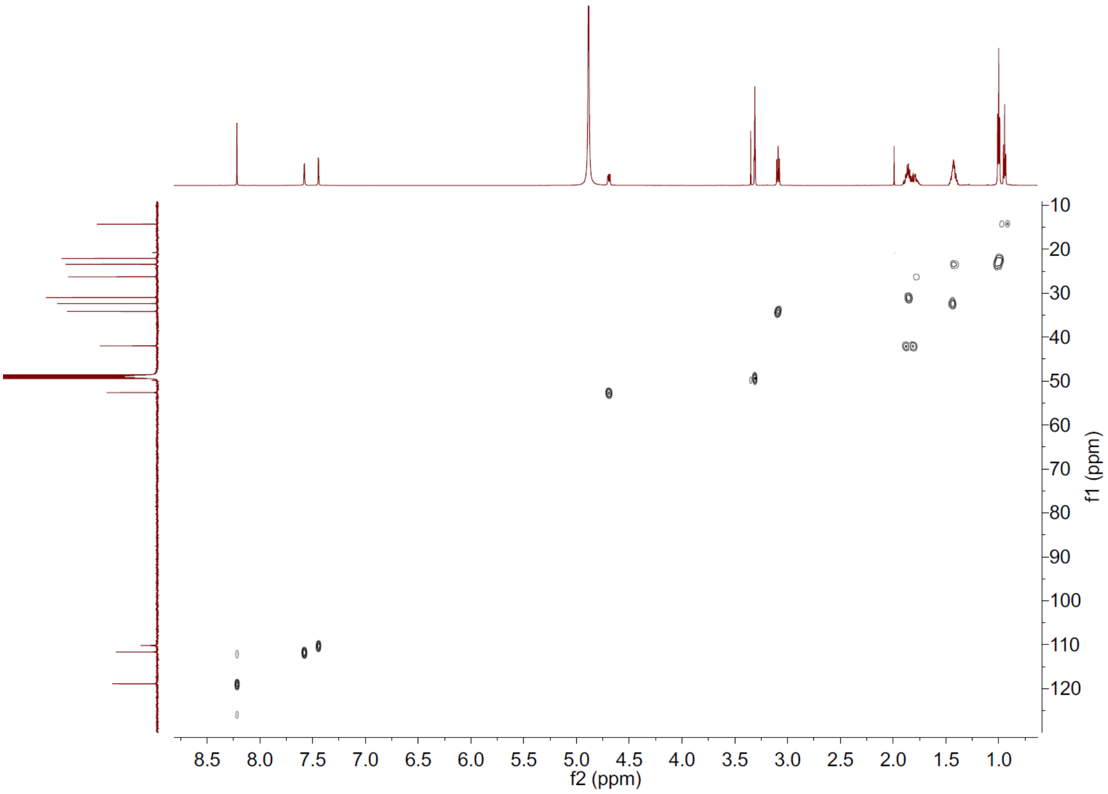

Supplementary Figure 60. HSQC (600 MHz) spectrum of compound **22** in MeOD.

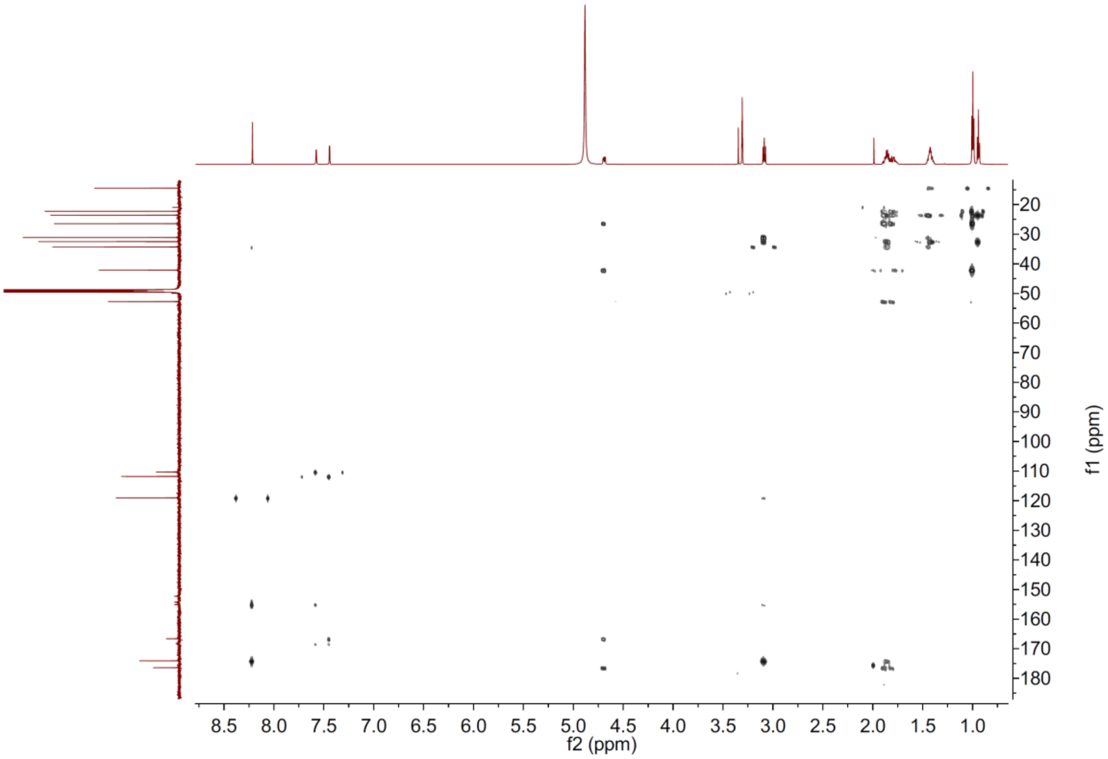

Supplementary Figure 61. HMBC (600 MHz) spectrum of compound **22** in MeOD.

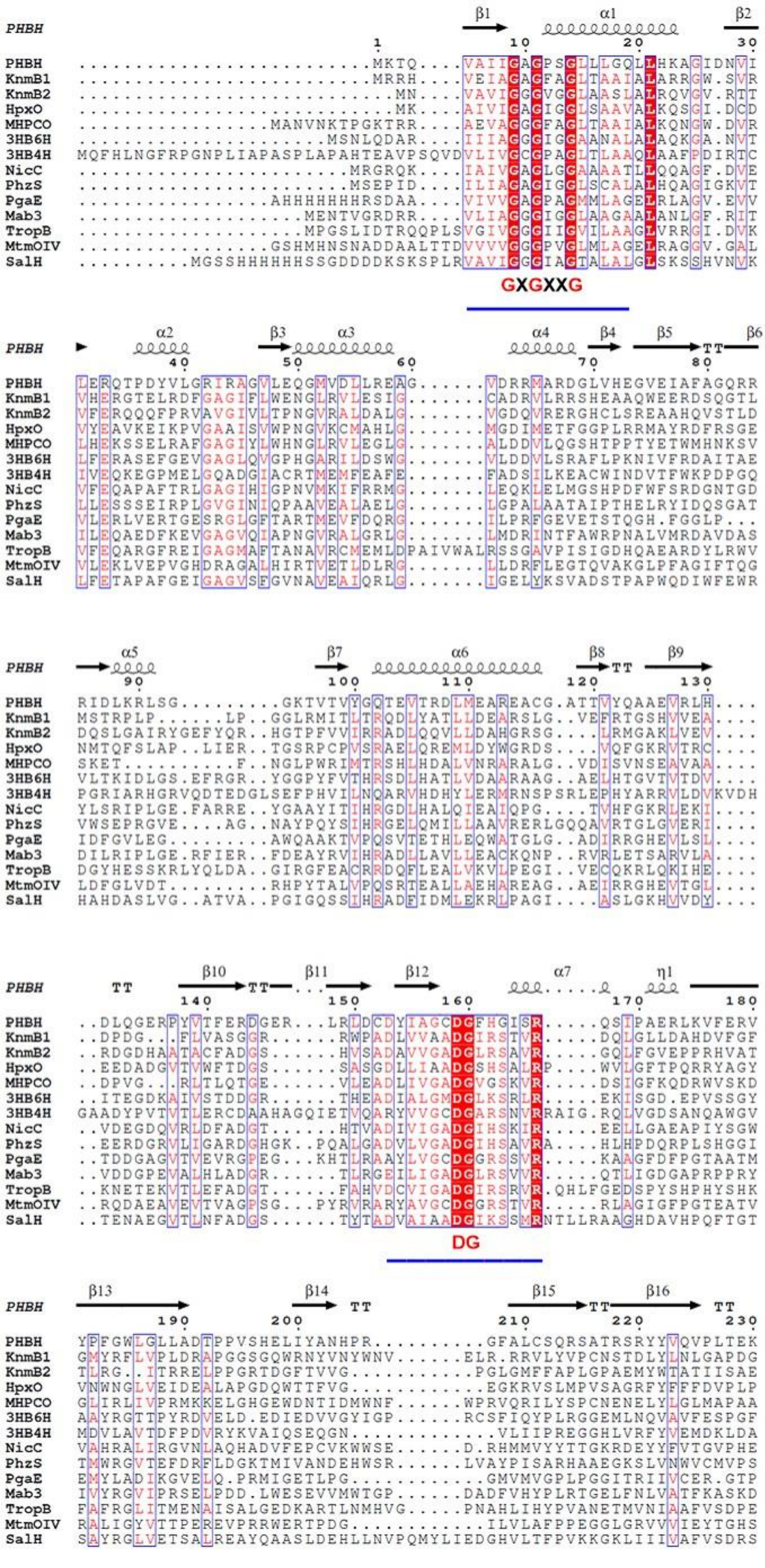

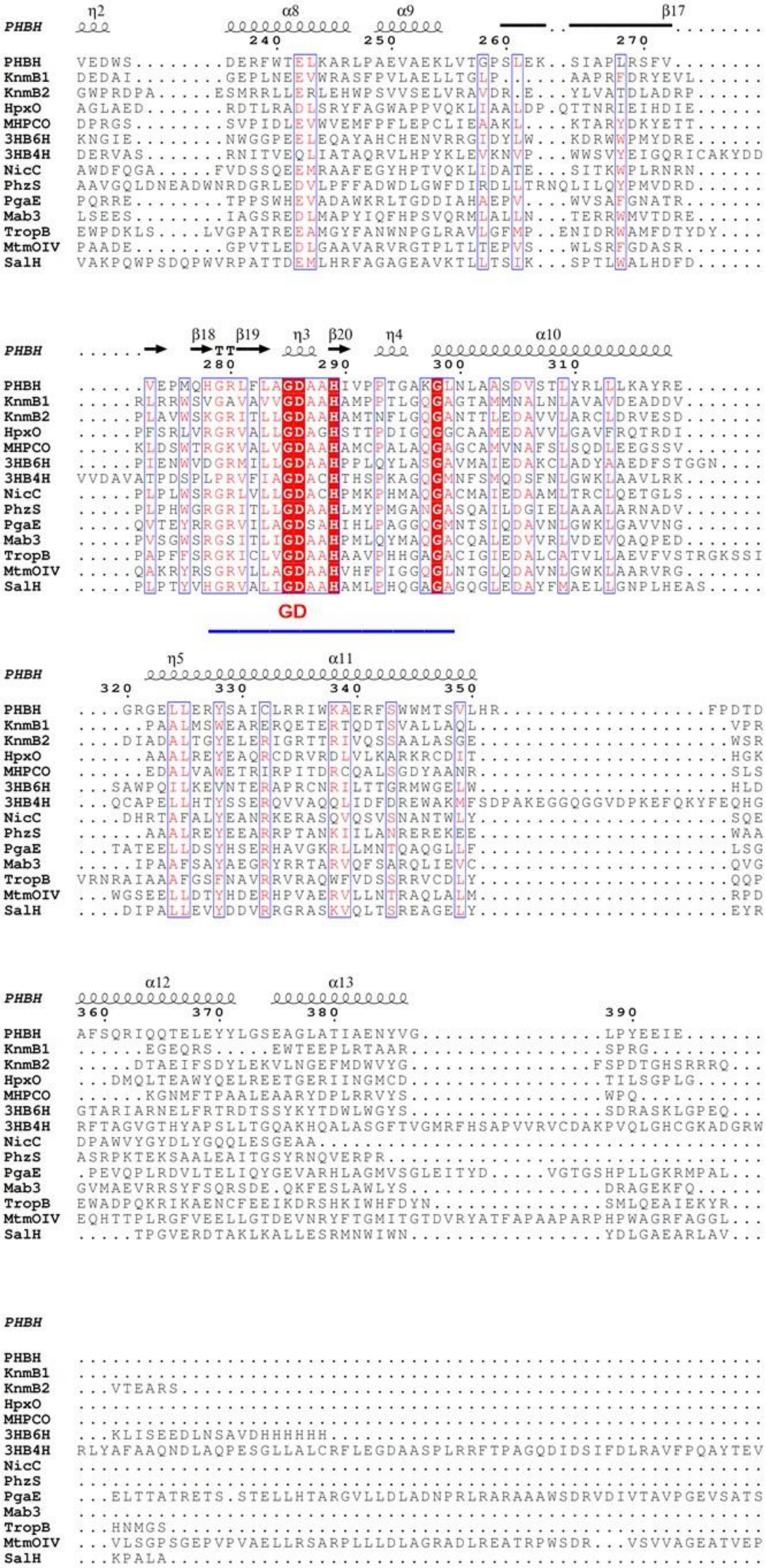
PHBH
η2
α8
α9
β17
240
250
260
270
PHBH VEDWS........DERFWTELKARLPAEVAEKLVTGPSLEK..SIAPLRSFV........
KnmB1 DEDAI........GEPLNEEVWRASFPVLAELLTGLP.....AAPRFDRYEVL......
KnmB2 GWPRDPA......ESMRRLLERLEHWPSVVSELVRAVDR.E...YLVATDLADRP......
HpxO AGLAED.......RDTLRADLSRYFAGWAPPVQKLIAALDP.QTTNRIEIHDIE......
MHPCO DPRGS........SVPIDLEVWVEMFPFLEPCLIEAAKL....KTARYDKYETT......
3HB6H KNGIE........NWGGPEELEQAYAHCHENVRRGIDYLW...KDRWWPMYDRE......
3HB4H DERVAS.......RNITVEQLIATAQRVLHPYKLEVKNVP...WWSVYEIGQRICAKYDD
NicC AWDFQGA.....FVDSSQEEMRAAFEGYHPTVQKLIDATE...SITKWPLRNRN......
PhzS AAVGQLDNEADWNRDGRLEDVLPFFADWDLGWFDIRDLLTRNQLILQYPMVDRD......
PgaE PQRRE........TPPSWHEVADAWKRLTGDDIAHAEPV....WVSAFGNATR.......
Mab3 LSEES........IAGSREDLMAPYIQFHPSVQRMLALLN...TERRWMVTDRE......
TropB EWPDKLS......LVGPATREEAMGYFANWNPGLRAVLGFMP..ENIDRWAMFDTYDY....
MtmOIV PAADE........GPVTLEDLGAAVARVRGTPTLTLTEPVS...WLSRFGDASR.......
SalH VAKPQWPSDQPWVRPATTDEMLHRFAGAGEAVKTLLTSIK...SPTLWALHDFD......
PHBH
β18
β19
η3
β20
η4
α10
TT
280
290
300
310
PHBH ......VEPMQHGRLFLAGDAAHIVPPTGAKGLNLAASDVSTLYRLLLKAYRE.......
KnmB1 .....RLRRWSVGAVAVVGDAAHAMPPTLGQGAGTAMMNALNLAVAVDEADDV.......
KnmB2 .....PLAVWSKGRITLLGDAAHAMTNFLGQGANTTLEDAVVLARCLDRVESD.......
HpxO .....PFSRLVRGRVALLGDAGHSTTPDIGQGGCAAMEDAVVLGAVFRQTRDI.......
MHPCO .....KLDSWTRGKVALVGDAAHAMCPALAQGAGCAMVNAFSLSQDLEEGSSV.......
3HB6H .....PIENWVDGRMILLGDAAHPPLQYLASGAVMAIEDAKCLADYAAEDFSTGGN....
3HB4H VVDAVATPDSPLPRVFIAGDACHTHSPKAGQGMNFSMQDSFNLGWKLAAVLRK.......
NicC .....PLPLWSRGRLVLLGDACHPMKPHMAQGACMAIEDAAMLTRCLQETGLS.......
PhzS .....PLPHWGRGRITLLGDAAHLMYPMGANGASQAILDGIELAAALARNADV.......
PgaE .....QVTEYRRGRVILAGDSAHIHLPAGGQGMNTSIQDAVNLGWKLGAVVNG.......
Mab3 .....PVSGWSRGSITLIGDAAHPMLQYMAQGACQALEDVVRLVDEVQAQPED.......
TropB .....PAPFFSRGKICLVGDAAHAAVPHHGAGACIGIEDALCATVLLAEVFVSTRGKSSI
MtmOIV .....QAKRYRSGRVLLAGDAAHVHFPIGGQGLNTGLQDAVNLGWKLAARVRG.......
SalH .....PLPTYVHGRVALIGDAAHAMLPHQGAGAGQGLEDAYFMAELLGNPLHEAS.....
GD
PHBH
η5
α11
320
330
340
350
PHBH ....GRGELLERYSAICLRRIWKAERFSWWMTSVLHR..................FPDTD
KnmB1 ......PAALMSWEARERQETERTQDTSVALLAQL.....................VPR
KnmB2 ....DIADALTGYELERIGRTTRIVQSSAALASGE.....................WSR
HpxO ......AAALREYEAQRCDRVRDLVLKARKRCDIT.....................HGK
MHPCO ......EDALVAWETRIRPITDRCQALSGDYAANR.....................SLS
3HB6H ....SAWPQILKEVNTERAPRCNRILTTGRMWGELW....................HLD
3HB4H ...QCAPELLHTYSSERQVVAQQLIDFDREWAKMFSDPAKEGGQGGVDPKEFQKYFEQHG
NicC ....DHRTAFALYEANRKERASQVQSVSNANTWLY.....................SQE
PhzS ......AAALREYEEARRPTANKIILANREREKEE.....................WAA
PgaE ...TATEELLDSYHSERHAVGKRLLMNTQAQGLLF.....................LSG
Mab3 .....IPAAFSAYAEGRYRRTARVQFSARQLIEVC.....................QVG
TropB VRNRAIAAAFGSFNAVRRVRAQWFVDSSRRVCDLY.....................QQP
MtmOIV ...WGSEELLDTYHDERHPVAERVLLNTRAQLALM.....................RPD
SalH ....DIPALLEVYDDVRRGRASKVQLTSREAGELY.....................EYR
PHBH
α12
α13
360
370
380
390
PHBH AFSQRIQQTELEYYLGSEAGLATIAENYVG................LPYEEIE.......
KnmB1 ......EGEQRS.....EWTEEPLRTAAR.................SPRG.........
KnmB2 .....DTAEIFSDYLEKVLNGEFMDWVYG................FSPDTGHSRRRQ...
HpxO ...DMQLTEAWYQELREETGERIINGMCD................TILSGPLG......
MHPCO ......KGNMFTPAALEAARYDPLRRVYS................WPQ..........
3HB6H GTARIARNELFRTRDTSSYKYTDWLWGYS................SDRASKLGPEQ...
3HB4H RFTAGVGTHYAPSLLTGQAKHQALASGFTVGMRFHSAPVVRVCDAKPVQLGHCGKADGRW
NicC DPAWVYGYDLYGQQLESGEAA.......................................
PhzS ASRPKTEKSAALEAITGSYRNQVERPR.................................
PgaE .PEVQPLRDVLTELIQYGEVARHLAGMVSGLEITYD.....VGTGSHPLLGKRMPAL...
Mab3 GVMAEVRRSYFSQRSDE.QKFESLAWLYS................DRAGEKFQ.......
TropB EWADPQKRIKAENCFEEIKDRSHKIWHFDYN..............SMLQEAIEKYR....
MtmOIV EQHTTPLRGFVEELLGTDEVNRYFTGMITGTDVRYATFAPAAPARPHPWAGRFAGGL...
SalH .....TPGVERDTAKLKALLESRMNWIWN................YDLGAEARLAV...
PHBH
PHBH ....
KnmB1 ....
KnmB2 ...VTEARS...
HpxO ....
MHPCO ....
3HB6H ...KLISEEDLNSAVDHHHHHH......................................
3HB4H RLYAFAAQNDLAQPESGLLALCRFLEGDAASPLRRFTPAGQDIDSIFDLRAVFPQAYTEV
NicC ....
PhzS ....
PgaE ...ELTTATRETS.STELLHTARGVLLDLADNPRLRARAAAWSDRVDIVTAVPGEVSATS
Mab3 ....
TropB ...HNMGS..
MtmOIV ...VLSGPSGEPVPVAELLRSARPLLLDLAGRADLREATRPWSDR...VSVVAGEATVEP
SalH ...KPALA..

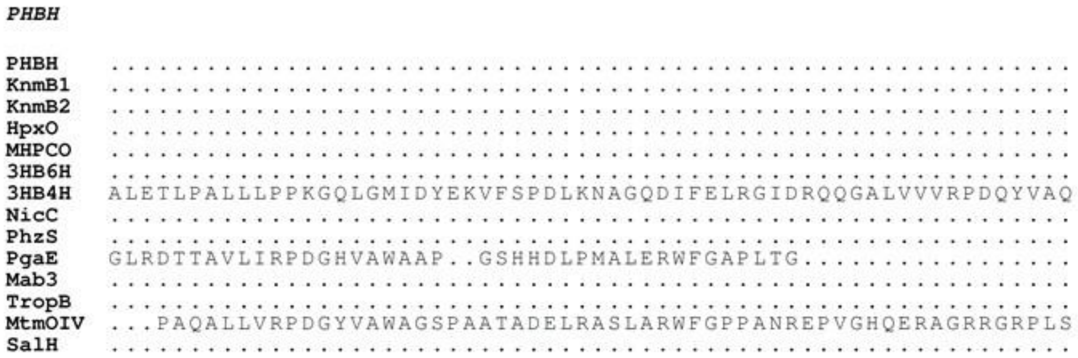

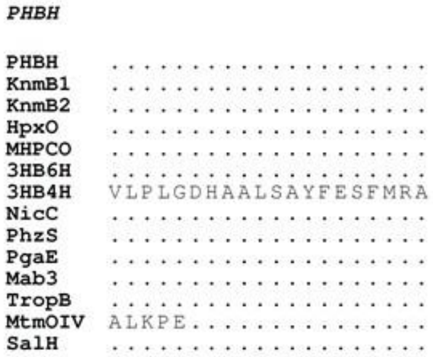

Supplementary Figure 62. Structure-based sequence alignment of KnmB1, KnmB2 and the other group A FPMOs. Identical residues are shown in red. FAD fingerprints are underlined in blue. Secondary structure assigned from the PHBH crystal structure (PDB: 1pbe) is indicated above the sequences. Uric acid monooxygenase (HpxO, PDB: 3rp6); 2-methyl-3-hydroxypyridine-5-carboxylic acid oxygenase (MHPCO, PDB: 3gmc); 3-hydroxybenzoate 6-hydroxylase (3HB6H, PDB: 4bk1); 3-hydroxybenzoate hydroxylase (3HB4H, PDB: 2dkh); 6-hydroxynicotinic acid 3-monooxygenase (NicC, PDB: 5eow); 5-methylphenazine 1-carboxylate monooxygenase (PhzS, PDB: 2rgj); aromatic hydroxylase involved in angucycline biosynthesis (PgaE, PDB: 2qa1); 3-aminobenzoate 6-monooxygenase (Mab3, GenBank: ARD05467); 3-methylorcinaldehyde monooxygenase (TropB, PDB: 6nes); premithramycin B monooxygenase (MtmOIV, PDB: 4k5s); salicylate hydroxylase (SalH, PDB: 5evy).

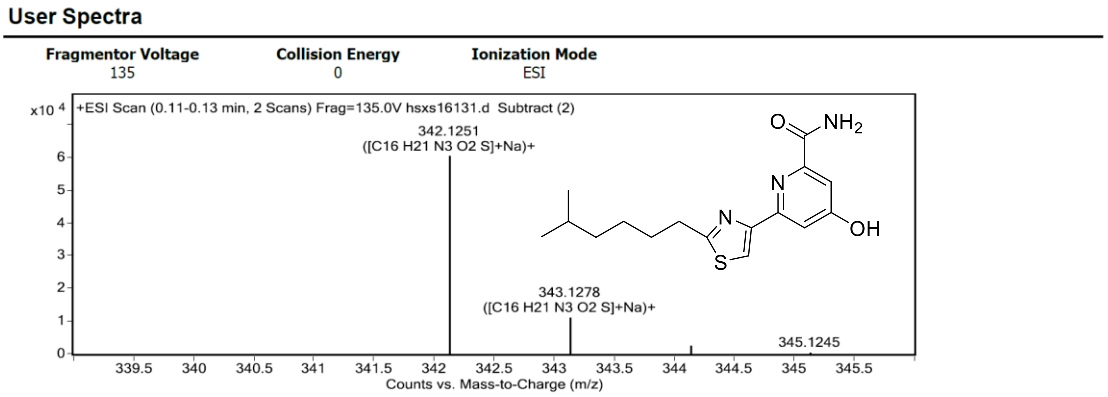

Supplementary Figure 63. HRESIMS spectrum of compound **24**.

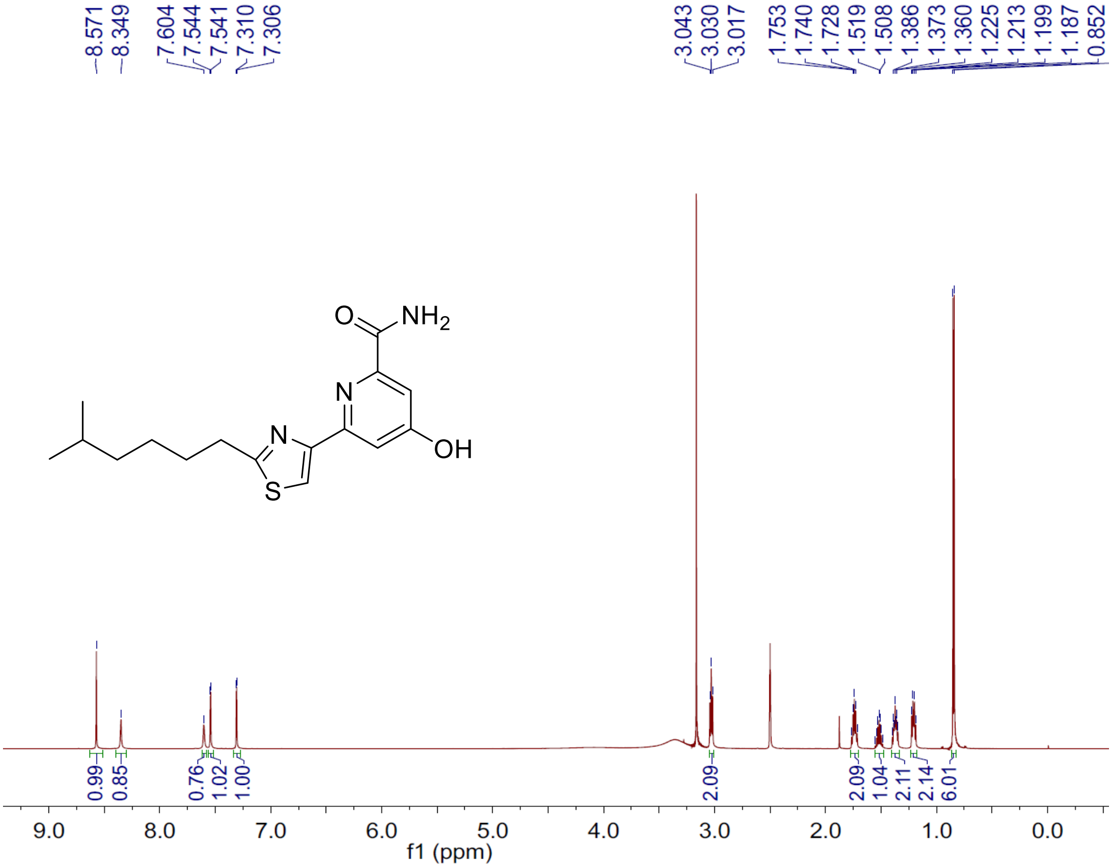

Supplementary Figure 64. ^{1}H-NMR (600 MHz) spectrum of compound **24** in DMSO-*d*$_6$.

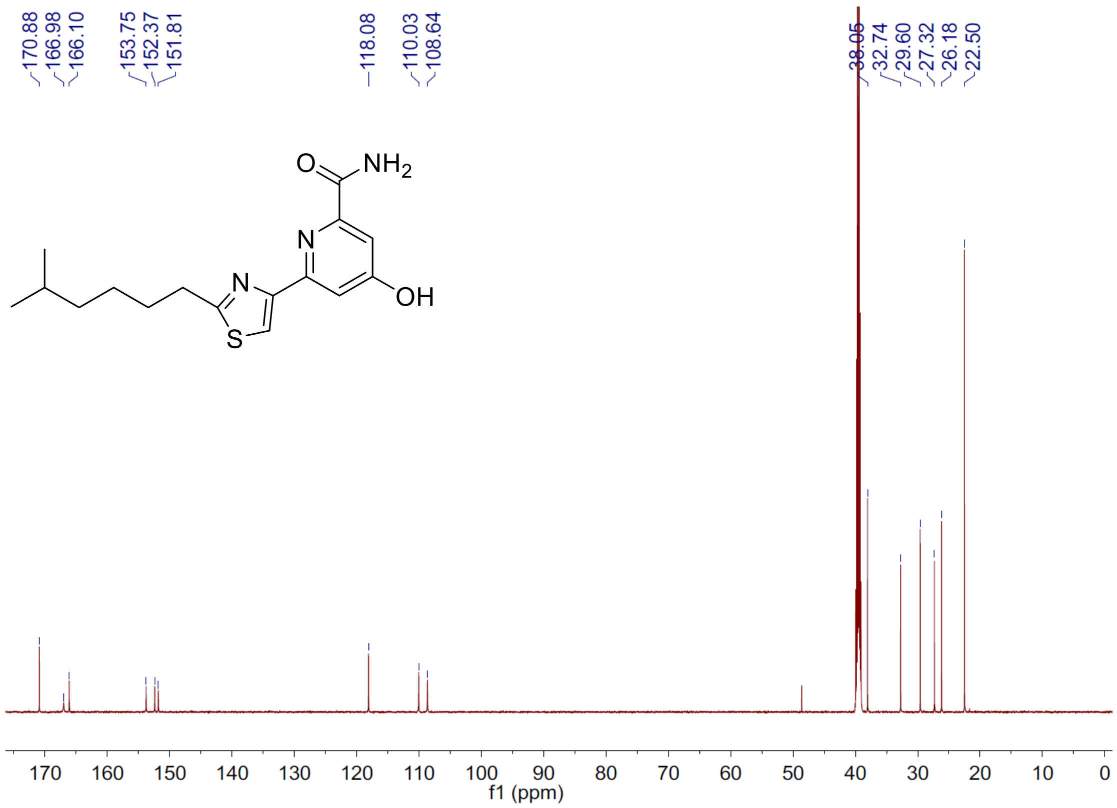

Supplementary Figure 65. ^{13}C-NMR (150 MHz) spectrum of compound **24** in DMSO-*d*$_6$.

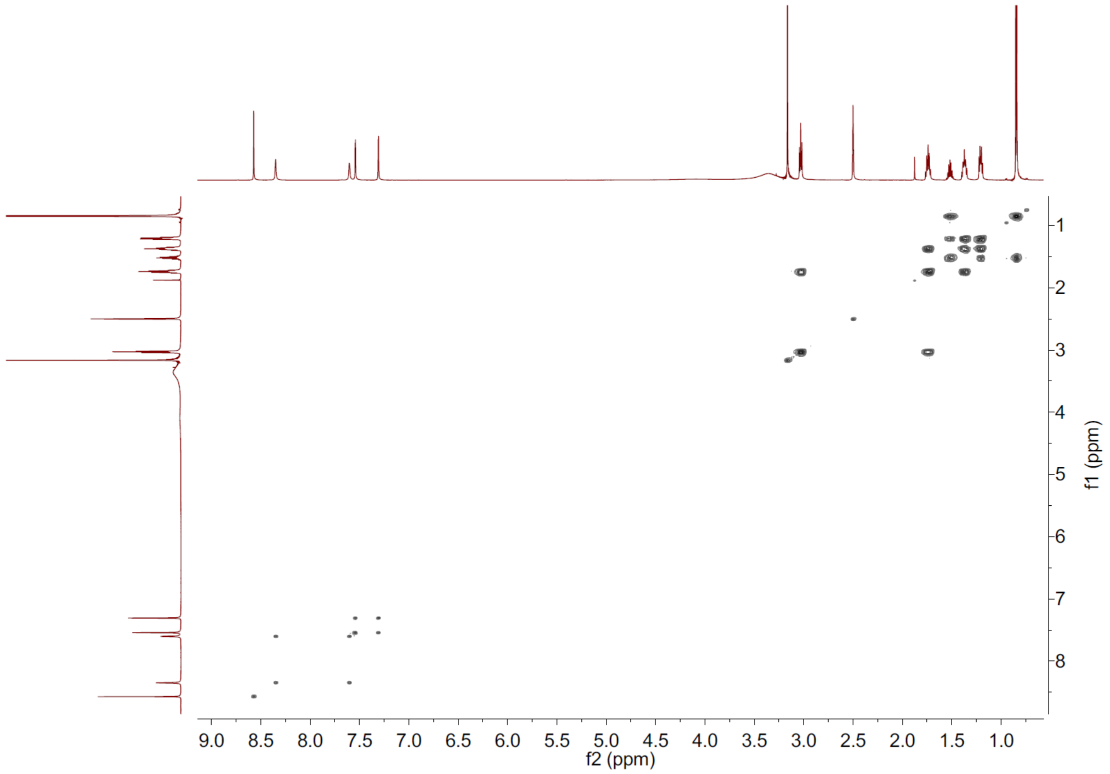

Supplementary Figure 66. ^{1}H-^{1}H COSY (600 MHz) spectrum of compound **24** in DMSO-*d*$_6$.

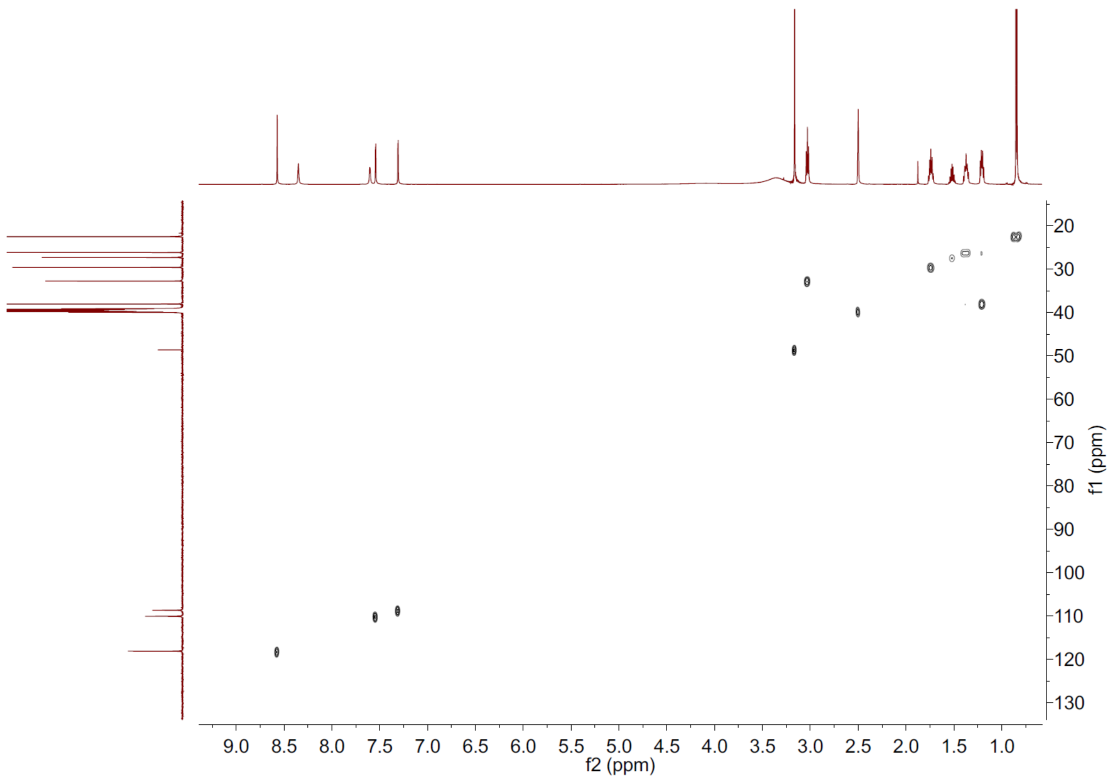

Supplementary Figure 67. HSQC (600 MHz) spectrum of compound **24** in DMSO-d_6.

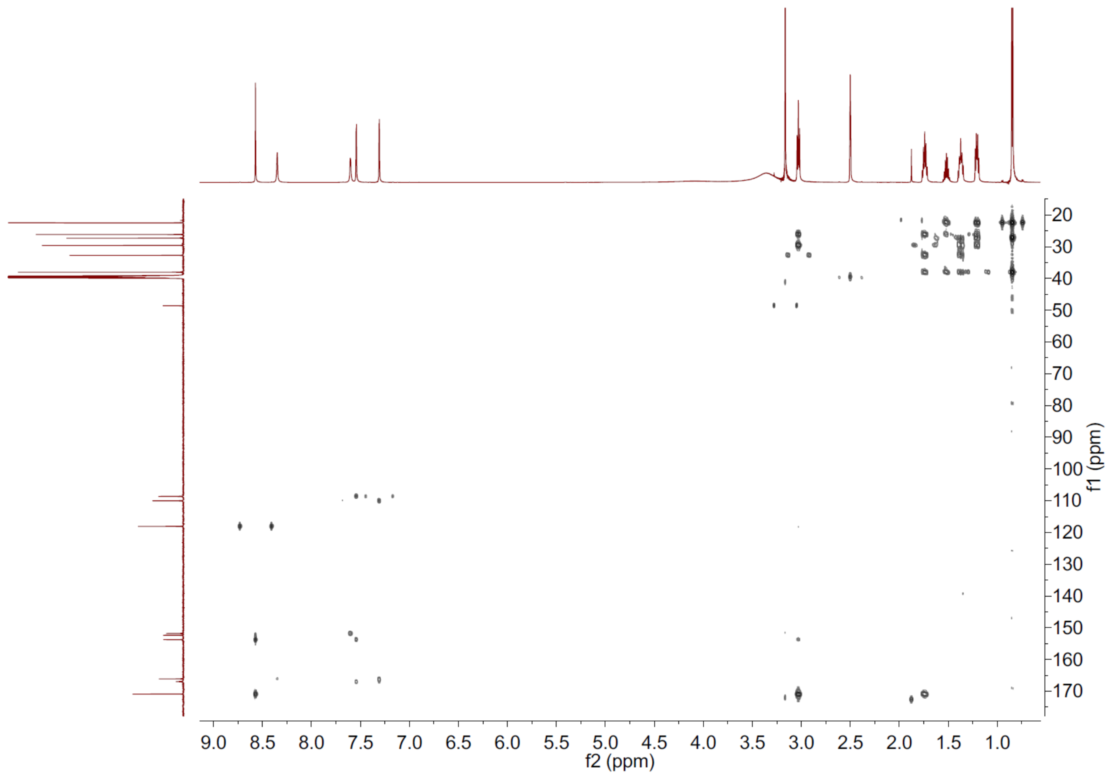

Supplementary Figure 68. HMBC (600 MHz) spectrum of compound **24** in DMSO-d_6.

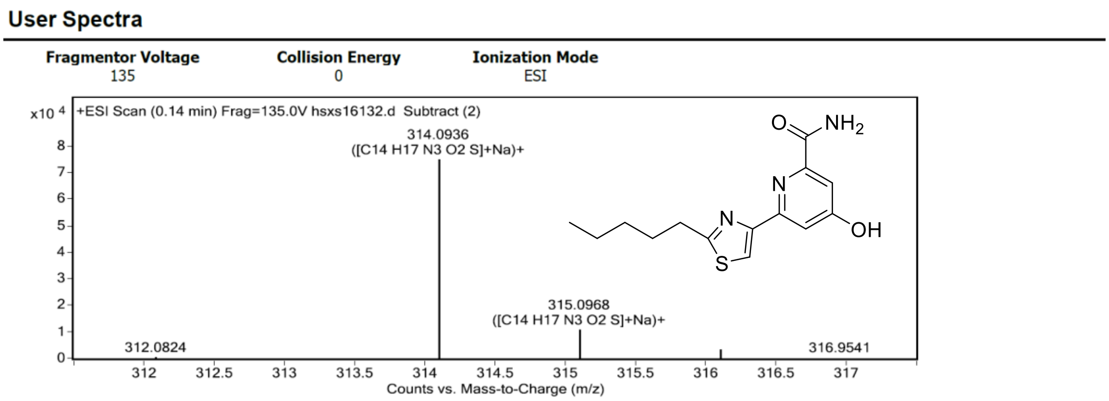

Supplementary Figure 69. HRESIMS spectrum of compound **25**.

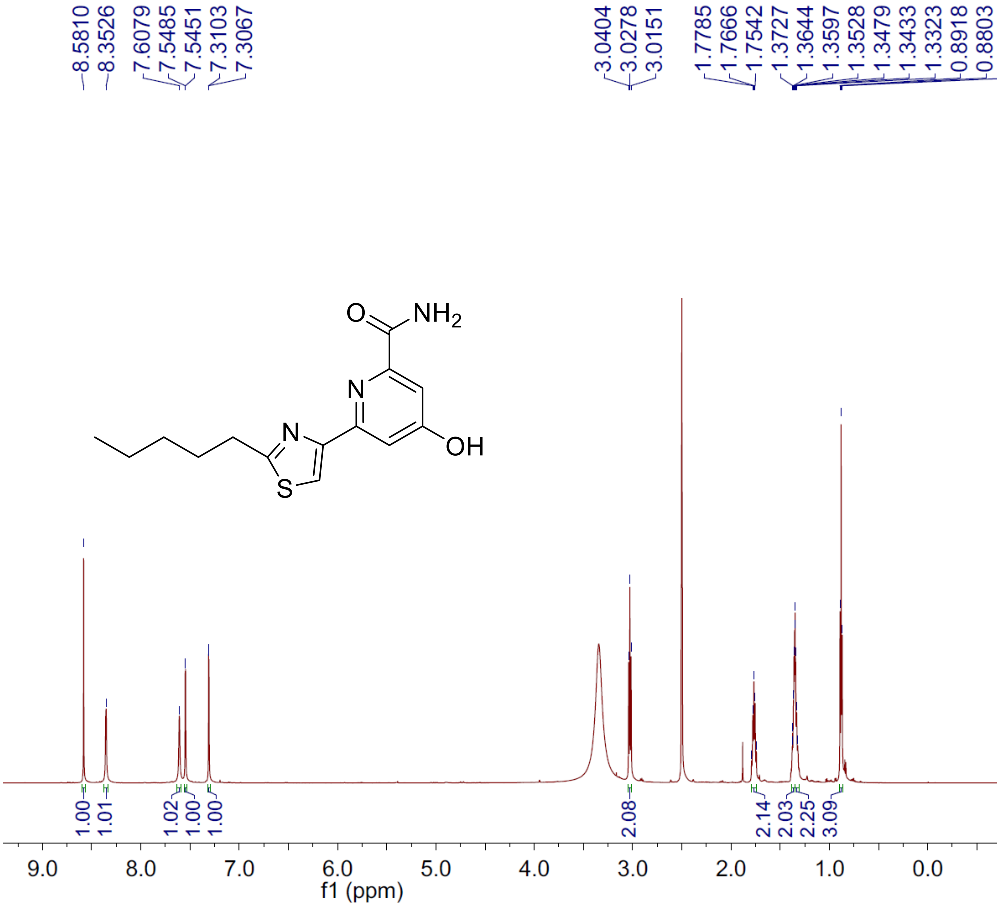

Supplementary Figure 70. ^{1}H-NMR (600 MHz) spectrum of compound **25** in DMSO-d_6.

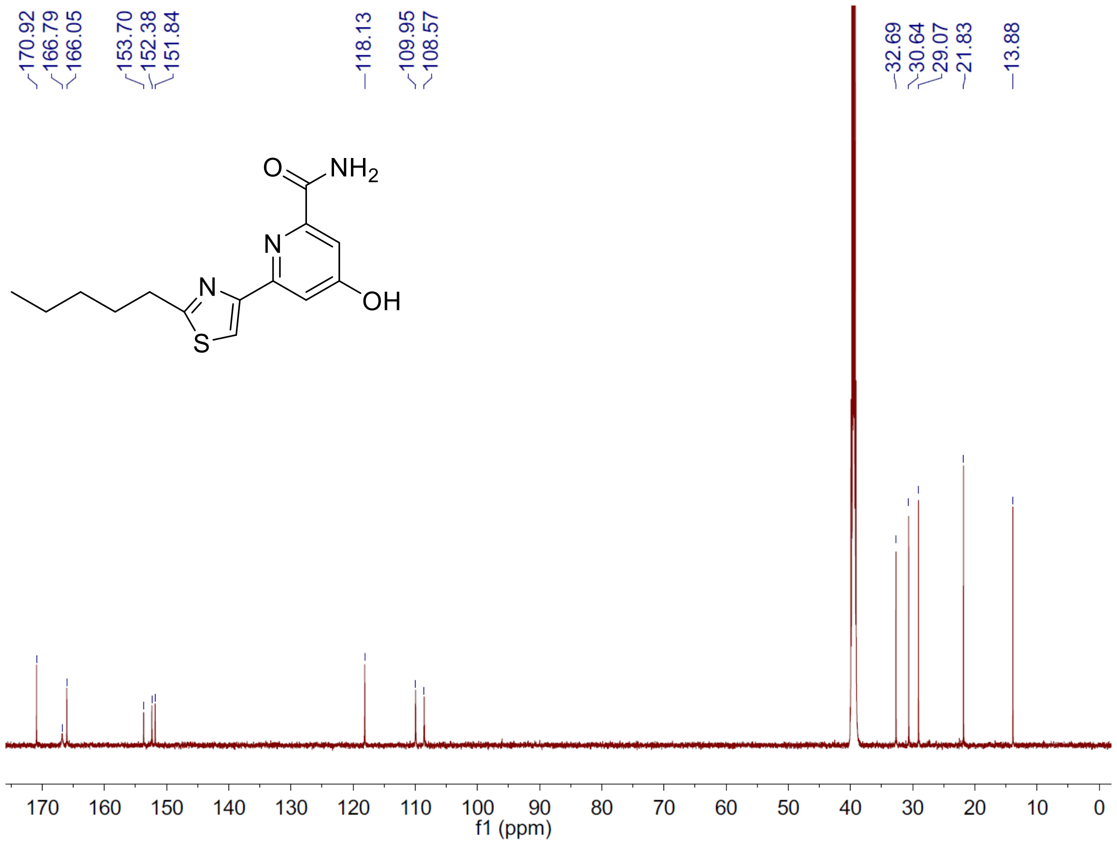

Supplementary Figure 71. ^{13}C-NMR (150 MHz) spectrum of compound **25** in DMSO-d_6.

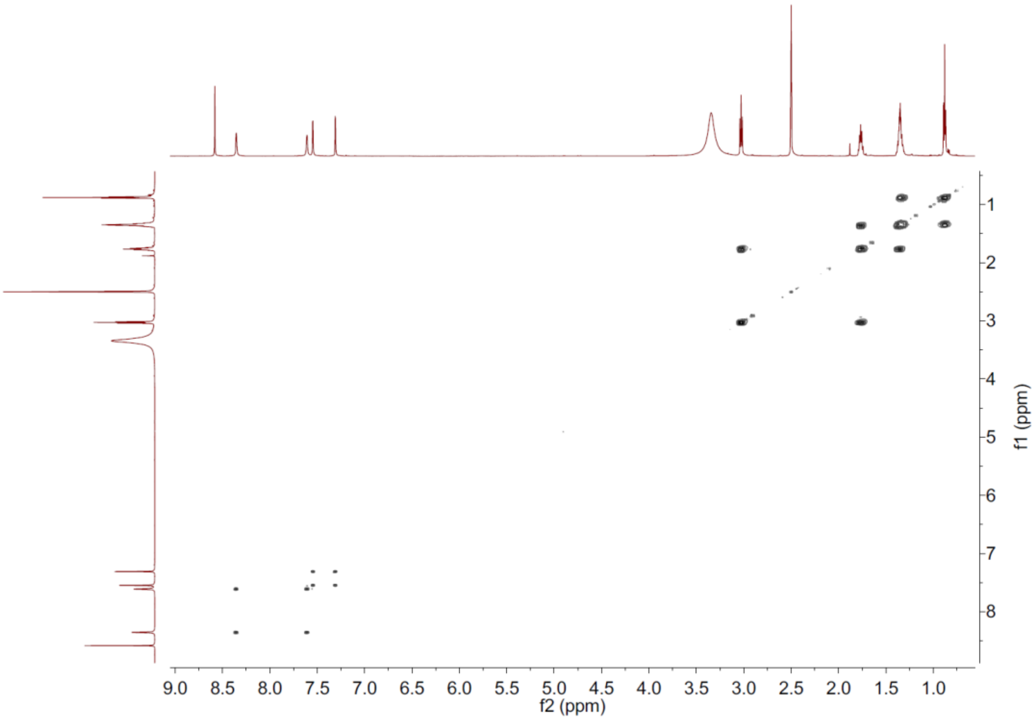

Supplementary Figure 72. ^{1}H-^{1}H COSY (600 MHz) spectrum of compound **25** in DMSO-d_6.

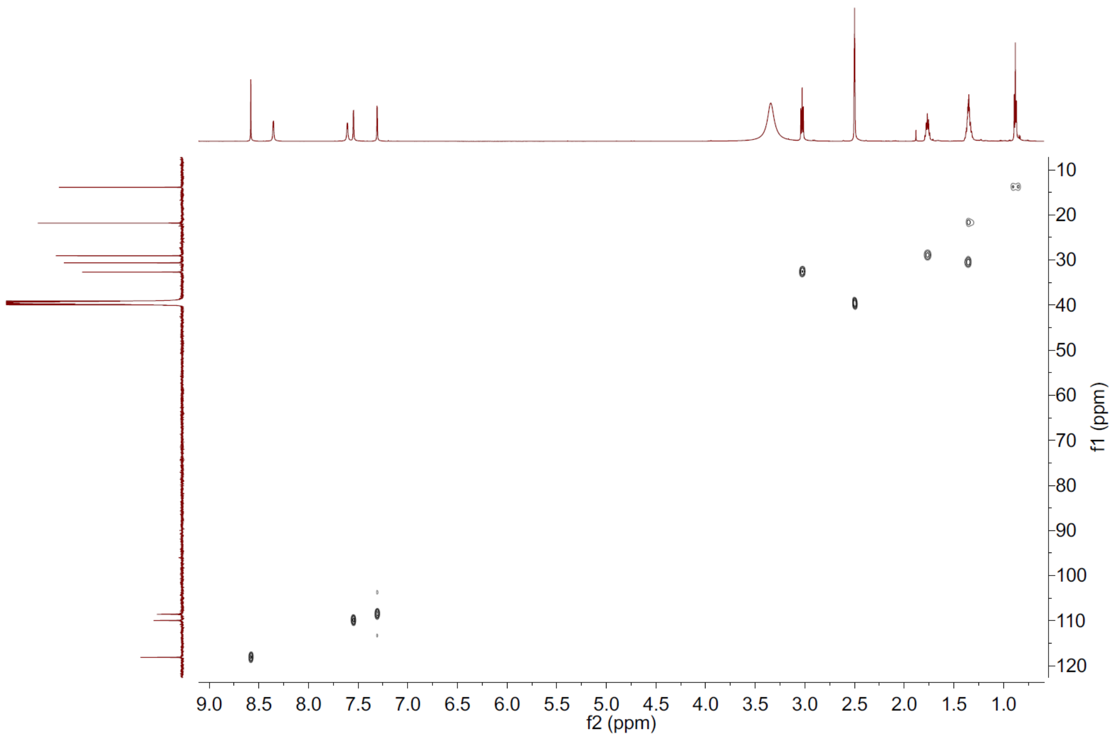

Supplementary Figure 73. HSQC (600 MHz) spectrum of compound **25** in DMSO-d_6.

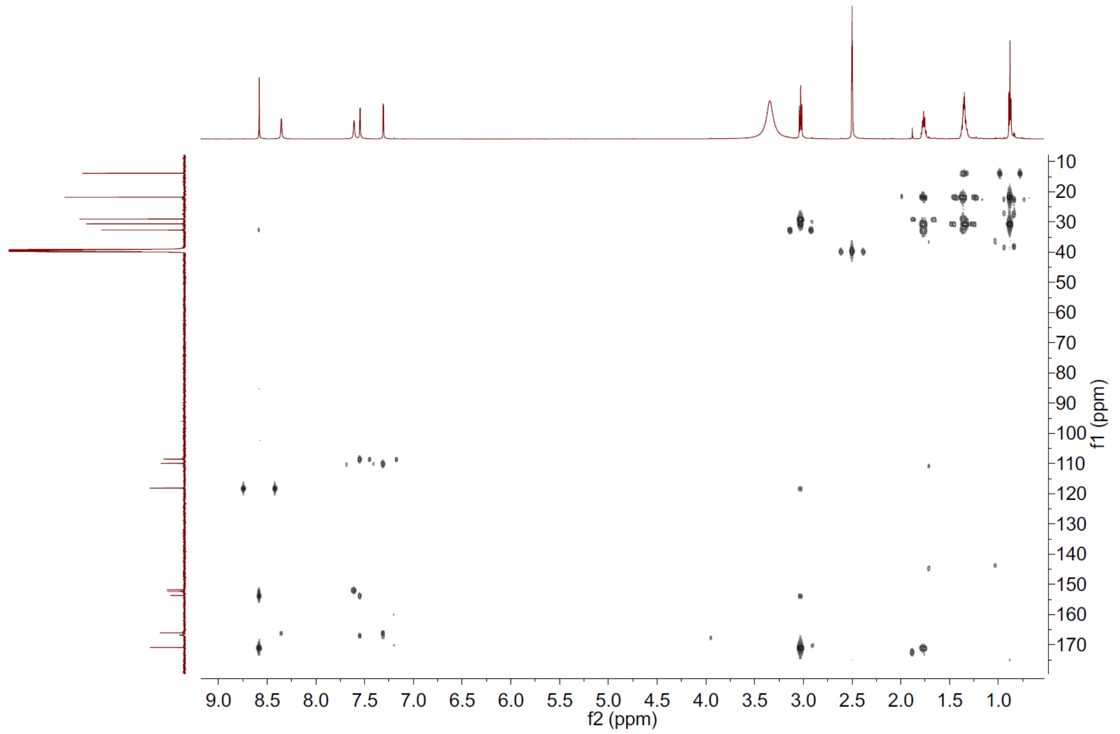

Supplementary Figure 74. HMBC (600 MHz) spectrum of compound **25** in DMSO-d_6.

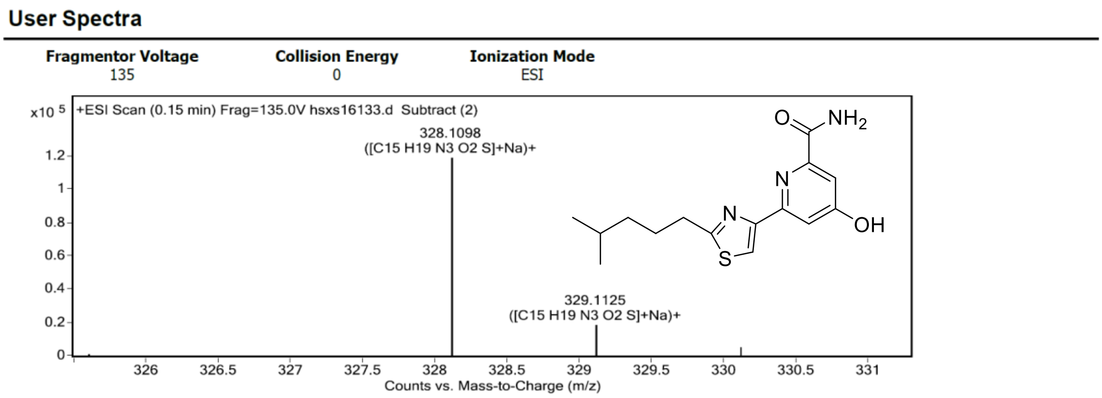

Supplementary Figure 75. HRESIMS spectrum of compound **26**.

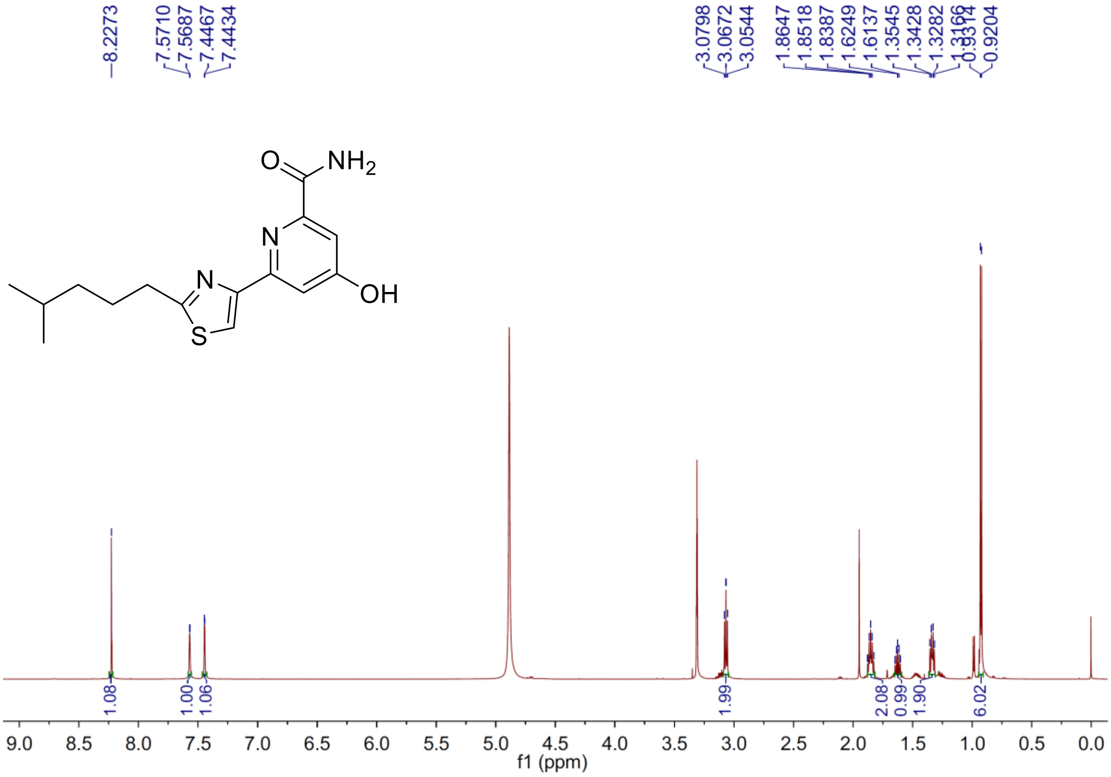

Supplementary Figure 76. ^{1}H-NMR (600 MHz) spectrum of compound **26** in MeOD.

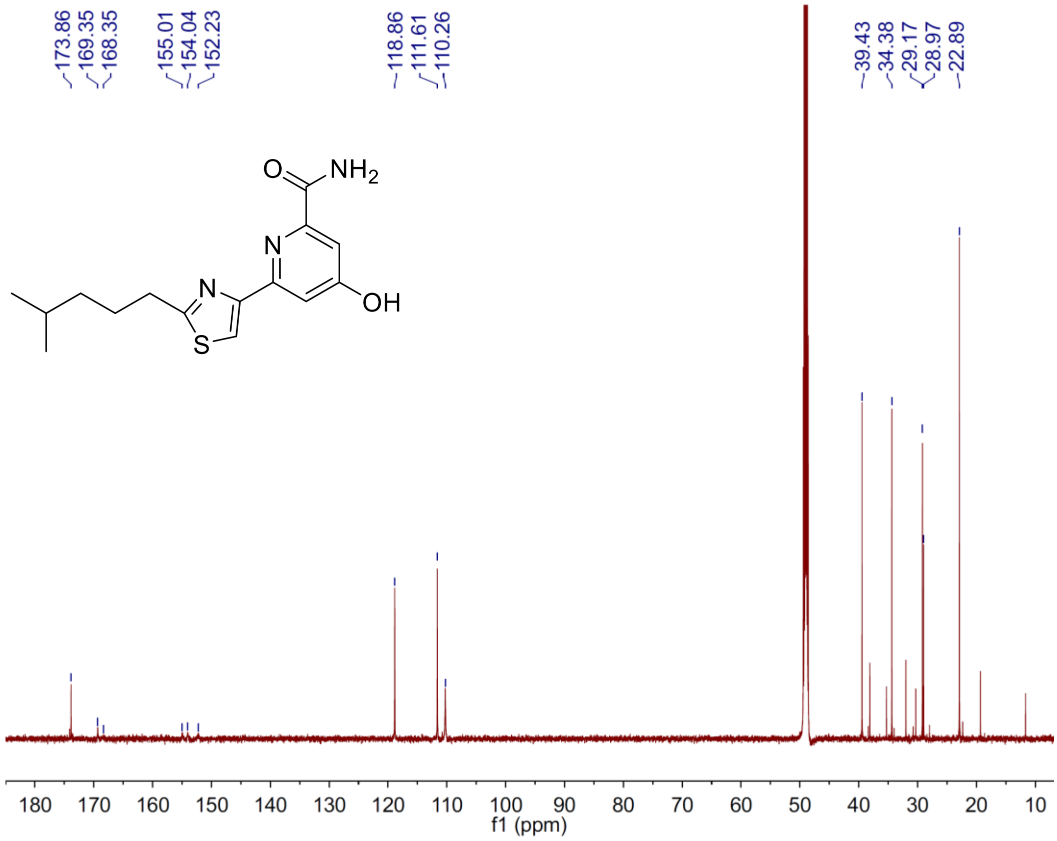

Supplementary Figure 77. ^{13}C-NMR (150 MHz) spectrum of compound **26** in MeOD.

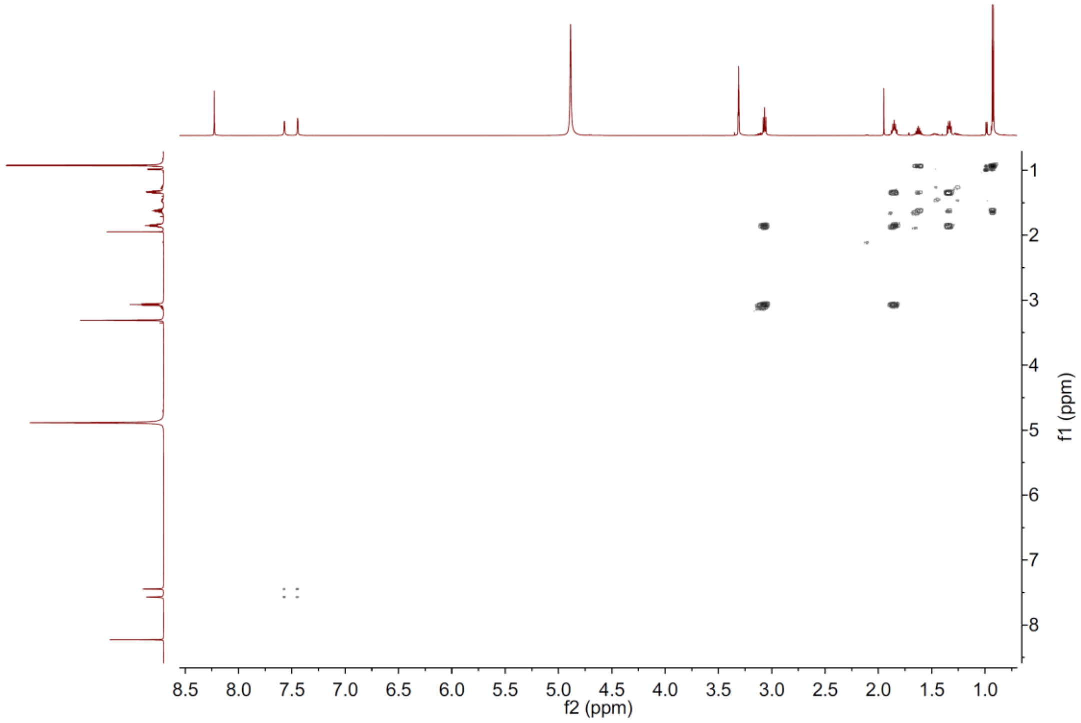

Supplementary Figure 78. ^{1}H-^{1}H COSY (600 MHz) spectrum of compound **26** in MeOD.

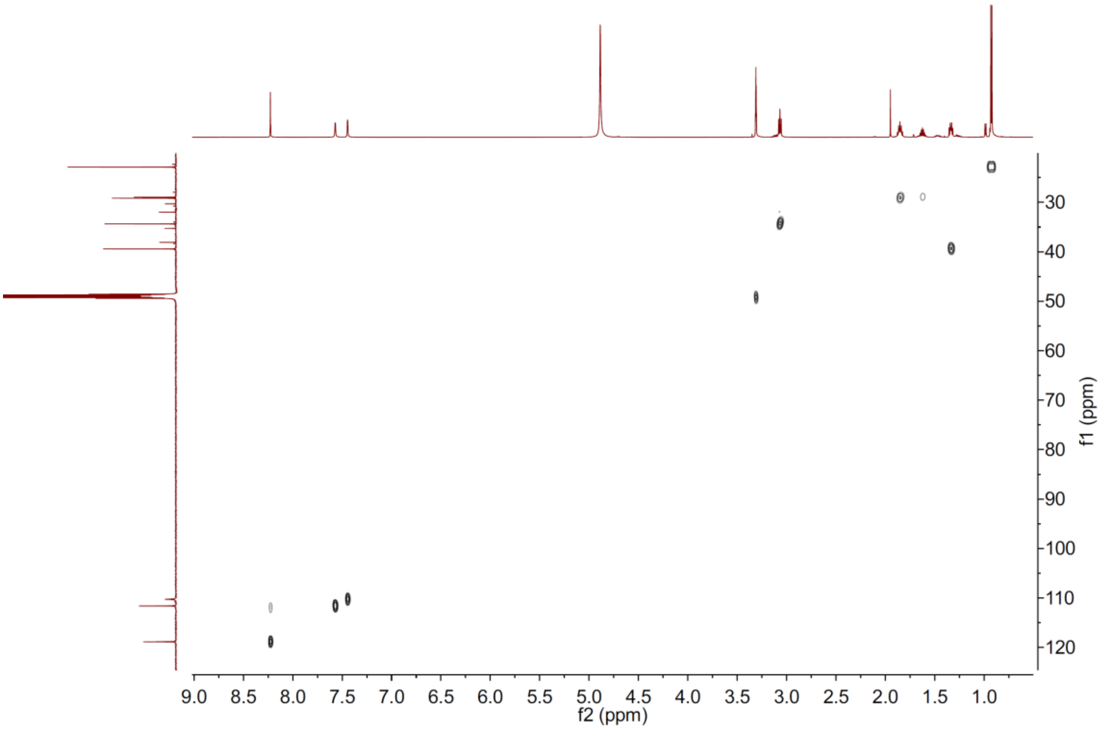

Supplementary Figure 79. HSQC (600 MHz) spectrum of compound **26** in MeOD.

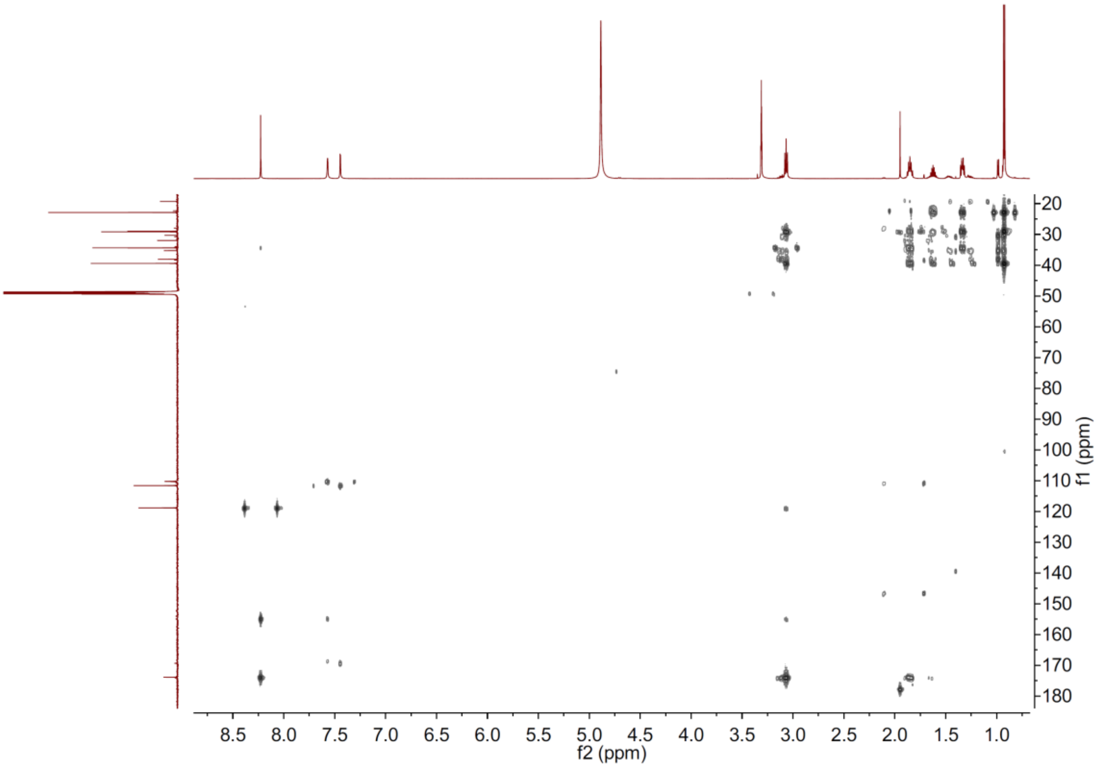

Supplementary Figure 80. HMBC (600 MHz) spectrum of compound **26** in MeOD.

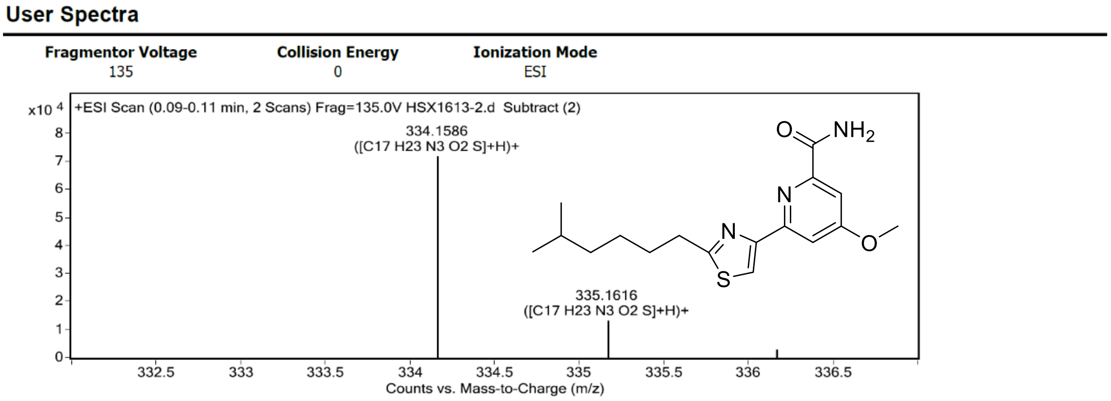

Supplementary Figure 81. HRESIMS spectrum of compound **32**.

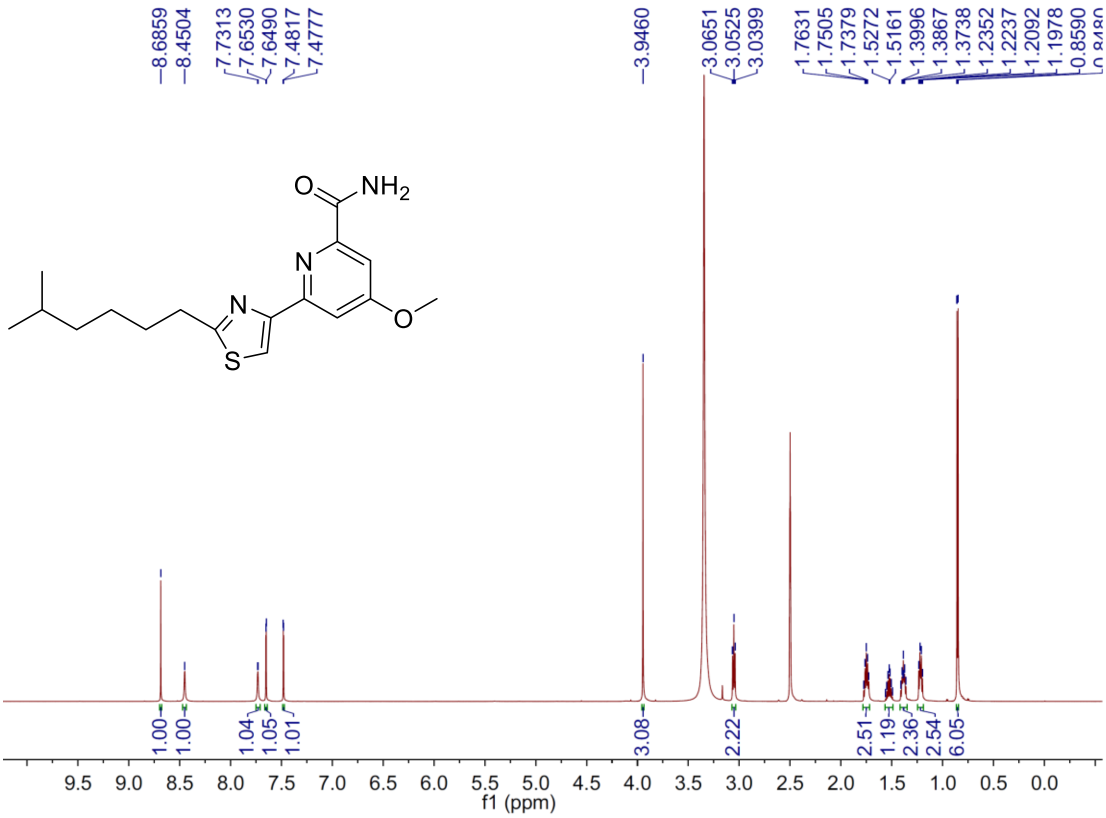

Supplementary Figure 82. ^{1}H-NMR (600 MHz) spectrum of compound **32** in DMSO-*d*$_6$.

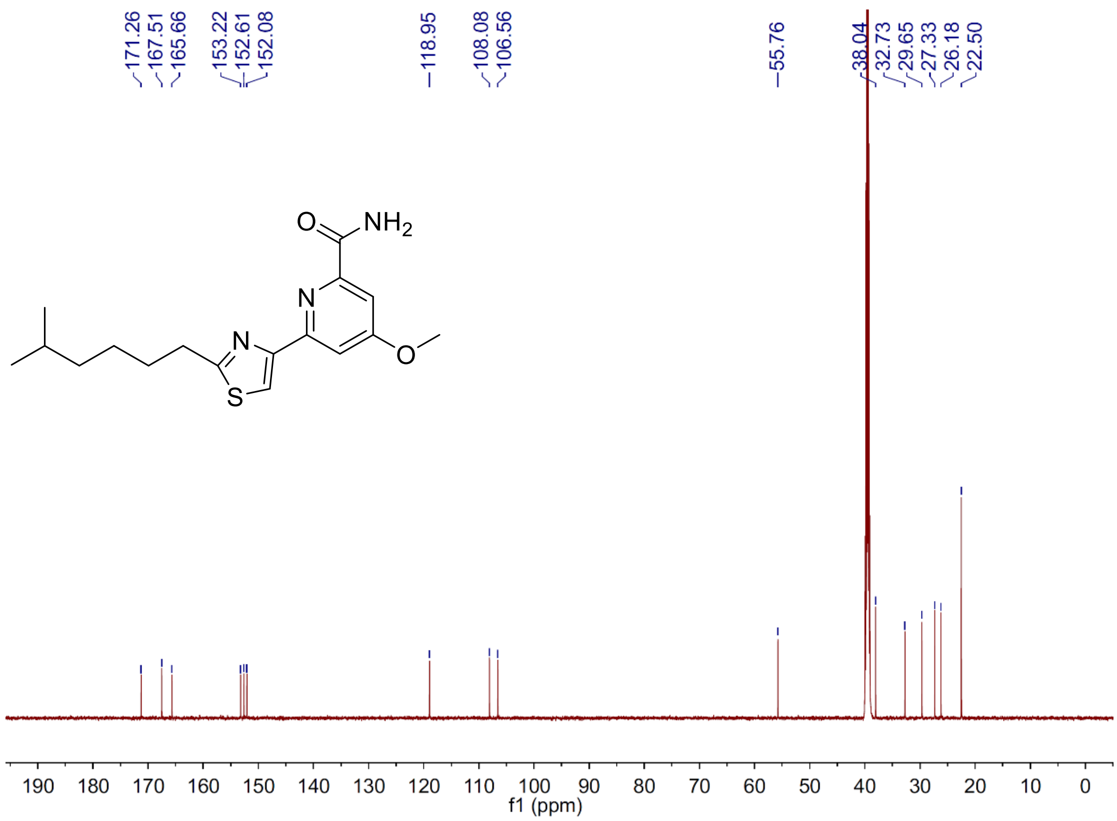

Supplementary Figure 83. ^{13}C-NMR (150 MHz) spectrum of compound **32** in DMSO-d_6.

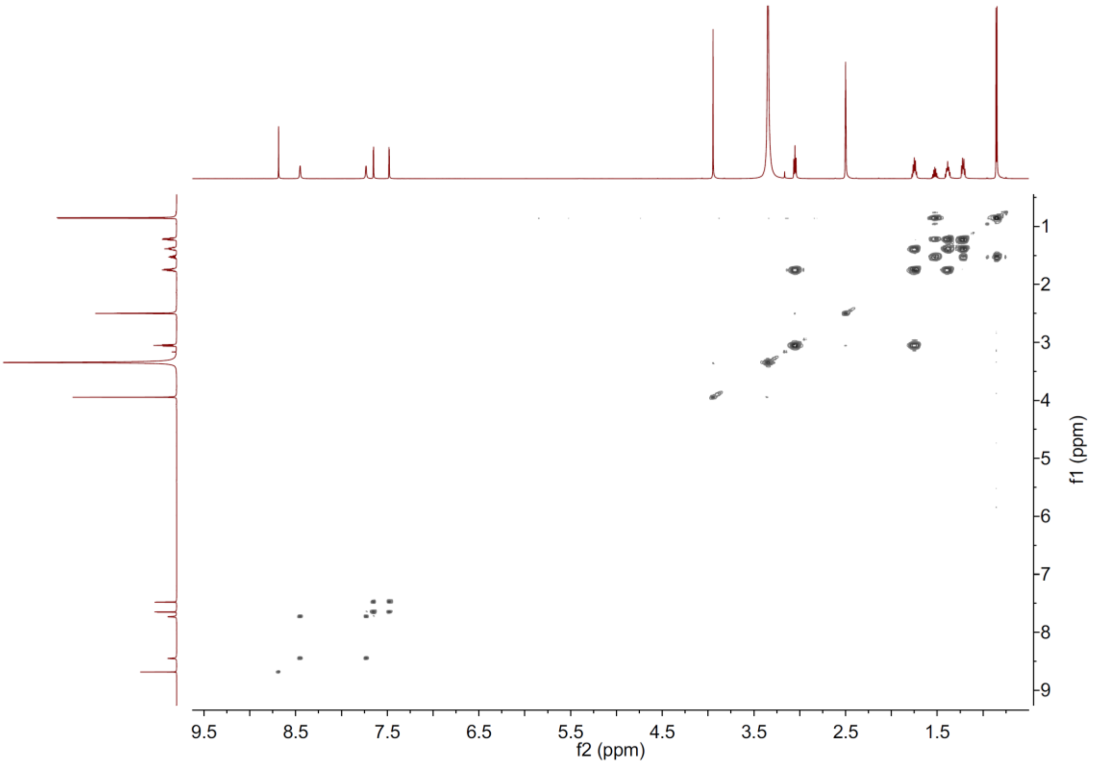

Supplementary Figure 84. ^{1}H-^{1}H COSY (600 MHz) spectrum of compound **32** in DMSO-d_6.

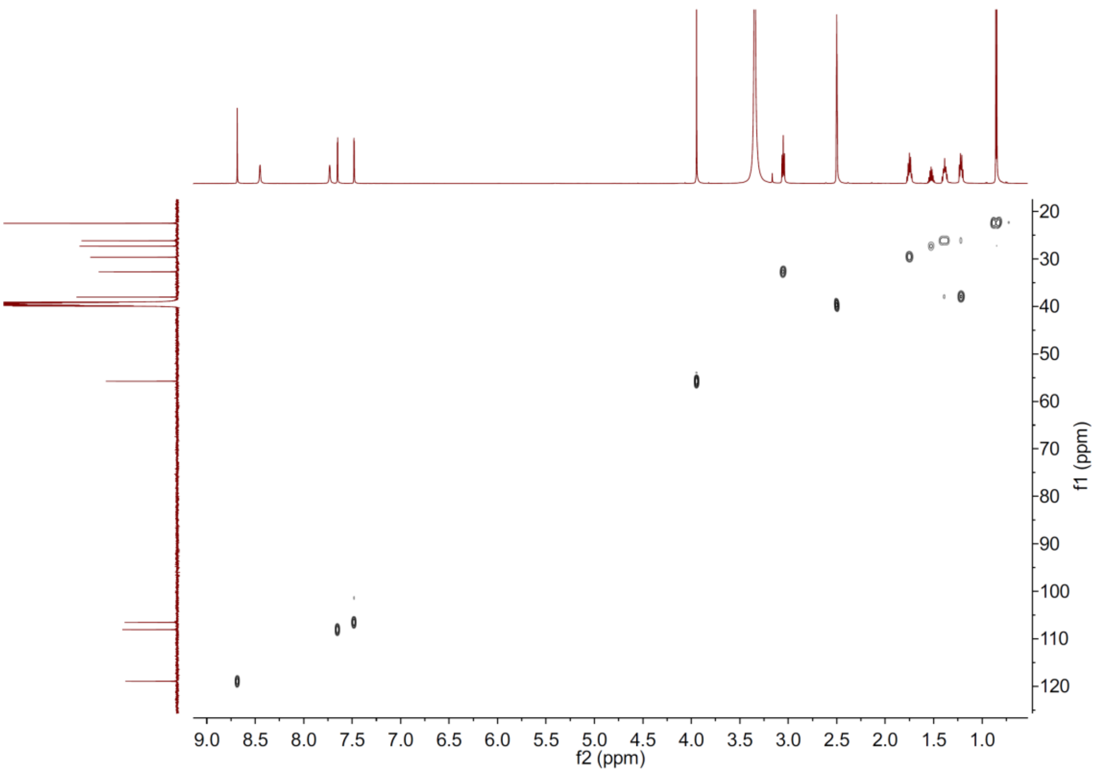

Supplementary Figure 85. HSQC (600 MHz) spectrum of compound **32** in DMSO-d_6.

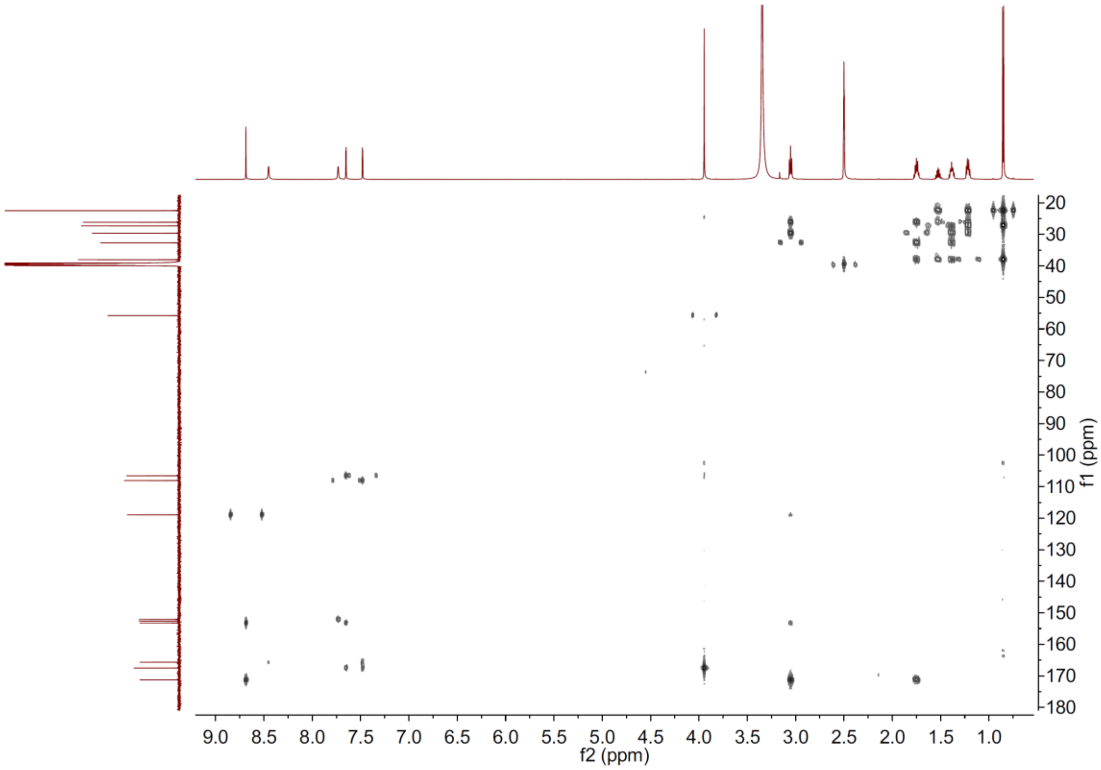

Supplementary Figure 86. HMBC (600 MHz) spectrum of compound **32** in DMSO-d_6.

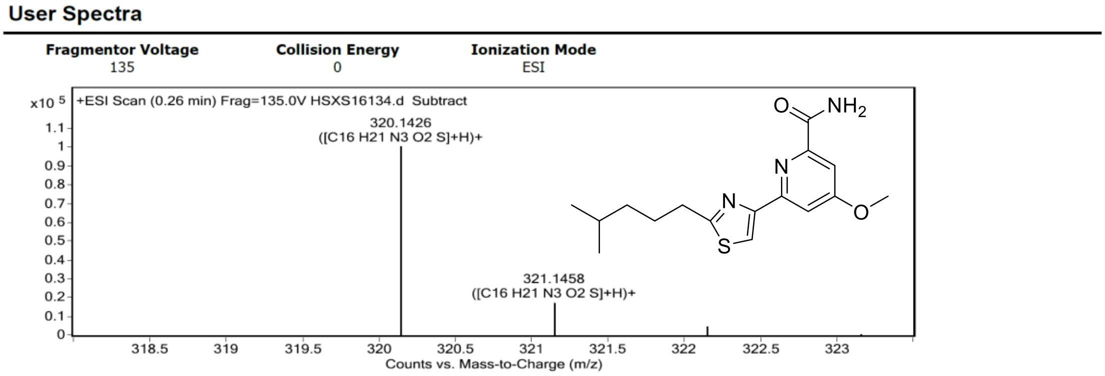

Supplementary Figure 87. HRESIMS spectrum of compound **33**.

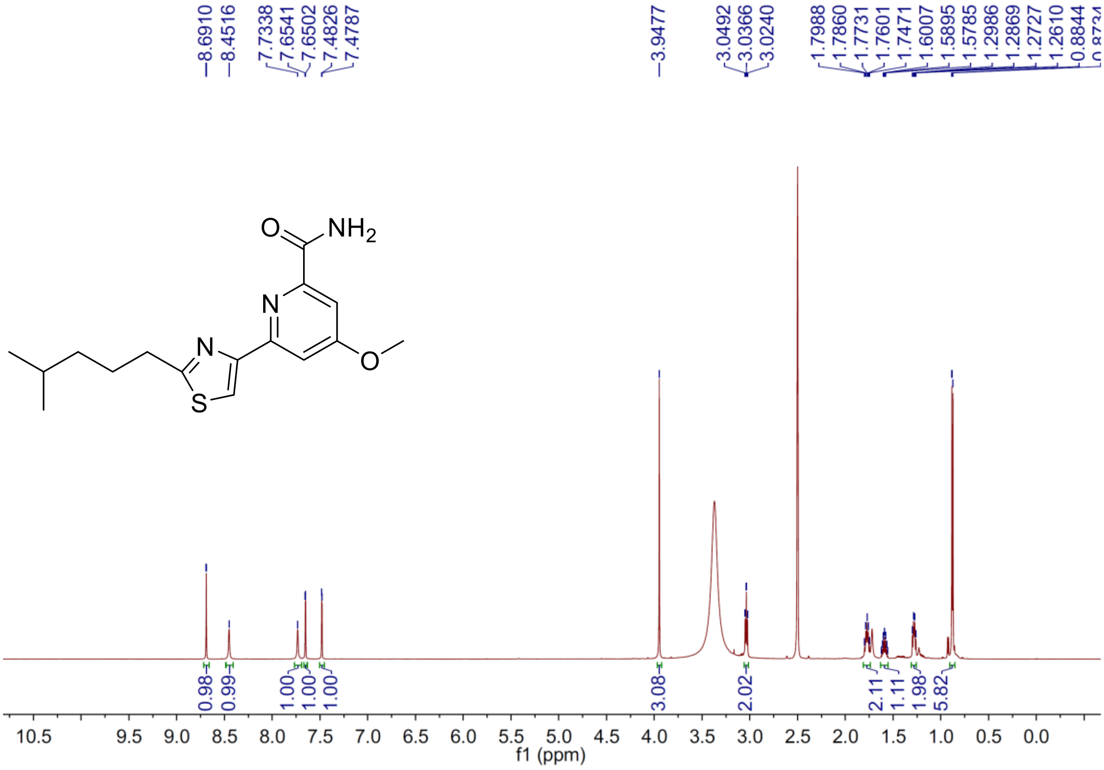

Supplementary Figure 88. ^{1}H-NMR (600 MHz) spectrum of compound **33** in DMSO-*d*$_6$.

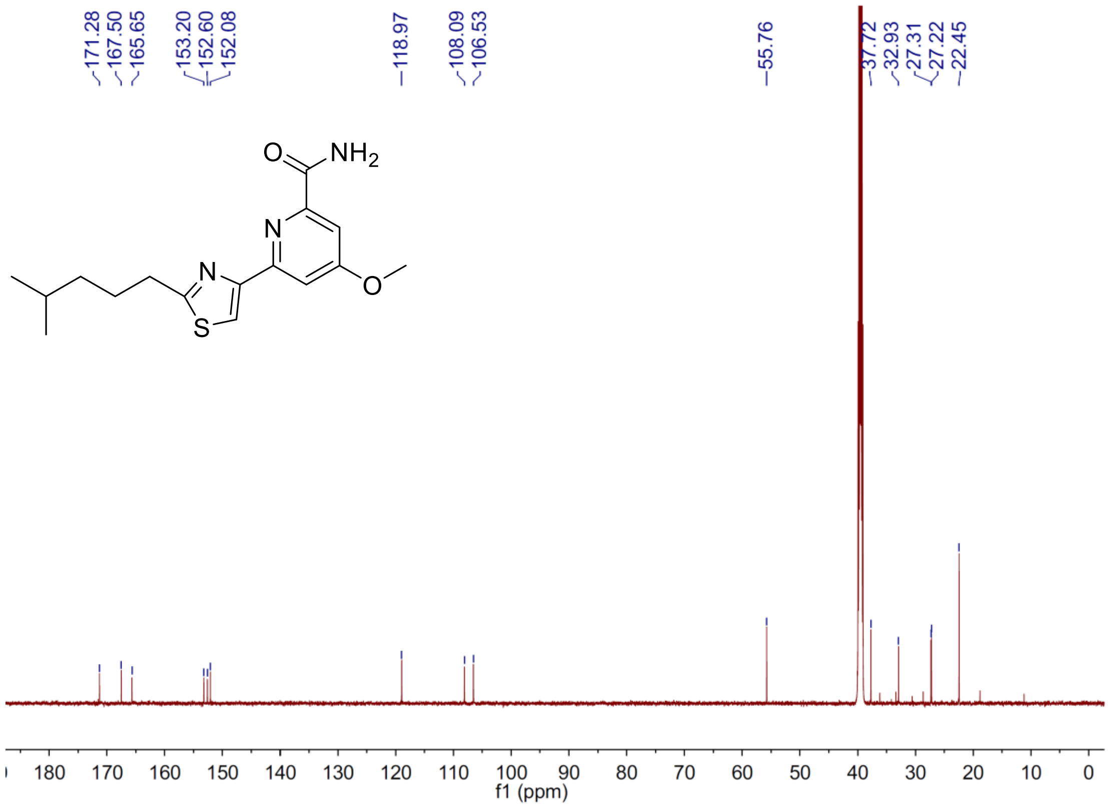

Supplementary Figure 89. ^{13}C-NMR (150 MHz) spectrum of compound **33** in DMSO-d_6.

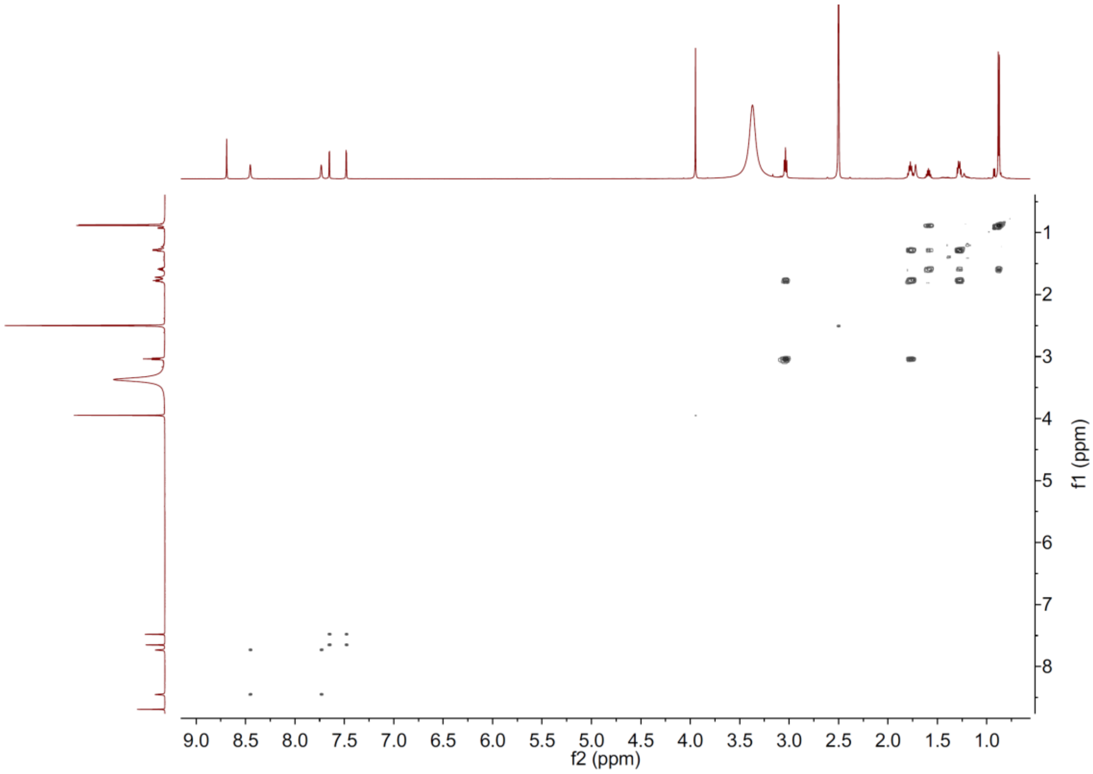

Supplementary Figure 90. ^{1}H-^{1}H COSY (600 MHz) spectrum of compound **33** in DMSO-d_6.

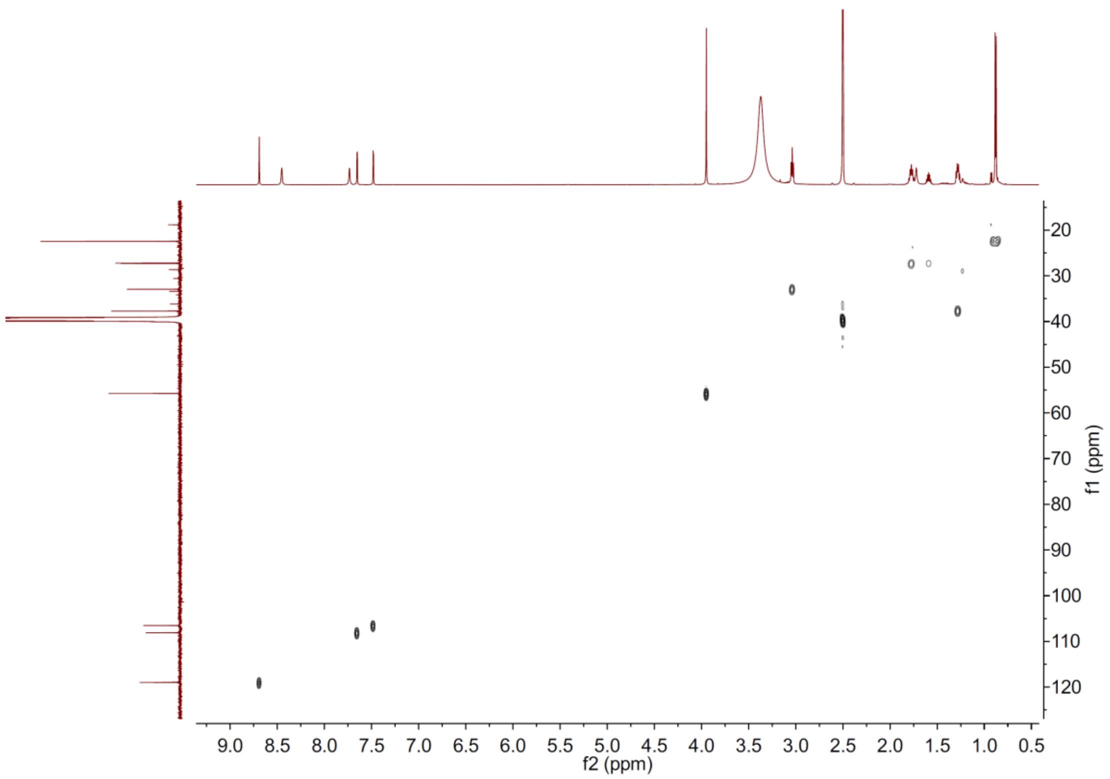

Supplementary Figure 91. HSQC (600 MHz) spectrum of compound **33** in DMSO-d_6.

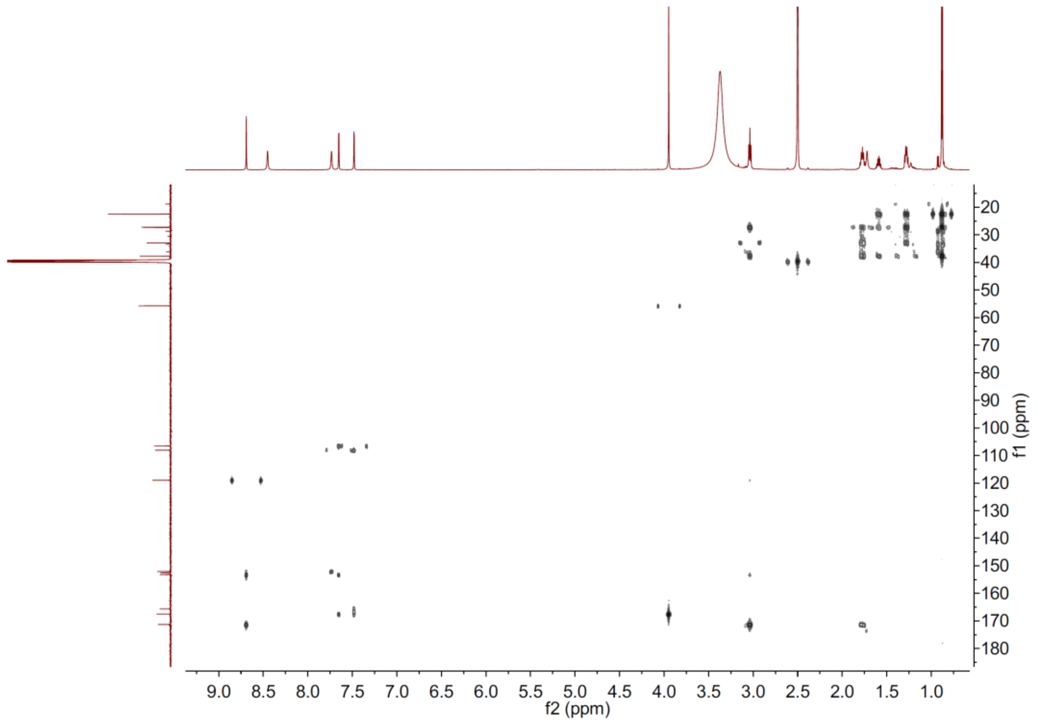

Supplementary Figure 92. HMBC (600 MHz) spectrum of compound **33** in DMSO-d_6.

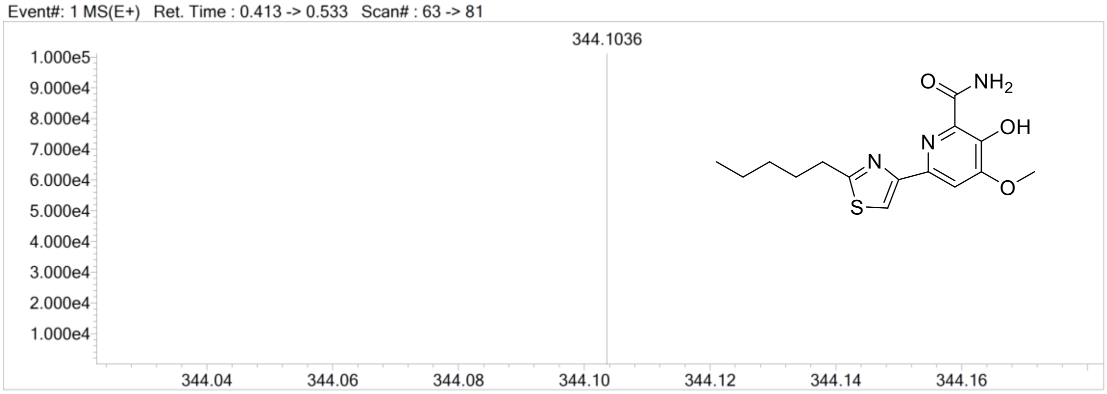

Formula (M)	Ion	Meas. m/z	Pred. m/z	Df. (mDa)	Df. (ppm)	DBE
C15 H19 N3 O3 S	[M+Na]+	344.1036	344.1039	-0.3	-0.87	8.0

Supplementary Figure 93. HRESIMS spectrum of compound **35**.

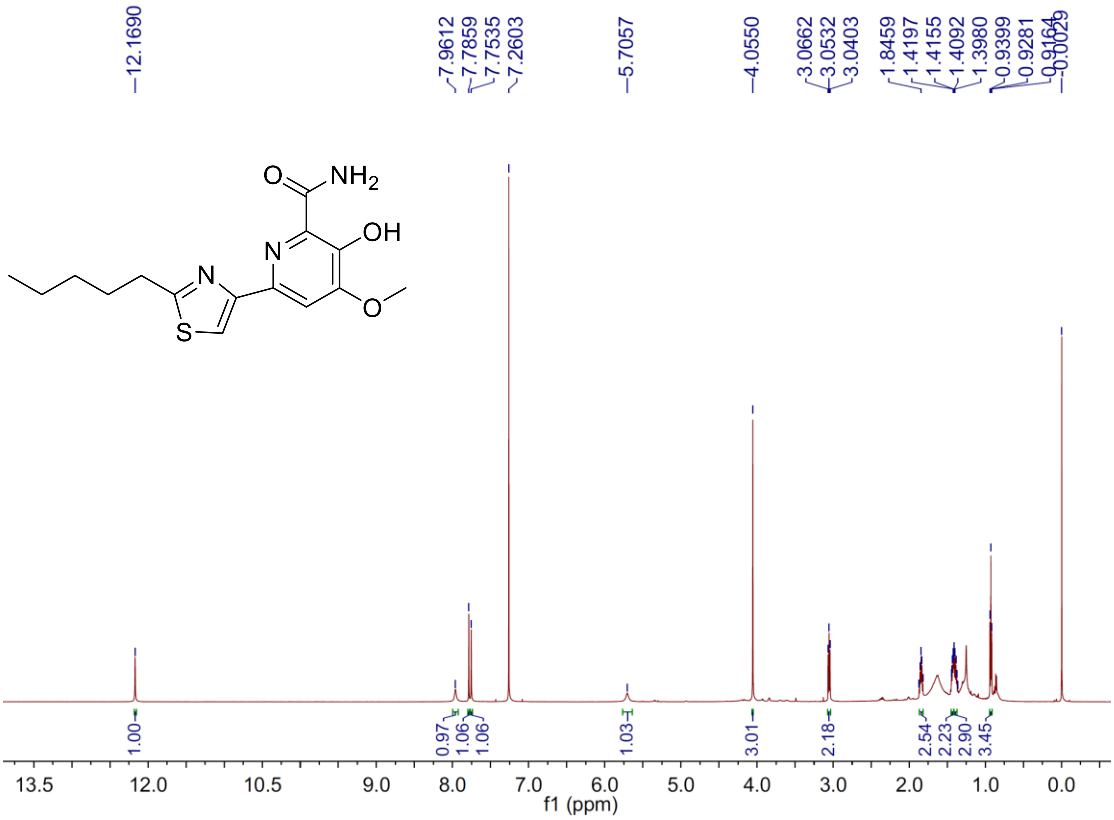

Supplementary Figure 94. ^{1}H-NMR (600 MHz) spectrum of compound **35** in $CDCl_3$.

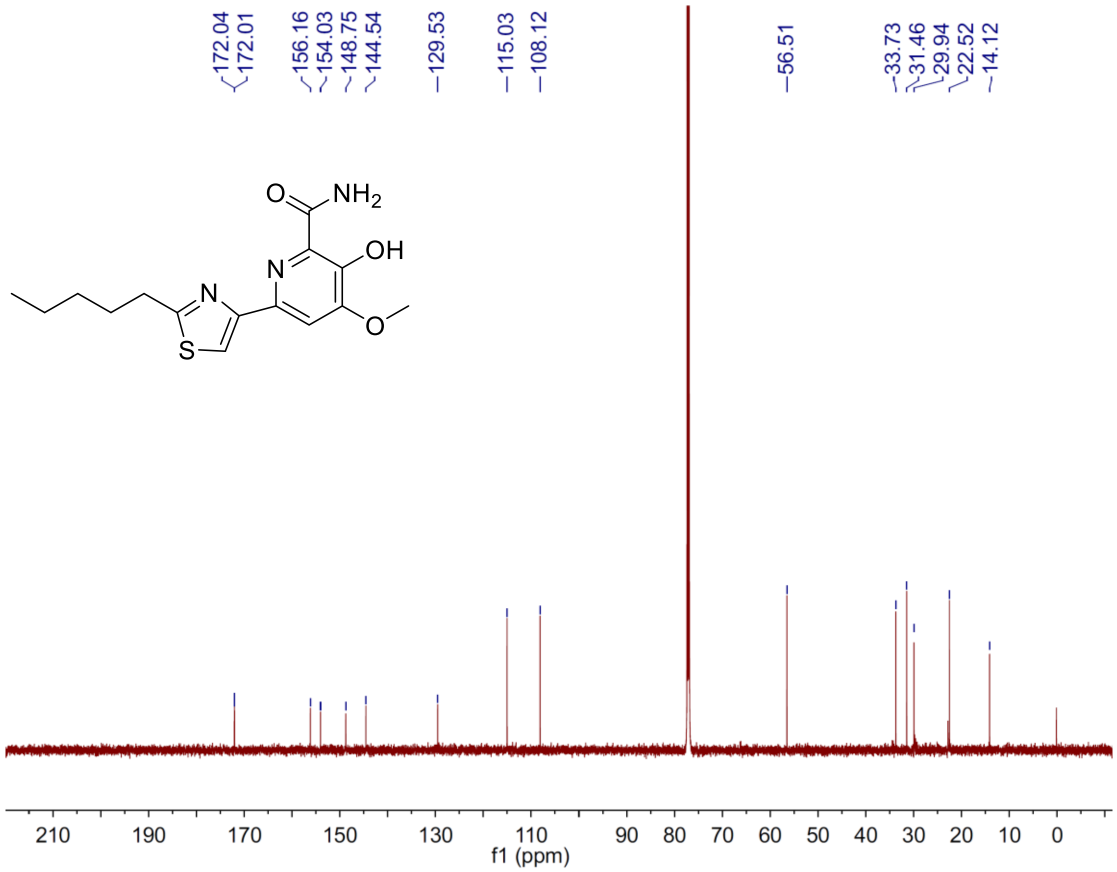

Supplementary Figure 95. ^{13}C-NMR (150 MHz) spectrum of compound **35** in $CDCl_3$.

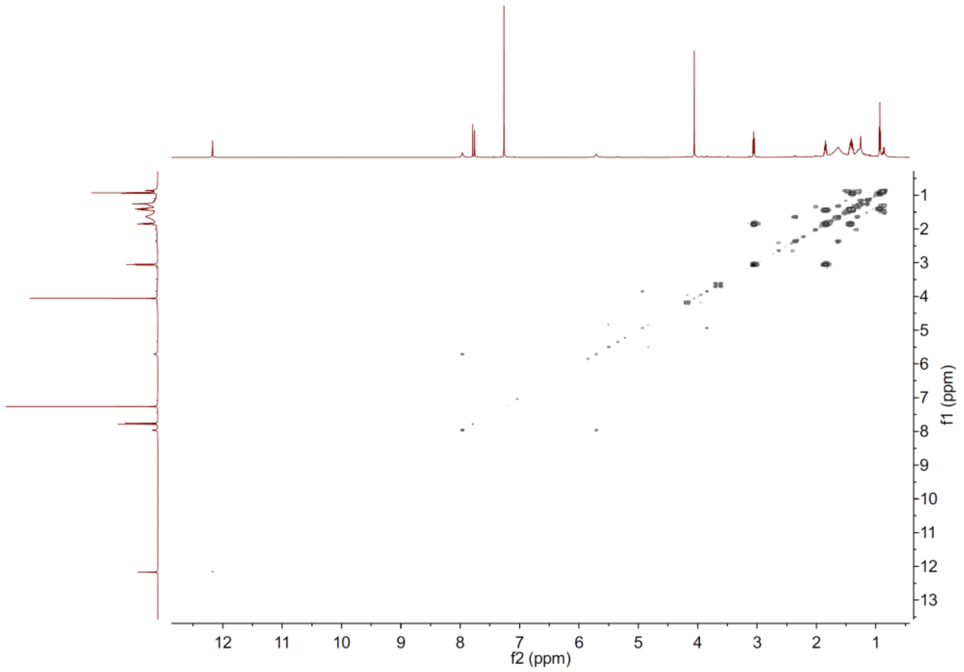

Supplementary Figure 96. ^{1}H-^{1}H COSY (600 MHz) spectrum of compound **35** in $CDCl_3$.

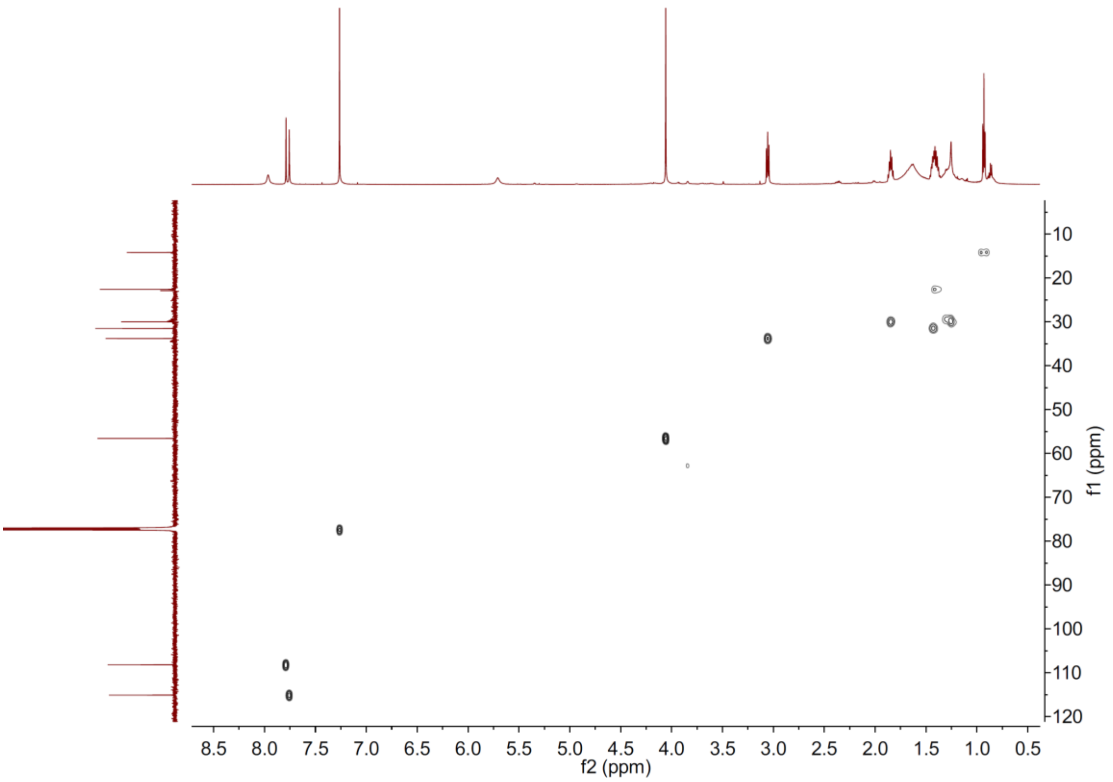

Supplementary Figure 97. HSQC (600 MHz) spectrum of compound **35** in $CDCl_3$.

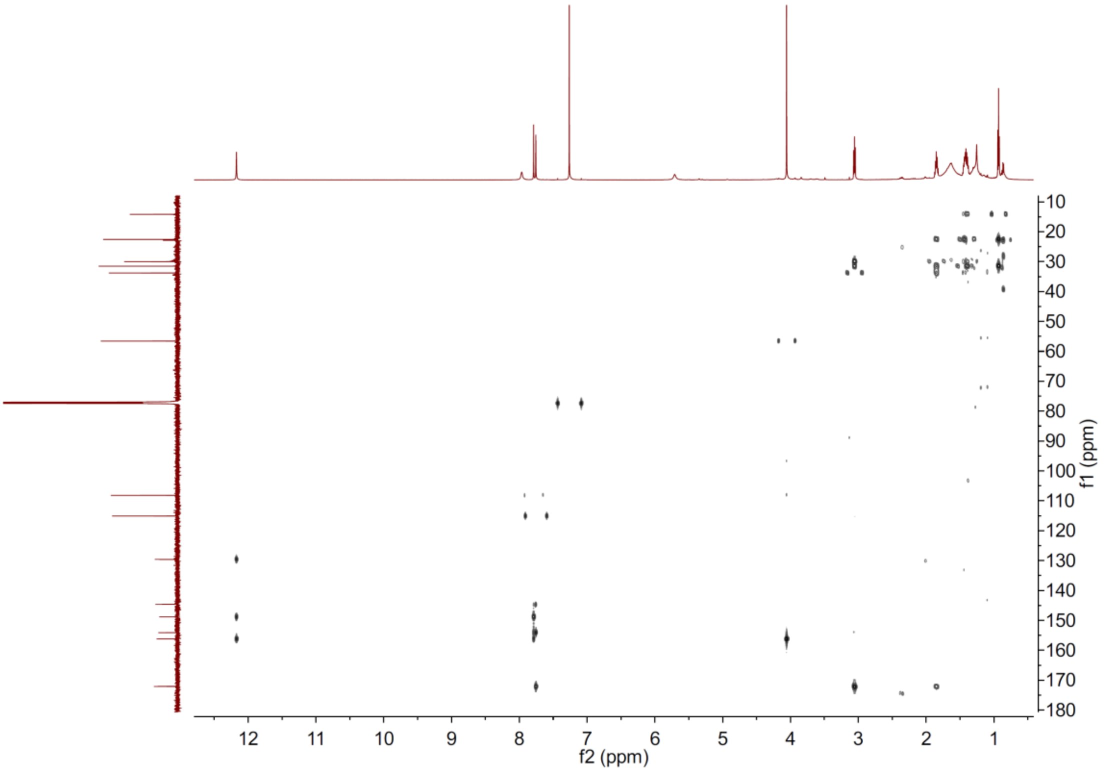

Supplementary Figure 98. HMBC (600 MHz) spectrum of compound **35** in $CDCl_3$.

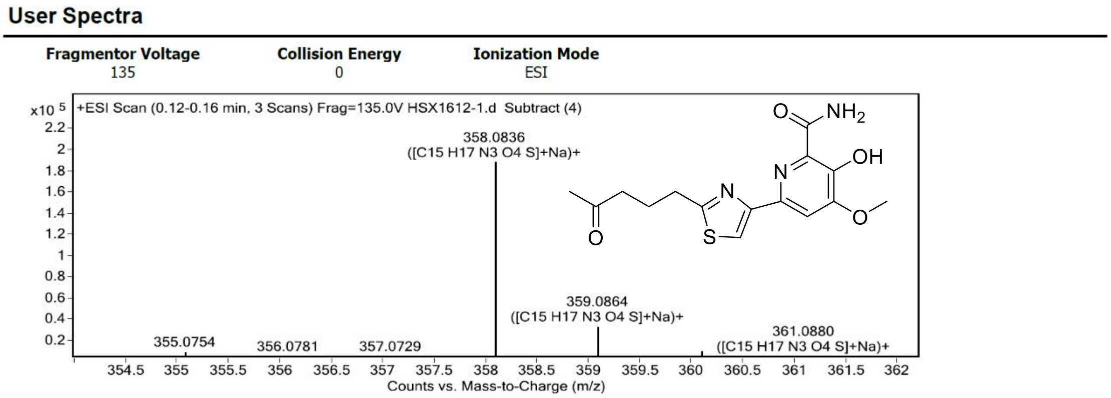

Supplementary Figure 99. HRESIMS spectrum of compound **36**.

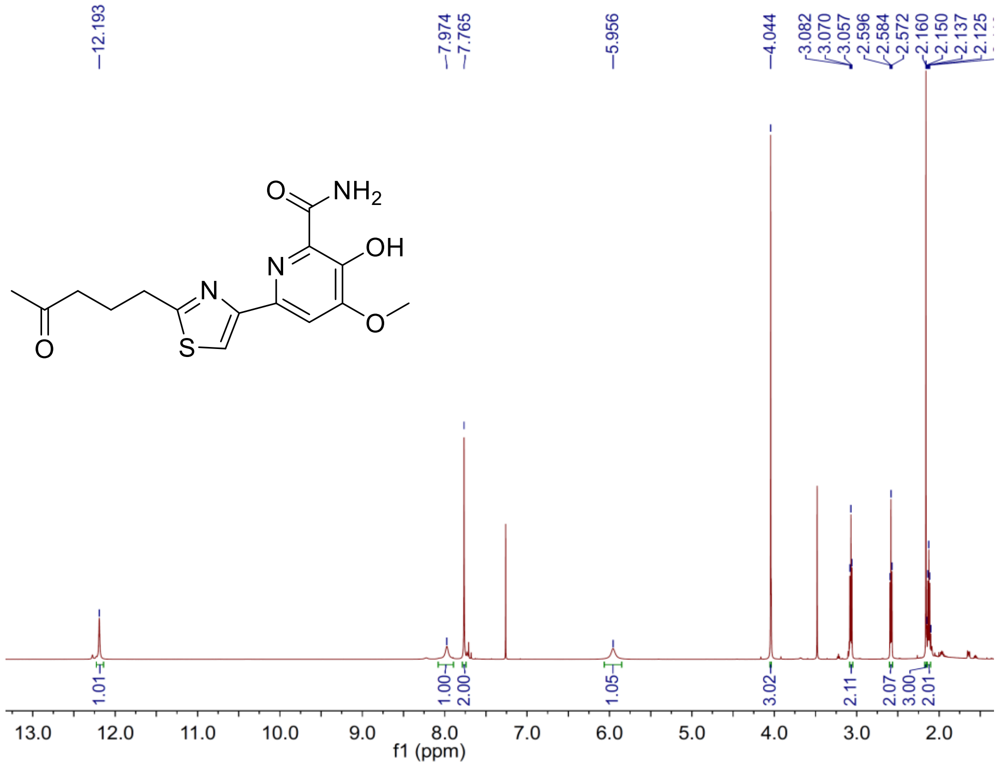

Supplementary Figure 100. ^{1}H-NMR (600 MHz) spectrum of compound **36** in $CDCl_3$.

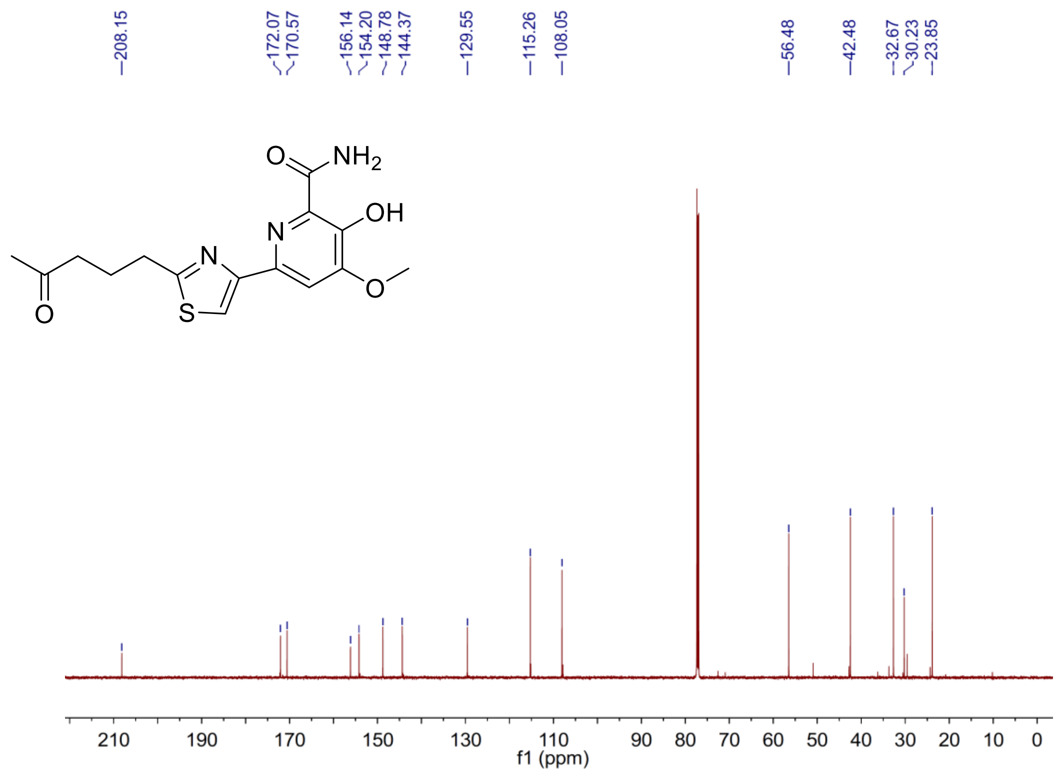

Supplementary Figure 101. ^{13}C-NMR (150 MHz) spectrum of compound **36** in $CDCl_3$.

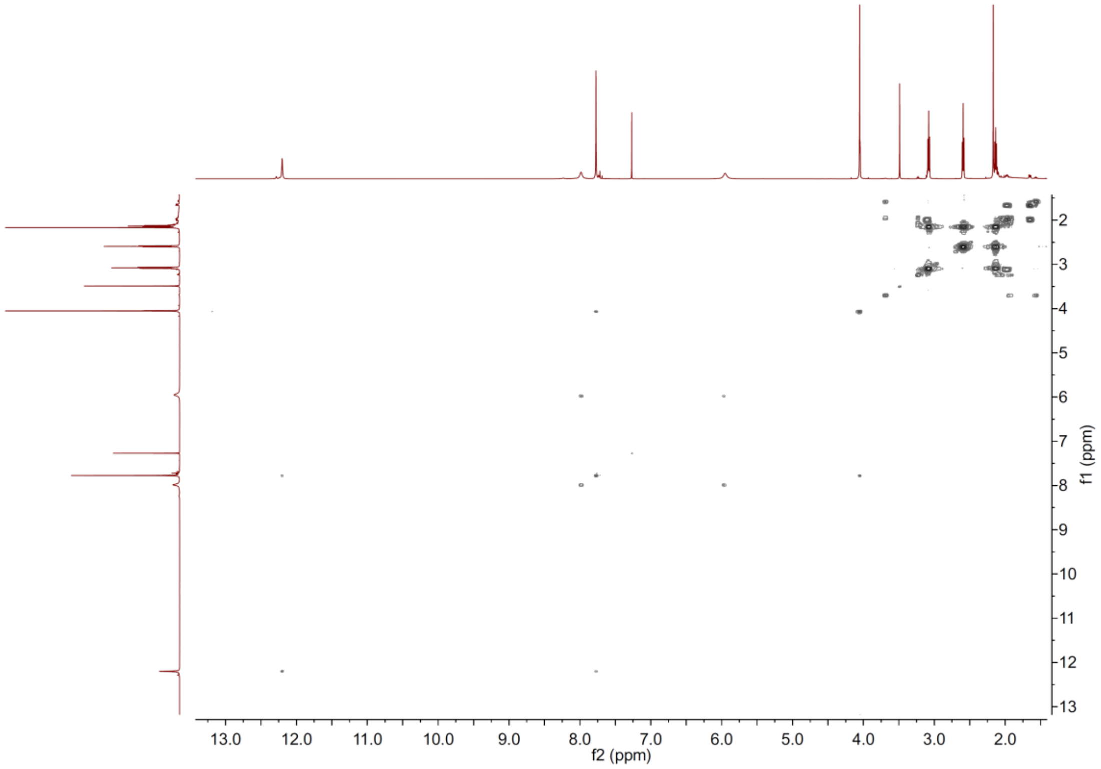

Supplementary Figure 102. ^{1}H-^{1}H COSY (600 MHz) spectrum of compound **36** in $CDCl_3$.

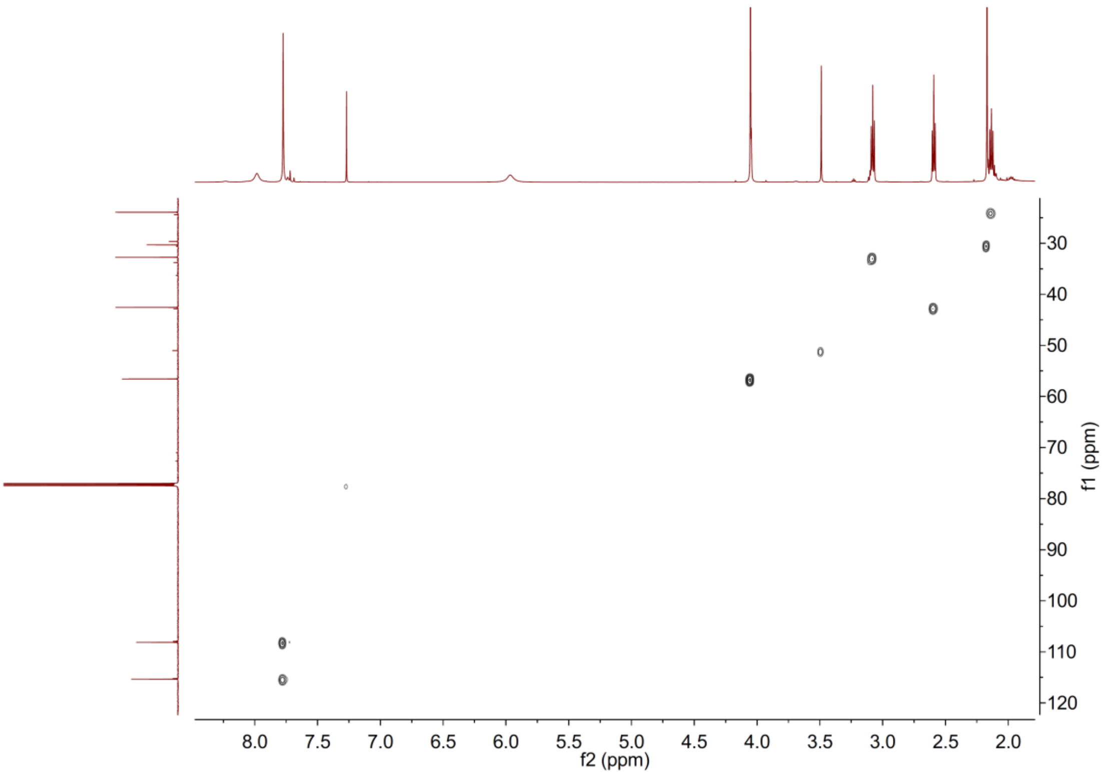

Supplementary Figure 103. HSQC (600 MHz) spectrum of compound **36** in $CDCl_3$.

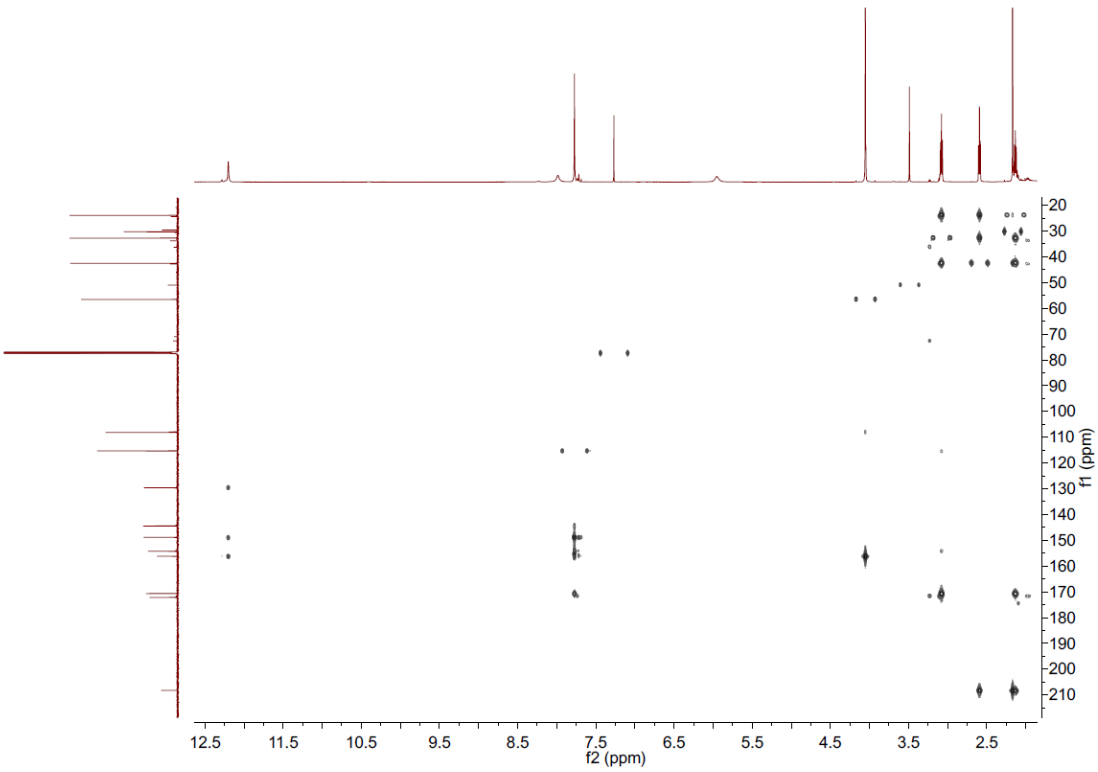

Supplementary Figure 104. HMBC (600 MHz) spectrum of compound **36** in $CDCl_3$.

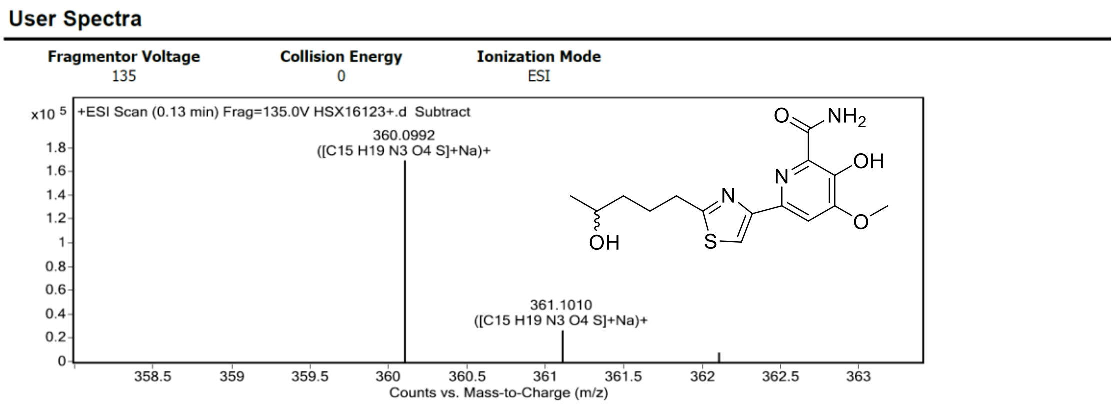

Supplementary Figure 105. HRESIMS spectrum of compound **37**.

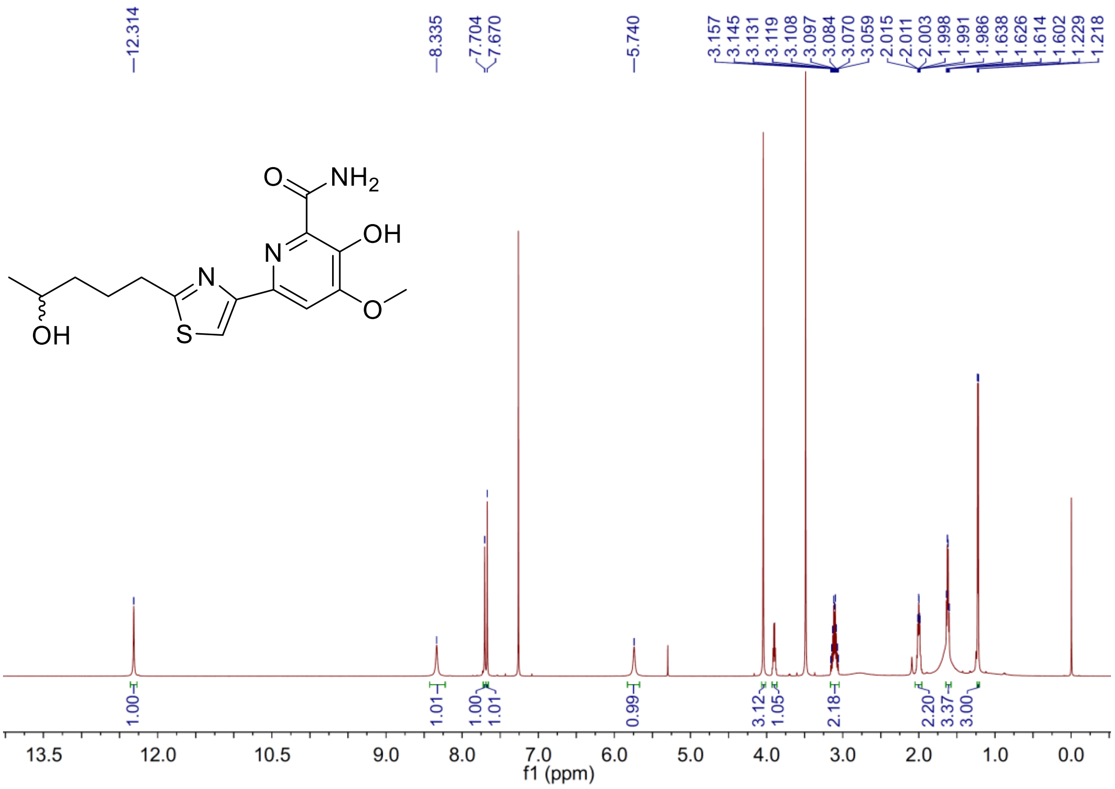

Supplementary Figure 106. ^{1}H-NMR (600 MHz) spectrum of compound **37** in $CDCl_3$.

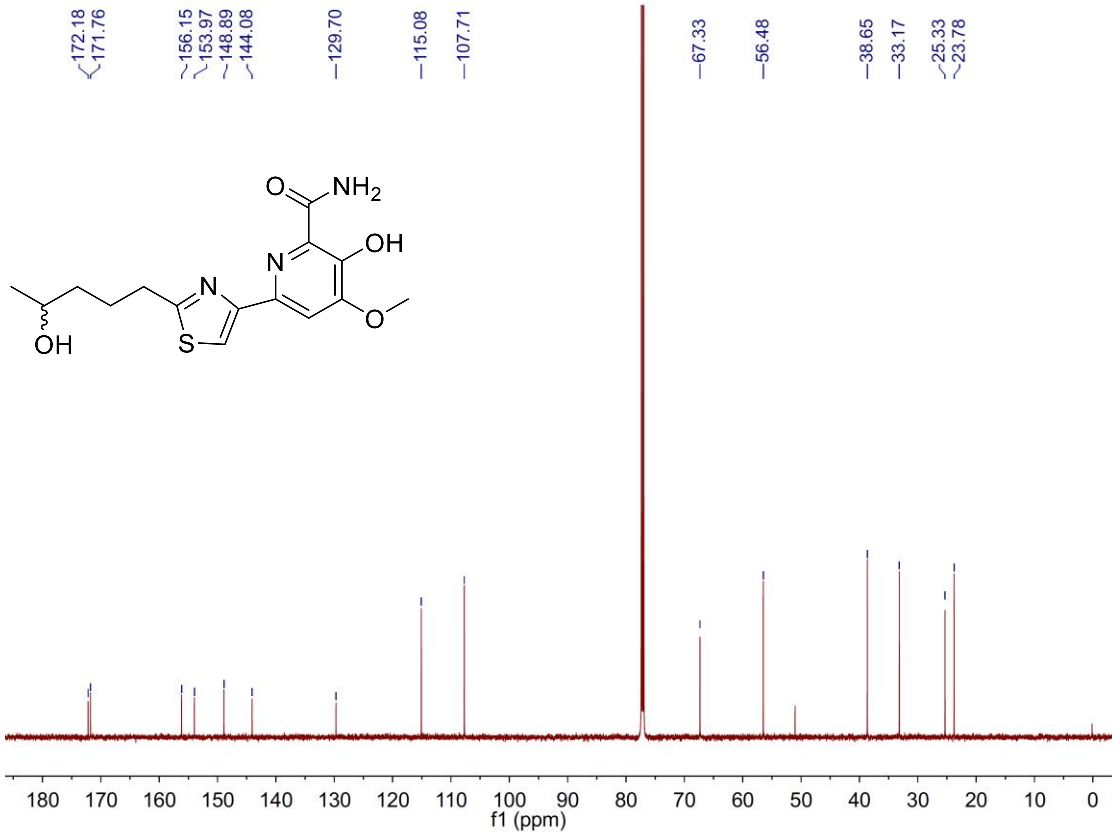

Supplementary Figure 107. ^{13}C-NMR (150 MHz) spectrum of compound **37** in $CDCl_3$.

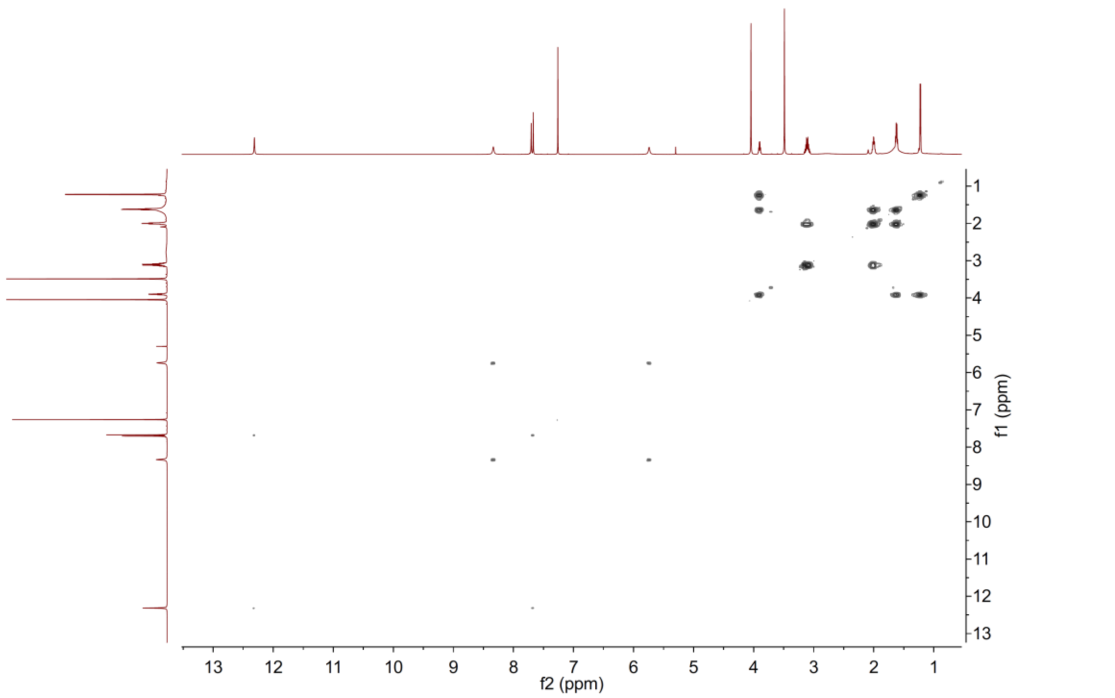

Supplementary Figure 108. ^{1}H-^{1}H COSY (600 MHz) spectrum of compound **37** in $CDCl_3$.

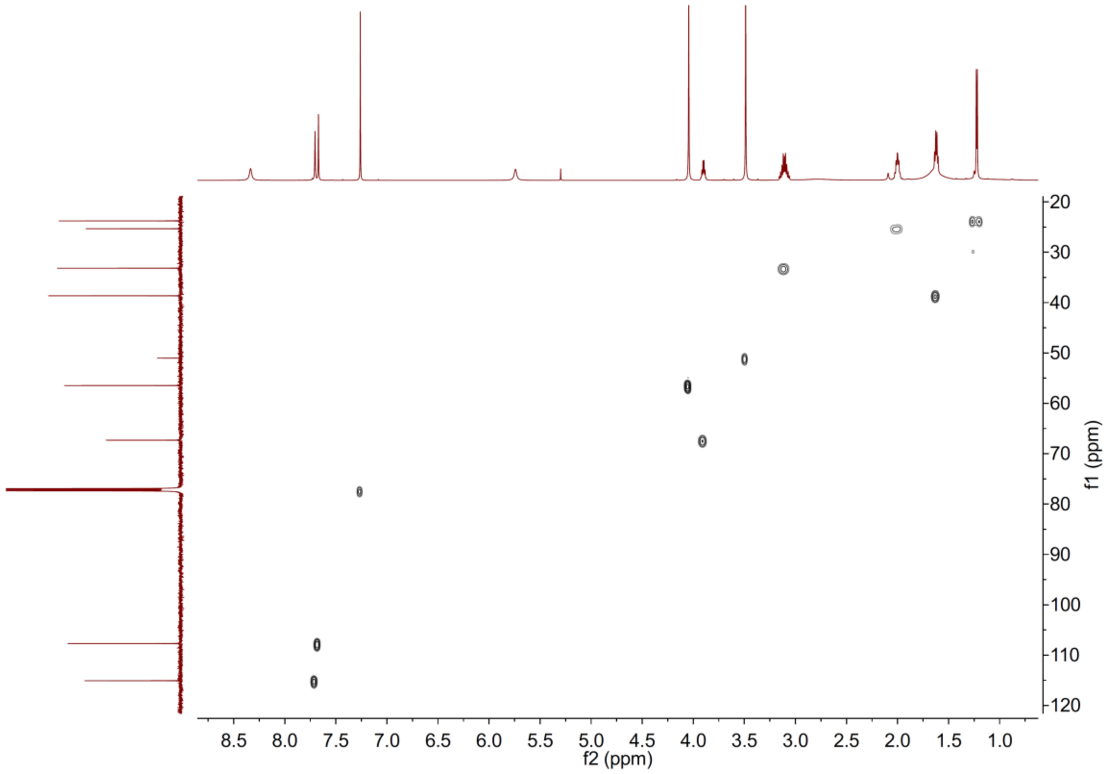

Supplementary Figure 109. HSQC (600 MHz) spectrum of compound **37** in $CDCl_3$.

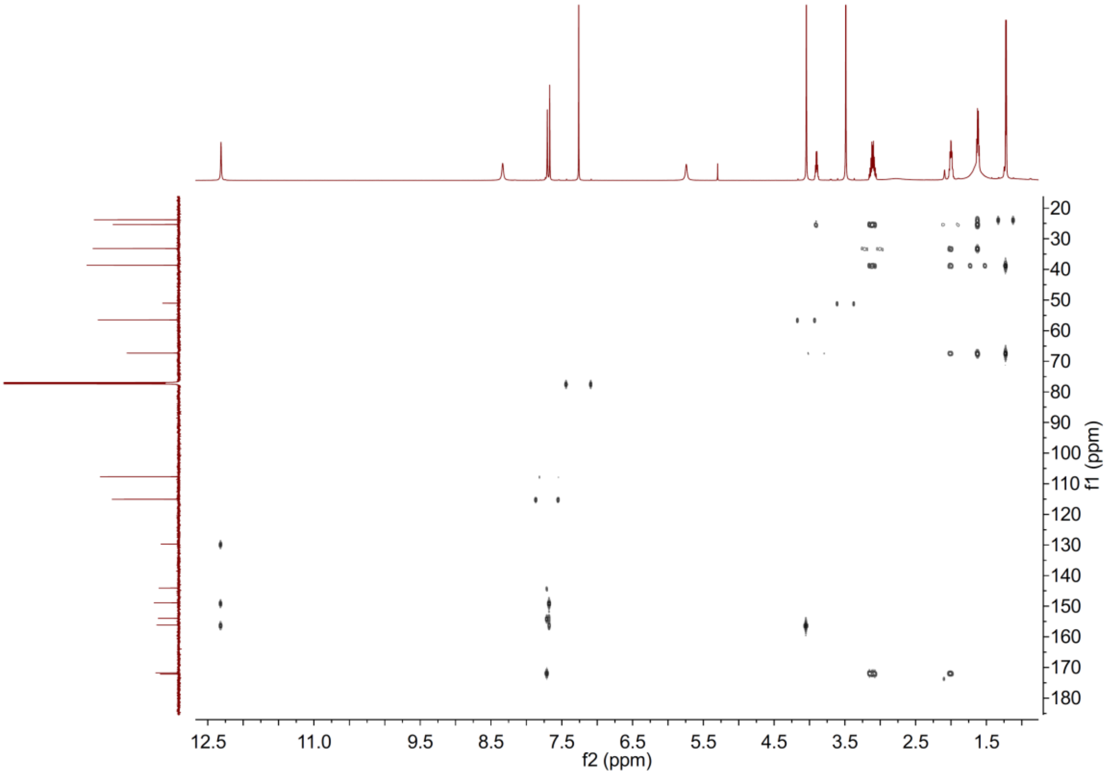

Supplementary Figure 110. HMBC (600 MHz) spectrum of compound **37** in $CDCl_3$.

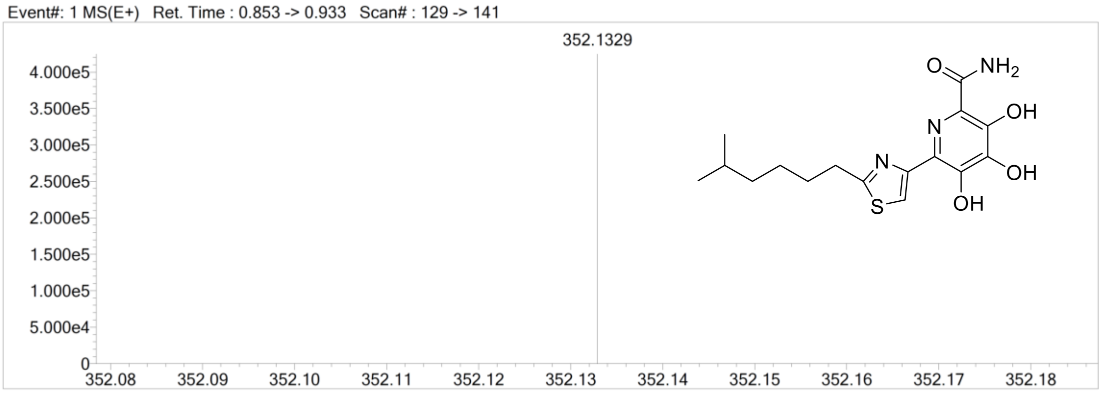

Formula (M)	Ion	Meas. m/z	Pred. m/z	Df. (mDa)	Df. (ppm)	DBE
C16 H21 N3 O4 S	[M+H]+	352.1329	352.1326	0.3	0.85	8.0

Supplementary Figure 111. HRESIMS spectrum of compound **28**.

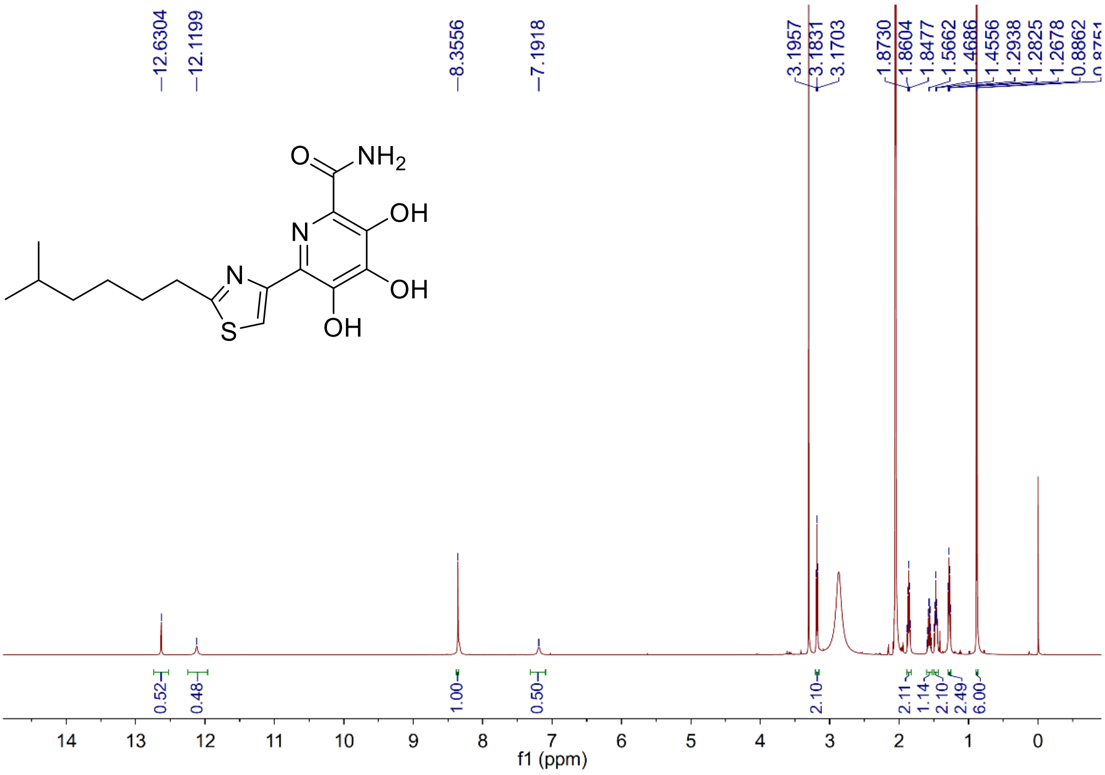

Supplementary Figure 112. ^{1}H-NMR (600 MHz) spectrum of compound **28** in Acetone-d_6.

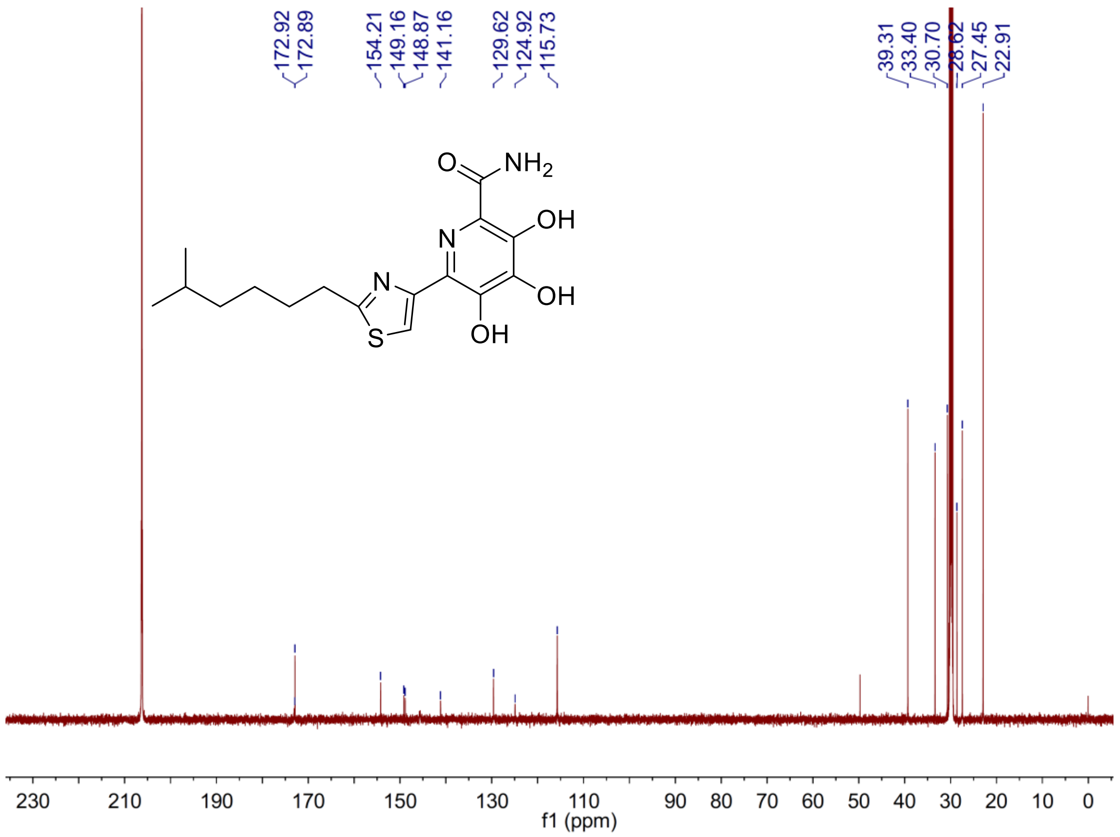

Supplementary Figure 113. ^{13}C-NMR (150 MHz) spectrum of compound **28** in Acetone-d_6.

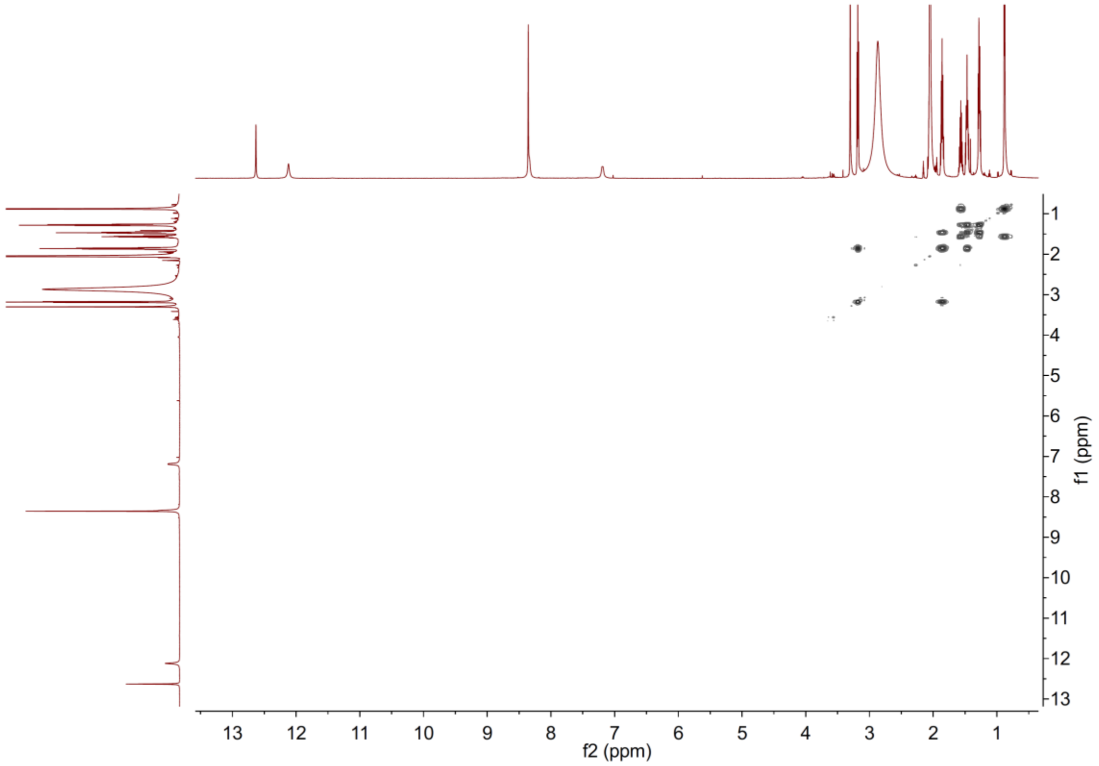

Supplementary Figure 114. ^{1}H-^{1}H COSY (600 MHz) spectrum of compound **28** in Acetone-d_6.

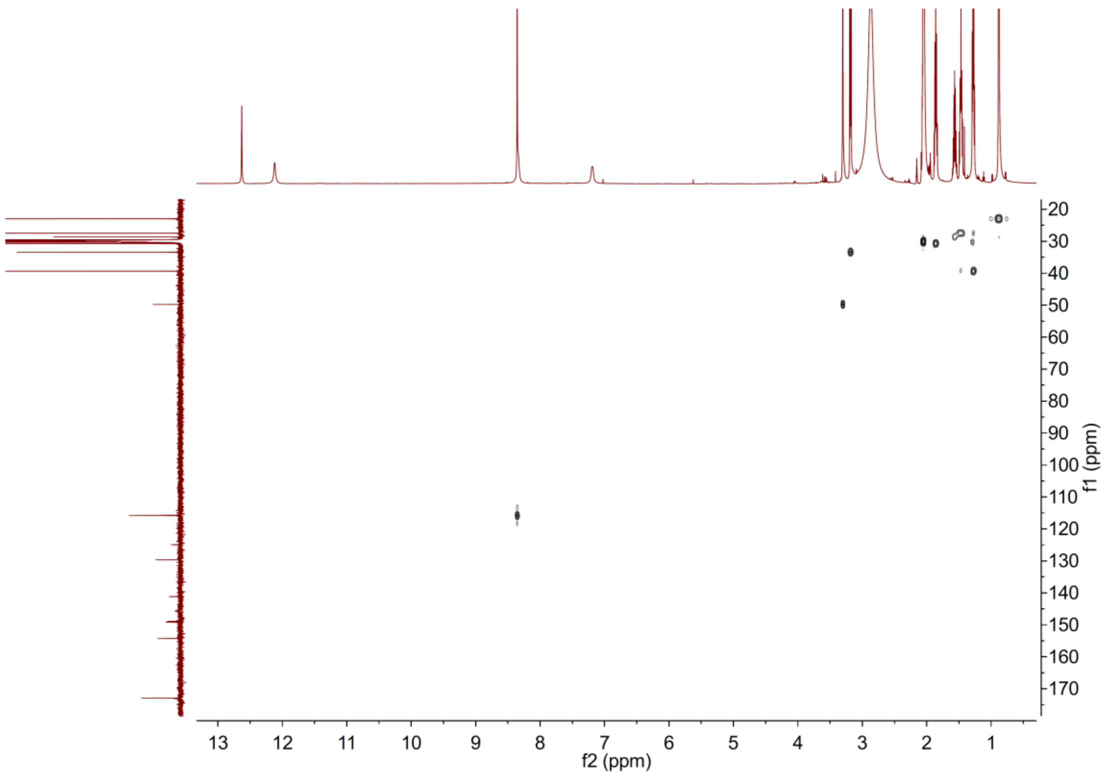

Supplementary Figure 115. HSQC (600 MHz) spectrum of compound **28** in Acetone-*d*6.

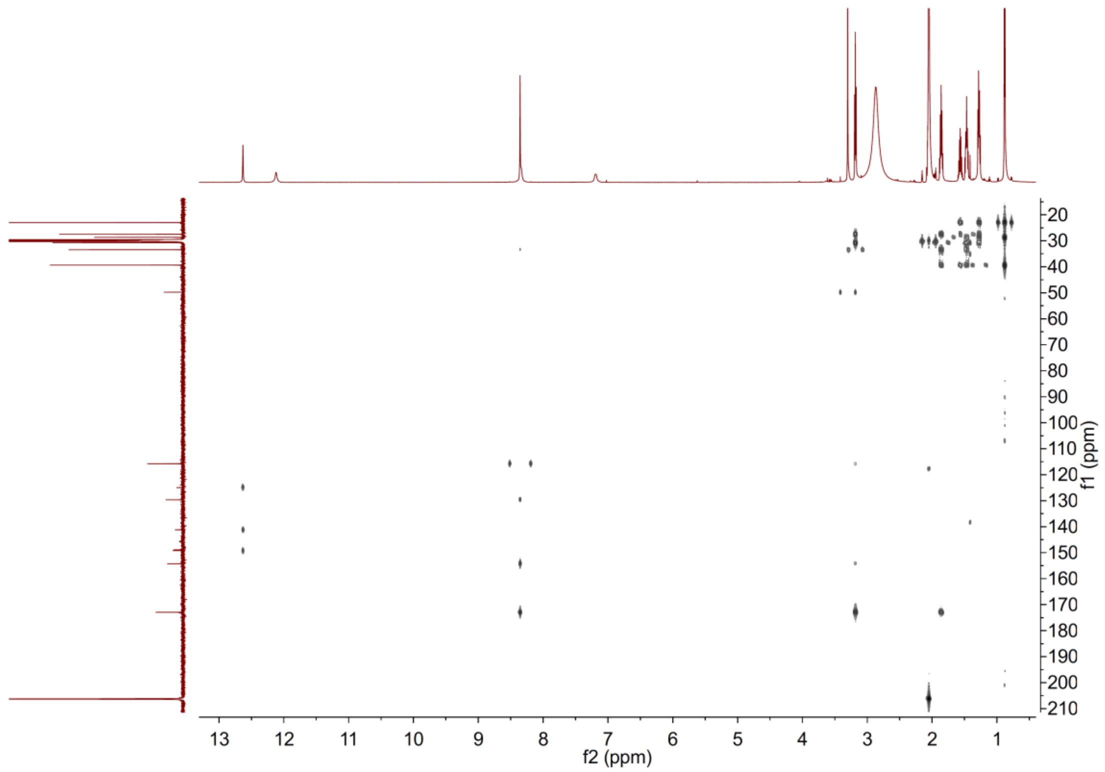

Supplementary Figure 116. HMBC (600 MHz) spectrum of compound **28** in Acetone-*d*6.

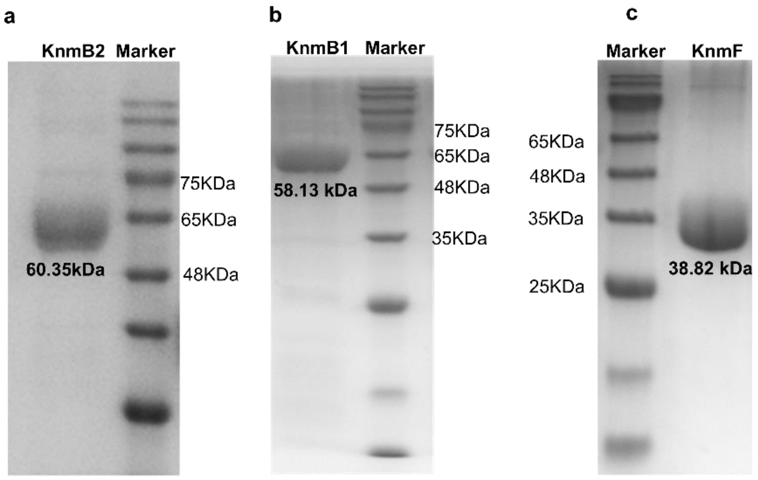

Supplementary Figure 117. SDS-PAGE analysis of proteins. (**a**) KnmB2 (recombinant KnmB2 fused with thioredoxin, His-tag, a thrombin site, a S-Tag and an enterokinase site, calculated molecular mass: 60.35 kDa). (**b**) KnmB1 (recombinant KnmB1 fused with thioredoxin, His-tag, a thrombin site, a S-Tag and an enterokinase site, calculated molecular mass: 58.13 kDa). (**c**) KnmF (recombinant KnmF fused with His-tag, calculated molecular mass: 38.82 kDa). A representative result of *n* = 3 independent experiments is shown.

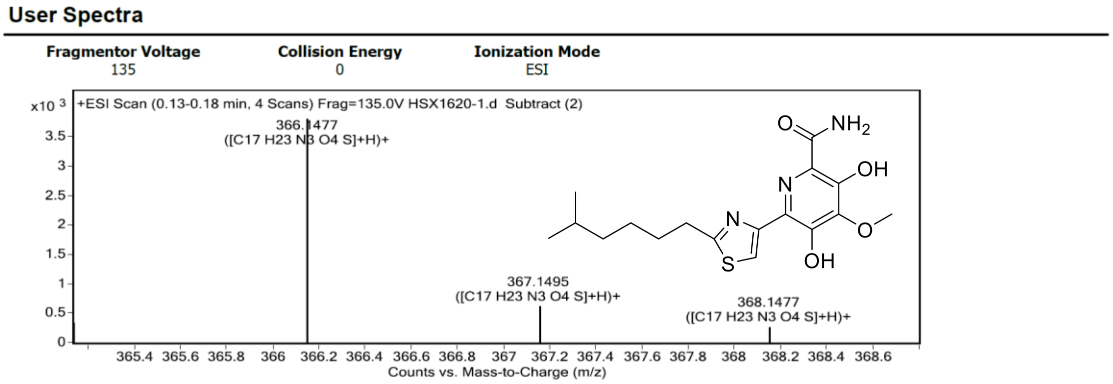

Supplementary Figure 118. HRESIMS spectrum of compound **30**.

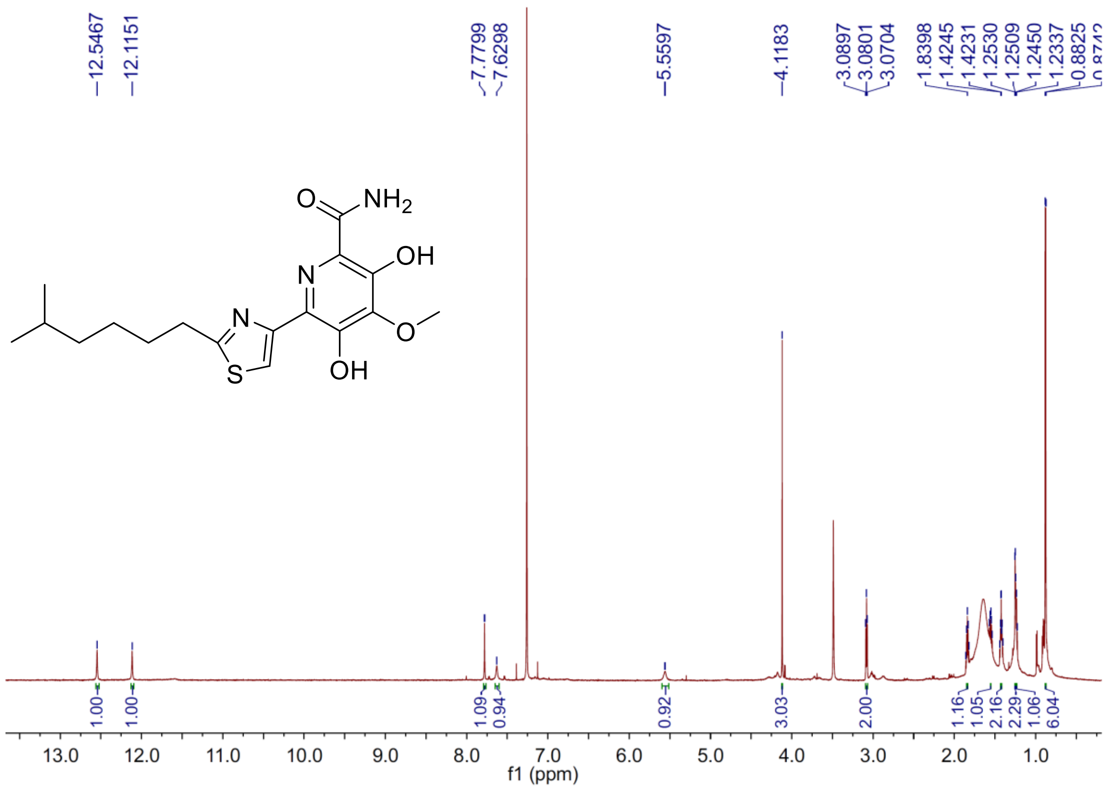

Supplementary Figure 119. ^{1}H-NMR (800 MHz) spectrum of compound **30** in $CDCl_3$.

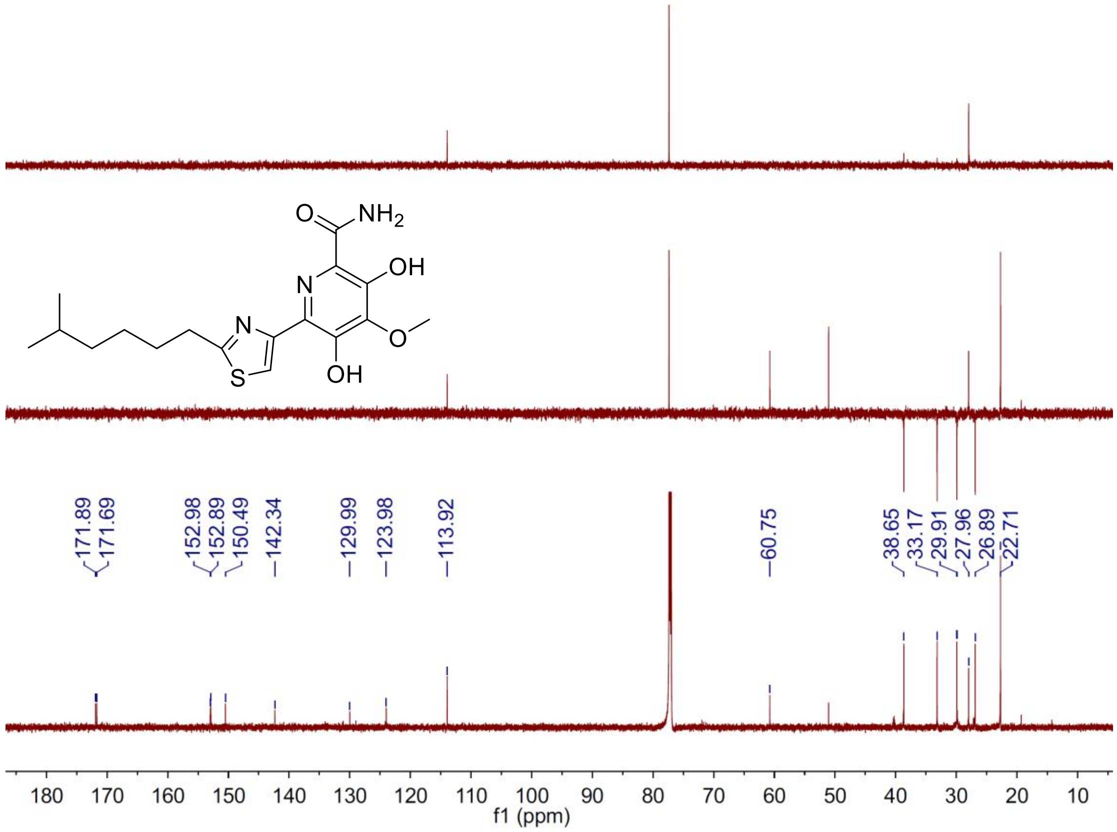

Supplementary Figure 120. ^{13}C-NMR (200 MHz) spectrum of compound **30** in $CDCl_3$.

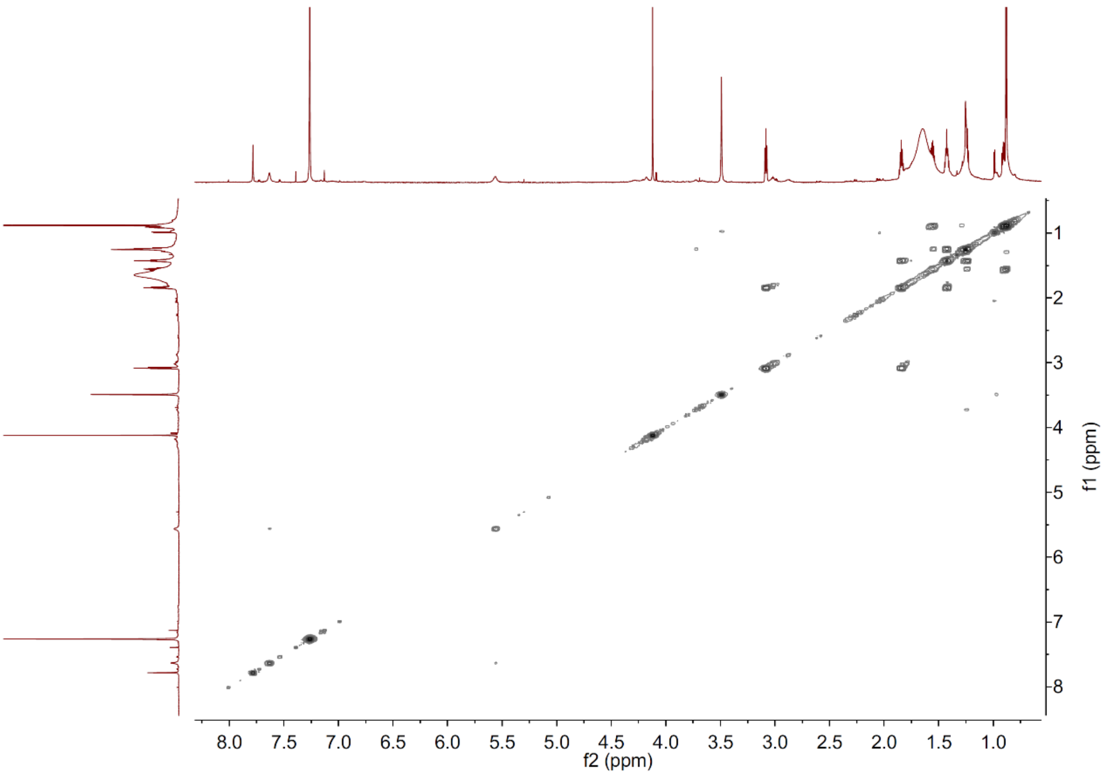

Supplementary Figure 121. ^1H-^1H COSY (800 MHz) spectrum of compound **30** in $CDCl_3$.

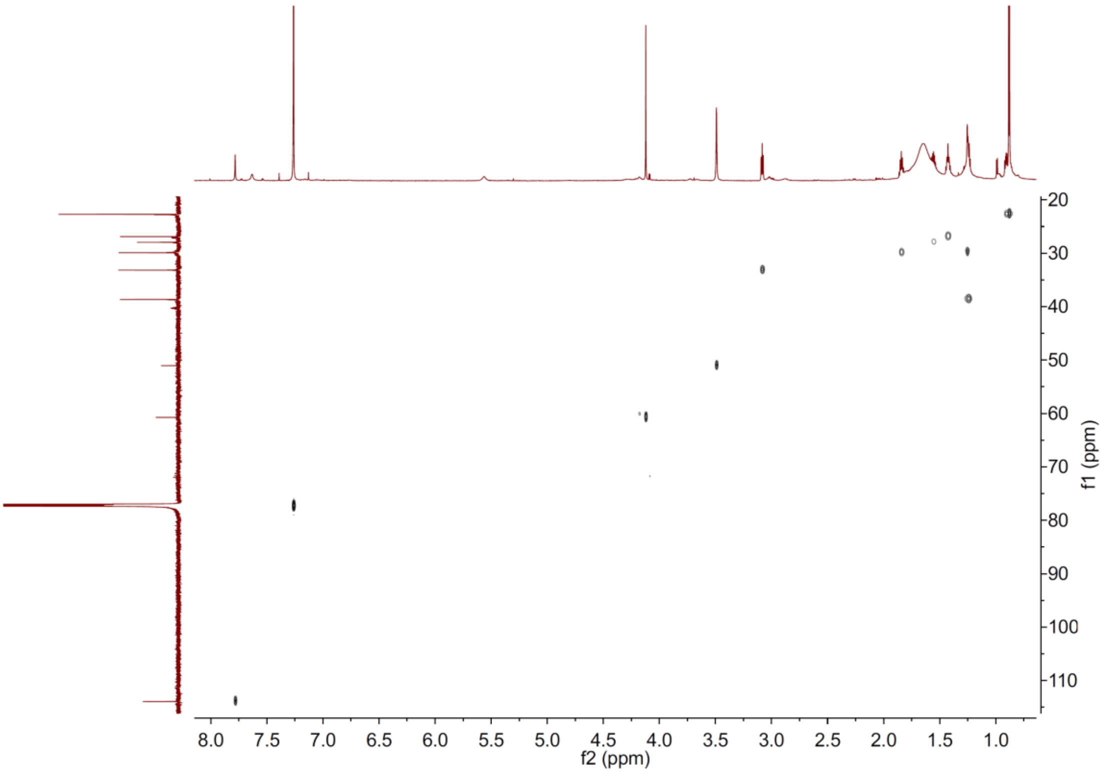

Supplementary Figure 122. HSQC (800 MHz) spectrum of compound **30** in $CDCl_3$.

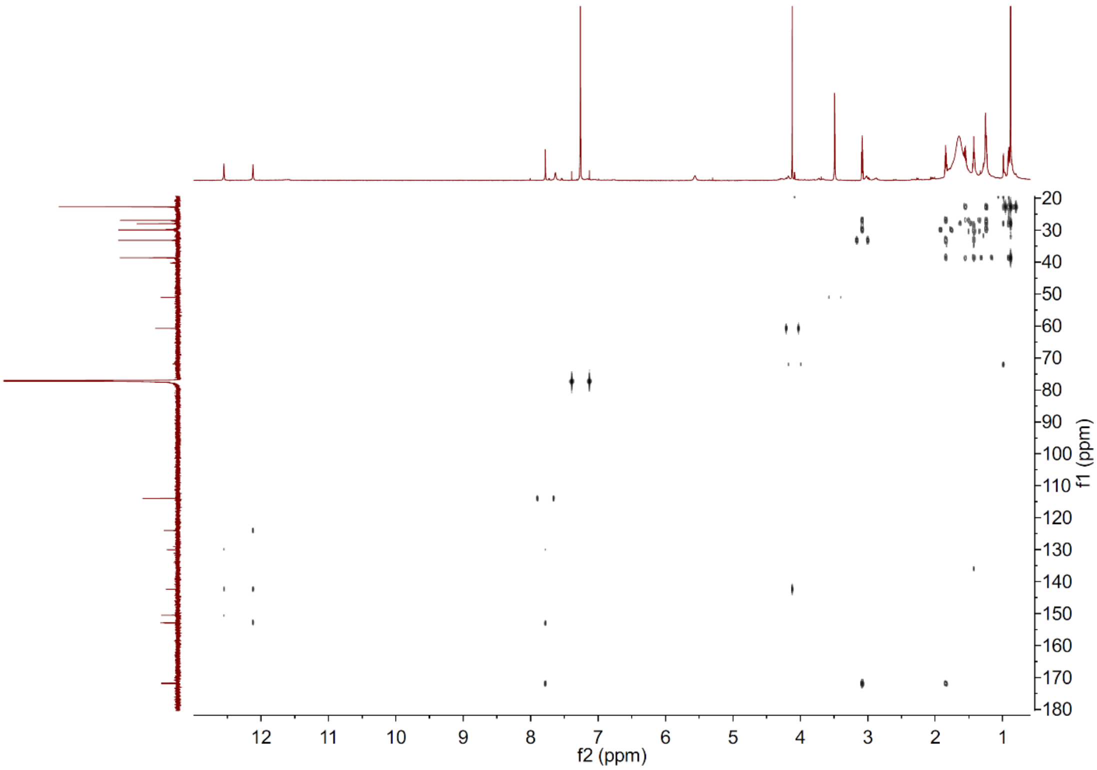

Supplementary Figure 123. HMBC (800 MHz) spectrum of compound **30** in $CDCl_3$.

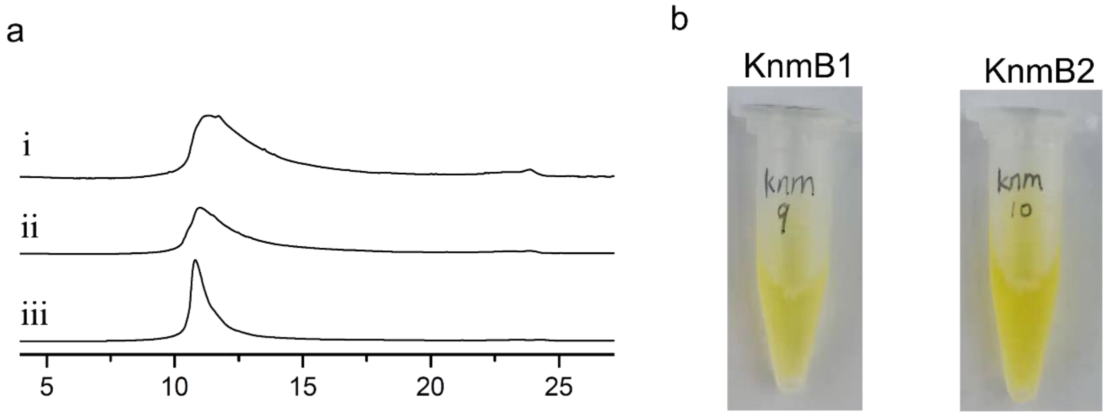

Supplementary Figure 124. Examination of the protein nature of KnmB1 and KnmB2. (**a**) HPLC analysis of (i) standard FAD; (ii) the supernatant of KnmB2; (iii) the supernatant of KnmB1. The UV absorbance was monitored at 450 nm. Source data are provided as a Source Data file. (**b**) Purified proteins KnmB1 and KnmB2. A representative result of *n* = 3 independent experiments is shown.

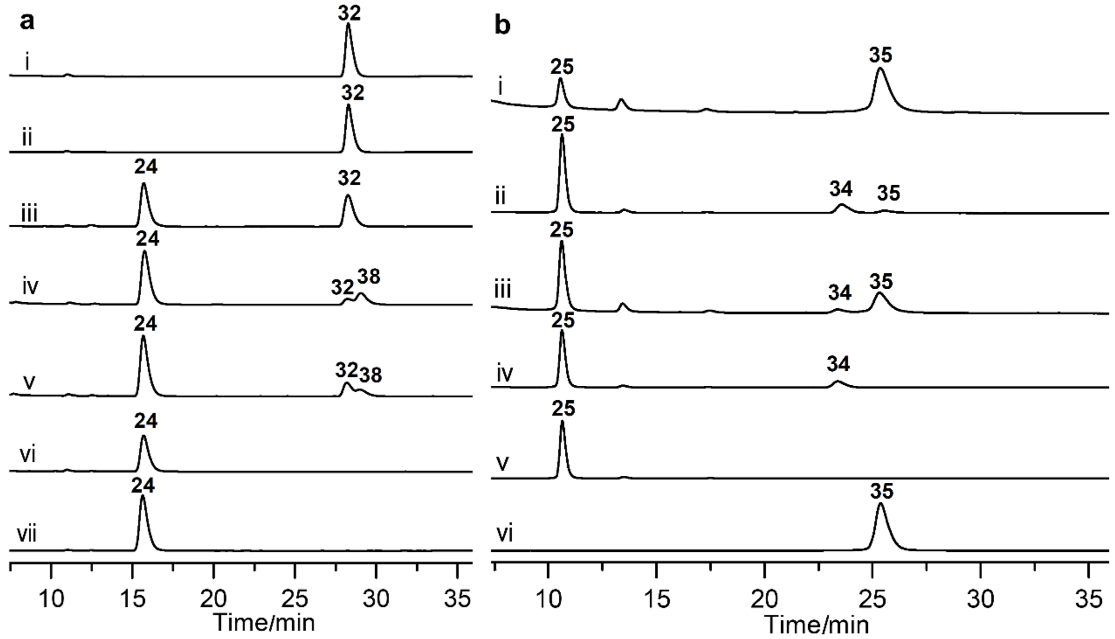

Supplementary Figure 125. In vitro characterization of KnmB2-catalyzed reactions. (**a**) (i) the control reaction in the presence of **32** and NADPH; (ii) the reaction in the presence of **32**, NADPH and KnmB2; (iii) the reaction in the presence of **24**, SAM and KnmF; (iv) the reaction in the presence of **24**, SAM, NADPH, KnmB2 and KnmF; (v) the reaction in the presence of **24**, SAM, NADH, KnmB2 and KnmF; (vi) the reaction in the presence of **24**, NADPH and KnmB2; (vii) the control reaction in the presence of **24**, SAM and NADPH (NADH). HPLC analysis was performed with analytical method C. (**b**) (i) the reaction was conducted in the presence of **25**, SAM, NADPH and KnmB2, and KnmF was added after 30 min; (ii) the reaction in the presence of **25**, NADH, KnmB2 and KnmF; (iii) the reaction in the presence of **25**, SAM, NADPH, KnmB2 and KnmF; (iv) the reaction in the presence of **25**, SAM and KnmF; (v) the control reaction in the presence of **25**, SAM and NADPH; (vi) standard **35**. HPLC analysis was performed with a 45 min elution system as follows: T = 0 min, 50% B; T = 34 min, 50% B; T = 34.1min, 100% B; T = 39 min,100% B; T = 39.1.1 min, 50% B; T = 45 min, 50% B (A, H_2O + 0.1% CH_3COOH; B, MeCN). The UV absorbance was monitored at 260 nm. A representative result of n = 3 independent experiments is shown. Source data are provided as a Source Data file.

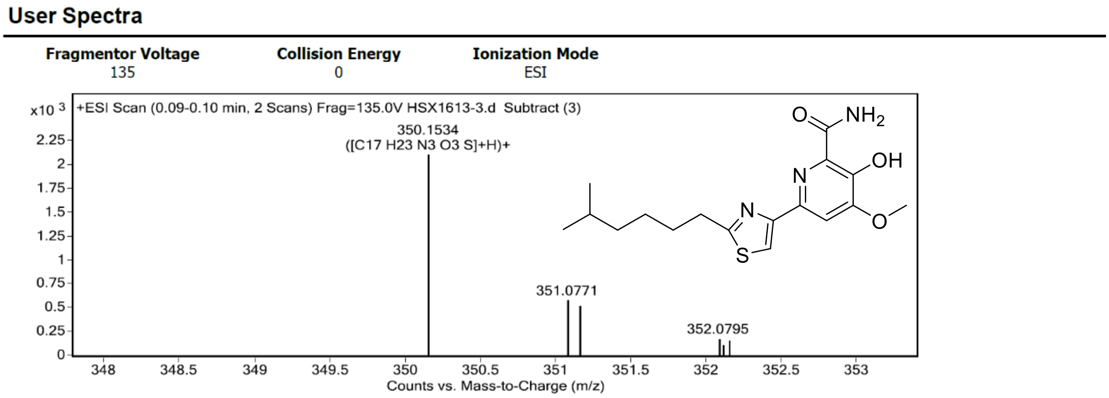

Supplementary Figure 126. HRESIMS spectrum of compound **38**.

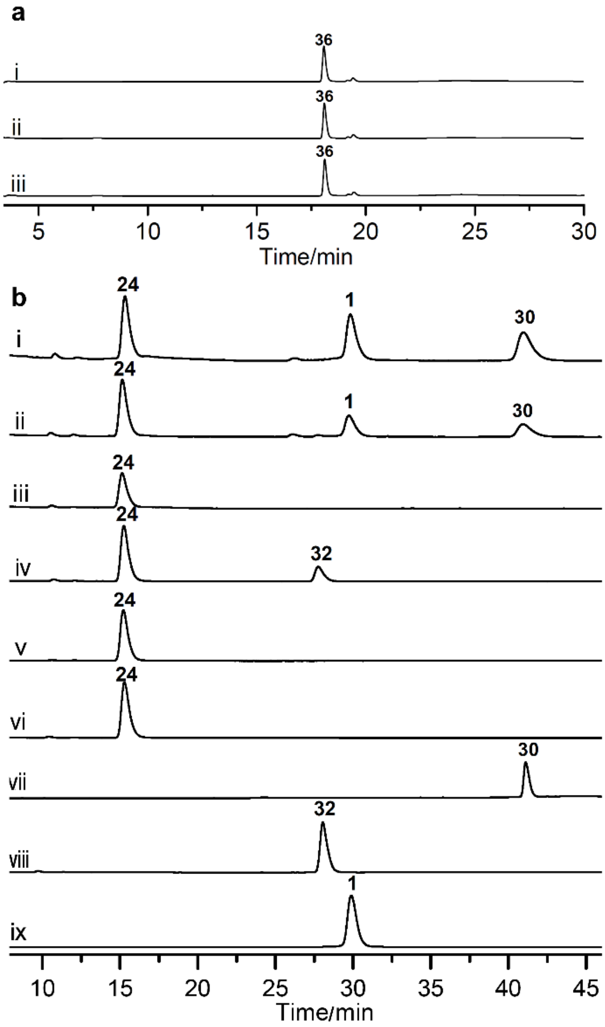

Supplementary Figure 127. In vitro characterization of KnmB1-catalyzed reactions. (**a**) (i) the reaction in the presence of **36**, NADPH and KnmB1; (ii) the reaction in the presence of **36**, NADPH, SAM, KnmB1 and KnmF; (iii) the control reaction in the presence of **36**, SAM and NADPH. HPLC analytical method: T = 0 min, 10% B; T = 20 min, 100% B; T = 25 min, 100% B; T = 25.1 min, 10% B; T = 30 min, 10% B (A, H_2O; B, CH_3OH). (**b**) (i) the reaction in the presence of **24**, SAM, NADPH, KnmB1, KnmB2 and KnmF; (ii) the reaction in the presence of **24**, SAM, NADH, KnmB1, KnmB2 and

KnmF; (iii) the reaction in the presence of **24**, NADPH, KnmB1 and KnmB2; (iv) the reaction in the presence of **24**, SAM, NADPH, KnmB1 and KnmF; (v) the reaction in the presence of **24**, NADPH and KnmB1; (vi) the control reaction in the presence of **24**, SAM and NADPH; (vii) standard **30**; (viii) standard **32**; (ix) standard **1**. HPLC analysis was performed with analytical method C. The UV absorbance was monitored at 260 nm. A representative result of n = 3 independent experiments is shown. Source data are provided as a Source Data file.

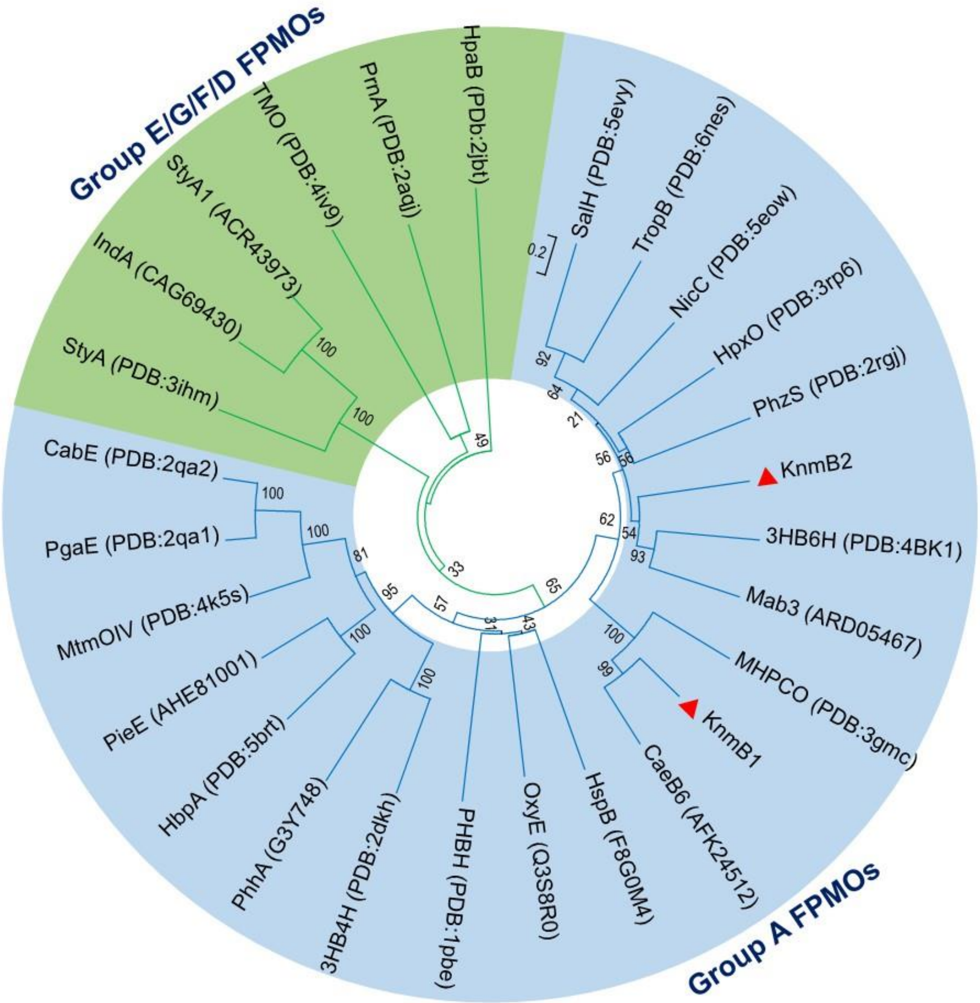

Supplementary Figure 128. Phylogenetic analysis of KnmB1, KnmB2 and the other group A FPMOs. KnmB1 and KnmB2 belong to group A FPMOs. The tree was calculated on the basis of an amino acid sequence alignment of 27 protein sequences of biochemically characterized enzymes by employing the ClustalW method in MEGA7[13]. Subsequently, the Neighbor-Joining method was used to generate the tree while the evolutionary distances were computed using the JTT matrix-based method[14]. The percentage of replicate trees in which the associated taxa clustered together in the bootstrap test (1000 replicates) are shown next to the branches[15]. To provide a comprehensive view, we used group D, E, F and G FPMOs representatives.

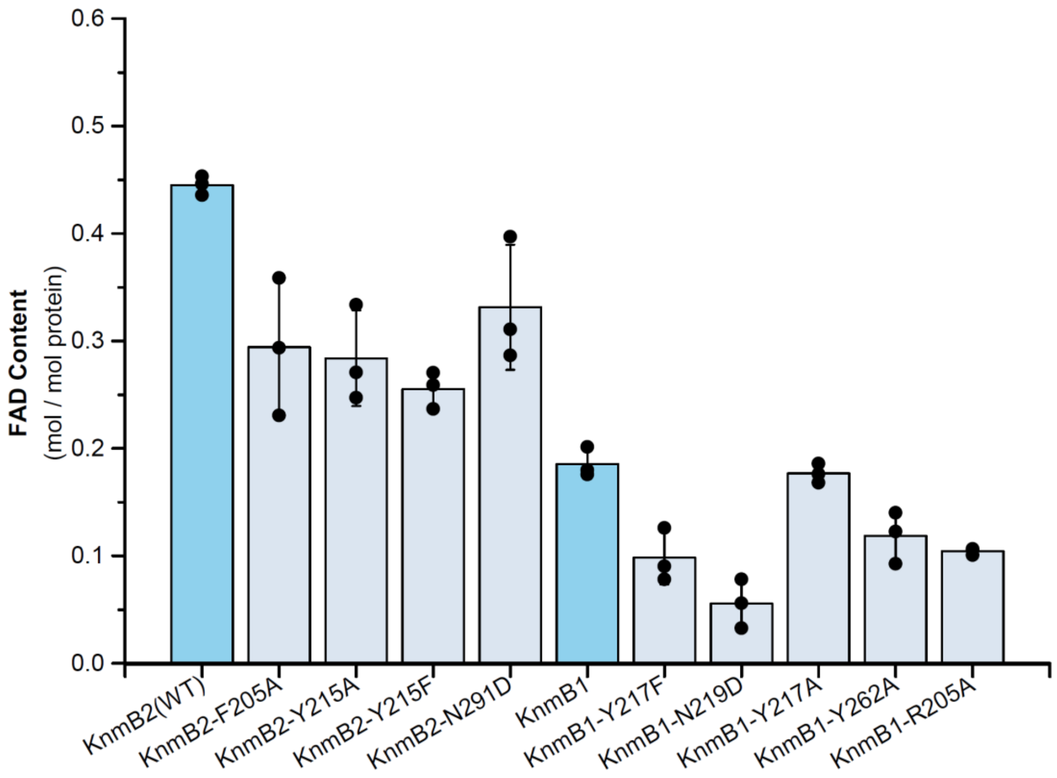

Supplementary Figure 129. FAD content of KnmB1, KnmB2 and variants. Data represent the mean of *n* = 3 independent replicates, and error bars denote S.D. Source data are provided as a Source Data file.

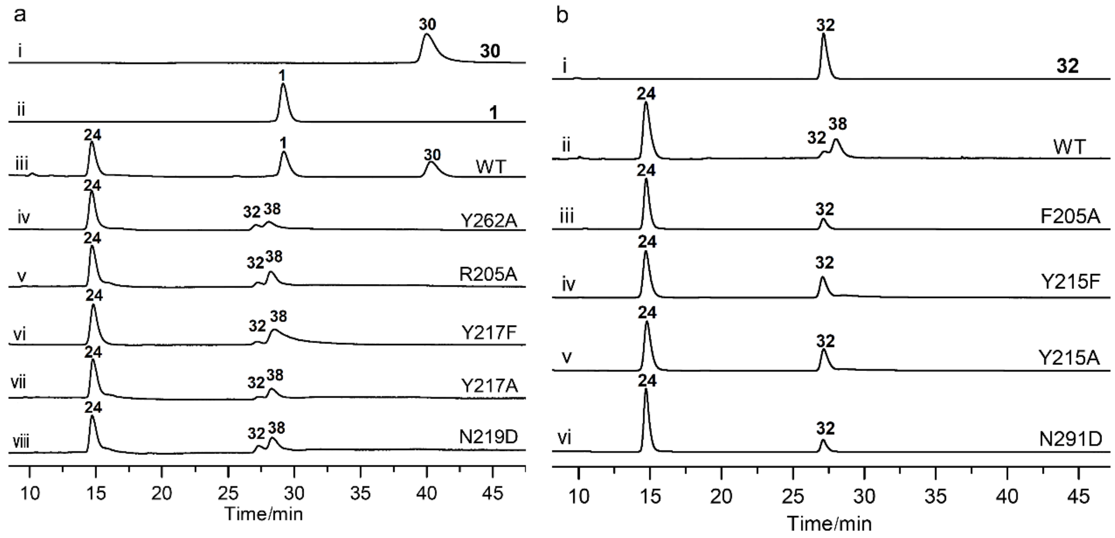

Supplementary Figure 130. In vitro characterization of the monooxygenase activity of active site variants of KnmB1 and KnmB2. (**a**) The reaction was conducted in the presence of **24**, SAM, NADPH, KnmB2 and KnmB1 variants, and KnmF was added after 30 min. (**b**) The reaction was conducted in the presence of **24**, SAM, NADPH, KnmB2 variants, and KnmF was added after 30 min. HPLC analysis was performed with analytical method C. The UV absorbance was monitored at 260 nm. A representative result of *n* = 3 independent experiments is shown. Source data are provided as a Source Data file.

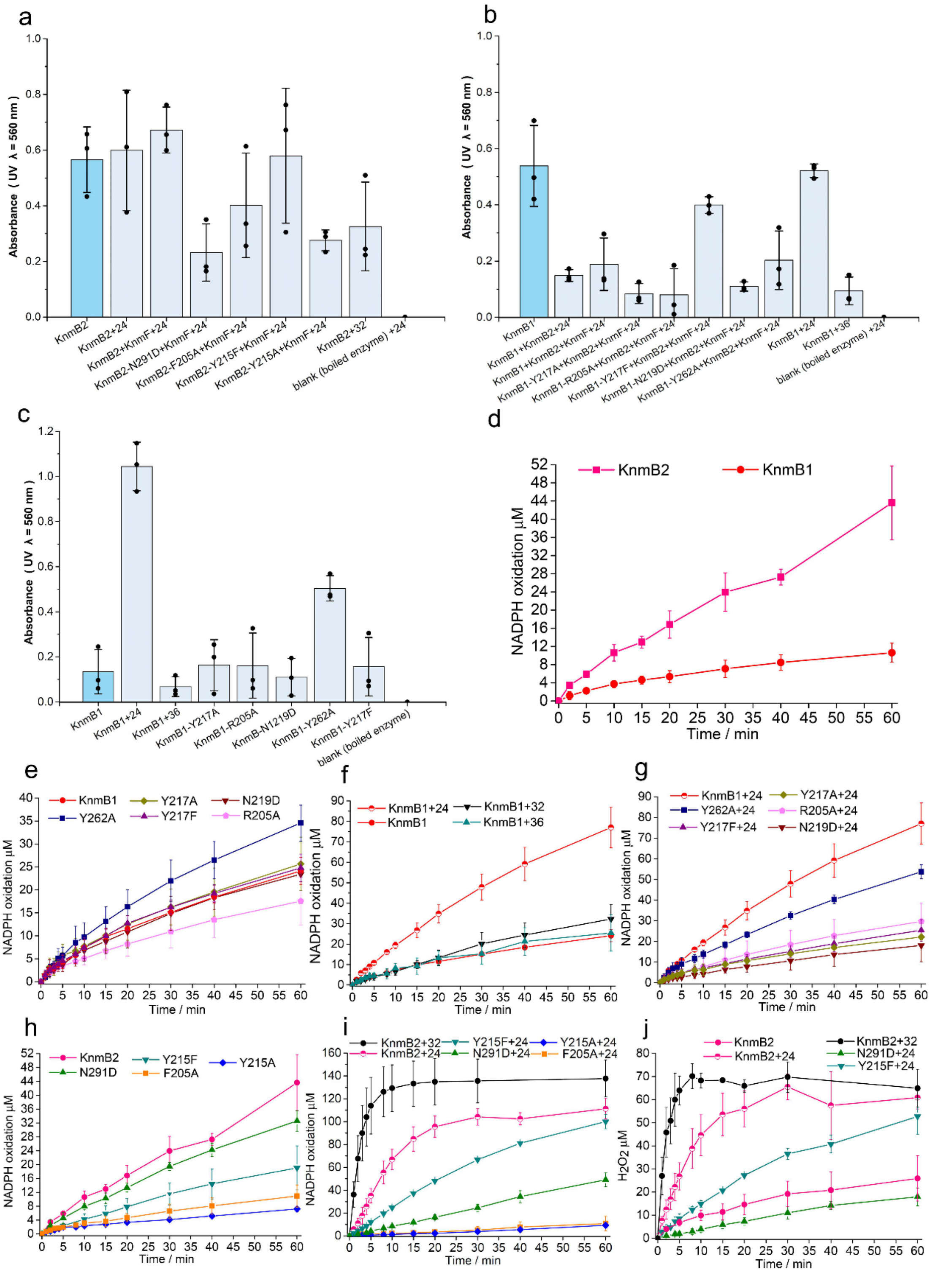

Supplementary Figure 131. H_2O_2 generation and NADPH oxidation for KnmB2, KnmB1 and their variants. (**a**) Hydrogen peroxide formation of KnmB2 and variants. Reaction conditions: 100 μL system containing 50 mM Tris-HCl buffer (pH 7.5), 1 mM NADPH, 16 μM KnmB2 or variants, 0.1 mM compound **24**, 1 mM SAM, and 2 μM KnmF at 30 °C for 3h. (**b**) Hydrogen peroxide formation of KnmB1 and variants. Reaction conditions: 100 μL system containing 50 mM Tris-HCl buffer (pH 7.5), 1 mM NADPH, 16 μM KnmB2, 17 μM KnmB1 or variants, 0.1 mM compound **24**,1 mM SAM, and 2

μM KnmF at 30 °C for 3h. (**c**) Hydrogen peroxide formation of KnmB1 and variants. Reaction conditions: 100μL system containing 50 mM Tris-HCl buffer (pH 7.5), 0.3 mM NADPH, and 6 μM KnmB1 or variants, or 0.1 mM **24** at 30 °C for 30 min. (**d**) NADPH oxidation of KnmB2 and KnmB1. Reaction conditions: 50 mM Tris-HCl buffer (pH 7.5), 0.15 mM NADPH, and 2 μM KnmB1 or KnmB2. (**e**) NADPH oxidation of KnmB1 and variants. (**f**) NADPH oxidation of KnmB1 in the presence **24**/**32**/**36**. (**g**) NADPH oxidation of KnmB1 and variants in the presence **24**. Reaction conditions (**e-g**) for KnmB1 and their variants: 50 mM Tris-HCl buffer (pH 7.5), 0.15 mM NADPH, and 6 μM KnmB1 or variants, or 0.1 mM substrate **24/32/36**. (**h**) NADPH oxidation of KnmB2 and variants. (**i**) NADPH oxidation of KnmB2 and variants in the presence **24** or **32**. (**j**) H_2O_2 generation for KnmB2 and variants in the presence **24** or **32**. Reaction conditions (**h-j**) for KnmB2 and their variants: 50 mM Tris-HCl buffer (pH 7.5), 0.15 mM NADPH, 2 μM KnmB2 or variants, or 0.1 mM substrate **24**/**32**. Values are the means ± SD of *n* = 3 independent experiments, except for KnmB2+**24** (T = 40 min), KnmB2-N219D+**24** (T = 4 min), KnmB2-Y215F+**24** (T= 1 min and 3 min) in **i** and **j**, and KnmB2+**32** (T= 8 min) in **j** (*n* = 2). Source data are provided as a Source Data file.

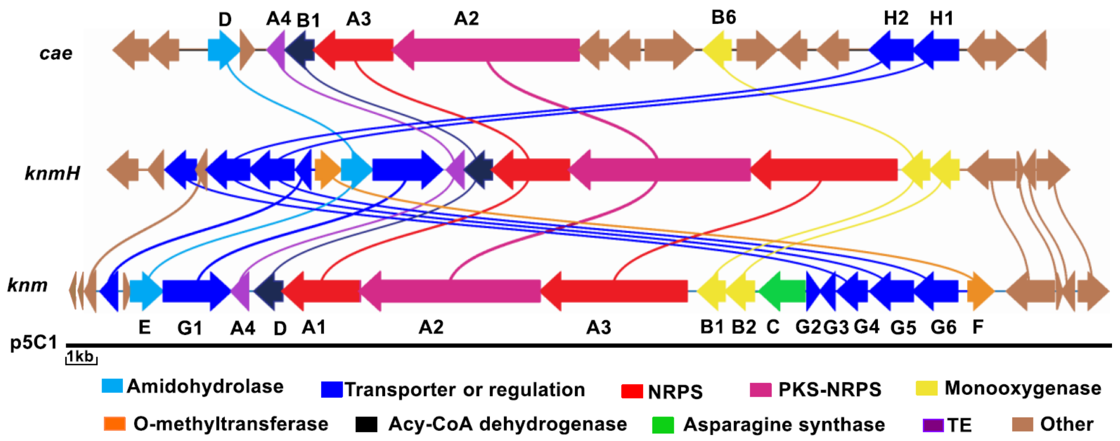

Supplementary Figure 132. Comparison of the organization of the *cae* BGC from *A. cyanogriseus* NRRL B-2194 (GenBank: JQ687072), *knmH* BGC from *L. aerocolonigenes* NBRC 13195 (GenBank: GCA_000974445.1) and *knm* BGC from *L. rhizosphaerae* NEAU-A2 (GenBank: OM436385-OM436408). The sequence alignment of gene clusters was created using Clustal Omega[16] and the figure was produced using EsPript 3.0[17].

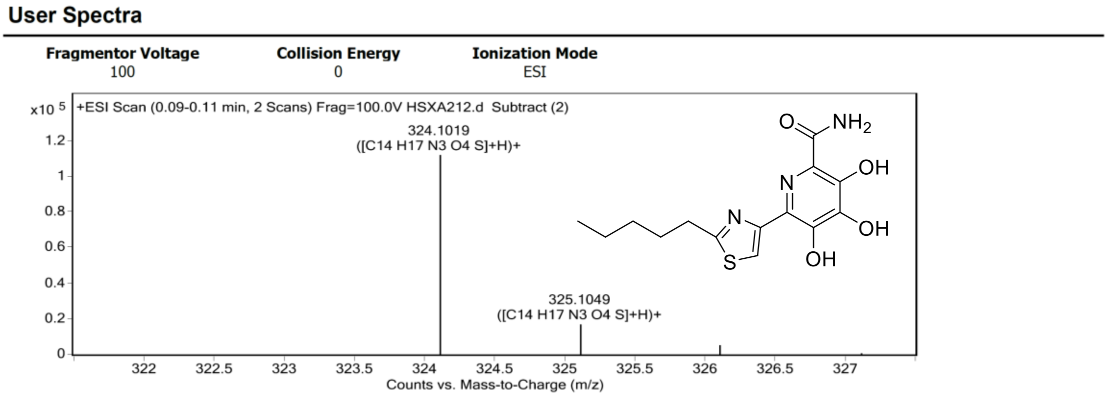

Supplementary Figure 133. HRESIMS spectrum of compound **29**.

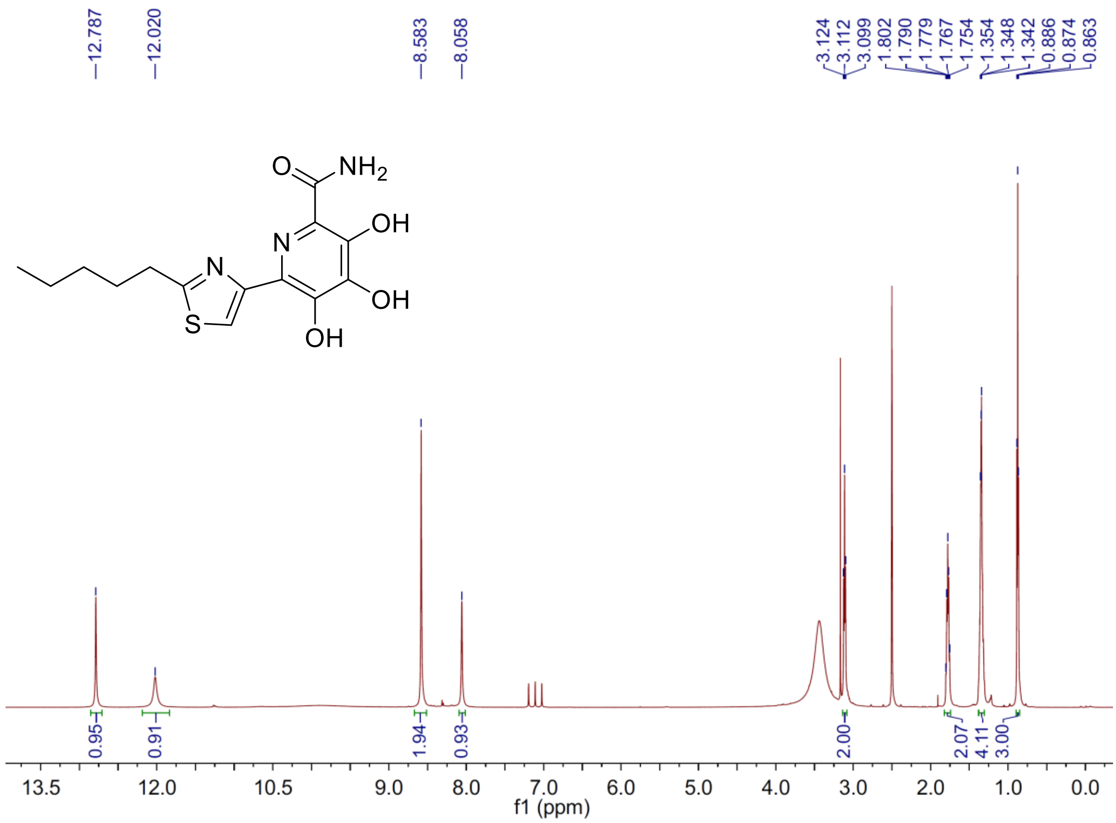

Supplementary Figure 134. ^{1}H-NMR (600 MHz) spectrum of compound **29** in DMSO-*d*$_6$.

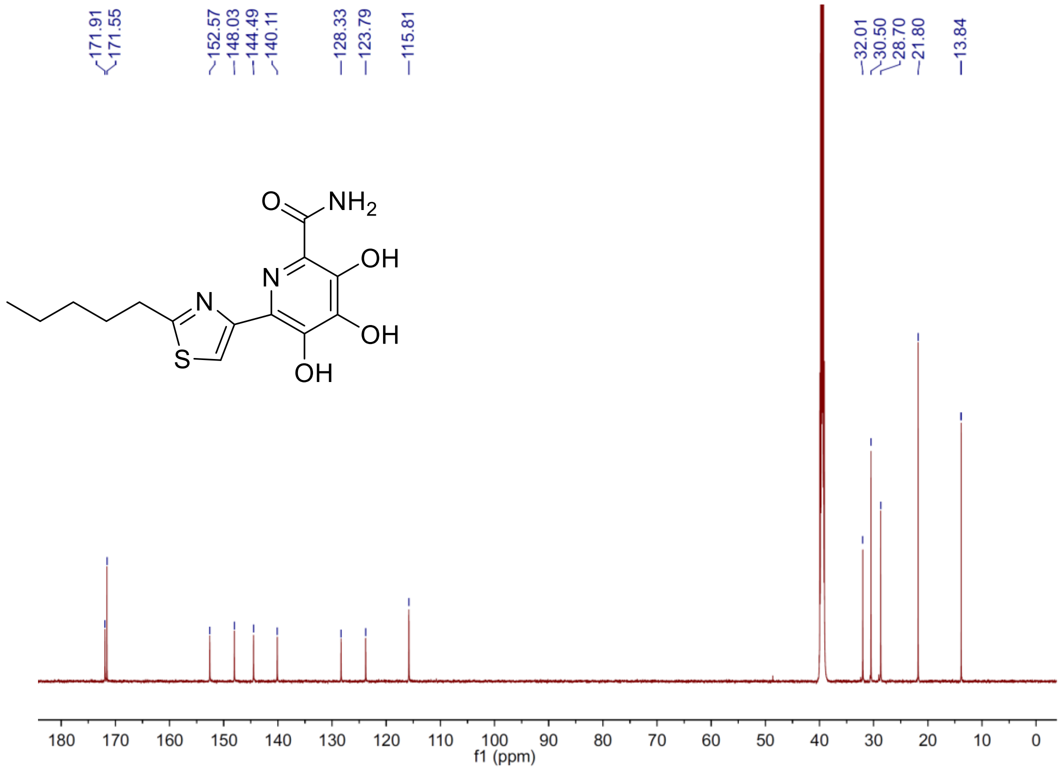

Supplementary Figure 135. ^{13}C-NMR (150 MHz) spectrum of compound **29** in DMSO-d_6.

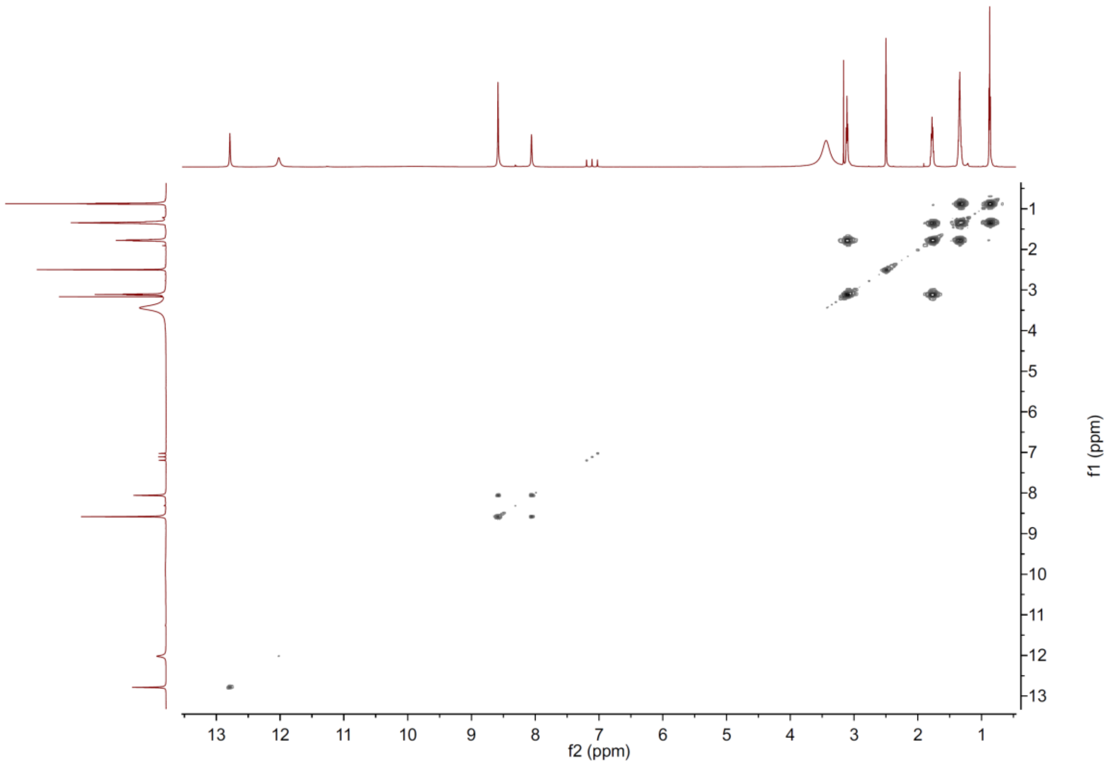

Supplementary Figure 136. ^{1}H-^{1}H COSY (600 MHz) spectrum of compound **29** in DMSO-d_6.

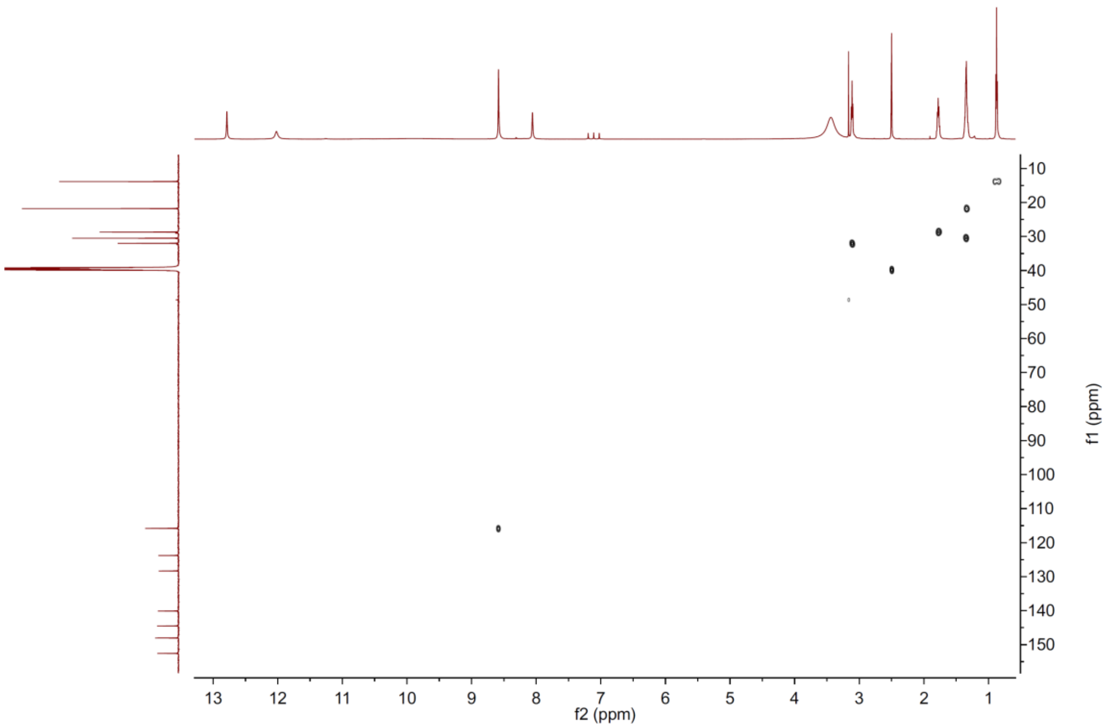

Supplementary Figure 137. HSQC (600 MHz) spectrum of compound **29** in DMSO-d_6.

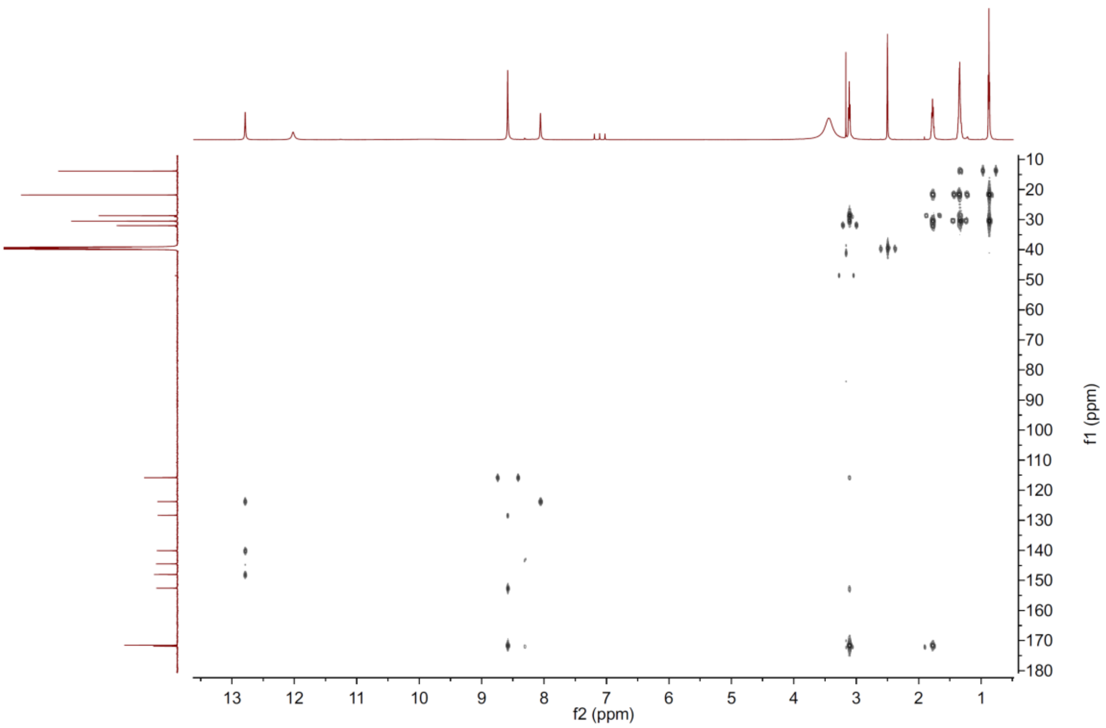

Supplementary Figure 138. HMBC (600 MHz) spectrum of compound **29** in DMSO-d_6.

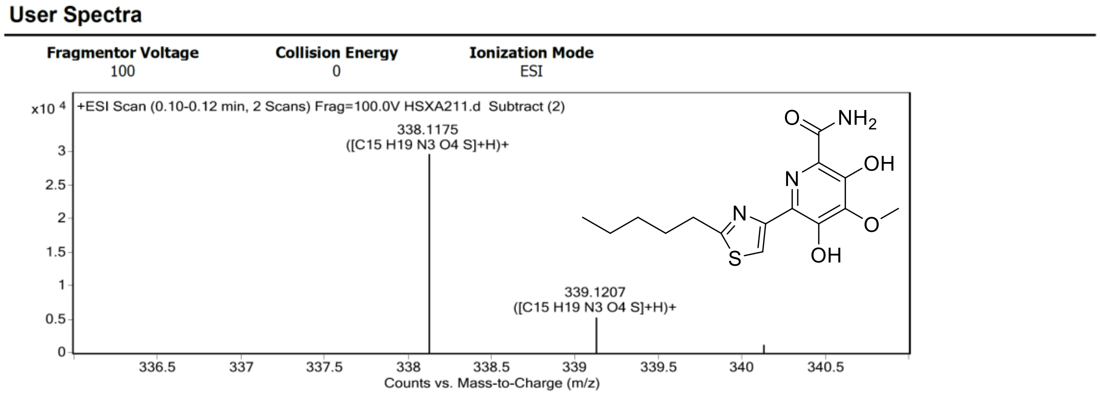

Supplementary Figure 139. HRESIMS spectrum of compound **31**.

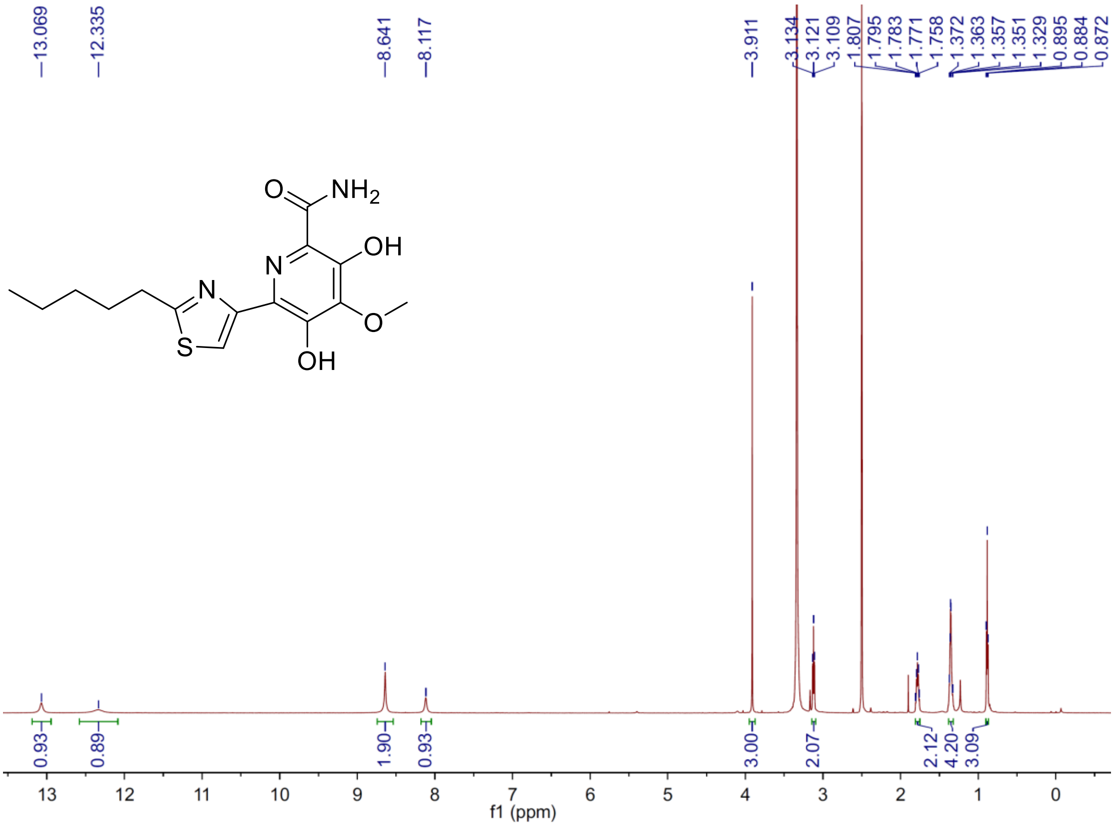

Supplementary Figure 140. ^{1}H-NMR (600 MHz) spectrum of compound **31** in DMSO-d_6.

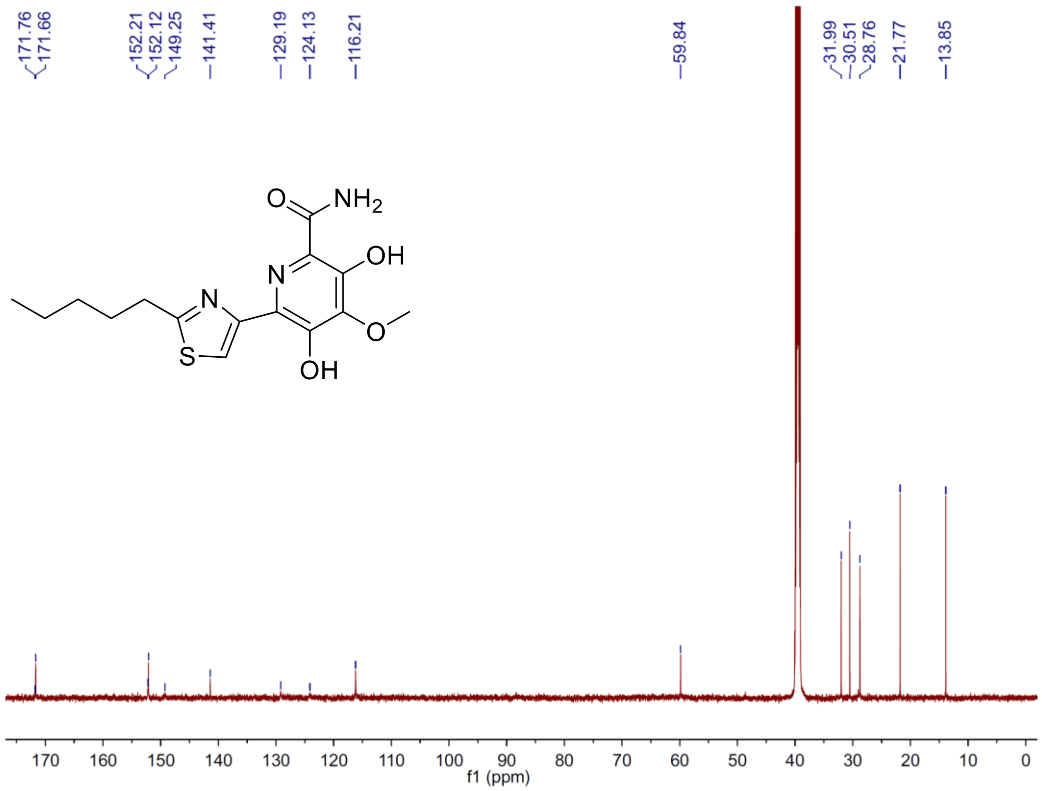

Supplementary Figure 141. ^{13}C-NMR (150 MHz) spectrum of compound **31** in DMSO-d_6.

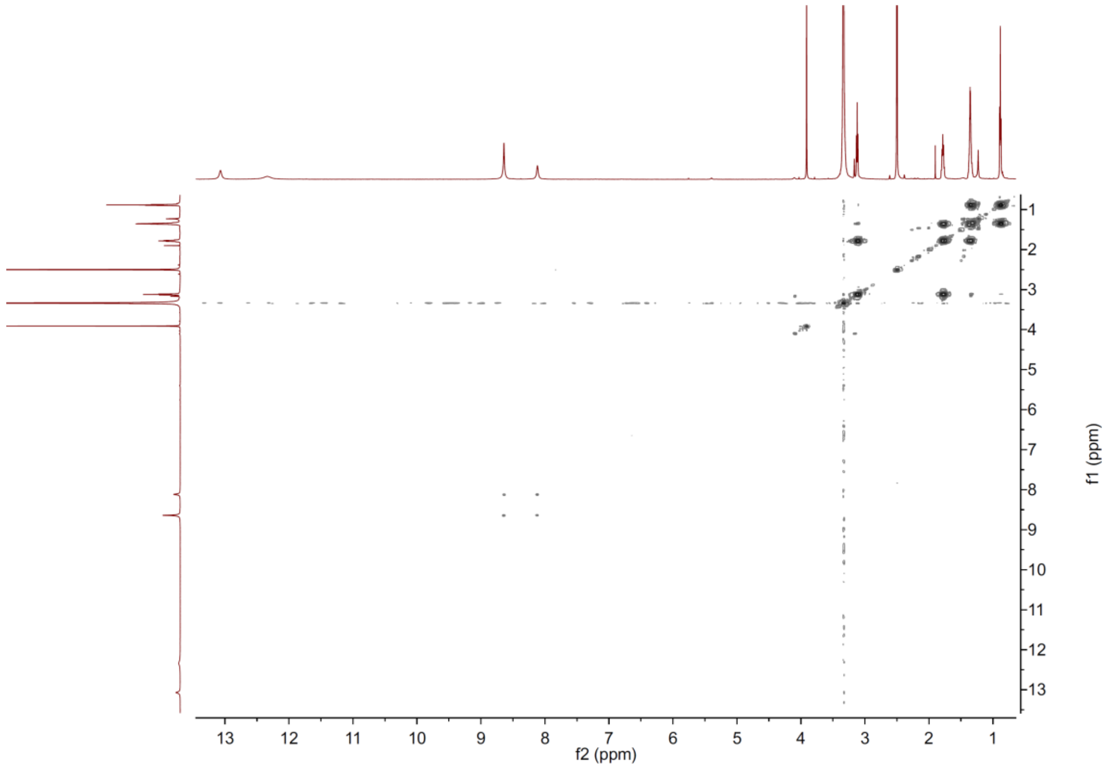

Supplementary Figure 142. ^{1}H-^{1}H COSY (600 MHz) spectrum of compound **31** in DMSO-d_6.

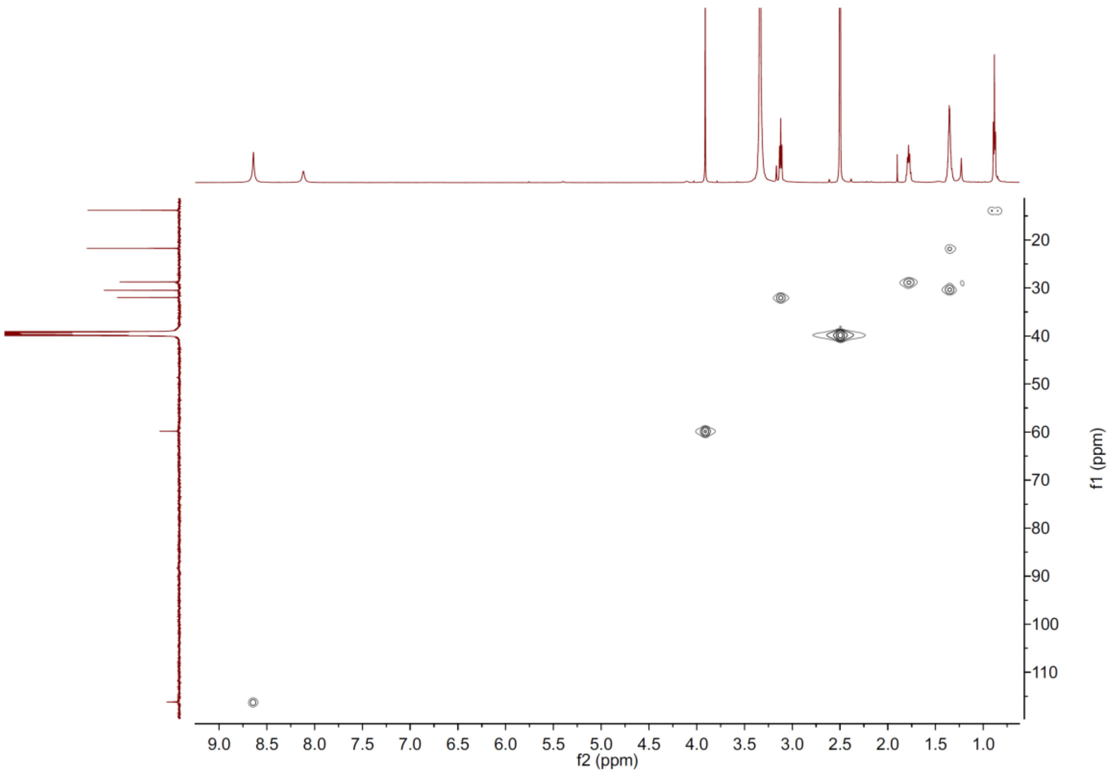

Supplementary Figure 143. HSQC (600 MHz) spectrum of compound **31** in DMSO-d_6.

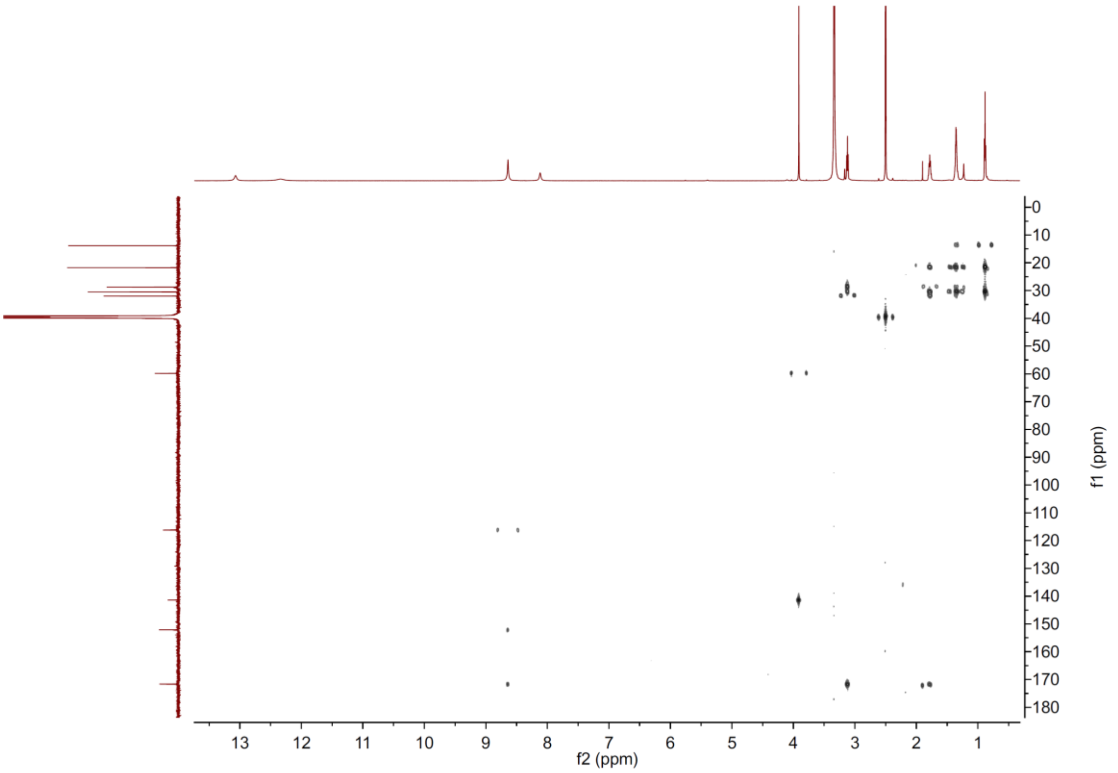

Supplementary Figure 144. HMBC (600 MHz) spectrum of compound **31** in DMSO-d_6.

User Spectra

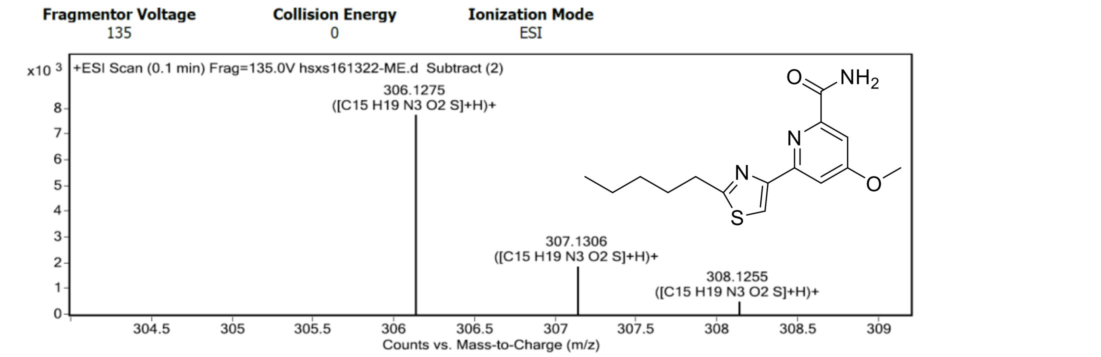

Supplementary Figure 145. HRESIMS spectrum of compound **34**.

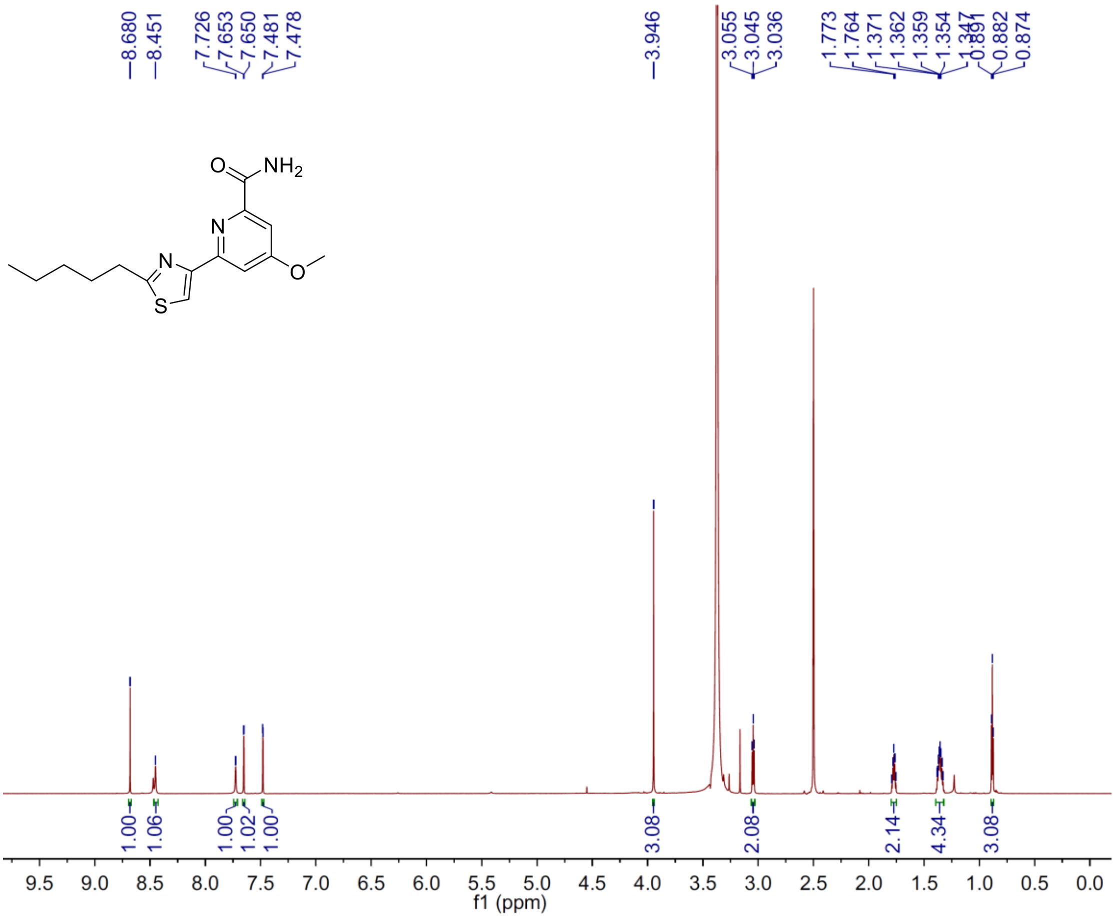

Supplementary Figure 146. ^{1}H-NMR (800 MHz) spectrum of compound **34** in DMSO-*d*$_6$.

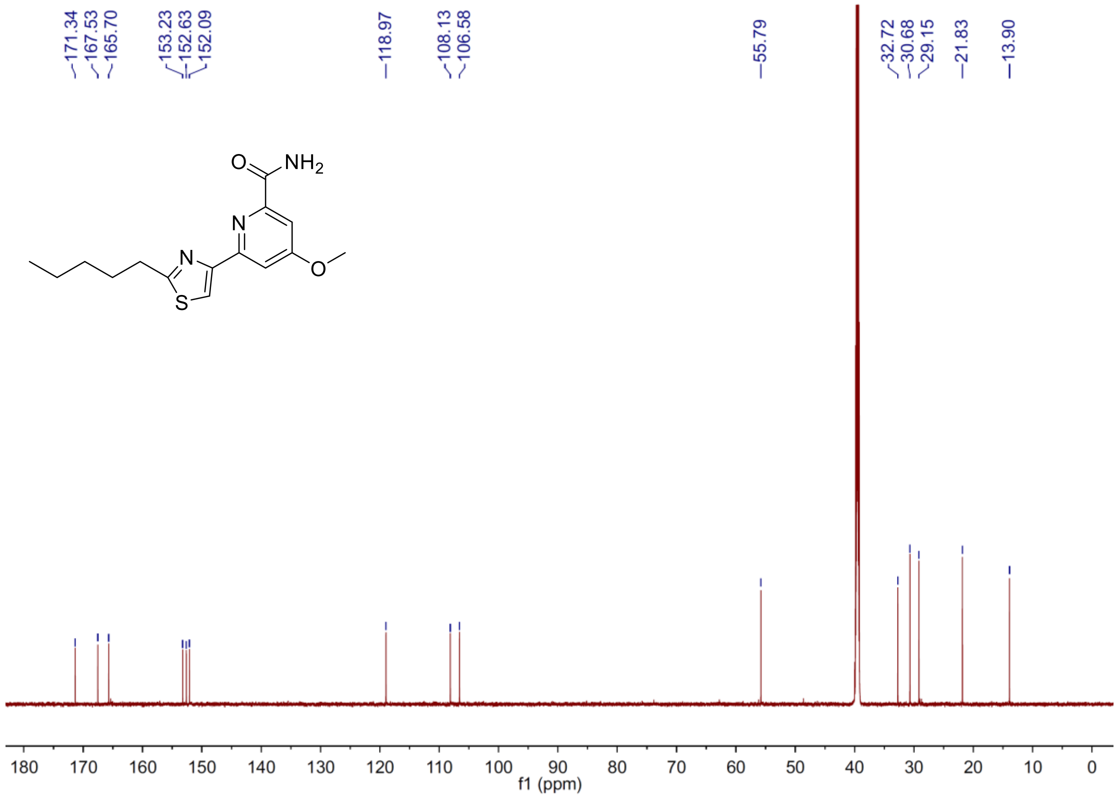

Supplementary Figure 147. ^{13}C-NMR (200 MHz) spectrum of compound **34** in DMSO-*d*$_6$.

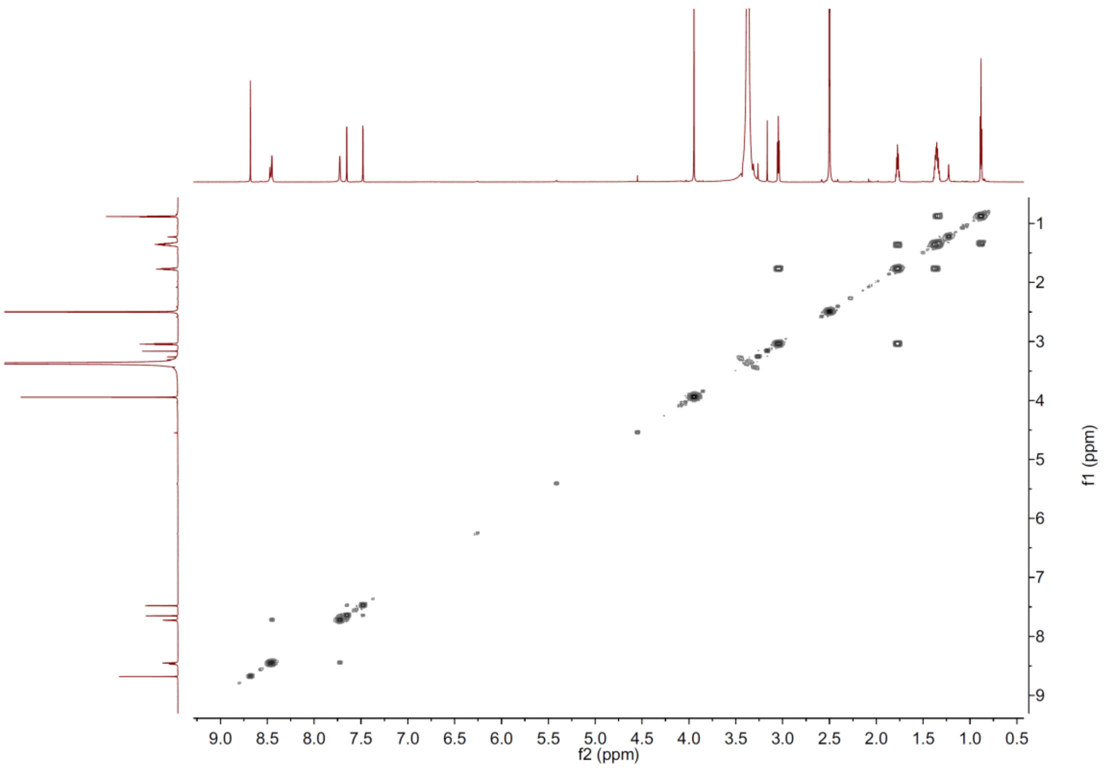

Supplementary Figure 148. ^{1}H-^{1}H COSY (800 MHz) spectrum of compound **34** in DMSO-*d*$_6$.

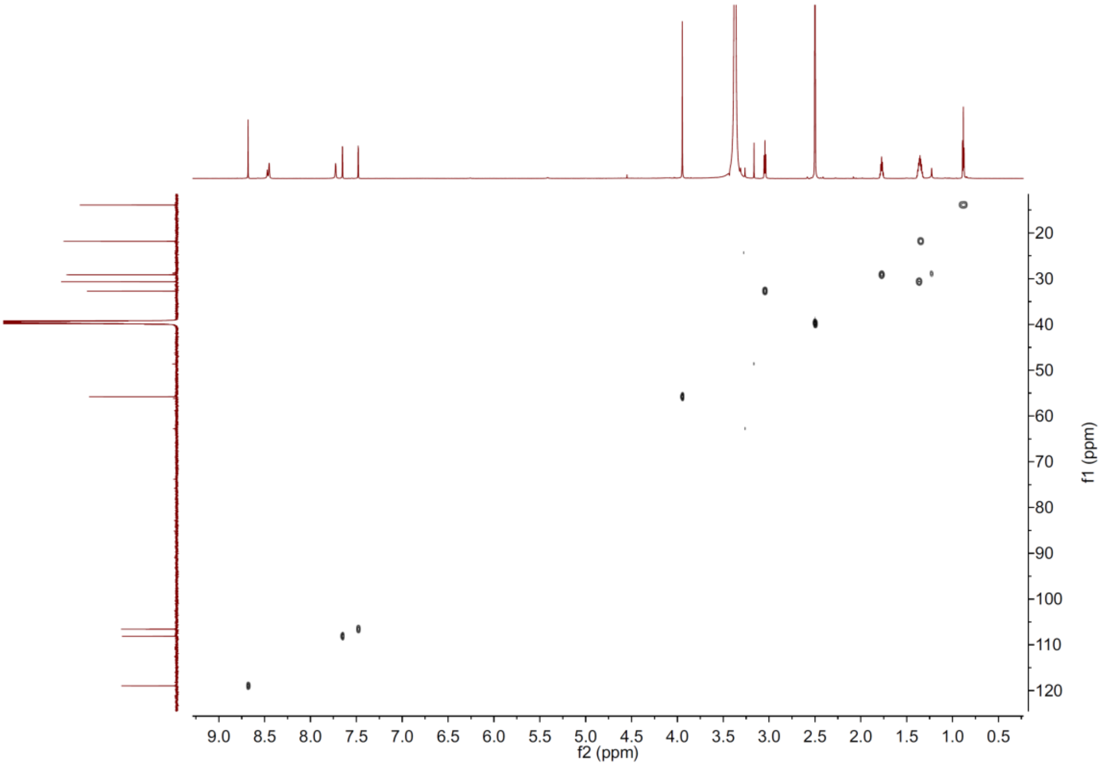

Supplementary Figure 149. HSQC (800 MHz) spectrum of compound **34** in DMSO-*d*$_6$.

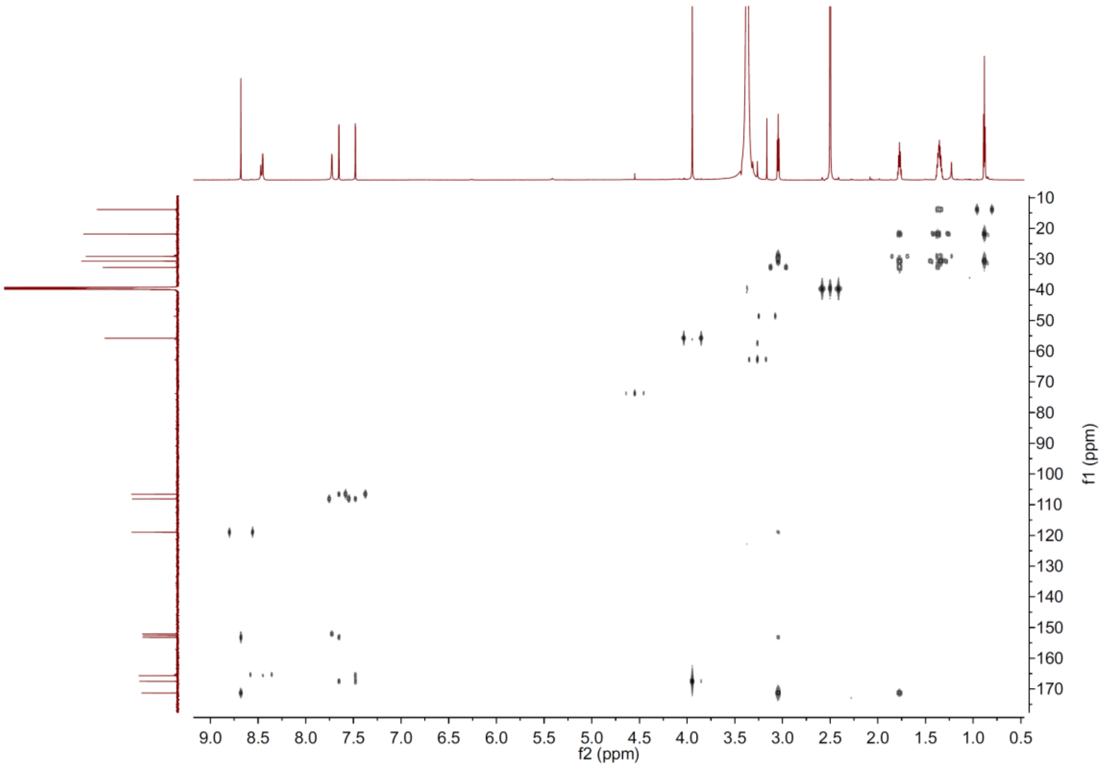

Supplementary Figure 150. HMBC (800 MHz) spectrum of compound **34** in DMSO-*d*$_6$.

Supplementary References

1. Deng, Y., Yang, C. H. & Smith Iii, A. B. Enantioselective total synthesis of (+)-peniciketals A and B: two Architecturally complex spiroketals. *J. Am. Chem. Soc.* **143**, 1740-1744 (2021).
2. Shen, A. L. & Kasper, C. B. Differential contributions of NADPH-cytochrome P450 oxidoreductase FAD binding site residues to flavin binding and catalysis. *J. Biol. Chem*. **275**, 41087-41091 (2000).
3. Chapman, S.K. & Reid, G. A. Flavoprotein Protocols. (Humana Press, Totowa, New Jersey, 1999).
4. Zhu, Z., Chen, Y., Shi, G. & Zhang, X. Selenium delays tomato fruit ripening by inhibiting ethylene biosynthesis and enhancing the antioxidant defense system. *Food Chemistry* **219**, 179-184 (2017).
5. Robinson, R. & Sobrado, P. Substrate binding modulates the activity of Mycobacterium smegmatis G, a flavin-dependent monooxygenase involved in the biosynthesis of hydroxamate-containing siderophores. *Biochemistry* **50**, 8489-8496 (2011).
6. Catucci, G., Gao, C., Rampolla, G., Gilardi, G. & Sadeghi, S.J. Uncoupled human flavin-containing monooxygenase 3 releases superoxide radical in addition to hydrogen peroxide. *Free Radic. Biol. Med*. **145**, 250-255 (2019).
7. Paget, M. S., Chamberlin, L., Atrih, A., Foster, S. J. & Buttner, M. J. Evidence that the extracytoplasmic function sigma factor sigmaE is required for normal cell wall structure in *Streptomyces coelicolor* A3(2). *J. Bacteriol.* **181**, 204-211 (1999).
8. Li, L. et al. The mildiomycin biosynthesis: initial steps for sequential generation of 5-hydroxymethylcytidine 5'-monophosphate and 5-hydroxymethylcytosine in *Streptoverticillium rimofaciens* ZJU5119. *Chembiochem* **9**, 1286-1294 (2008).
9. Datsenko, K. A. & Wanner, B. L. One-step inactivation of chromosomal genes in *Escherichia coli* K-12 using PCR products. *Proc. Natl. Acad. Sci. USA* **97**, 6640-6645 (2000).
10. Chater, K. F. & Wilde, L. C. *Streptomyces albus* G mutants defective in the SalGI restriction-modification system. *J. Gen. Microbiol*. **116**, 323-334 (1980).
11. Yan, Y. et al. Non-enzymatic pyridine ring formation in the biosynthesis of the rubrolone tropolone alkaloids. *Nat. Commun*. **7**, 13083 (2016).
12. Gust, B., Challis, G. L., Fowler, K., Kieser, T. & Chater, K. F. PCR-targeted *Streptomyces* gene replacement identifies a protein domain needed for biosynthesis of the sesquiterpene soil odor geosmin. *Proc. Natl. Acad. Sci. USA* **100**, 1541-1546 (2003).
13. Kumar, S., Stecher, G. & Tamura, K. MEGA7: molecular evolutionary genetics analysis version 7.0 for bigger datasets. *Mol. Biol. Evol*. **33**,1870-1874 (2016).
14. Saitou, N. & Nei M. The neighbor-joining method: A new method for reconstructing phylogenetic trees. *Mol. Biol. Evol.* **4**, 406-425 (1987).
15. Felsenstein, J. Confidence limits on phylogenies: An approach using the bootstrap. *Evolution* **39**, 783-791 (1985).
16. Sievers, F. & Higgins, D. G. The clustal omega multiple alignment package. *Methods Mol. Biol.* **2231**, 3-16 (2021).

17. Gouet, P., Robert, X. & Courcelle, E. ESPript/ENDscript: extracting and rendering sequence and 3D information from atomic structures of proteins. *Nucleic Acids Res*. **31**, 3320-3323 (2003).
