# Supplementary material for: Discovery and biosynthesis of karnamicins as angiotensin converting enzyme inhibitors
Source: Nat Commun. 2023 Jan 13;14:209. doi: 10.1038/s41467-023-35829-1 (PMC9838390; doi:10.1038/s41467-023-35829-1)
Supplement: Supplementary file 2 — Reporting Summary [file 41467_2023_35829_MOESM2_ESM.pdf]

nature portfolio

Corresponding author(s): Wensheng Xiang, Sheng-Xiong Huang

Last updated by author(s): Dec 23, 2022

Reporting Summary

Nature Portfolio wishes to improve the reproducibility of the work that we publish. This form provides structure for consistency and transparency in reporting. For further information on Nature Portfolio policies, see our Editorial Policies and the Editorial Policy Checklist.

Statistics

For all statistical analyses, confirm that the following items are present in the figure legend, table legend, main text, or Methods section.

n/a	Confirmed	
☐	☒	The exact sample size (*n*) for each experimental group/condition, given as a discrete number and unit of measurement
☐	☒	A statement on whether measurements were taken from distinct samples or whether the same sample was measured repeatedly
☒	☐	The statistical test(s) used AND whether they are one- or two-sided<br>*Only common tests should be described solely by name; describe more complex techniques in the Methods section.*
☒	☐	A description of all covariates tested
☒	☐	A description of any assumptions or corrections, such as tests of normality and adjustment for multiple comparisons
☐	☒	A full description of the statistical parameters including central tendency (e.g. means) or other basic estimates (e.g. regression coefficient) AND variation (e.g. standard deviation) or associated estimates of uncertainty (e.g. confidence intervals)
☒	☐	For null hypothesis testing, the test statistic (e.g. *F*, *t*, *r*) with confidence intervals, effect sizes, degrees of freedom and *P* value noted<br>*Give P values as exact values whenever suitable.*
☒	☐	For Bayesian analysis, information on the choice of priors and Markov chain Monte Carlo settings
☒	☐	For hierarchical and complex designs, identification of the appropriate level for tests and full reporting of outcomes
☒	☐	Estimates of effect sizes (e.g. Cohen's *d*, Pearson's *r*), indicating how they were calculated

Our web collection on statistics for biologists contains articles on many of the points above.

Software and code

Policy information about availability of computer code

Data collection	Chromaster was used for HPLC data collection. NanoDrop 2000c was used for collecting the content data of FAD, NADPH and H2O2.
Data analysis	MEGA version 7 was used for amino acid alignment and phylogenetic tree construction. OriginPro 9.0 was used for HPLC and NanoDrop data visualization. ChemBioDraw Ultra 20.0 was used for drawing chemical structures (Fig. 1, Fig. 2 and Fig. 3). MestReNova 6.1 was used for NMR data analysis. PyMOL 2.5.2 was used for protein structure visualization. GraphPad Prism 7.0. was used for estimating the IC50 values. Molecular docking was performed using Autodock Vina 1.2.2. The ligands used for molecular docking (substrates 24, 27, and FAD) were generated using ChemBio3D Ultra 12.0. The sequence alignment of gene clusters was created using Clustal Omega and the figure was produced using EsPript 3.0. GeneMarkS Version 2.0 was used for the prokaryotic genome annotation. antiSMASH 4.0 and PRISM 3 were used for genome mining. AlphaFold was used for protein structures prediction.

For manuscripts utilizing custom algorithms or software that are central to the research but not yet described in published literature, software must be made available to editors and reviewers. We strongly encourage code deposition in a community repository (e.g. GitHub). See the Nature Portfolio guidelines for submitting code & software for further information.

Data

Policy information about availability of data

All manuscripts must include a data availability statement. This statement should provide the following information, where applicable:

- Accession codes, unique identifiers, or web links for publicly available datasets
- A description of any restrictions on data availability
- For clinical datasets or third party data, please ensure that the statement adheres to our policy

The sequence data for ORFs in knm cluster of L. rhizosphaerae NEAU-A2 have been deposited in GenBank with accession codes OM436385(https://

www.ncbi.nlm.nih.gov/nuccore/OM436385), OM436386 (https://www.ncbi.nlm.nih.gov/nuccore/OM436386), OM436387 (https://www.ncbi.nlm.nih.gov/nuccore/OM436387), OM436388 (https://www.ncbi.nlm.nih.gov/nuccore/OM436388), OM436389 (https://www.ncbi.nlm.nih.gov/nuccore/OM436389), OM436390 (https://www.ncbi.nlm.nih.gov/nuccore/OM436390), OM436391 (https://www.ncbi.nlm.nih.gov/nuccore/OM436391), OM436392 (https://www.ncbi.nlm.nih.gov/nuccore/OM436392), OM436393 (https://www.ncbi.nlm.nih.gov/nuccore/OM436393), OM436394 (https://www.ncbi.nlm.nih.gov/nuccore/OM436394), OM436395 (https://www.ncbi.nlm.nih.gov/nuccore/OM436395), OM436396 (https://www.ncbi.nlm.nih.gov/nuccore/OM436396), OM436397 (https://www.ncbi.nlm.nih.gov/nuccore/OM436397), OM436398 (https://www.ncbi.nlm.nih.gov/nuccore/OM436398), OM436399 (https://www.ncbi.nlm.nih.gov/nuccore/OM436399), OM436400 (https://www.ncbi.nlm.nih.gov/nuccore/OM436400), OM436401 (https://www.ncbi.nlm.nih.gov/nuccore/OM436401), OM436402 (https://www.ncbi.nlm.nih.gov/nuccore/OM436402), OM436403 (https://www.ncbi.nlm.nih.gov/nuccore/OM436403), OM436404 (https://www.ncbi.nlm.nih.gov/nuccore/OM436404), OM436405 (https://www.ncbi.nlm.nih.gov/nuccore/OM436405), OM436406 (https://www.ncbi.nlm.nih.gov/nuccore/OM436406), OM436407 (https://www.ncbi.nlm.nih.gov/nuccore/OM436407), and OM436408 (https://www.ncbi.nlm.nih.gov/nuccore/OM436408). Crystallographic data for the structure of 7 reported in this Article have been deposited at the Cambridge Crystallographic Data Centre, under deposition number CCDC 2143094 (https://www.ccdc.cam.ac.uk/structures/Search?Ccdcid=2143094&DatabaseToSearch=Published). Copies of the data can be obtained free of charge via https://www.ccdc.cam.ac.uk/structures/. All data that support the findings of this study are available in the main text and the supplementary information. Plasmids generated in this study are available from the corresponding author. Source data are provided with this paper.

Field-specific reporting

Please select the one below that is the best fit for your research. If you are not sure, read the appropriate sections before making your selection.

☒ Life sciences ☐ Behavioural & social sciences ☐ Ecological, evolutionary & environmental sciences

For a reference copy of the document with all sections, see nature.com/documents/nr-reporting-summary-flat.pdf

Life sciences study design

All studies must disclose on these points even when the disclosure is negative.

Sample size	No sample size calculation was performed. Replicates of n≥2 were chosen for PCR verifications, enzyme activity assays, SDS-PAGE analysis of proteins and ACE inhibitory assays, as they are sufficient for us to assess the presence/absence of a band (gene or protein), a peak (representative of a metabolite), or inhibitory activity.
Data exclusions	No data was excluded.
Replication	All PCR verifications, enzyme assays, protein analysis and ACE inhibitory activity experiments were verified with at least two independent replicates. All attempts at replication were successful.
Randomization	Double crossover mutant strains were randomly selected for metabolic extracts analysis after PCR verification.
Blinding	Complete blinding was not feasible for this study, as samples were prepared and measured by the same researcher. Besides, these experiments required the insight of the researcher into the enzymes/genes and/or metabolites being analyzed. Blinding was performed during the ACE inhibitory activity assay.

Reporting for specific materials, systems and methods

We require information from authors about some types of materials, experimental systems and methods used in many studies. Here, indicate whether each material, system or method listed is relevant to your study. If you are not sure if a list item applies to your research, read the appropriate section before selecting a response.

Materials & experimental systems

n/a	Involved in the study	
☒	☐	Antibodies
☒	☐	Eukaryotic cell lines
☒	☐	Palaeontology and archaeology
☒	☐	Animals and other organisms
☒	☐	Human research participants
☒	☐	Clinical data
☒	☐	Dual use research of concern

Methods

n/a	Involved in the study	
☒	☐	ChIP-seq
☒	☐	Flow cytometry
☒	☐	MRI-based neuroimaging
